# The Effect of Different Pollination on the Expression of Dangshan Su Pear MicroRNA

**DOI:** 10.1155/2017/2794040

**Published:** 2017-04-10

**Authors:** Xi Cheng, Chongchong Yan, Jinyun Zhang, Chenhui Ma, Shumei Li, Qing Jin, Nan Zhang, Yunpeng Cao, Yi Lin, Yongping Cai

**Affiliations:** ^1^School of Life Science, Anhui Agricultural University, Changjiang West Road, No. 130, Hefei 230036, China; ^2^Horticultural Institute, Anhui Academy of Agricultural Sciences, Hefei, Anhui 230031, China

## Abstract

The high-throughput sequencing of pear “Dangshan Su” × “Yali” (whose fruits lignin and stone cell content are high and quality is poor) and pear “Dangshan Su” × “Wonhwang” (whose fruits with low content of lignin and stone cell and the quality are better ) found that the expressions of these two miRNAs (pyr-1809 and pyr-novel-miR-144-3p) were significantly different; their corresponding target genes encode two kinds of laccase (Pbr018935.1 and Pbr003857.1). qRT-PCR results showed that these two enzymes are involved in the formation of lignin and stone cells and the existence of these two miRNAs has a negative effect on them. It was concluded that the effect of pollination on the development of stone cells may affect the synthesis of lignin, through the regulation of laccase controlled by miRNAs, and ultimately affect the formation of stone cell and fruit quality.

## 1. Introduction

MicroRNAs (miRNAs) are small, endogenous, noncoding 20–25 nucleotide (nt) small RNAs [1, 2] that negatively control gene expression by cleaving or inhibiting the translation of target gene transcripts [1, 3–6]. Since miRNAs were first reported in 1993 [7], more than 35,000 mature miRNAs have been identified from 221 species including* Capra hircus, Salmo salar*, and* Plutella xylostella* (miRBase release 21, June 2014, http://www.mirbase.org/) [8, 9]. Plant microRNAs are released as a duplex from their primary transcripts (primiRNAs) containing stem-loop structures by RNase III enzymes. In the miRNA duplex, miRNA (guide strand) associates with argonaute (AGO) proteins to inhibit gene expression through cleavage and/or translational inhibition of target RNAs, while miRNA^*∗*^ (passenger strand) is often degraded [3–6]. A number of studies have suggested that miRNAs play an important role in regulating plant development [10–12], secondary metabolism, and diverse responses to stresses [13, 14]. Kaja et al. [15] reported that mdm-miR169a, mdm-miR160e, mdm-miR167b-g, and mdm-miR168a-b were involved in fire blight resistance in apple trees. Eldem et al. [16] validated that miRNAs (miR156, miR164, miR166, miR168, miR169, miR171, and miR395) were detected in drought responses in* Prunus persica*. In* Populus trichocarpa* [17], ptr-miR397a was identified as a negative regulator of laccase genes during the biosynthetic pathway of lignin.

*Pyrus bretschneideri* cv. Dangshan Su is a Chinese pear variety, widely grown across China and other Asian countries [18]. It is self-incompatible plants with obvious xenia phenomenon. And if undergoing self-pollination, it would not produce any fruits. Furthermore, different styles of pollination can also affect its fruit quality to a great extent [19]. In recent years, due to the deterioration of varieties and inappropriate choices of pollination tree, its fruit flavor and quality were significantly decreased. One of the most prominent manifestations is the increase in the content of stone cells, resulting in rough flesh and poor taste. It has been clarified that the stone cells are formed by the thickening of the secondary wall of the cell wall of the pulp cells and lignifications. Lignin is one of the main components of stone cells; lignin biosynthesis is closely related to the development of stone cell [20].

In recent years, it has been reported that miRNAs were widely involved in the regulation of pear fruit development and fruit quality, via sugar and acid metabolism and hormone signaling [21]. And no relevant report has revealed the molecular mechanism underlying different styles of pollination on pear fruits. In addition, small RNAs have been annotated for pear genome [22], and the genome wide identification of pear miRNAs was recently reported [21, 23]. However, the role of microRNA for regulatory functions is unknown in Dangshan Su pear undergoing different styles of pollination, as well as the specific microRNAs which are affected by xenia phenomenon and involved in the regulation of lignin metabolism and stone cell formation in pear fruit. Previous studies have learned “Yali” (*P. bretschneideri* Rehd.) as male parent, whose fruit lignin and stone cell content are high and quality is poor, while the fruits with low content of lignin and stone cell have better quality, using “Wonhwang” (*P. pyrifolia* Nakai) as the male parent. Thus, in this study, we used “Yali” and “Wonhwang” to pollinate “Dangshan Su” and built “Dangshan Su” microRNA expression profiles under different pollen donators using Solexa high-throughput sequencing technology. With bioinformatics method, the xenia phenomenon effect on “Dangshan Su” microRNA expression levels was analyzed to predict differently expressed microRNAs and their corresponding target genes. Furthermore, the lignin metabolism-related enzyme genes and its corresponding microRNA expression changes were determined using Poly (T) method and real-time fluorescent quantitative PCR (qRT-PCR) assay at different developmental stages of “Dangshan Su” × “Yali” and “Dangshan Su” × “Wonhwang”, respectively. The above researches can lay a foundation for the regulation of lignin and stone cell formation and improve the quality of pear fruit by exploring the related miRNA which control the lignin synthesis and stone cell development, which also provide a certain theoretical basis and technical methods for subsequent research on xenia affecting fruit quality by regulating miRNA expression in which way. These results also could provide an important theoretical basis for the screening of suitable “Dangshan Su” pollinated varieties and the improvement of “Dangshan Su” fruit quality.

## 2. Materials and Methods

### 2.1. Materials

Fruits were obtained from 50-year-old pear trees grown on an orchard localized at Dangshan County, Anhui, China. The “Dangshan Su” female parents were fertilized with pollens from “Yali” (*P. bretschneideri* Rehd.) and “Wonhwang” (*P. pyrifolia* Nakai) male parents. Pollens were collected from “Yali” and “Wonhwang” flower androecia and used to manually pollinate “Dangshan Su,” respectively. At the end of pollination processing, flowers were covered with bags for seven days. Pear fruits of different male parent pollination were collected after pollination at 23 days after flowering and the samples were taken every 8 days (120 pear fruits were taken from 47 d, 63 d, 79 d, and 145 d after flowering and 40 pears at other times) and stored at −80°C in our laboratory at Anhui Agricultural University, China.

### 2.2. Small RNA Sequencing and Library Construction

For each group, total RNA was extracted using the pBiozol Total RNA Extraction Reagent (BioFlux) following the manufacturer's instructions. All RNA samples were examined for protein contamination (A260/A280 ratios) and reagent contamination (A260/A230 ratios) by using a Nanodrop ND 1000 spectrophotometer (NanoDrop, Wilmington, DE). Before library construction, the RNAs of the “DangshanSu” × “Yali” (DangsuA) or “DangshanSu” × “Wonhwang” (DangsuB) were pooled together to form sample groups, respectively.

For library construction, total RNA was firstly resolved on denatured 15% polyacrylamide gels and 18–30 nt gel fragments were excised. A pair of adaptors was ligated to both ends of the eluted small RNA fragments. Subsequently, the adapter-ligated small RNAs were reversely transcribed into cDNA with Super-Script II Reverse Transcriptase (Invitrogen) and amplified by PCR. Finally, the resulting library was quantified and sequenced by using the Illumina HiSeq 2000 platform (BGI, Shenzhen, China).

### 2.3. Sequencing Data Analysis

For the raw data, all low quality reads, such as reads with adapter contaminants or poly A sequences, were filtered. After sequencing, the sequences that are longer than 30 nt or shorter than 18 nt and without adapters were removed, the remaining ones were used for subsequent analysis.

The clean sequencing data were mapped into two genome and Rfam database v11 (http://www.sanger.ac.uk/Software/Rfm/). Reads aligned in the genome, excluding those matching tRNAs, rRNAs, snRNA, and snoRNAs, were used for further analysis. Mature miRNAs and their precursors were retrieved from miRBase (version 21; http://www.mirbase.org) [24, 25].

### 2.4. miRNA Identification and Quantitation

The remaining reads were applied to predicting novel miRNAs and quantitative analysis using the miRDeep2 with the parameters for plants described in miREvo. To assess the reliability of predicted novel microRNAs, their MFEI (minimum folding free energy index) were calculated to compare with the known microRNAs [26–28].

The frequency of microRNAs from different libraries was normalized by total clean reads of microRNAs in each sample group. If the normalized read count of a given microRNA is zero, the expression value was modified to 1 for further analysis.

### 2.5. Identification of Target Genes for Differentially Expressed miRNAs

The expression differences between small RNA libraries of both samples were determined using EdgeR package with R language. Then, tools named “TAPIR” and “TargetFinder” were used for the microRNAs target prediction, which was evaluated with a better prediction performance [29]. The target RNA sequences were parsed from pear genomes with their corresponding annotation files (http://peargenome.njau.edu.cn/) [22].

### 2.6. Functional Enrichment and Cluster Analysis for Differential miRNAs' Target Transcripts

In order to understand the function of the differentially expressed genes, hyper geometric distribution test was carried out to identify GO functions and KEGG pathways in which differentially expressed genes are significantly enriched *p* value < 0.05, comparing total transcripts. This method firstly maps all target gene candidates to GO terms in the database (http://www.geneontology.org/), calculating gene numbers for each term, then using hypergeometric test to find significantly enriched GO terms in target gene candidates comparing to the reference gene background. The calculating formula is

(1) $$\begin{array}{c} P=1-\overset{m-1}{\underset{i=0}{\sum }}\frac{\left(\begin{array}{c} M\\ i \end{array}\right)\left(\begin{array}{c} N-M\\ n-i \end{array}\right)}{\left(\begin{array}{c} N\\ n \end{array}\right)}. \end{array}$$

In the formula above, *N* is the number of all genes with GO annotation; *n* is the number of target gene candidates in *N*; *M* is the number of all genes that are annotated to a certain GO term; and *m* is the number of target gene candidates in *M*. We used the Bonferroni Correction for the *p* value to obtain a corrected *p* value. GO terms with corrected *p* value ≤ 0.05 are defined as significantly enriched in target gene candidates.

KEGG calculating formula is the same as that in GO analysis. Here *N* is the number of all genes with KEGG annotation, *n* is the number of target gene candidates in *N*, *M* is the number of all genes annotated to a certain pathway, and m is the number of target gene candidates in *M*. Genes with FDR ≤ 0.05 are considered as significantly enriched in target gene candidates. The KEGG analysis could reveal the main pathways which the target gene candidates are involved in.

And differentially expressed genes hierarchically clustered to represent the expression patterns by using ward method with Euclidean distance as a measurement of similarity. All above analyses were programmed in python 2.7.5 with numpy (1.9.2), scipy (0.15.1), and matplotlib (1.4.3).

### 2.7. Extraction and qRT-PCR Analysis for miRNAs

To identify significantly expressed miRNAs between “Dangshan Su” × “Yali” and “Dangshan Su” × “Wonhwang”, miRNA quantification from fruit samples at 15 d, 23 d, 31 d, 47 d, 55 d, 63 d, and 145 d after flowering (DAF) was performed using Poly (T) RT-PCR. To produce miRNA fused with Poly (T) cDNA, 0.5 mg total RNA was used for the reverse transcription with miRNA mature sequence specific Poly (A) RT primers according to the Poly (T) RT-PCR protocol [30]. The 5s rRNA was used as an internal reference [21]. All the primers were listed in the Supplementary Material_1 (see Supplementary Material available online at https://doi.org/10.1155/2017/2794040). qRT-PCR analysis on cDNA samples at different developmental stages from two pollinated pear varieties was carried out with three repeats. Relative gene expression levels were calculated by the 2^−ΔΔCT^ method [31]. Reactions contained the following: 10 *μ*L of 2x* TransStar*® Tip Green qPCR SuperMix, 2 *μ*L of template cDNA, 0.4 *μ*L of forward and Universal miRNA qPCR Primer, 0.4 *μ*L of Passive Reference Dye (50x), and water to 20 *μ*L. PCR amplification was carried out as follows: 50°C for 2 min, 94°C for 34 s, followed by 40 cycles of 94°C for 5 s, and 60°C for 30 s.

### 2.8. RNA Extraction and qRT-PCR Analysis for Target Genes

To examine the expression of target genes in “Dangshan Su” × “Yali” and “Dangshan Su” × “Wonhwang,” fruit samples at 15 d, 23 d, 31 d, 47 d, 55 d, 63 d, and 145 d after flowering (DAF) were used. Total RNAs from samples were extracted using the Trizol reagent (Invitrogen) according to the manufacturer's instructions. The DNase-treated RNA was reverse-transcribed using M-MLV reverse transcriptase (Invitrogen).

Primers (Supplementary Material_1) for qRT-PCR were designed using Primer Express 3.0 software (Applied Biosystems), and the* Tubulin* gene (GenBank: AB239680.1) was used as an internal reference with primers synthesized by Sangon Biotech Co., Ltd. (Shanghai, China). qRT-PCR was carried out as above mentioned. Reaction system contained 10 *μ*L of SYBR Premix Ex Taq II (2x), 1 *μ*L of template cDNA, 0.5 *μ*L of forward and reverse primer, and water to 20 *μ*L. PCR amplification was carried out using the following program: 50°C for 2 min, 95°C for 30 s, followed by 40 cycles of 95°C for 15 s, 60°C for 20 s, and 72°C for 20 s.

### 2.9. Determination of Stone Cell Content

Stone cell content was measured as described by Cai et al. [32] Pulp (5 g) from between 2 mm beneath the peel to 0.5 mm from the core was collected and stored at −20°C for 24 h then homogenized at 20,000 rpm for 3 min. Homogenized pulp was incubated in water; the upper suspension was decanted, and this procedure was repeated several times. Stone cells were oven dried and weighed three times, and the content was calculated as follows: (stone cell content (%) = weight of stone cells (g DW)/weight of pulp (g FW) × 100).

### 2.10. Determination of Lignin Content

Lignin content was measured using the Klason method [33]. Pulp (5 g) from between 2 mm beneath the peel and 0.5 mm from the core was collected, oven dried, ground into a uniform powder, and passed through a 200-mesh sieve. The powder was extracted with methanol and oven dried. A small amount (0.2 g) of this powder was extracted with 15 mL of 72% H_2_SO_4_ at 30°C for 1 h, combined with 115 mL of distilled water, and boiled for 1 h. The volume was kept constant during boiling. The mixture was filtered and the residue was rinsed with 500 mL of hot water, air dried, and weighed.

## 3. Results and Discussion

### 3.1. RNA Library Construction, Sequencing, and Data Analysis

To characterize the difference of small RNA expression levels between “Dangshan Su” × “Yali” (DangsuA) and “Dangshan Su” × “Wonhwang” (DangsuB), small RNA libraries were constructed for DangsuA and DangsuB, respectively. Using deep sequencing, a total of 10,830,189 and 9,925,992 reads were generated from DangsuA and DangsuB, respectively. After removing adapter sequences and low-quality reads, 10,809,068 (99.81%) and 9,906,978 (99.81%) clean reads from each sample group were maintained for analysis (Table 1).

**Figure 1:**
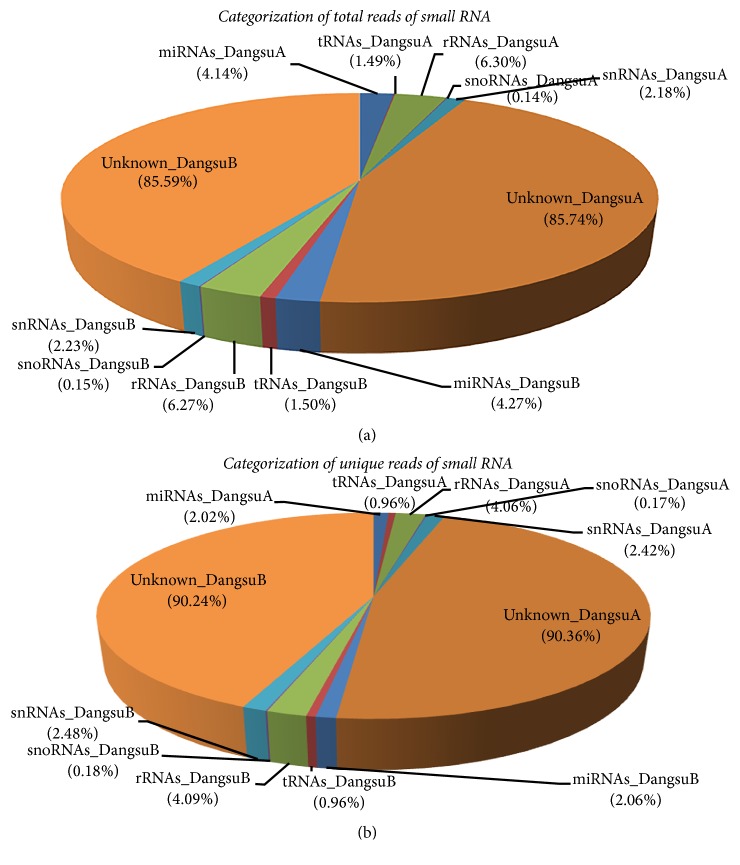
Categorization of total reads and unique reads of small RNA.

**Figure 2:**
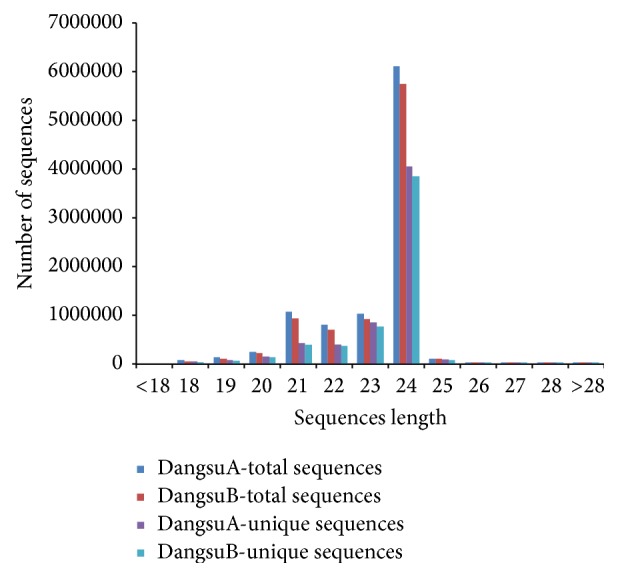
The length distribution of small RNA.

All these reads from both samples were annotated to small RNA sequences deposited in the Rfam11.0 database (http://rfam.janelia.org/). Using “blastn” program with command “blastn-short task,” we separated the small RNAs that matched other noncoding sequences, such as ribosomal RNA (rRNA), small nuclear RNA (snRNA), small nucleolar RNA (snoRNA), and transfer RNA (tRNA). The annotation of these small RNAs is listed in Figure 1. The remaining reads were considered as potential miRNAs products with various lengths of fragments (Figure 2). The 21–24 nt sequences accounted for 93.86% and 94.01% of the total clean reads in DangsuA and DangsuB, respectively (Figure 2). Both samples shared a similar distribution pattern, in which the 24 nt reads were the most abundant class (63.85% in DangsuA, 66.62% in DangsuB). This result is consistent with other species, such as tomato [34],* Citrus trifoliate* [35], and* Medicago truncatula* [36], which is the typical size range for dicer-derived products in plants [37].

**Figure 3:**
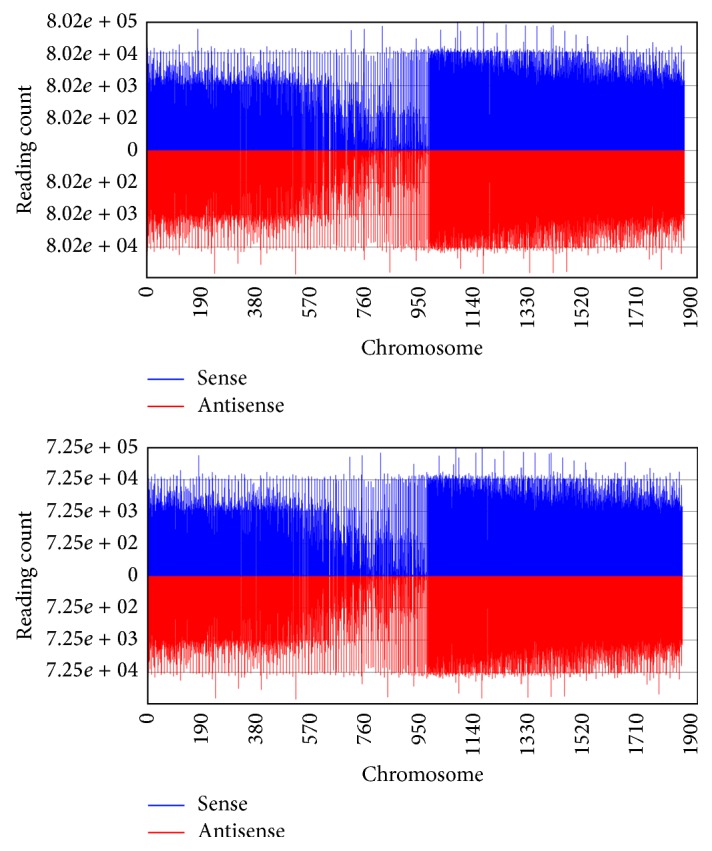
Distribution of sequence reads mapped to pear genome.

The remaining reads were then aligned to the pear genomes (http://peargenome.njau.edu.cn/), generating 53.72% and 54.05% genome-matched reads within DangsuA and DangsuB libraries, respectively (Table 2 and Figure 3). This mapping rate is lower than that in other research [21], due to the usage of alignment parameter “bowtie -n 1 -l 18 -a -m 30 –best –strata.” These small RNA reads were grouped into several RNA classes including rRNA, tRNA, snRNA, and snoRNA. The remaining unclassified small RNA reads contained the highest fraction of unique and total clean reads, which are likely composed of some known miRNAs and new types of regulatory novel miRNAs.

### 3.2. Identification of Both the Known and Novel MicroRNAs

#### 3.2.1. Identification of the Known MicroRNAs

Due to the lack of pear miRNA related information within the miRBase21 database (announced in June 2014), we had to compare our measured sRNA sequences with miRNA precursor/miRNA sequences from other published plants within the miRBase21 database, in order to display sequences and quantity of miRNA families from the samples tested, regardless of species.

After normalizing the miRNA expression levels of both DangsuA and DangsuB, 617 types of the known microRNAs were identified. Out of these microRNAs, 451 are present in both sample groups and other 71 species; 82 and 84 miRNAs are specifically expressed in DangsuA and DangsuB and distributed among 44 and 42 species, respectively (Supplementary Material_2).

**Figure 4:**
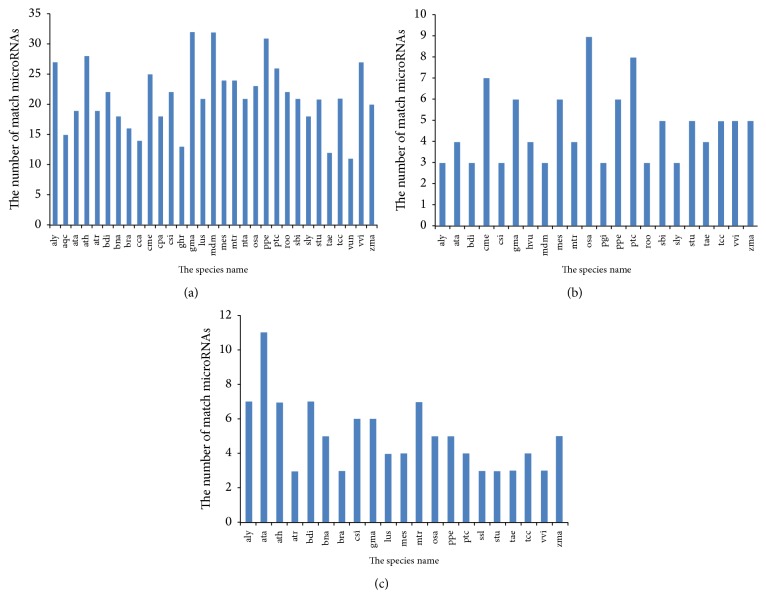
(a) Known miRNA matching number in different species. (b) Known miRNA matching number in DangsuA. (c) Known miRNA matching number in DangsuB.

The most common species, matching the microRNAs in DangsuA and DangsuB, are* Malus domestica* and* Glycine max*, each with 32 matches, followed by* Prunus persica* with 31,* Arabidopsis thaliana* with 28, and* Vitis vinifera* with 27 matches, respectively (Figure 4(a)). Species* Oryza sativa* with 9,* Populus trichocarpa* with 8, and* Cucumis melo* with 7 matches were the DangsuA specific ones (Figure 4(b)). The 11 miRNAs that were expressed in DangsuB were matched in* Aegilops triaristata*, while* Arabidopsis*,* Brachypodium distachyon*, and* Medicago truncatula* all had 7 miRNAs, respectively (Figure 4(c)). As shown in Figure 4, although apples and beans both have 32 microRNA matches with our samples, there are a total of 413 microRNAs announced for* Malus domestica* and 1215 for* Glycine max* on the whole in the miRbase database [25]. Moreover, microRNA matching rate for* Malus domestica* and* Glycine max* is 7.75% and 2.63%, respectively, indicating that the species relationship should be correlated with the microRNA matching rate.

#### 3.2.2. Identification of Novel miRNAs

**Figure 5:**
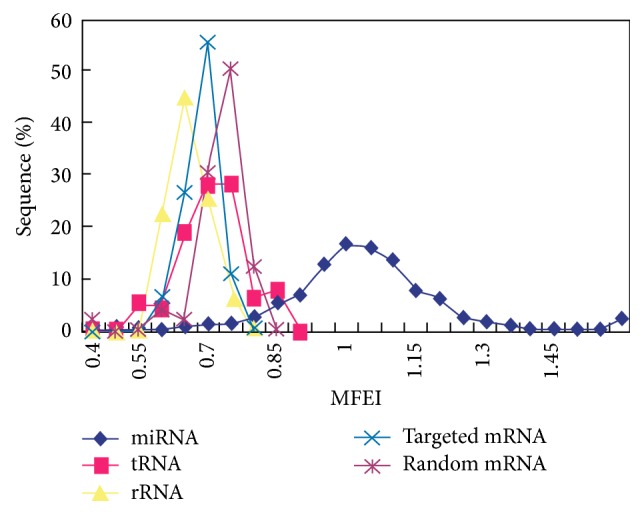
Comparison of the minimum folding free energy index (MFEI) of miRNA precursors with other RNAs.

**Figure 6:**
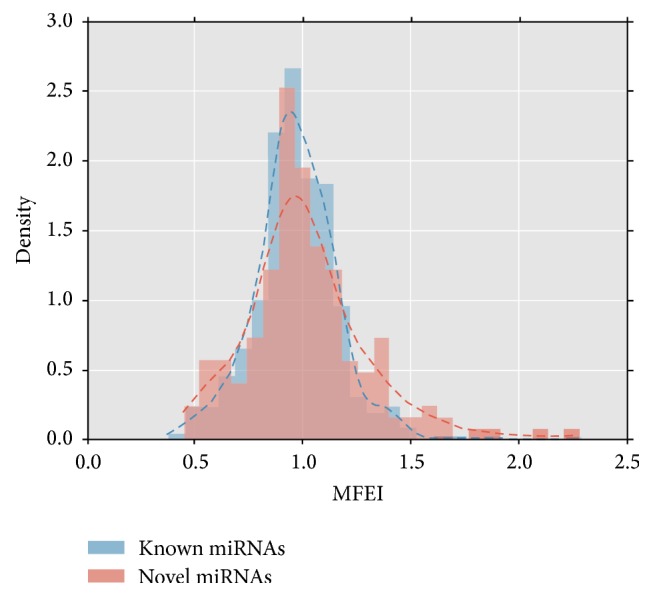
The MFEI of the candidate novel miRNA.

**Figure 7:**
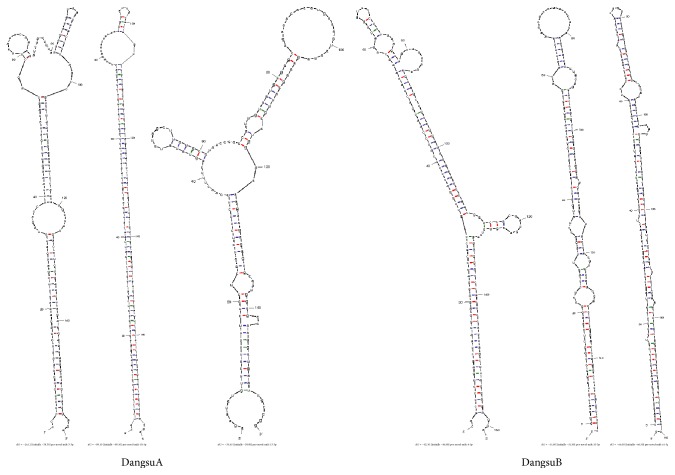
The stem-loop structure of the candidate novel miRNA.

Iconic hairpin structure of miRNA precursors can be used to predict new miRNA. Through the interception of a certain length of sRNA to compare with the pear genome, we can explore the secondary structure, Dicer enzyme cutting site, energy, and other characteristics information and predict new microRNA by software Mireap (http://sourceforge.net/projects/mireap/). Compared with other RNAs, MFEI of novel microRNAs larger than 0.85 are relatively more credible (Figure 5) [28]. A total of 168 and 106 new kinds of miRNAs were found in DangsuA and DangsuB, respectively (Supplementary Material_3). The mature sequence length of these novel miRNAs is within the range of 18–24 nt, with both 21 nt and 24 nt dominant. It can be predicted that the length of premiRNA sequences is within the range of 62~294 nt, and minimum hairpin folding free energy (MFE) has the range of 168.4 kcal/moL~−20.9 kcal/moL, minimum folding free energy index (MFEI) is in the range between 0.45 and 2.09, with the majority of 0.85. All above information indicated high reliability prediction of the novel microRNAs (Figure 6). The predictive novel miRNA precursor sequence can form a good stem-loop structure (Figure 7), which served as another evidence for high reliability prediction of the novel microRNAs.

**Figure 8:**
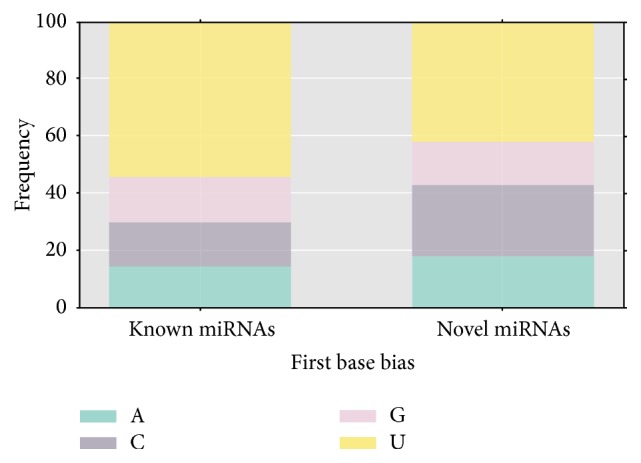
miRNA first nucleotide bias.

There exists a certain degree of base preference at different sites in microRNA sequences, which helps in not only microRNA cutting, but also target gene identification and other functions. For example, the U-base of the first mature sequence (5′) is characterized to help in microRNA identification by ARGONAUTEI. Conservative small molecule RNA was subjected to base preferences analysis to initially determine the reliability of the resulting sequence. The results showed that most of the first base of known and novel microRNAs were U, and the known microRNAs had substantially equal nucleotide contents for A, C, and G, while the novel ones had relatively more C nucleotide content but relatively less of the G base (Figure 8).

### 3.3. Cluster Analysis of MicroRNAs from the Differently Pollinated “Dangshan Su”

**Figure 9:**
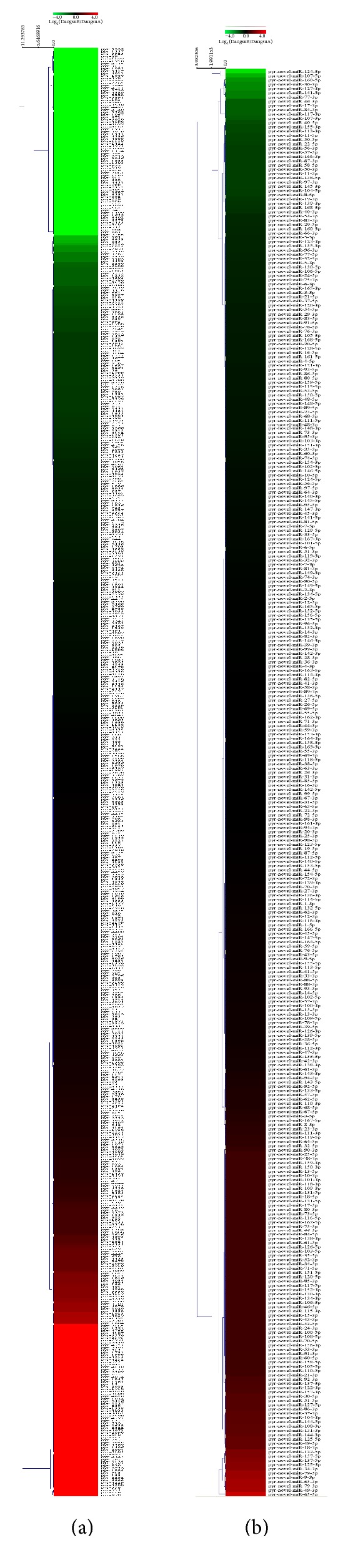
Cluster analysis of microRNA expression. (a) Expression pattern of known microRNA. (b) Expression pattern of novel microRNA.

Expression pattern analysis of these microRNAs indicated that the expression quantity of some microRNAs in DangsuB (Figure 9(b)) was higher than those in DangsuA (Figure 9(a)), while other microRNAs showed a reversed pattern. As showed in Figure 9(a), pyr-miR3229, pyr-miR1557, pyr-miR3925, and other known microRNAs had less expression quantity in DangsuB than in DangsuA; pyr-miR595, pyr-miR3496, and others in DangsuA and DangsuB had the same expression quantity basically; and pyr-miR870, pyr-miR518, pyr-miR753, and so forth apparently had higher expression quantity in DangsuB than in DangsuA. Data from Figure 9(b) showed that pyr-novel-miR-124-3p, pyr-novel-miR-107-5p, pyr-novel-miR-160-5p, and other novel microRNAs were less expressed in DangsuB than in DangsuA; pyr-novel-miR-61-5p, pyr-novel-miR-49-3p, and so forth were consistently expressed in both; and pyr-novel-miR-65-5p, pyr-novel-miR-49-3p, pyr-novel-miR-79-3p, and others expression quantity was significantly higher in DangsuB than in DangsuA; and the pyr-novel-miR-150-3p, pyr-novel-miR-109-3p, and so forth were only expressed in DangsuA; and the pyr-novel-miR-159-3p, pyr-novel-miR-80-3p, and so forth were only expressed in DangsuB.

### 3.4. The Variance Analysis of MicroRNA in “Dangshan Su” Different Pollination

**Figure 10:**
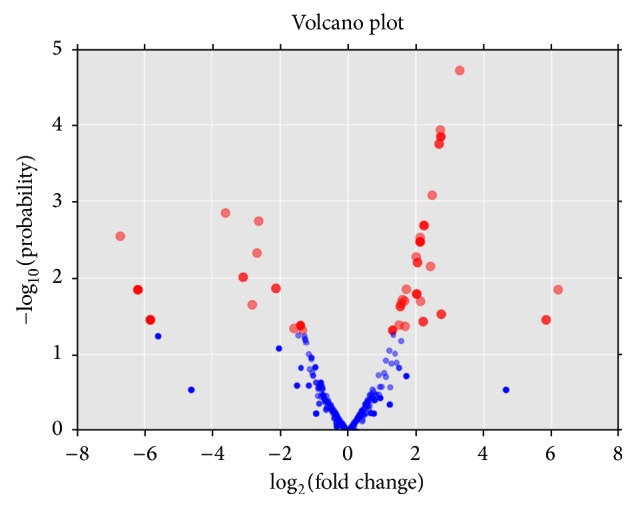
The volcano plot for differentially expressed miRNA. Note: abscissa represents the microRNA expression multiple changes in the different samples, while ordinate represents the significant level of microRNA expression changes, and blue dots indicate no significant differences in the microRNA and red dot indicates there are significant differences in microRNA.

To check out if there are significant differences in expression levels between two samples, we performed the microRNA expressed statistics in DangsuA and DangsuB using microRNA edgeR (R package). With volcano plot analysis (Figure 10), the overall distribution of differential microRNAs can be inferred, based on fold changes and the significant level (*p* value < 0.05).

**Figure 11:**
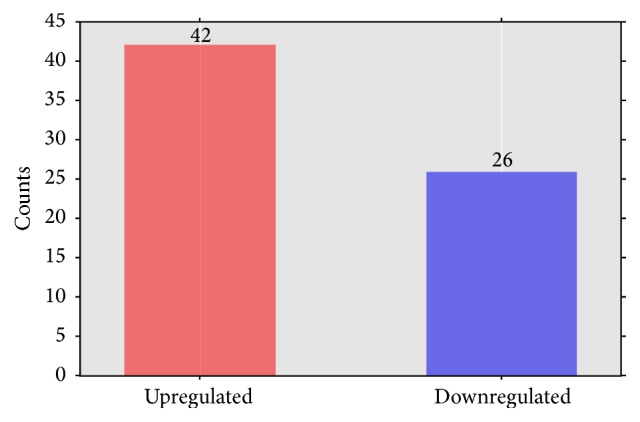
The number of different expressed microRNAs.

As showed in Figure 11, a total of 68 kinds of microRNAs with significant expression differences were identified in both samples (DangsuA and DangsuB). And 42 microRNAs had the higher expression quantity in DangsuB than in DangsuA, where 23 kinds are known microRNAs and 19 kinds are novel microRNAs. 26 microRNAs had less expressions in DangsuB than in DangsuA, where 16 kinds are known microRNAs and 10 kinds are novel microRNAs. In DangsuB, pyr-miR870 and pyr-novel-miR-65-5p showed 2^6.2^ and 2^3.3^ times higher than those in DangsuA. There are some microRNAs, such as pyr-miR3329 and pyr-novel-miR-10-3p, with the significantly reduced expression (Supplementary Material_4). These results suggested that difference in pollination should be able to significantly alter some microRNAs expression in “Dangshan Su.”

### 3.5. Target Gene Prediction for Differentially Expressed MicroRNA in “Dangshan Su” Different Pollination

According to the known interactions between miRNAs and target genes, it was found that 2–8 nucleotides in the miRNA 5′ end were completely complementary with UTR region in target mRNA 3′ end [38, 39]. This feature is verified by a variety of target gene prediction methods. However, there are other restrictions in miRNA target gene prediction: the specific matching circumstances of miRNA 3′ end and the target gene, miRNA secondary structure, thermodynamic stability of the dimer of miRNA and miRNA target genes, and so on. In order to reduce target gene prediction false positive results, we used the software in plant microRNA target gene forecast, TAPIR and TargetFinder, which both have good predictions for the target genes [29]. The combined use of these two software programs leads to a number of predicted miRNA target genes and their corresponding target genes.

**Figure 12:**
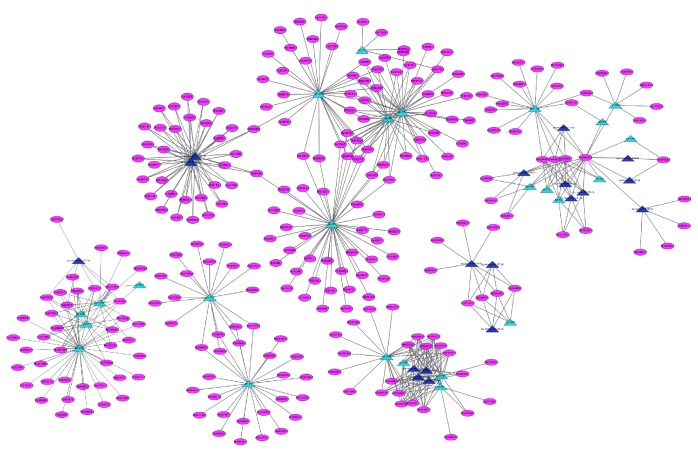
Relationship of different expression miRNAs and their target genes.

Among 68 differentially expressed microRNAs, 641 target genes were obtained and classified into different gene families which were involved in transcriptional regulation of stress response, signal transduction, growth, and transmembrane transport in plants (Supplementary Material_5). Regulatory network from the differentially expressed microRNA target genes and their corresponding mapping (Figure 12), was divided into six parts (Supplementary Material_6). As showed in the network, a microRNA gene can regulate multiple targets at the same time, and a target gene can also be regulated by more microRNAs. For example, pyr-miR401 and pyr-novel-miR-127-3p regulate 40 and 33 target genes, respectively, indicating that they may play an important regulatory role in pear fruit xenia phenomena. And the target gene Pbr016259.1 was regulated by 16 differentially expressed microRNAs. As Pbr016259.1 encodes for an acidic amino acid ligase, it can be inferred that acidic amino acid ligase plays an important regulatory role in pear fruit growth start, and its expression could be simultaneously controlled by multiple microRNAs in synergy regulation.

### 3.6. Function Annotation of Differentially Expressed MicroRNA Target Gene in “Dangshan Su” Different Pollination

#### 3.6.1. GO Function Annotation of Differentially Expressed MicroRNA Target Gene

Gene Ontology (GO) is an international standardized classification system for gene function, which supplies a set of controlled vocabularies to comprehensively describe the property of genes and gene products. There are three ontologies in GO: molecular function, cellular component, and biological process. The basic unit of GO is GO term, each of which belongs to one type of ontology.

**Figure 13:**
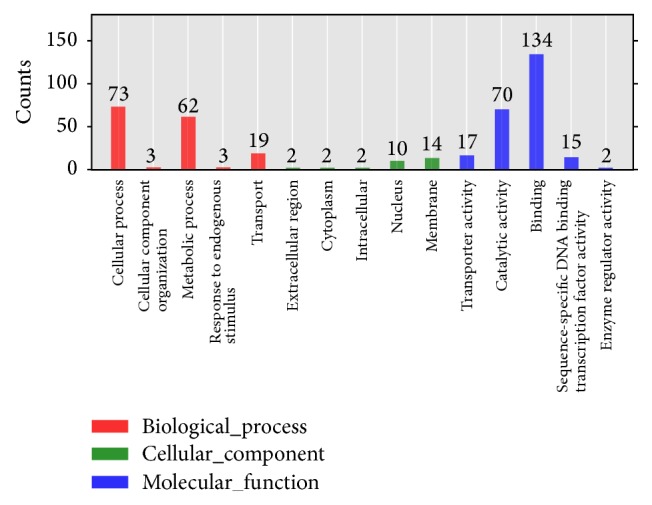
GO annotation class of different expressed genes.

GO enrichment analysis can predict target gene candidates of novel miRNAs, as well as their biological function. The result could reveal the functions significantly related to predicted target gene candidates of novel miRNAs. As shown in Figure 13, among the differentially expressed microRNAs target genes, there are two significantly enriched functions, cellular physiological processes and metabolic processes, accounting for 45.63% and 38.75%, respectively, in the entire biological process. Nucleus and film composition of cellular component were also significantly enriched, accounting for 33.33% and 46.67% of cellular component, respectively. Catalytic activity and binding combination of molecular function were significantly enriched, accounting for 29.41% and 56.30% of molecular function, severally. Moreover, most of the GO terms (*q* value ≤ 0.05) were related to molecular function (80%), a small amount with biological process (20%), and no cellular component was detected (Supplementary Material_7). That means in the different pollination of “Dangshan Su,” the effect of male parent to “Dangshan Su” stone cell formation and quality was altered by the microRNA expression quantity of target genes which has the molecular regulation functions and biological processes and is not related to “Dangshan Su” cellular components.

#### 3.6.2. Pathway Analysis of Differentially Expressed MicroRNA Target Genes

KEGG pathway analysis is also used for the target gene candidates. In organisms, genes usually interact with each other to play different roles in certain biological function. The analysis based on pathways could facilitate the understanding of biological functions of genes. KEGG is the major public pathway-related database [40]. KEGG pathway analysis identifies significantly enriched metabolic pathways or signal transduction pathways in target gene candidates comparing with the whole reference gene background.

KEGG pathway analysis of the differentially expressed microRNA target genes (Supplementary Material_8) suggested that 68 target genes were enriched into 52 KEGG pathways. When the parameter *p* value ≤ 0.05, the significant enrichment of KEGG pathways mainly has plant pathogen interactions (Ko04626), organic selenium compound metabolized (Ko00450), sulfur metabolism (Ko00920), single stick biosynthesis (Ko00902), other polysaccharides degradation (Ko00511), purine metabolism (Ko00230), and so on.

### 3.7. Impact of the Differential Expressed MicroRNAs on the Lignin Metabolism in Different Pollinated “Dangshan Su”

#### 3.7.1. MicroRNA Target Gene Prediction for Lignin Metabolism Regulation

Stone cells are formed by secondary thickening and lignin deposition of parenchyma cell wall [32, 41]. Therefore, lignin biosynthesis, transport, and deposition are closely related to stone cell development. One feature of pear fruits is of the enrichment of stone cells during their development, which directly affects pear fruit quality. The metabolic regulation of lignin was significantly affected by microRNA. Wu et al. detected the presence of significant expression differences of miR3711, miR419, and miR5260 in different developmental stages of “Dangshan Su” and found that their common target gene was* HCT* which is encoding a key enzyme of lignin metabolism [21]. Rubinelli et al. confirmed that overexpression of miRNA of miR156 family in transgenic* Populus tomentosa* can have effective influence on the tree structure and the lignin content [42]. Lu et al. proved that the ptr-miR397a overexpression significantly and negatively regulated laccase in lignin metabolic pathway and also significantly affected lignin content in* Populus tomentosa* [17].

Through the differentially expressed microRNA GO enrichment and KEGG pathway analysis, we discovered that the expression level of pyr-novel-miR-144-3p in DangsuB was significantly higher than that in DangsuA. Through target genes prediction of this microRNA, 6 target genes (Pbr028952.1, Pbr002903.1, Pbr018935.1, Pbr001412.1, Pbr016259.1, and Pbr003857.1) were obtained. Pbr028952.1 encodes for an ATP enzyme. Both Pbr002903.1 and Pbr001412.1 play the function of transport substrates via transmembrane. Pbr016259.1 encodes for an acidic amino acid ligase. Pbr018935.1, as well as Pbr003857.1, encodes for a laccase (PbrLAC35 or PbrLAC57, resp.) which is involved in the last key step of lignin metabolic pathways. Therefore, we proposed that via different pollination on “Dangshan Su,” the different males could affect pyr-novel-miR-144-3p expression, which subsequently regulated the laccase expression within lignin monomer polymerization process and thus controlled lignin and stone cells synthesis in pear fruit; this result is consistent with Wu and Niu [21, 23].

#### 3.7.2. Evolutionary Analysis of Target Genes of Lignin Metabolic Regulation

To further study the homologous evolutionary relationships and functions between* PbrLAC35* and* PbrLAC57* in “Dangshan Su,” we used NCBI BLAST to retrieve the genes which has 75% more similarity with* PbrLAC35* and* PbrLAC57* amino acid sequence in other species. The online analysis tools like ExPAsy (http://www.expasy.org/) and WoLF PSORT (http://www.genscript.com/wolf-psort.html) were used to screen essential information of amino acids, such as length, molecular weight, isoelectric point, and subcellular localization of proteins (Tables 3 and 4). Proteins similar to PbrLAC35 and PbrLAC57 sequence in various species have the molecular weight between 63 kD and 68 kD and isoelectric point greater than 8.71, belonging to basic protein. And most of them are localized in chloroplasts and vacuole.

**Figure 14:**
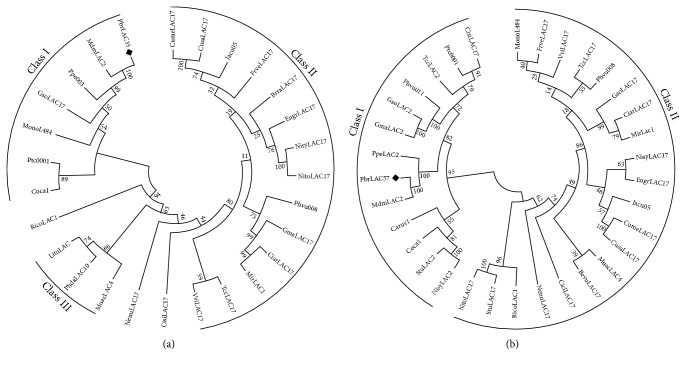
Phylogenetic tree of PbrLAC35 and PbrLAC57 in* P. bretschneideri* Rehd. constructed with neighbor-joining methods. (a) Phylogenetic tree of PbrLAC35. (b) Phylogenetic tree of PbrLAC35.

In order to further analyze homologous evolutionary relationships of PbrLAC35 and PbrLAC57 in different species, we used ClustalW tool in MEGA6.0 software to run the gene multiple sequence alignment for the amino acid sequences in different species then used neighbor-joining method (N-J) (bootstrap = 1000) to build composite evolutionary trees of different species. As showed in Figure 14(a), similar sequences from 27 species can be divided into three categories (I, II, and III). The predicted target gene PbrLAC35 was targeted in class I and united with the apple MdmLAC2, and then the yellow peach Ppe003 was grouped into a branch with bootstrap support rate up to 99%. And this branch subsequently combined with the GsoLAC17 of the soybean, the MonoL484 of the morus, the Ptc0001 of western balsam poplar, and the Coca1 of coffee to form class I. As shown in another phylogenic tree (Figure 14(b)), which was constructed with similar sequences from 28 species and composed of two classes (I and II), the predicted target gene* PbrLAC57* was located in class I. Similar to the clustering of PbrLAC35, PbrLAC57 was firstly combined with MdmLAC2; then peach PpeLAC2 was clustered into a branch supported by bootstrap up to 100%. But it can also be seen that the bootstrap support rate of the later clustering is relatively low, only 50% and 82%, respectively, which also shows that the conservatism of the target gene prediction in the close genetic relationship species among the species is very high, but the conservatism in different species is very low. This may be related to the two laccases function, and the major role in the Rosaceae is involved in the lignin metabolism, while in some wood and Gramineae plants, it may have participation into the lignin metabolism pathway of tree type building.

#### 3.7.3. Differentially Expressed MicroRNAs and Their Target Genes qRT-PCR Validation

**Figure 15:**
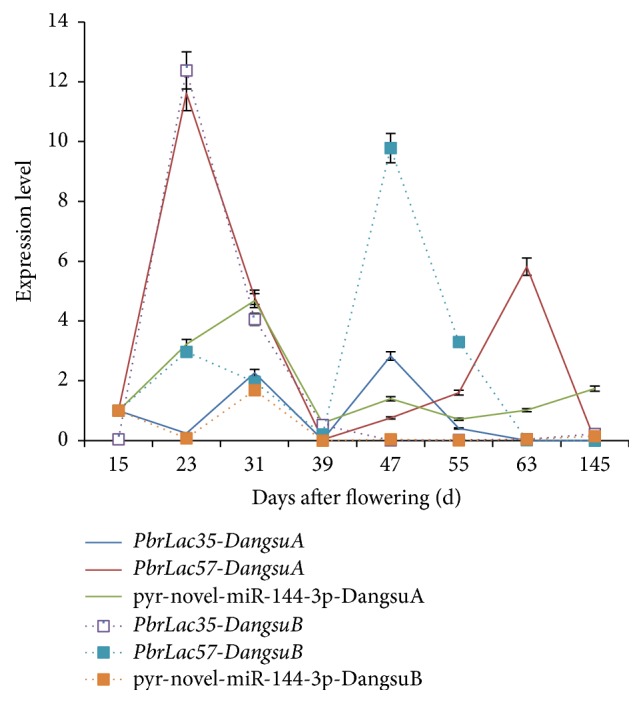
Expression pattern of miRNAs and target genes.

In order to verify the above results, we used qRT-PCR to determine the expression patterns of both pyr-novel-miR-144-3p and its target genes* PbrLAC35* and* PbrLAC57* at the different developmental stages of “Dangshan Su” with different pollination. It can be found that the expression of pyr-novel-miR-144-3p in DangsuA at different times after the flowering was significantly higher than that in DangsuB (Figure 15). In DangsuA, pyr-novel-miR-144-3p showed an expression trend of initial increase, subsequent decrease, and the maximum at 31 d. Although its expression also reached a maximum at 31 d in DangsuB, the trend was dropping, increasing, and dropping till a stable level of expression. These results suggested that different pollination types had a significant difference in pyr-novel-miR-144-3p expression pattern. However, its expression peak at 31 d was similar within both pollinated “Dangshan Su,” thereafter entering a stable expression period in the late stage.

In DangsuA, the target gene* PbrLAC35* expression trend is down-up-down-up-down and reaches a maximum at 47 d;* PbrLAC57* expression trend is firstly up-down-up-down and reaches maximum at 23 d. We can see that when the pyr-novel-miR-144-3p reached a maximum expression at 31 d,* PbrLAC57* expression decreased sharply from 58.08 to 4.79 and* PbrLAC35* expression and the pyr-novel -miR-144-3p expression are in trade-off and taking-turns status.

In DangsuB, the expression trends of both* PbrLAC35* and* PbrLAC57* showed a status of fluctuation until reaching a peak at 31 d and 47 d, respectively. It is noticeable that pyr-novel-miR-144-3p expression experienced a level from high to low during 15 to 23 days after flowering, while the expression levels of both* PbrLAC35* and* PbrLAC57* were maintained in a rising patterns. Furthermore, when pyr-novel-miR-144-3p expression began to rise from 23 d to 31 d, the expression of its two target genes declined significantly. Therefore, we concluded that, in the different pollination “Dangshan Su,” pyr-novel-miR-144-3p significantly regulates expression levels of laccases (*PbrLAC35* and* PbrLAC57*) in lignin metabolic pathways. The results were consistent with previous conclusion from the target gene prediction.

**Figure 16:**
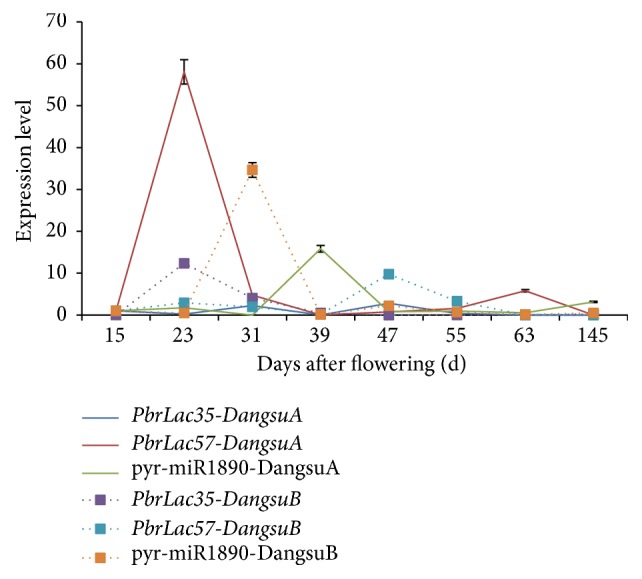
Expression pattern of miRNAs and target genes.

According to previous reports, ptr-miR397a can significantly lower the expression of laccase in lignin synthesis regulation in* Populus tomentosa* [17, 21]. qRT-PCR analysis was used to verify the expression of microRNA pyr-miR1890 whose sequence is the same with ptr-miR397a.  Figure 16 showed that the expression trends of pyr-miR1890 in DangsuA and DangsuB were both decreased after the first rise and reached the maximum at 39 d in DangsuA and at 31 d in DangsuB, respectively. With the increase of pyr-miR1890 expression, the expression of the two target laccases showed a significant decrease, indicating that pyr-miR1890 also played a role in the regulation of laccase (*PbrLAC57* and* PbrLAC35*), which was consistent with the previous reports that a microRNA could regulate multiple target genes and the same target gene is regulated by multiple microRNA [43].

#### 3.7.4. Analysis of Lignin and Stone Cell Content in “Dangshan Su” Different Pollination

**Figure 17:**
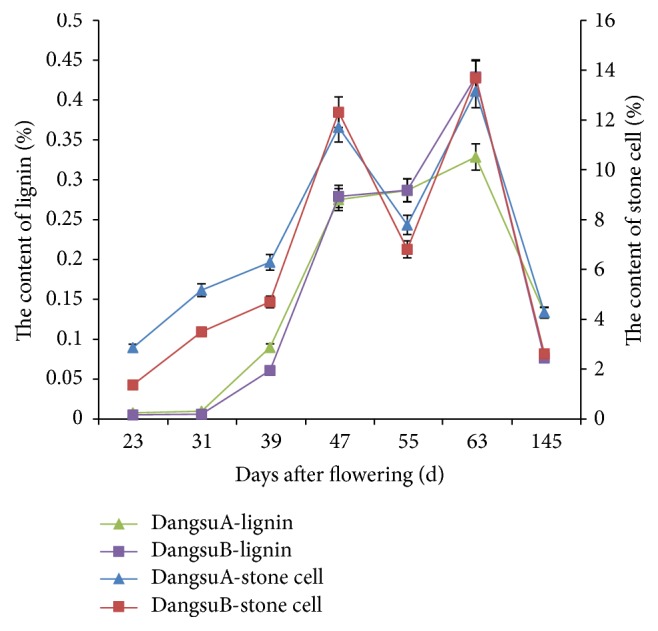
Content of stone cells and lignin in different pollination varieties of “Dangshan Su.”

Figure 17 showed that the trends of lignin content were firstly increasing until up to the maximum at 63 days after flowing then decreased. At 23, 31, 39, and 145 days after flowering, DangsuA lignin content was higher than DangsuB, while in other times DangsuA lignin content was less than DangsuB. The expression of* PbrLAC57* was maximal at 23 d in DangsuA and at 47 d in DangsuB. Therefore, we inferred that due to the impact of xenia phenomenon,* PbrLAC57* expression trend was variable in both DangsuA and DangsuB and is expressed in the early and late fruit development stage of DangsuA, while it is expressed in the mid stage of DangsuB. Moreover the DangsuA lignin content was higher than DangsuB at the development of early and late stage, while DangsuA lignin content was less than in DangsuB at the mid-stage. Thus, laccase PbrLAC57 may be a key regulatory enzyme for catalyzing lignin monomer coupling polymerization into lignin polymer.

“Dangshan Su” pear by different pollination had the same changing trend of stone cell content, increasing at first, reaching a small peak at 47 d, and then down-up again to reach maximum at 63 d, then decreasing. At 47 d and 63 d, DangsuA stone cells content was less than DangsuB, while in other times, they were higher than DangsuB. Thus it can be seen that stone cell contents were significantly related to lignin contents with a correlation coefficient of 0.871 and 0.981 in DangsuA and DangsuB, respectively.

#### 3.7.5. Pathway Analysis of MicroRNA Regulation in Pear Fruit Quality

**Figure 18:**
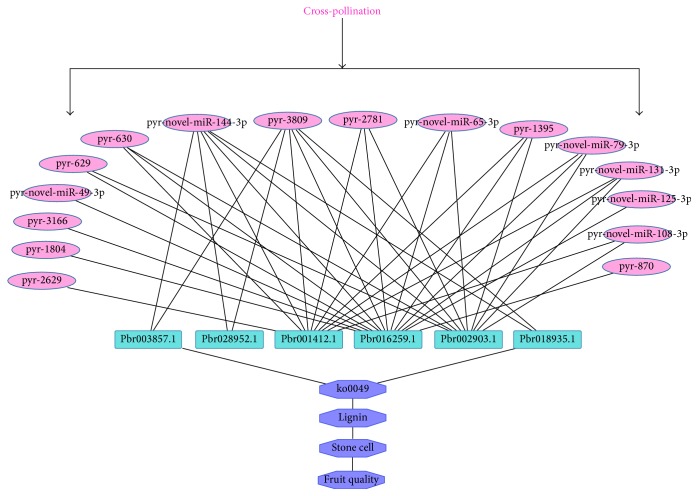
Pathway analysis of microRNA regulation in pear fruit quality.

According to the previous results in this study, we hypothesized that different pollination types could regulate “Dangshan Su” fruit quality through the following steps (Figure 18): (1) different males affect the expression quantity of pyr-novel-miR-144-3p and pyr-miR1890 by xenia phenomenon; (2) the expression changes of pyr-novel-miR-144-3p and pyr-miR1890 result in the corresponding regulation of target gene (Pbr003857.1, Pbr028952.1, Pbr001412.1, Pbr016259.1, Pbr002903.1, and Pbr018935.1) expression level changes, especially laccases* PbrLAC35* and* PbrLAC57* expressions have significant difference in different developmental stages of different pollination; (3)* PbrLAC35* and* PbrLAC57*, through expression changing, cause catalyzing of lignin monomer coupling polymerization reaction change and lignin content change; and (4) the change of lignin content results in the content change of stone cells formed by lignin deposition and achieves the effect of pear fruit quality by changing the stone cell contents.

At the same time, xenia cannot regulate lignin biosynthesis through direct effects on lignin key enzyme genes in the phenylpropanoid metabolism of the metabolic pathway upstream. Instead, it can be indirect regulation through microRNAs, which are regulators of the related laccases, to affect the synthesis of lignin and the formation of stone cells in order to control the quality of pear. This is also consistent with the principle of the evolution of species. In the critical period of lignin synthesis, the regulation can precisely catalyze the laccases for lignin monomer polymerization, which not only can accurately promote or inhibit the synthesis of lignin, but also can avoid the regulation of the upstream related gene expression and the impact of the entire secondary metabolic pathways.

## 4. Conclusions

We found that xenia phenomenon, instead of direct regulation, indirectly controlled lignin biosynthesis via the microRNAs which target the key genes in the phenylpropanoid metabolism, such as laccase genes. Moreover, it is found the formation of stone cells was significantly related to the lignin synthesis, which could further affect pear quality.

## Supplementary Material

These Supplementary Materials contained the detailed data of the primer sequences used in this study, those known and novel microRNAs, differentially expressed microRNAs, the target genes of the differentially expressed microRNAs and which corresponding GO function enrichment analysis and KEGG pathway analysis in “Dangshan su” Pear of different male parent pollination.

## Figures and Tables

**Table 1:** Distribution of counts of sequ-seqs type in 2 libraries.

Type	DangsuA		DangsuB	
	Count	Percent (%)	Count	Percent (%)
Total reads	10830189		9925992	
High reads	10809068	99.81%	9906978	99.81%
3′adapter null	25767	0.24%	21055	0.21%
Insert null	38463	0.36%	30237	0.31%
5′adapter contaminants	7239	0.07%	5888	0.06%
Smaller than 18 nt	43248	0.40%	31796	0.32%
PolyA	766	0.01%	1082	0.01%
Clean reads	10693585	98.93%	9816920	99.09%

**Table 2:** Distribution of sequence reads mapped to genome.

Sample name	Type	Numbers	Mapped	Unmapped
DangsuA	Unique reads	6074111	3514387 (57.858%)	2559724 (42.142%)
DangsuB	Unique reads	5649359	3275734 (57.984%)	2373625 (42.016%)
DangsuA	Total reads	9557093	5134308 (53.722%)	4422785 (46.278%)
DangsuB	Total reads	8773522	4742027 (54.049%)	4031495 (45.951%)

**Table 3:** Protein properties in different species which are similar to *PbrLAC35* protein sequences.

Gene name	Accession number	Length (aa)	Molecular weight (Da)	pI	Subcellular localization prediction
*MonoL484*	EXC10697.1	577	63851.3	9.61	Chlo
*PhdaLAC10*	XP_008778578.1	577	63139.5	9.23	Chlo
*CiarLAC17*	XP_004492322.1	580	64299.7	8.91	Chlo
*CisiLAC17*	XP_006480873.1	578	63944.6	9.44	Chlo
*CumeLAC17*	XP_008463808.1	578	64278.1	9.46	Chlo
*LituLAC*	AAB17194.1	585	64720.1	9.54	Chlo
*EugrLAC17*	XP_010031544.1	585	64702.3	8.92	Vacu
*Coca1*	CDP09087.1	587	64180.4	8.94	Chlo
*FrveLAC17*	XP_004294637.1	584	64356.1	9.33	Chlo
*GsoLAC17*	KHN32142.1	590	65611	9.02	Chlo
*GmaLAC17*	XP_006602563.1	578	63733.2	9.24	Chlo
*Jacu05*	KDP44151.1	576	63764.9	9.3	E.R.
*MdmLAC2*	XP_008356891.1	584	64570.4	9.68	Chlo
*MirLAC1*	KEH22816.1	580	64124.6	9.35	Chlo
*NenuLAC17*	XP_010256874.1	583	64130.5	8.88	Chlo
*NisyLAC17*	XP_009774063.1	581	64525.3	9.05	Chlo
*NitoLAC17*	XP_009587566.1	581	64628.3	8.97	Chlo
*Phvu008*	ESW12292.1	625	68966	9.13	Chlo
*Ptc0001*	EEE83028.2	576	63551.8	9.27	Chlo
*Ppe003*	EMJ09215.1	587	64588.1	9.77	Chlo
*TccLAC17*	EOY13993.1	577	63443.8	9.16	Chlo
*VviLAC17*	XP_002284473.1	585	64295.7	8.71	E.R.
*RicoLAC1*	XP_002531565.1	577	63727.7	9.47	Chlo
*BrraLAC17*	XP_009120521.1	573	63648.2	9.33	E.R.
*MuacLAC4*	XP_009403441.1	579	63250.4	8.97	Vacu
*CusaLAC17*	XP_004148786.1	579	64451.3	9.42	Chlo
*PpeLAC17*	XP_008222577.1	581	64247	9.19	Chlo
*PbrLAC35*	Pbr018935.1	585	64538.4	9.79	Chlo

**Table 4:** Protein properties in different species which are similar to *PbrLAC57* protein sequences.

Gene name	Accession number	Length (aa)	Molecular weight (Da)	pI	Subcellular localization prediction
*GmaLAC2*	XP_006592190.1	588	65380.7	9.10	Chlo
*MdmLAC2 *	XP_008384938.1	585	64520.8	9.63	Chlo
*NenuLAC17*	XP_010264408.1	591	65467.7	9.17	Chlo
*GsoLAC17 *	KHN16226.1	578	63795.2	9.19	E.R.
*CiclLAC17*	XP_006480873.1	578	63944.6	9.44	Chlo
*NisyLAC2*	XP_009796024.1	583	64518.8	9.14	Chlo
*RicoLAC1*	EEF30831.1	577	63727.9	9.47	Chlo
*CiarLAC17*	XP_004492318.1	580	64073.6	9.35	Chlo
*Ptc0001*	EEE83028.2	576	63551.8	9.27	Chlo
*CumLAC17*	XP_008463808.1	578	64278.1	9.46	Chlo
*MuacLAC4*	XP_009403441.1	579	63250.4	8.97	Vacu
*NisyLAC17*	XP_009796361.1	581	64514.1	9.05	Chlo
*Phvu011*	ESW04043.1	607	67189.9	9.41	Chlo
*Jacu05*	KDP44151.1	576	63764.9	9.30	E.R.
*EugrLAC17*	XP_010031544.1	585	64702.3	8.92	Vacu
*VviLAC17*	XP_003632204.1	585	64388.2	9.02	Chlo
*CisiLAC17*	XP_006488364.1	583	64515.8	9.25	Chlo
*GsoLAC2*	XP_006592189.1	590	65611.0	9.02	Chlo
*Coca1*	CDP09087.1	587	64180.4	8.94	Chlo
*CusaLAC17*	XP_004148786.1	579	64451.3	9.42	Chlo
*MonoL484*	XP_010092993.1	586	64520.2	9.28	Chlo
*MirLAC1*	XP_003623041.1	581	64658.1	9.16	Vacu
*TccLAC17*	XP_007016584.1	577	63460.1	9.20	Chlo
*BevuLAC17*	XP_010690924.1	582	64862.8	9.33	Chlo
*StuLAC17*	XP_004246425.1	571	63525.7	9.32	Chlo
*NitoLAC17*	XP_009630096.1	601	66685.2	9.20	Cyto
*Phvu008*	XP_007140298.1	625	68966.0	9.13	Chlo
*Caruv1*	XP_006293883.1	593	66097.0	9.47	Cyto
*PpeLAC2*	XP_008222248.1	587	64484.0	9.71	Chlo
*FrveLAC17*	XP_004294637.1	584	64356.1	9.33	Chlo
*TccLAC2*	XP_007016364.1	585	64146.6	9.39	Chlo
*StuLAC2*	XP_006350913.1	584	64771.4	9.22	Chlo
*PbrLAC57*	Pbr003857.1	584	64544.1	9.68	Chlo

## Additional Points

*Availability and Requirements.* GEO Submission (GSE80747). GSM2136459 DangsuA miRNA-seq. GSM2136460 DangsuB miRNA-seq. Database homepage: http://www.ncbi.nlm.nih.gov/geo/query/acc.cgi?acc=GSE80747.
