# Supplementary material for: The Effect of Different Pollination on the Expression of Dangshan Su Pear MicroRNA
Source: Biomed Res Int. 2017 Apr 10;2017:2794040. doi: 10.1155/2017/2794040 (PMC5402243; doi:10.1155/2017/2794040)
Supplement: Supplementary file 1 — These Supplementary Materials contained the detailed data of the primer sequences used in this study, those known and novel microRNAs, differentially expressed microRNAs, the target genes of the differentially expressed microRNAs and which corresponding GO function enrichment analysis and KEGG pathway analysis in “Dangshan su” Pear of different male parent pollination. [file 2794040.f1.zip › Supplementary Material/Supplementary Material 1. Primers for qRT-PCR.docx]

**Supplementary Material_1. Primers for qRT-PCR**

| **Homolog** | **Forward primer sequence (5’ to 3’)** | **Reverse primer sequence (5’ to 3’)** |
| --- | --- | --- |
| ***LAC35*** | **AGCCAATGTGCCCAAAACTG** | **GCCATGGGTTGGTTCCAA** |
| ***LAC57*** | **TGGCTTTTCTTCTTGCTCTTATCTC** | **ACTGCCAGCTGCAGCAATG** |
| **pyr-miR1890** | **TCATTGAGTGCAGCGTTGATG** | **Poly (T)** |
| **pyr-novel-miR-144-3p** | **TGCCAAAGGAGAGTTGCCCTT** | **Poly (T)** |
| ***Tubulin*** | **AGAACAAGAACTCGTCCTAC** | **GAACTGCTCGCTCACTCTCC** |
| **5s rRNA** | **GAAAGATGCCAATTCATGCG** | **Universal miRNA qRT-PCR Primer** |
