# Supplementary material for: The Effect of Different Pollination on the Expression of Dangshan Su Pear MicroRNA
Source: Biomed Res Int. 2017 Apr 10;2017:2794040. doi: 10.1155/2017/2794040 (PMC5402243; doi:10.1155/2017/2794040)
Supplement: Supplementary file 1 — These Supplementary Materials contained the detailed data of the primer sequences used in this study, those known and novel microRNAs, differentially expressed microRNAs, the target genes of the differentially expressed microRNAs and which corresponding GO function enrichment analysis and KEGG pathway analysis in “Dangshan su” Pear of different male parent pollination. [file 2794040.f1.zip › Supplementary Material/Supplementary Material7/GO.enrich.html]

Gene Ontology Enrichment Analysis


# Gene Ontology Enrichment Analysis

---

  

Note: Total detectd: 表示检测到的所有在该GO中基因数目

\* 各GO条目内的基因or蛋白的层次聚类仅针对富集的GO条目，层次聚类采用欧氏距离，聚类标准采用wald法。

GO topological diagram, marked GO iterm is significant enriched, plots are:biological\_process,cellular\_component,molecular\_function  
 Note: In topo plot, color indicate the enrichment qvalue, color 'yellow' is 0.05~1e-3, 'orange' is 1e-3~1e-8, 'red' is 1e-8~0.0

| # | GOid | Description | Class | Ratio | pvalue | qvalue | diffNum | diff\_cluster | Totaldetectd |
| 1 | GO:0043531 | ADP binding | molecular\_function | 18.2156532885 | 4.52201437045e-48 | 1.25666779355e-44 | 59 | cluster\_plot | 342 |
| 2 | GO:0032559 | adenyl ribonucleotide binding | molecular\_function | 2.76489350357 | 2.20985929863e-12 | 3.07059949545e-09 | 74 | cluster\_plot | 2826 |
| 3 | GO:0030554 | adenyl nucleotide binding | molecular\_function | 2.71871574151 | 4.54305437372e-12 | 4.20838270152e-09 | 74 | cluster\_plot | 2874 |
| 4 | GO:0032555 | purine ribonucleotide binding | molecular\_function | 2.4965882983 | 1.21473740202e-10 | 4.38513891938e-08 | 75 | cluster\_plot | 3172 |
| 5 | GO:0032550 | purine ribonucleoside binding | molecular\_function | 2.4965882983 | 1.21473740202e-10 | 4.38513891938e-08 | 75 | cluster\_plot | 3172 |
| 6 | GO:0001883 | purine nucleoside binding | molecular\_function | 2.4965882983 | 1.21473740202e-10 | 4.38513891938e-08 | 75 | cluster\_plot | 3172 |
| 7 | GO:0032549 | ribonucleoside binding | molecular\_function | 2.4965882983 | 1.21473740202e-10 | 4.38513891938e-08 | 75 | cluster\_plot | 3172 |
| 8 | GO:0001882 | nucleoside binding | molecular\_function | 2.49422931723 | 1.26236456837e-10 | 4.38513891938e-08 | 75 | cluster\_plot | 3175 |
| 9 | GO:0032553 | ribonucleotide binding | molecular\_function | 2.46857172138 | 1.91928145597e-10 | 5.92631462904e-08 | 75 | cluster\_plot | 3208 |
| 10 | GO:0017076 | purine nucleotide binding | molecular\_function | 2.45327697714 | 2.46519514107e-10 | 6.71017616491e-08 | 75 | cluster\_plot | 3228 |
| 11 | GO:0097367 | carbohydrate derivative binding | molecular\_function | 2.44872544285 | 2.65606109442e-10 | 6.71017616491e-08 | 75 | cluster\_plot | 3234 |
| 12 | GO:0043168 | anion binding | molecular\_function | 2.30811683635 | 1.19563552138e-09 | 2.76889259492e-07 | 79 | cluster\_plot | 3614 |
| 13 | GO:0036094 | small molecule binding | molecular\_function | 2.23422812981 | 6.32962315984e-09 | 1.32094260486e-06 | 77 | cluster\_plot | 3639 |
| 14 | GO:1901265 | nucleoside phosphate binding | molecular\_function | 2.23842876521 | 7.12995288698e-09 | 1.32094260486e-06 | 76 | cluster\_plot | 3585 |
| 15 | GO:0000166 | nucleotide binding | molecular\_function | 2.23842876521 | 7.12995288698e-09 | 1.32094260486e-06 | 76 | cluster\_plot | 3585 |
| 16 | GO:0004781 | sulfate adenylyltransferase (ATP) activity | molecular\_function | 70.3926940639 | 1.60723275068e-06 | 0.000262735283184 | 4 | cluster\_plot | 6 |
| 17 | GO:0004779 | sulfate adenylyltransferase activity | molecular\_function | 70.3926940639 | 1.60723275068e-06 | 0.000262735283184 | 4 | cluster\_plot | 6 |
| 18 | GO:0000103 | sulfate assimilation | biological\_process | 38.396014944 | 1.0055434952e-05 | 0.00154387499432 | 4 | cluster\_plot | 11 |
| 19 | GO:0046915 | transition metal ion transmembrane transporter activity | molecular\_function | 20.3055848261 | 1.05554605585e-05 | 0.00154387499432 | 5 | cluster\_plot | 26 |
| 20 | GO:0044183 | protein binding involved in protein folding | molecular\_function | 105.589041096 | 1.64959182083e-05 | 0.00228955704897 | 3 | cluster\_plot | 3 |
| 21 | GO:1901363 | heterocyclic compound binding | molecular\_function | 1.66087730115 | 1.78383069231e-05 | 0.00228955704897 | 101 | cluster\_plot | 6421 |
| 22 | GO:0097159 | organic cyclic compound binding | molecular\_function | 1.66010167352 | 1.81253166885e-05 | 0.00228955704897 | 101 | cluster\_plot | 6424 |
| 23 | GO:0007165 | signal transduction | biological\_process | 3.75316496786 | 2.89493622242e-05 | 0.00349783815744 | 15 | cluster\_plot | 422 |
| 24 | GO:0000041 | transition metal ion transport | biological\_process | 15.084148728 | 3.80564757282e-05 | 0.00440662275203 | 5 | cluster\_plot | 35 |
| 25 | GO:0043167 | ion binding | molecular\_function | 1.64193484958 | 5.36104719135e-05 | 0.00595934005791 | 91 | cluster\_plot | 5852 |
| 26 | GO:0070566 | adenylyltransferase activity | molecular\_function | 22.2292718097 | 6.13722397418e-05 | 0.00655974824009 | 4 | cluster\_plot | 19 |
| 27 | GO:0005375 | copper ion transmembrane transporter activity | molecular\_function | 39.595890411 | 0.000131319076882 | 0.0130334183806 | 3 | cluster\_plot | 8 |
| 28 | GO:0035434 | copper ion transmembrane transport | biological\_process | 39.595890411 | 0.000131319076882 | 0.0130334183806 | 3 | cluster\_plot | 8 |
| 29 | GO:0050794 | regulation of cellular process | biological\_process | 1.96679959465 | 0.000369172994713 | 0.0353769569761 | 35 | cluster\_plot | 1879 |
| 30 | GO:0051716 | cellular response to stimulus | biological\_process | 2.83841508322 | 0.000527395832052 | 0.0488544339091 | 15 | cluster\_plot | 558 |
| 31 | GO:0006825 | copper ion transport | biological\_process | 18.6333601934 | 0.000851028885405 | 0.0762906216949 | 3 | cluster\_plot | 17 |
| 32 | GO:0050789 | regulation of biological process | biological\_process | 1.86177150547 | 0.000909370497496 | 0.0789731441419 | 35 | cluster\_plot | 1985 |
| 33 | GO:0065007 | biological regulation | biological\_process | 1.82680001896 | 0.00122763786562 | 0.103381988744 | 35 | cluster\_plot | 2023 |
| 34 | GO:0006790 | sulfur compound metabolic process | biological\_process | 5.55731795242 | 0.00271551150976 | 0.22195313193 | 5 | cluster\_plot | 95 |
| 35 | GO:0015698 | inorganic anion transport | biological\_process | 6.59931506849 | 0.00401862943137 | 0.307914168242 | 4 | cluster\_plot | 64 |
| 36 | GO:0001071 | nucleic acid binding transcription factor activity | molecular\_function | 2.27562588569 | 0.00409961289131 | 0.307914168242 | 15 | cluster\_plot | 696 |
| 37 | GO:0003700 | sequence-specific DNA binding transcription factor activity | molecular\_function | 2.27562588569 | 0.00409961289131 | 0.307914168242 | 15 | cluster\_plot | 696 |
| 38 | GO:0006013 | mannose metabolic process | biological\_process | 23.4642313546 | 0.00462011288503 | 0.322652462777 | 2 | cluster\_plot | 9 |
| 39 | GO:0009719 | response to endogenous stimulus | biological\_process | 9.59900373599 | 0.00476025583802 | 0.322652462777 | 3 | cluster\_plot | 33 |
| 40 | GO:0009725 | response to hormone | biological\_process | 9.59900373599 | 0.00476025583802 | 0.322652462777 | 3 | cluster\_plot | 33 |
| 41 | GO:0010033 | response to organic substance | biological\_process | 9.59900373599 | 0.00476025583802 | 0.322652462777 | 3 | cluster\_plot | 33 |
| 42 | GO:0015291 | secondary active transmembrane transporter activity | molecular\_function | 3.3596513076 | 0.00635072427229 | 0.420206256016 | 7 | cluster\_plot | 220 |
| 43 | GO:0071577 | zinc ion transmembrane transport | biological\_process | 17.598173516 | 0.00750138513348 | 0.457784290211 | 2 | cluster\_plot | 12 |
| 44 | GO:0005385 | zinc ion transmembrane transporter activity | molecular\_function | 17.598173516 | 0.00750138513348 | 0.457784290211 | 2 | cluster\_plot | 12 |
| 45 | GO:0006829 | zinc ion transport | biological\_process | 17.598173516 | 0.00750138513348 | 0.457784290211 | 2 | cluster\_plot | 12 |
| 46 | GO:0050896 | response to stimulus | biological\_process | 1.96953651785 | 0.00757757371347 | 0.457784290211 | 18 | cluster\_plot | 965 |
| 47 | GO:0005315 | inorganic phosphate transmembrane transporter activity | molecular\_function | 16.2444678609 | 0.00860129813047 | 0.478060150091 | 2 | cluster\_plot | 13 |
| 48 | GO:0004559 | alpha-mannosidase activity | molecular\_function | 16.2444678609 | 0.00860129813047 | 0.478060150091 | 2 | cluster\_plot | 13 |
| 49 | GO:0015923 | mannosidase activity | molecular\_function | 16.2444678609 | 0.00860129813047 | 0.478060150091 | 2 | cluster\_plot | 13 |
| 50 | GO:0006817 | phosphate ion transport | biological\_process | 16.2444678609 | 0.00860129813047 | 0.478060150091 | 2 | cluster\_plot | 13 |
| 51 | GO:0034220 | ion transmembrane transport | biological\_process | 3.46193577364 | 0.00970632725335 | 0.528899675237 | 6 | cluster\_plot | 183 |
| 52 | GO:0008271 | secondary active sulfate transmembrane transporter activity | molecular\_function | 14.0785388128 | 0.0110019520143 | 0.576875936747 | 2 | cluster\_plot | 15 |
| 53 | GO:0033926 | glycopeptide alpha-N-acetylgalactosaminidase activity | molecular\_function | 14.0785388128 | 0.0110019520143 | 0.576875936747 | 2 | cluster\_plot | 15 |
| 54 | GO:0042221 | response to chemical | biological\_process | 6.8862418106 | 0.0112130976849 | 0.577059230857 | 3 | cluster\_plot | 46 |
| 55 | GO:0006811 | ion transport | biological\_process | 2.13477065979 | 0.0114267600828 | 0.577363023093 | 13 | cluster\_plot | 643 |
| 56 | GO:0055085 | transmembrane transport | biological\_process | 1.91327820785 | 0.0141657173374 | 0.702973722866 | 16 | cluster\_plot | 883 |
| 57 | GO:0046873 | metal ion transmembrane transporter activity | molecular\_function | 3.4963258641 | 0.0168791394956 | 0.822932081724 | 5 | cluster\_plot | 151 |
| 58 | GO:1901682 | sulfur compound transmembrane transporter activity | molecular\_function | 10.5589041096 | 0.0181122902433 | 0.82514843584 | 2 | cluster\_plot | 20 |
| 59 | GO:0072348 | sulfur compound transport | biological\_process | 10.5589041096 | 0.0181122902433 | 0.82514843584 | 2 | cluster\_plot | 20 |
| 60 | GO:0008272 | sulfate transport | biological\_process | 10.5589041096 | 0.0181122902433 | 0.82514843584 | 2 | cluster\_plot | 20 |
| 61 | GO:0015116 | sulfate transmembrane transporter activity | molecular\_function | 10.5589041096 | 0.0181122902433 | 0.82514843584 | 2 | cluster\_plot | 20 |
| 62 | GO:0006355 | regulation of transcription, DNA-templated | biological\_process | 1.71969122306 | 0.0190366287794 | 0.83874368571 | 20 | cluster\_plot | 1228 |
| 63 | GO:2001141 | regulation of RNA biosynthetic process | biological\_process | 1.71969122306 | 0.0190366287794 | 0.83874368571 | 20 | cluster\_plot | 1228 |
| 64 | GO:0051252 | regulation of RNA metabolic process | biological\_process | 1.71689497717 | 0.0193161554104 | 0.83874368571 | 20 | cluster\_plot | 1230 |
| 65 | GO:0031326 | regulation of cellular biosynthetic process | biological\_process | 1.69077727936 | 0.0221317930316 | 0.904474306395 | 20 | cluster\_plot | 1249 |
| 66 | GO:0010556 | regulation of macromolecule biosynthetic process | biological\_process | 1.69077727936 | 0.0221317930316 | 0.904474306395 | 20 | cluster\_plot | 1249 |
| 67 | GO:2000112 | regulation of cellular macromolecule biosynthetic process | biological\_process | 1.69077727936 | 0.0221317930316 | 0.904474306395 | 20 | cluster\_plot | 1249 |
| 68 | GO:0009889 | regulation of biosynthetic process | biological\_process | 1.69077727936 | 0.0221317930316 | 0.904474306395 | 20 | cluster\_plot | 1249 |
| 69 | GO:1901677 | phosphate transmembrane transporter activity | molecular\_function | 9.18165574747 | 0.0230870605966 | 0.929839730406 | 2 | cluster\_plot | 23 |
| 70 | GO:0006820 | anion transport | biological\_process | 3.199667912 | 0.0234317250511 | 0.930239484528 | 5 | cluster\_plot | 165 |
| 71 | GO:0032296 | double-stranded RNA-specific ribonuclease activity | molecular\_function | 8.79908675799 | 0.0248559695143 | 0.959371378892 | 2 | cluster\_plot | 24 |
| 72 | GO:0004525 | ribonuclease III activity | molecular\_function | 8.79908675799 | 0.0248559695143 | 0.959371378892 | 2 | cluster\_plot | 24 |
| 73 | GO:0010468 | regulation of gene expression | biological\_process | 1.66412988331 | 0.0254230362675 | 0.967816682017 | 20 | cluster\_plot | 1269 |
| 74 | GO:0022857 | transmembrane transporter activity | molecular\_function | 1.81009784736 | 0.0257886355756 | 0.968467814387 | 15 | cluster\_plot | 875 |
| 75 | GO:0009668 | plastid membrane organization | biological\_process | 52.7945205479 | 0.0279750570926 | 0.984084603297 | 1 | cluster\_plot | 2 |
| 76 | GO:0016419 | S-malonyltransferase activity | molecular\_function | 52.7945205479 | 0.0279750570926 | 0.984084603297 | 1 | cluster\_plot | 2 |
| 77 | GO:0016420 | malonyltransferase activity | molecular\_function | 52.7945205479 | 0.0279750570926 | 0.984084603297 | 1 | cluster\_plot | 2 |
| 78 | GO:0004314 | [acyl-carrier-protein] S-malonyltransferase activity | molecular\_function | 52.7945205479 | 0.0279750570926 | 0.984084603297 | 1 | cluster\_plot | 2 |
| 79 | GO:0010027 | thylakoid membrane organization | biological\_process | 52.7945205479 | 0.0279750570926 | 0.984084603297 | 1 | cluster\_plot | 2 |
| 80 | GO:0051274 | beta-glucan biosynthetic process | biological\_process | 4.72786751176 | 0.0287932966641 | 1.0 | 3 | cluster\_plot | 67 |
| 81 | GO:0005488 | binding | molecular\_function | 1.22884588387 | 0.0291660903792 | 1.0 | 134 | cluster\_plot | 11514 |
| 82 | GO:0060255 | regulation of macromolecule metabolic process | biological\_process | 1.6307187814 | 0.0302380713471 | 1.0 | 20 | cluster\_plot | 1295 |
| 83 | GO:0051171 | regulation of nitrogen compound metabolic process | biological\_process | 1.6307187814 | 0.0302380713471 | 1.0 | 20 | cluster\_plot | 1295 |
| 84 | GO:0019219 | regulation of nucleobase-containing compound metabolic process | biological\_process | 1.6307187814 | 0.0302380713471 | 1.0 | 20 | cluster\_plot | 1295 |
| 85 | GO:0051273 | beta-glucan metabolic process | biological\_process | 4.5252446184 | 0.0320449533245 | 1.0 | 3 | cluster\_plot | 70 |
| 86 | GO:0022890 | inorganic cation transmembrane transporter activity | molecular\_function | 2.39198474975 | 0.0322937686912 | 1.0 | 7 | cluster\_plot | 309 |
| 87 | GO:0016891 | endoribonuclease activity, producing 5'-phosphomonoesters | molecular\_function | 7.2820028342 | 0.0344774302027 | 1.0 | 2 | cluster\_plot | 29 |
| 88 | GO:0080090 | regulation of primary metabolic process | biological\_process | 1.5962062146 | 0.0361494971654 | 1.0 | 20 | cluster\_plot | 1323 |
| 89 | GO:0016021 | integral component of membrane | cellular\_component | 1.66051220965 | 0.0369915862084 | 1.0 | 17 | cluster\_plot | 1081 |
| 90 | GO:0031323 | regulation of cellular metabolic process | biological\_process | 1.59139474146 | 0.0370585007675 | 1.0 | 20 | cluster\_plot | 1327 |
| 91 | GO:0015075 | ion transmembrane transporter activity | molecular\_function | 1.96994479657 | 0.0378036343906 | 1.0 | 10 | cluster\_plot | 536 |
| 92 | GO:0035251 | UDP-glucosyltransferase activity | molecular\_function | 4.06111696523 | 0.0416056402688 | 1.0 | 3 | cluster\_plot | 78 |
| 93 | GO:0009250 | glucan biosynthetic process | biological\_process | 4.00971042136 | 0.0428903703864 | 1.0 | 3 | cluster\_plot | 79 |
| 94 | GO:0030001 | metal ion transport | biological\_process | 2.40887546226 | 0.0441356696521 | 1.0 | 6 | cluster\_plot | 263 |
| 95 | GO:0016570 | histone modification | biological\_process | 6.03365949119 | 0.0475854876013 | 1.0 | 2 | cluster\_plot | 35 |
| 96 | GO:0016893 | endonuclease activity, active with either ribo- or deoxyribonucleic acids and producing 5'-phosphomonoesters | molecular\_function | 6.03365949119 | 0.0475854876013 | 1.0 | 2 | cluster\_plot | 35 |
| 97 | GO:0016569 | covalent chromatin modification | biological\_process | 6.03365949119 | 0.0475854876013 | 1.0 | 2 | cluster\_plot | 35 |
| 98 | GO:0005215 | transporter activity | molecular\_function | 1.5885076979 | 0.0511641760653 | 1.0 | 17 | cluster\_plot | 1130 |
| 99 | GO:0046527 | glucosyltransferase activity | molecular\_function | 3.6410014171 | 0.053866107391 | 1.0 | 3 | cluster\_plot | 87 |
| 100 | GO:0016759 | cellulose synthase activity | molecular\_function | 5.55731795242 | 0.0547123866095 | 1.0 | 2 | cluster\_plot | 38 |
| 101 | GO:0016760 | cellulose synthase (UDP-forming) activity | molecular\_function | 5.55731795242 | 0.0547123866095 | 1.0 | 2 | cluster\_plot | 38 |
| 102 | GO:0016721 | oxidoreductase activity, acting on superoxide radicals as acceptor | molecular\_function | 21.1178082192 | 0.0551649144868 | 1.0 | 1 | cluster\_plot | 5 |
| 103 | GO:0004784 | superoxide dismutase activity | molecular\_function | 21.1178082192 | 0.0551649144868 | 1.0 | 1 | cluster\_plot | 5 |
| 104 | GO:0000271 | polysaccharide biosynthetic process | biological\_process | 3.599626401 | 0.055323657579 | 1.0 | 3 | cluster\_plot | 88 |
| 105 | GO:0033692 | cellular polysaccharide biosynthetic process | biological\_process | 3.599626401 | 0.055323657579 | 1.0 | 3 | cluster\_plot | 88 |
| 106 | GO:0022892 | substrate-specific transporter activity | molecular\_function | 1.74134850383 | 0.0616296460793 | 1.0 | 11 | cluster\_plot | 667 |
| 107 | GO:0016568 | chromatin modification | biological\_process | 5.15068493151 | 0.0621857253305 | 1.0 | 2 | cluster\_plot | 41 |
| 108 | GO:0016417 | S-acyltransferase activity | molecular\_function | 17.598173516 | 0.064057551414 | 1.0 | 1 | cluster\_plot | 6 |
| 109 | GO:0004521 | endoribonuclease activity | molecular\_function | 5.02804957599 | 0.0647494518721 | 1.0 | 2 | cluster\_plot | 42 |
| 110 | GO:0019222 | regulation of metabolic process | biological\_process | 1.47780323437 | 0.066212426942 | 1.0 | 20 | cluster\_plot | 1429 |
| 111 | GO:0009698 | phenylpropanoid metabolic process | biological\_process | 4.91111819051 | 0.0673479711914 | 1.0 | 2 | cluster\_plot | 43 |
| 112 | GO:0046271 | phenylpropanoid catabolic process | biological\_process | 4.91111819051 | 0.0673479711914 | 1.0 | 2 | cluster\_plot | 43 |
| 113 | GO:0046274 | lignin catabolic process | biological\_process | 4.91111819051 | 0.0673479711914 | 1.0 | 2 | cluster\_plot | 43 |
| 114 | GO:0052716 | hydroquinone:oxygen oxidoreductase activity | molecular\_function | 4.91111819051 | 0.0673479711914 | 1.0 | 2 | cluster\_plot | 43 |
| 115 | GO:0009808 | lignin metabolic process | biological\_process | 4.91111819051 | 0.0673479711914 | 1.0 | 2 | cluster\_plot | 43 |
| 116 | GO:0008289 | lipid binding | molecular\_function | 3.26564044626 | 0.0692638838948 | 1.0 | 3 | cluster\_plot | 97 |
| 117 | GO:0004312 | fatty acid synthase activity | molecular\_function | 15.084148728 | 0.0728662092993 | 1.0 | 1 | cluster\_plot | 7 |
| 118 | GO:0019748 | secondary metabolic process | biological\_process | 4.49315068493 | 0.0780724323638 | 1.0 | 2 | cluster\_plot | 47 |
| 119 | GO:0047325 | inositol tetrakisphosphate 1-kinase activity | molecular\_function | 13.198630137 | 0.0815916838998 | 1.0 | 1 | cluster\_plot | 8 |
| 120 | GO:0051765 | inositol tetrakisphosphate kinase activity | molecular\_function | 13.198630137 | 0.0815916838998 | 1.0 | 1 | cluster\_plot | 8 |
| 121 | GO:0032957 | inositol trisphosphate metabolic process | biological\_process | 13.198630137 | 0.0815916838998 | 1.0 | 1 | cluster\_plot | 8 |
| 122 | GO:0052726 | inositol-1,3,4-trisphosphate 5-kinase activity | molecular\_function | 13.198630137 | 0.0815916838998 | 1.0 | 1 | cluster\_plot | 8 |
| 123 | GO:0052725 | inositol-1,3,4-trisphosphate 6-kinase activity | molecular\_function | 13.198630137 | 0.0815916838998 | 1.0 | 1 | cluster\_plot | 8 |
| 124 | GO:0043647 | inositol phosphate metabolic process | biological\_process | 13.198630137 | 0.0815916838998 | 1.0 | 1 | cluster\_plot | 8 |
| 125 | GO:0030244 | cellulose biosynthetic process | biological\_process | 4.22356164384 | 0.0864389664914 | 1.0 | 2 | cluster\_plot | 50 |
| 126 | GO:0022891 | substrate-specific transmembrane transporter activity | molecular\_function | 1.67336039772 | 0.0874263716932 | 1.0 | 10 | cluster\_plot | 631 |
| 127 | GO:0008509 | anion transmembrane transporter activity | molecular\_function | 2.93302891933 | 0.0882029094639 | 1.0 | 3 | cluster\_plot | 108 |
| 128 | GO:0044425 | membrane part | cellular\_component | 1.43197129252 | 0.0891633888876 | 1.0 | 19 | cluster\_plot | 1401 |
| 129 | GO:0006743 | ubiquinone metabolic process | biological\_process | 11.7321156773 | 0.0902347633752 | 1.0 | 1 | cluster\_plot | 9 |
| 130 | GO:0006744 | ubiquinone biosynthetic process | biological\_process | 11.7321156773 | 0.0902347633752 | 1.0 | 1 | cluster\_plot | 9 |
| 131 | GO:0044765 | single-organism transport | biological\_process | 1.44313040222 | 0.0907681302445 | 1.0 | 18 | cluster\_plot | 1317 |
| 132 | GO:0030243 | cellulose metabolic process | biological\_process | 3.98449211683 | 0.0950581843506 | 1.0 | 2 | cluster\_plot | 53 |
| 133 | GO:0015103 | inorganic anion transmembrane transporter activity | molecular\_function | 3.98449211683 | 0.0950581843506 | 1.0 | 2 | cluster\_plot | 53 |
| 134 | GO:0016682 | oxidoreductase activity, acting on diphenols and related substances as donors, oxygen as acceptor | molecular\_function | 3.98449211683 | 0.0950581843506 | 1.0 | 2 | cluster\_plot | 53 |
| 135 | GO:0008324 | cation transmembrane transporter activity | molecular\_function | 1.82499577203 | 0.0987761353811 | 1.0 | 7 | cluster\_plot | 405 |
| 136 | GO:0042181 | ketone biosynthetic process | biological\_process | 10.5589041096 | 0.0987962284221 | 1.0 | 1 | cluster\_plot | 10 |
| 137 | GO:1901661 | quinone metabolic process | biological\_process | 10.5589041096 | 0.0987962284221 | 1.0 | 1 | cluster\_plot | 10 |
| 138 | GO:1901663 | quinone biosynthetic process | biological\_process | 10.5589041096 | 0.0987962284221 | 1.0 | 1 | cluster\_plot | 10 |
| 139 | GO:0042180 | cellular ketone metabolic process | biological\_process | 10.5589041096 | 0.0987962284221 | 1.0 | 1 | cluster\_plot | 10 |
| 140 | GO:0006812 | cation transport | biological\_process | 1.73809121145 | 0.100140998557 | 1.0 | 8 | cluster\_plot | 486 |
| 141 | GO:0004806 | triglyceride lipase activity | molecular\_function | 3.8396014944 | 0.100934396946 | 1.0 | 2 | cluster\_plot | 55 |
| 142 | GO:0004540 | ribonuclease activity | molecular\_function | 3.8396014944 | 0.100934396946 | 1.0 | 2 | cluster\_plot | 55 |
| 143 | GO:0006352 | DNA-templated transcription, initiation | biological\_process | 3.70487863494 | 0.106907727254 | 1.0 | 2 | cluster\_plot | 57 |
| 144 | GO:0016413 | O-acetyltransferase activity | molecular\_function | 9.59900373599 | 0.107276852309 | 1.0 | 1 | cluster\_plot | 11 |
| 145 | GO:0016412 | serine O-acyltransferase activity | molecular\_function | 9.59900373599 | 0.107276852309 | 1.0 | 1 | cluster\_plot | 11 |
| 146 | GO:0009001 | serine O-acetyltransferase activity | molecular\_function | 9.59900373599 | 0.107276852309 | 1.0 | 1 | cluster\_plot | 11 |
| 147 | GO:0051766 | inositol trisphosphate kinase activity | molecular\_function | 9.59900373599 | 0.107276852309 | 1.0 | 1 | cluster\_plot | 11 |
| 148 | GO:0022804 | active transmembrane transporter activity | molecular\_function | 1.7853219509 | 0.107282394273 | 1.0 | 7 | cluster\_plot | 414 |
| 149 | GO:0030247 | polysaccharide binding | molecular\_function | 3.6410014171 | 0.109929012431 | 1.0 | 2 | cluster\_plot | 58 |
| 150 | GO:0001871 | pattern binding | molecular\_function | 3.6410014171 | 0.109929012431 | 1.0 | 2 | cluster\_plot | 58 |
| 151 | GO:0016679 | oxidoreductase activity, acting on diphenols and related substances as donors | molecular\_function | 3.6410014171 | 0.109929012431 | 1.0 | 2 | cluster\_plot | 58 |
| 152 | GO:0034637 | cellular carbohydrate biosynthetic process | biological\_process | 2.6397260274 | 0.111019190208 | 1.0 | 3 | cluster\_plot | 120 |
| 153 | GO:0032040 | small-subunit processome | cellular\_component | 8.79908675799 | 0.115677400927 | 1.0 | 1 | cluster\_plot | 12 |
| 154 | GO:0016298 | lipase activity | molecular\_function | 3.40609809987 | 0.122228759062 | 1.0 | 2 | cluster\_plot | 62 |
| 155 | GO:0019318 | hexose metabolic process | biological\_process | 3.40609809987 | 0.122228759062 | 1.0 | 2 | cluster\_plot | 62 |
| 156 | GO:0003674 | molecular\_function | molecular\_function | 1.11585344454 | 0.127663445314 | 1.0 | 198 | cluster\_plot | 18736 |
| 157 | GO:0004519 | endonuclease activity | molecular\_function | 3.29965753425 | 0.128498027488 | 1.0 | 2 | cluster\_plot | 64 |
| 158 | GO:0044042 | glucan metabolic process | biological\_process | 2.45555909525 | 0.129438608358 | 1.0 | 3 | cluster\_plot | 129 |
| 159 | GO:0006073 | cellular glucan metabolic process | biological\_process | 2.45555909525 | 0.129438608358 | 1.0 | 3 | cluster\_plot | 129 |
| 160 | GO:0072593 | reactive oxygen species metabolic process | biological\_process | 7.54207436399 | 0.132241299679 | 1.0 | 1 | cluster\_plot | 14 |
| 161 | GO:0061024 | membrane organization | biological\_process | 7.54207436399 | 0.132241299679 | 1.0 | 1 | cluster\_plot | 14 |
| 162 | GO:0044802 | single-organism membrane organization | biological\_process | 7.54207436399 | 0.132241299679 | 1.0 | 1 | cluster\_plot | 14 |
| 163 | GO:0006801 | superoxide metabolic process | biological\_process | 7.54207436399 | 0.132241299679 | 1.0 | 1 | cluster\_plot | 14 |
| 164 | GO:0005996 | monosaccharide metabolic process | biological\_process | 3.1519116745 | 0.13803671935 | 1.0 | 2 | cluster\_plot | 67 |
| 165 | GO:0016779 | nucleotidyltransferase activity | molecular\_function | 2.05027264264 | 0.138477758895 | 1.0 | 4 | cluster\_plot | 206 |
| 166 | GO:0016051 | carbohydrate biosynthetic process | biological\_process | 2.36393375588 | 0.140103242499 | 1.0 | 3 | cluster\_plot | 134 |
| 167 | GO:0030684 | preribosome | cellular\_component | 7.03926940639 | 0.140406145513 | 1.0 | 1 | cluster\_plot | 15 |
| 168 | GO:0006465 | signal peptide processing | biological\_process | 7.03926940639 | 0.140406145513 | 1.0 | 1 | cluster\_plot | 15 |
| 169 | GO:0004743 | pyruvate kinase activity | molecular\_function | 7.03926940639 | 0.140406145513 | 1.0 | 1 | cluster\_plot | 15 |
| 170 | GO:0031420 | alkali metal ion binding | molecular\_function | 7.03926940639 | 0.140406145513 | 1.0 | 1 | cluster\_plot | 15 |
| 171 | GO:0030955 | potassium ion binding | molecular\_function | 7.03926940639 | 0.140406145513 | 1.0 | 1 | cluster\_plot | 15 |
| 172 | GO:0015299 | solute:proton antiporter activity | molecular\_function | 3.06055191582 | 0.144478543926 | 1.0 | 2 | cluster\_plot | 69 |
| 173 | GO:0015298 | solute:cation antiporter activity | molecular\_function | 3.06055191582 | 0.144478543926 | 1.0 | 2 | cluster\_plot | 69 |
| 174 | GO:0072509 | divalent inorganic cation transmembrane transporter activity | molecular\_function | 3.06055191582 | 0.144478543926 | 1.0 | 2 | cluster\_plot | 69 |
| 175 | GO:0019344 | cysteine biosynthetic process | biological\_process | 6.59931506849 | 0.148493907603 | 1.0 | 1 | cluster\_plot | 16 |
| 176 | GO:0006535 | cysteine biosynthetic process from serine | biological\_process | 6.59931506849 | 0.148493907603 | 1.0 | 1 | cluster\_plot | 16 |
| 177 | GO:0044264 | cellular polysaccharide metabolic process | biological\_process | 2.29541393687 | 0.148838050044 | 1.0 | 3 | cluster\_plot | 138 |
| 178 | GO:0005543 | phospholipid binding | molecular\_function | 2.9743391858 | 0.150981005434 | 1.0 | 2 | cluster\_plot | 71 |
| 179 | GO:0000148 | 1,3-beta-D-glucan synthase complex | cellular\_component | 6.21112006446 | 0.156505316149 | 1.0 | 1 | cluster\_plot | 17 |
| 180 | GO:0006075 | (1->3)-beta-D-glucan biosynthetic process | biological\_process | 6.21112006446 | 0.156505316149 | 1.0 | 1 | cluster\_plot | 17 |
| 181 | GO:0003843 | 1,3-beta-D-glucan synthase activity | molecular\_function | 6.21112006446 | 0.156505316149 | 1.0 | 1 | cluster\_plot | 17 |
| 182 | GO:0006074 | (1->3)-beta-D-glucan metabolic process | biological\_process | 6.21112006446 | 0.156505316149 | 1.0 | 1 | cluster\_plot | 17 |
| 183 | GO:0044723 | single-organism carbohydrate metabolic process | biological\_process | 1.65846661407 | 0.163461196213 | 1.0 | 6 | cluster\_plot | 382 |
| 184 | GO:0005634 | nucleus | cellular\_component | 1.45439450545 | 0.1637517239 | 1.0 | 10 | cluster\_plot | 726 |
| 185 | GO:0006325 | chromatin organization | biological\_process | 2.81570776256 | 0.164149761168 | 1.0 | 2 | cluster\_plot | 75 |
| 186 | GO:0009982 | pseudouridine synthase activity | molecular\_function | 5.86605783866 | 0.164441094416 | 1.0 | 1 | cluster\_plot | 18 |
| 187 | GO:0008194 | UDP-glycosyltransferase activity | molecular\_function | 2.16963783074 | 0.166801867515 | 1.0 | 3 | cluster\_plot | 146 |
| 188 | GO:0005515 | protein binding | molecular\_function | 1.18343881677 | 0.167592005945 | 1.0 | 45 | cluster\_plot | 4015 |
| 189 | GO:0006534 | cysteine metabolic process | biological\_process | 5.55731795242 | 0.172301958776 | 1.0 | 1 | cluster\_plot | 19 |
| 190 | GO:0000159 | protein phosphatase type 2A complex | cellular\_component | 5.27945205479 | 0.180088618774 | 1.0 | 1 | cluster\_plot | 20 |
| 191 | GO:0008601 | protein phosphatase type 2A regulator activity | molecular\_function | 5.27945205479 | 0.180088618774 | 1.0 | 1 | cluster\_plot | 20 |
| 192 | GO:0008287 | protein serine/threonine phosphatase complex | cellular\_component | 5.27945205479 | 0.180088618774 | 1.0 | 1 | cluster\_plot | 20 |
| 193 | GO:0019888 | protein phosphatase regulator activity | molecular\_function | 5.02804957599 | 0.187801777266 | 1.0 | 1 | cluster\_plot | 21 |
| 194 | GO:0001522 | pseudouridine synthesis | biological\_process | 5.02804957599 | 0.187801777266 | 1.0 | 1 | cluster\_plot | 21 |
| 195 | GO:0051861 | glycolipid binding | molecular\_function | 5.02804957599 | 0.187801777266 | 1.0 | 1 | cluster\_plot | 21 |
| 196 | GO:0019208 | phosphatase regulator activity | molecular\_function | 5.02804957599 | 0.187801777266 | 1.0 | 1 | cluster\_plot | 21 |
| 197 | GO:0046836 | glycolipid transport | biological\_process | 5.02804957599 | 0.187801777266 | 1.0 | 1 | cluster\_plot | 21 |
| 198 | GO:0017089 | glycolipid transporter activity | molecular\_function | 5.02804957599 | 0.187801777266 | 1.0 | 1 | cluster\_plot | 21 |
| 199 | GO:0043565 | sequence-specific DNA binding | molecular\_function | 1.56428209031 | 0.195061829677 | 1.0 | 6 | cluster\_plot | 405 |
| 200 | GO:0051604 | protein maturation | biological\_process | 4.59082787373 | 0.203010367731 | 1.0 | 1 | cluster\_plot | 23 |
| 201 | GO:0016485 | protein processing | biological\_process | 4.59082787373 | 0.203010367731 | 1.0 | 1 | cluster\_plot | 23 |
| 202 | GO:0048046 | apoplast | cellular\_component | 2.34642313546 | 0.214966135995 | 1.0 | 2 | cluster\_plot | 90 |
| 203 | GO:0051276 | chromosome organization | biological\_process | 2.34642313546 | 0.214966135995 | 1.0 | 2 | cluster\_plot | 90 |
| 204 | GO:0015297 | antiporter activity | molecular\_function | 1.89680912148 | 0.216520163148 | 1.0 | 3 | cluster\_plot | 167 |
| 205 | GO:0005976 | polysaccharide metabolic process | biological\_process | 1.87436167626 | 0.221409464924 | 1.0 | 3 | cluster\_plot | 169 |
| 206 | GO:0009070 | serine family amino acid biosynthetic process | biological\_process | 4.06111696523 | 0.225289182116 | 1.0 | 1 | cluster\_plot | 26 |
| 207 | GO:0000097 | sulfur amino acid biosynthetic process | biological\_process | 3.91070522577 | 0.232575721322 | 1.0 | 1 | cluster\_plot | 27 |
| 208 | GO:1902589 | single-organism organelle organization | biological\_process | 2.1997716895 | 0.235686426266 | 1.0 | 2 | cluster\_plot | 96 |
| 209 | GO:1901264 | carbohydrate derivative transport | biological\_process | 3.5196347032 | 0.254025346112 | 1.0 | 1 | cluster\_plot | 30 |
| 210 | GO:0043227 | membrane-bounded organelle | cellular\_component | 1.2908195733 | 0.25944875263 | 1.0 | 10 | cluster\_plot | 818 |
| 211 | GO:0043231 | intracellular membrane-bounded organelle | cellular\_component | 1.2908195733 | 0.25944875263 | 1.0 | 10 | cluster\_plot | 818 |
| 212 | GO:0000096 | sulfur amino acid metabolic process | biological\_process | 3.29965753425 | 0.26798986971 | 1.0 | 1 | cluster\_plot | 32 |
| 213 | GO:0008374 | O-acyltransferase activity | molecular\_function | 3.29965753425 | 0.26798986971 | 1.0 | 1 | cluster\_plot | 32 |
| 214 | GO:0005576 | extracellular region | cellular\_component | 1.99224605841 | 0.270396491111 | 1.0 | 2 | cluster\_plot | 106 |
| 215 | GO:0019439 | aromatic compound catabolic process | biological\_process | 1.99224605841 | 0.270396491111 | 1.0 | 2 | cluster\_plot | 106 |
| 216 | GO:0015171 | amino acid transmembrane transporter activity | molecular\_function | 3.10556003223 | 0.281692104298 | 1.0 | 1 | cluster\_plot | 34 |
| 217 | GO:1901361 | organic cyclic compound catabolic process | biological\_process | 1.90250524497 | 0.287747160961 | 1.0 | 2 | cluster\_plot | 111 |
| 218 | GO:0006518 | peptide metabolic process | biological\_process | 2.93302891933 | 0.295136992759 | 1.0 | 1 | cluster\_plot | 36 |
| 219 | GO:0003333 | amino acid transmembrane transport | biological\_process | 2.77865897621 | 0.308329384531 | 1.0 | 1 | cluster\_plot | 38 |
| 220 | GO:0006563 | L-serine metabolic process | biological\_process | 2.77865897621 | 0.308329384531 | 1.0 | 1 | cluster\_plot | 38 |
| 221 | GO:0006865 | amino acid transport | biological\_process | 2.77865897621 | 0.308329384531 | 1.0 | 1 | cluster\_plot | 38 |
| 222 | GO:0004518 | nuclease activity | molecular\_function | 1.78964476434 | 0.3119439206 | 1.0 | 2 | cluster\_plot | 118 |
| 223 | GO:1901505 | carbohydrate derivative transporter activity | molecular\_function | 2.70741131015 | 0.31483238448 | 1.0 | 1 | cluster\_plot | 39 |
| 224 | GO:0006733 | oxidoreduction coenzyme metabolic process | biological\_process | 2.6397260274 | 0.321274037315 | 1.0 | 1 | cluster\_plot | 40 |
| 225 | GO:0006869 | lipid transport | biological\_process | 2.57534246575 | 0.327654923774 | 1.0 | 1 | cluster\_plot | 41 |
| 226 | GO:0009523 | photosystem II | cellular\_component | 2.514024788 | 0.333975618969 | 1.0 | 1 | cluster\_plot | 42 |
| 227 | GO:0019751 | polyol metabolic process | biological\_process | 2.514024788 | 0.333975618969 | 1.0 | 1 | cluster\_plot | 42 |
| 228 | GO:0044459 | plasma membrane part | cellular\_component | 2.514024788 | 0.333975618969 | 1.0 | 1 | cluster\_plot | 42 |
| 229 | GO:0043177 | organic acid binding | molecular\_function | 2.45555909525 | 0.34023669265 | 1.0 | 1 | cluster\_plot | 43 |
| 230 | GO:0016597 | amino acid binding | molecular\_function | 2.45555909525 | 0.34023669265 | 1.0 | 1 | cluster\_plot | 43 |
| 231 | GO:0031406 | carboxylic acid binding | molecular\_function | 2.45555909525 | 0.34023669265 | 1.0 | 1 | cluster\_plot | 43 |
| 232 | GO:0044712 | single-organism catabolic process | biological\_process | 1.45306019857 | 0.345110698038 | 1.0 | 3 | cluster\_plot | 218 |
| 233 | GO:0016772 | transferase activity, transferring phosphorus-containing groups | molecular\_function | 1.10419912257 | 0.351754088133 | 1.0 | 24 | cluster\_plot | 2295 |
| 234 | GO:0005342 | organic acid transmembrane transporter activity | molecular\_function | 2.34642313546 | 0.352582227102 | 1.0 | 1 | cluster\_plot | 45 |
| 235 | GO:0046943 | carboxylic acid transmembrane transporter activity | molecular\_function | 2.34642313546 | 0.352582227102 | 1.0 | 1 | cluster\_plot | 45 |
| 236 | GO:0006810 | transport | biological\_process | 1.11641167547 | 0.355651088451 | 1.0 | 19 | cluster\_plot | 1797 |
| 237 | GO:0009987 | cellular process | biological\_process | 1.05748387982 | 0.355727056103 | 1.0 | 73 | cluster\_plot | 7289 |
| 238 | GO:0003677 | DNA binding | molecular\_function | 1.12628310502 | 0.35907932759 | 1.0 | 16 | cluster\_plot | 1500 |
| 239 | GO:0051234 | establishment of localization | biological\_process | 1.11207970112 | 0.36155882408 | 1.0 | 19 | cluster\_plot | 1804 |
| 240 | GO:0006066 | alcohol metabolic process | biological\_process | 2.1997716895 | 0.370667301303 | 1.0 | 1 | cluster\_plot | 48 |
| 241 | GO:0052689 | carboxylic ester hydrolase activity | molecular\_function | 1.38326254711 | 0.373040865135 | 1.0 | 3 | cluster\_plot | 229 |
| 242 | GO:0009451 | RNA modification | biological\_process | 2.15487838971 | 0.376582310029 | 1.0 | 1 | cluster\_plot | 49 |
| 243 | GO:0000287 | magnesium ion binding | molecular\_function | 1.49771689498 | 0.389738825524 | 1.0 | 2 | cluster\_plot | 141 |
| 244 | GO:0043412 | macromolecule modification | biological\_process | 1.08397522357 | 0.390275956935 | 1.0 | 22 | cluster\_plot | 2143 |
| 245 | GO:0005319 | lipid transporter activity | molecular\_function | 2.03055848261 | 0.393994748698 | 1.0 | 1 | cluster\_plot | 52 |
| 246 | GO:0044262 | cellular carbohydrate metabolic process | biological\_process | 1.32538545309 | 0.398216666383 | 1.0 | 3 | cluster\_plot | 239 |
| 247 | GO:0006096 | glycolytic process | biological\_process | 1.9198007472 | 0.410919234053 | 1.0 | 1 | cluster\_plot | 55 |
| 248 | GO:0008514 | organic anion transmembrane transporter activity | molecular\_function | 1.9198007472 | 0.410919234053 | 1.0 | 1 | cluster\_plot | 55 |
| 249 | GO:1901615 | organic hydroxy compound metabolic process | biological\_process | 1.885518591 | 0.416454677603 | 1.0 | 1 | cluster\_plot | 56 |
| 250 | GO:0009069 | serine family amino acid metabolic process | biological\_process | 1.885518591 | 0.416454677603 | 1.0 | 1 | cluster\_plot | 56 |
| 251 | GO:0016773 | phosphotransferase activity, alcohol group as acceptor | molecular\_function | 1.06871499085 | 0.420727026822 | 1.0 | 20 | cluster\_plot | 1976 |
| 252 | GO:0016884 | carbon-nitrogen ligase activity, with glutamine as amido-N-donor | molecular\_function | 1.85243931747 | 0.421937930097 | 1.0 | 1 | cluster\_plot | 57 |
| 253 | GO:0036211 | protein modification process | biological\_process | 1.06553092889 | 0.422551699366 | 1.0 | 21 | cluster\_plot | 2081 |
| 254 | GO:0006464 | cellular protein modification process | biological\_process | 1.06553092889 | 0.422551699366 | 1.0 | 21 | cluster\_plot | 2081 |
| 255 | GO:0009521 | photosystem | cellular\_component | 1.82050070855 | 0.4273694853 | 1.0 | 1 | cluster\_plot | 58 |
| 256 | GO:0008762 | UDP-N-acetylmuramate dehydrogenase activity | molecular\_function | 1.78964476434 | 0.432749832339 | 1.0 | 1 | cluster\_plot | 59 |
| 257 | GO:0043603 | cellular amide metabolic process | biological\_process | 1.7598173516 | 0.438079455486 | 1.0 | 1 | cluster\_plot | 60 |
| 258 | GO:0006468 | protein phosphorylation | biological\_process | 1.06060420744 | 0.439617424162 | 1.0 | 18 | cluster\_plot | 1792 |
| 259 | GO:0015078 | hydrogen ion transmembrane transporter activity | molecular\_function | 1.34508332606 | 0.441461724937 | 1.0 | 2 | cluster\_plot | 157 |
| 260 | GO:0044436 | thylakoid part | cellular\_component | 1.73096788682 | 0.443358834672 | 1.0 | 1 | cluster\_plot | 61 |
| 261 | GO:0008026 | ATP-dependent helicase activity | molecular\_function | 1.33657014045 | 0.444613895852 | 1.0 | 2 | cluster\_plot | 158 |
| 262 | GO:0070035 | purine NTP-dependent helicase activity | molecular\_function | 1.33657014045 | 0.444613895852 | 1.0 | 2 | cluster\_plot | 158 |
| 263 | GO:0016301 | kinase activity | molecular\_function | 1.04699098756 | 0.454961745992 | 1.0 | 20 | cluster\_plot | 2017 |
| 264 | GO:0016020 | membrane | cellular\_component | 1.0596749644 | 0.455441484759 | 1.0 | 14 | cluster\_plot | 1395 |
| 265 | GO:0006090 | pyruvate metabolic process | biological\_process | 1.64982876712 | 0.458900238411 | 1.0 | 1 | cluster\_plot | 64 |
| 266 | GO:0044272 | sulfur compound biosynthetic process | biological\_process | 1.64982876712 | 0.458900238411 | 1.0 | 1 | cluster\_plot | 64 |
| 267 | GO:0004672 | protein kinase activity | molecular\_function | 1.04314091094 | 0.46613405781 | 1.0 | 18 | cluster\_plot | 1822 |
| 268 | GO:0071705 | nitrogen compound transport | biological\_process | 1.599833956 | 0.469018547913 | 1.0 | 1 | cluster\_plot | 66 |
| 269 | GO:0015979 | photosynthesis | biological\_process | 1.57595583725 | 0.474006287254 | 1.0 | 1 | cluster\_plot | 67 |
| 270 | GO:0030246 | carbohydrate binding | molecular\_function | 1.24957445084 | 0.478603795266 | 1.0 | 2 | cluster\_plot | 169 |
| 271 | GO:0016407 | acetyltransferase activity | molecular\_function | 1.55278001612 | 0.478947016195 | 1.0 | 1 | cluster\_plot | 68 |
| 272 | GO:0016310 | phosphorylation | biological\_process | 1.03293627159 | 0.48197639942 | 1.0 | 18 | cluster\_plot | 1840 |
| 273 | GO:0016866 | intramolecular transferase activity | molecular\_function | 1.5084148728 | 0.488689216994 | 1.0 | 1 | cluster\_plot | 70 |
| 274 | GO:1902494 | catalytic complex | cellular\_component | 1.1313111546 | 0.497572104912 | 1.0 | 3 | cluster\_plot | 280 |
| 275 | GO:0006996 | organelle organization | biological\_process | 1.18639372018 | 0.505435562657 | 1.0 | 2 | cluster\_plot | 178 |
| 276 | GO:0015849 | organic acid transport | biological\_process | 1.42687893373 | 0.507628774632 | 1.0 | 1 | cluster\_plot | 74 |
| 277 | GO:0046942 | carboxylic acid transport | biological\_process | 1.42687893373 | 0.507628774632 | 1.0 | 1 | cluster\_plot | 74 |
| 278 | GO:0003723 | RNA binding | molecular\_function | 1.04131204237 | 0.526836798532 | 1.0 | 5 | cluster\_plot | 507 |
| 279 | GO:1990234 | transferase complex | cellular\_component | 1.11734435022 | 0.536972084931 | 1.0 | 2 | cluster\_plot | 189 |
| 280 | GO:0006091 | generation of precursor metabolites and energy | biological\_process | 1.30356840859 | 0.539095188232 | 1.0 | 1 | cluster\_plot | 81 |
| 281 | GO:0044724 | single-organism carbohydrate catabolic process | biological\_process | 1.30356840859 | 0.539095188232 | 1.0 | 1 | cluster\_plot | 81 |
| 282 | GO:0016567 | protein ubiquitination | biological\_process | 1.28767123288 | 0.543422586241 | 1.0 | 1 | cluster\_plot | 82 |
| 283 | GO:0070647 | protein modification by small protein conjugation or removal | biological\_process | 1.257012394 | 0.551955466729 | 1.0 | 1 | cluster\_plot | 84 |
| 284 | GO:0032446 | protein modification by small protein conjugation | biological\_process | 1.257012394 | 0.551955466729 | 1.0 | 1 | cluster\_plot | 84 |
| 285 | GO:0006260 | DNA replication | biological\_process | 1.21366713903 | 0.564455724398 | 1.0 | 1 | cluster\_plot | 87 |
| 286 | GO:0016879 | ligase activity, forming carbon-nitrogen bonds | molecular\_function | 1.05589041096 | 0.567081003456 | 1.0 | 2 | cluster\_plot | 200 |
| 287 | GO:0032774 | RNA biosynthetic process | biological\_process | 1.0301369863 | 0.580287450207 | 1.0 | 2 | cluster\_plot | 205 |
| 288 | GO:0051536 | iron-sulfur cluster binding | molecular\_function | 1.16031913292 | 0.580580138262 | 1.0 | 1 | cluster\_plot | 91 |
| 289 | GO:0051540 | metal cluster binding | molecular\_function | 1.16031913292 | 0.580580138262 | 1.0 | 1 | cluster\_plot | 91 |
| 290 | GO:0015711 | organic anion transport | biological\_process | 1.14770696843 | 0.584516775983 | 1.0 | 1 | cluster\_plot | 92 |
| 291 | GO:0009108 | coenzyme biosynthetic process | biological\_process | 1.13536603329 | 0.588416339845 | 1.0 | 1 | cluster\_plot | 93 |
| 292 | GO:0015077 | monovalent inorganic cation transmembrane transporter activity | molecular\_function | 1.01042144589 | 0.590635655076 | 1.0 | 2 | cluster\_plot | 209 |
| 293 | GO:0005507 | copper ion binding | molecular\_function | 1.0056099152 | 0.593192543619 | 1.0 | 2 | cluster\_plot | 210 |
| 294 | GO:0016070 | RNA metabolic process | biological\_process | 0.956422473695 | 0.600281864305 | 1.0 | 5 | cluster\_plot | 552 |
| 295 | GO:0006855 | drug transmembrane transport | biological\_process | 1.08854681542 | 0.603650813585 | 1.0 | 1 | cluster\_plot | 97 |
| 296 | GO:0090484 | drug transporter activity | molecular\_function | 1.08854681542 | 0.603650813585 | 1.0 | 1 | cluster\_plot | 97 |
| 297 | GO:0015893 | drug transport | biological\_process | 1.08854681542 | 0.603650813585 | 1.0 | 1 | cluster\_plot | 97 |
| 298 | GO:0015238 | drug transmembrane transporter activity | molecular\_function | 1.08854681542 | 0.603650813585 | 1.0 | 1 | cluster\_plot | 97 |
| 299 | GO:0003676 | nucleic acid binding | molecular\_function | 0.959521116241 | 0.606170559233 | 1.0 | 23 | cluster\_plot | 2531 |
| 300 | GO:0005575 | cellular\_component | cellular\_component | 0.965127369021 | 0.612576766377 | 1.0 | 47 | cluster\_plot | 5142 |
| 301 | GO:0016881 | acid-amino acid ligase activity | molecular\_function | 1.01527924131 | 0.628962526299 | 1.0 | 1 | cluster\_plot | 104 |
| 302 | GO:0006396 | RNA processing | biological\_process | 0.918165574747 | 0.641804103072 | 1.0 | 2 | cluster\_plot | 230 |
| 303 | GO:0000151 | ubiquitin ligase complex | cellular\_component | 0.977676306443 | 0.642691443187 | 1.0 | 1 | cluster\_plot | 108 |
| 304 | GO:0004553 | hydrolase activity, hydrolyzing O-glycosyl compounds | molecular\_function | 0.907122346185 | 0.645408345531 | 1.0 | 5 | cluster\_plot | 582 |
| 305 | GO:0004842 | ubiquitin-protein transferase activity | molecular\_function | 0.951252622485 | 0.652652616859 | 1.0 | 1 | cluster\_plot | 111 |
| 306 | GO:0016052 | carbohydrate catabolic process | biological\_process | 0.918165574747 | 0.665502127107 | 1.0 | 1 | cluster\_plot | 115 |
| 307 | GO:0044763 | single-organism cellular process | biological\_process | 0.930148159735 | 0.668356689404 | 1.0 | 27 | cluster\_plot | 3065 |
| 308 | GO:0004386 | helicase activity | molecular\_function | 0.865483943409 | 0.673005055461 | 1.0 | 2 | cluster\_plot | 244 |
| 309 | GO:0006732 | coenzyme metabolic process | biological\_process | 0.894822382169 | 0.67482530674 | 1.0 | 1 | cluster\_plot | 118 |
| 310 | GO:0006796 | phosphate-containing compound metabolic process | biological\_process | 0.914816133526 | 0.67857695421 | 1.0 | 19 | cluster\_plot | 2193 |
| 311 | GO:0006793 | phosphorus metabolic process | biological\_process | 0.913982588074 | 0.679984024187 | 1.0 | 19 | cluster\_plot | 2195 |
| 312 | GO:0016798 | hydrolase activity, acting on glycosyl bonds | molecular\_function | 0.85984561153 | 0.689809263295 | 1.0 | 5 | cluster\_plot | 614 |
| 313 | GO:0016788 | hydrolase activity, acting on ester bonds | molecular\_function | 0.858447488584 | 0.697967180013 | 1.0 | 6 | cluster\_plot | 738 |
| 314 | GO:0016043 | cellular component organization | biological\_process | 0.831409772409 | 0.699477853019 | 1.0 | 3 | cluster\_plot | 381 |
| 315 | GO:0030234 | enzyme regulator activity | molecular\_function | 0.818519698418 | 0.701945866194 | 1.0 | 2 | cluster\_plot | 258 |
| 316 | GO:0016887 | ATPase activity | molecular\_function | 0.838008262666 | 0.702139400203 | 1.0 | 4 | cluster\_plot | 504 |
| 317 | GO:0051188 | cofactor biosynthetic process | biological\_process | 0.812223393045 | 0.709582959582 | 1.0 | 1 | cluster\_plot | 130 |
| 318 | GO:0003924 | GTPase activity | molecular\_function | 0.782141045155 | 0.7229414335 | 1.0 | 1 | cluster\_plot | 135 |
| 319 | GO:0071840 | cellular component organization or biogenesis | biological\_process | 0.776390008058 | 0.741536094971 | 1.0 | 3 | cluster\_plot | 408 |
| 320 | GO:0030599 | pectinesterase activity | molecular\_function | 0.723212610246 | 0.750200237499 | 1.0 | 1 | cluster\_plot | 146 |
| 321 | GO:0004857 | enzyme inhibitor activity | molecular\_function | 0.713439466864 | 0.754859184281 | 1.0 | 1 | cluster\_plot | 148 |
| 322 | GO:0008150 | biological\_process | biological\_process | 0.930615277455 | 0.759590485103 | 1.0 | 117 | cluster\_plot | 13275 |
| 323 | GO:0016747 | transferase activity, transferring acyl groups other than amino-acyl groups | molecular\_function | 0.711037313777 | 0.771395104802 | 1.0 | 2 | cluster\_plot | 297 |
| 324 | GO:1901607 | alpha-amino acid biosynthetic process | biological\_process | 0.672541663031 | 0.774768902356 | 1.0 | 1 | cluster\_plot | 157 |
| 325 | GO:0090304 | nucleic acid metabolic process | biological\_process | 0.781176629563 | 0.776519646929 | 1.0 | 6 | cluster\_plot | 811 |
| 326 | GO:0016209 | antioxidant activity | molecular\_function | 0.668285070227 | 0.776878441479 | 1.0 | 1 | cluster\_plot | 158 |
| 327 | GO:0043229 | intracellular organelle | cellular\_component | 0.811599086056 | 0.781906907339 | 1.0 | 10 | cluster\_plot | 1301 |
| 328 | GO:0043226 | organelle | cellular\_component | 0.811599086056 | 0.781906907339 | 1.0 | 10 | cluster\_plot | 1301 |
| 329 | GO:0020037 | heme binding | molecular\_function | 0.74358479645 | 0.783633319504 | 1.0 | 4 | cluster\_plot | 568 |
| 330 | GO:0046906 | tetrapyrrole binding | molecular\_function | 0.74358479645 | 0.783633319504 | 1.0 | 4 | cluster\_plot | 568 |
| 331 | GO:0044267 | cellular protein metabolic process | biological\_process | 0.857496826914 | 0.78438177163 | 1.0 | 22 | cluster\_plot | 2709 |
| 332 | GO:0016874 | ligase activity | molecular\_function | 0.676852827538 | 0.794078444555 | 1.0 | 2 | cluster\_plot | 312 |
| 333 | GO:0051186 | cofactor metabolic process | biological\_process | 0.632269707161 | 0.794994234858 | 1.0 | 1 | cluster\_plot | 167 |
| 334 | GO:0016740 | transferase activity | molecular\_function | 0.861511809043 | 0.811037290374 | 1.0 | 32 | cluster\_plot | 3922 |
| 335 | GO:0044699 | single-organism process | biological\_process | 0.87642290289 | 0.812405503342 | 1.0 | 44 | cluster\_plot | 5301 |
| 336 | GO:0050660 | flavin adenine dinucleotide binding | molecular\_function | 0.58988291115 | 0.816874571502 | 1.0 | 1 | cluster\_plot | 179 |
| 337 | GO:0005975 | carbohydrate metabolic process | biological\_process | 0.746589179466 | 0.823123112127 | 1.0 | 7 | cluster\_plot | 990 |
| 338 | GO:0008168 | methyltransferase activity | molecular\_function | 0.630382334901 | 0.824974529885 | 1.0 | 2 | cluster\_plot | 335 |
| 339 | GO:0005506 | iron ion binding | molecular\_function | 0.664082019471 | 0.827677156295 | 1.0 | 3 | cluster\_plot | 477 |
| 340 | GO:1901575 | organic substance catabolic process | biological\_process | 0.658559507875 | 0.831812450149 | 1.0 | 3 | cluster\_plot | 481 |
| 341 | GO:0009059 | macromolecule biosynthetic process | biological\_process | 0.719925280199 | 0.835501014388 | 1.0 | 6 | cluster\_plot | 880 |
| 342 | GO:0005737 | cytoplasm | cellular\_component | 0.613889773813 | 0.835878192918 | 1.0 | 2 | cluster\_plot | 344 |
| 343 | GO:0016491 | oxidoreductase activity | molecular\_function | 0.784077037841 | 0.851241708211 | 1.0 | 15 | cluster\_plot | 2020 |
| 344 | GO:0034654 | nucleobase-containing compound biosynthetic process | biological\_process | 0.586605783866 | 0.853746220629 | 1.0 | 2 | cluster\_plot | 360 |
| 345 | GO:0044248 | cellular catabolic process | biological\_process | 0.583364867933 | 0.855849737318 | 1.0 | 2 | cluster\_plot | 362 |
| 346 | GO:0016616 | oxidoreductase activity, acting on the CH-OH group of donors, NAD or NADP as acceptor | molecular\_function | 0.507639620653 | 0.860566289388 | 1.0 | 1 | cluster\_plot | 208 |
| 347 | GO:0042623 | ATPase activity, coupled | molecular\_function | 0.572298325723 | 0.862994837682 | 1.0 | 2 | cluster\_plot | 369 |
| 348 | GO:0016758 | transferase activity, transferring hexosyl groups | molecular\_function | 0.613889773813 | 0.864459584852 | 1.0 | 3 | cluster\_plot | 516 |
| 349 | GO:0016741 | transferase activity, transferring one-carbon groups | molecular\_function | 0.567683016645 | 0.86595589087 | 1.0 | 2 | cluster\_plot | 372 |
| 350 | GO:0071702 | organic substance transport | biological\_process | 0.563141552511 | 0.868857741922 | 1.0 | 2 | cluster\_plot | 375 |
| 351 | GO:0032787 | monocarboxylic acid metabolic process | biological\_process | 0.491111819051 | 0.869440167316 | 1.0 | 1 | cluster\_plot | 215 |
| 352 | GO:0008652 | cellular amino acid biosynthetic process | biological\_process | 0.488838153222 | 0.870660741989 | 1.0 | 1 | cluster\_plot | 216 |
| 353 | GO:0009056 | catabolic process | biological\_process | 0.604517410854 | 0.871084473401 | 1.0 | 3 | cluster\_plot | 524 |
| 354 | GO:0044711 | single-organism biosynthetic process | biological\_process | 0.657466009314 | 0.873461555596 | 1.0 | 5 | cluster\_plot | 803 |
| 355 | GO:0016746 | transferase activity, transferring acyl groups | molecular\_function | 0.551378804678 | 0.876314221203 | 1.0 | 2 | cluster\_plot | 383 |
| 356 | GO:0044283 | small molecule biosynthetic process | biological\_process | 0.547093477181 | 0.879007553282 | 1.0 | 2 | cluster\_plot | 386 |
| 357 | GO:1901605 | alpha-amino acid metabolic process | biological\_process | 0.465149960775 | 0.883354610629 | 1.0 | 1 | cluster\_plot | 227 |
| 358 | GO:0016706 | oxidoreductase activity, acting on paired donors, with incorporation or reduction of molecular oxygen, 2-oxoglutarate as one donor, and incorporation of one atom each of oxygen into both donors | molecular\_function | 0.457095416 | 0.887653963082 | 1.0 | 1 | cluster\_plot | 231 |
| 359 | GO:0016853 | isomerase activity | molecular\_function | 0.453171850197 | 0.889743645073 | 1.0 | 1 | cluster\_plot | 233 |
| 360 | GO:0016614 | oxidoreductase activity, acting on CH-OH group of donors | molecular\_function | 0.4399543379 | 0.896755172551 | 1.0 | 1 | cluster\_plot | 240 |
| 361 | GO:0017111 | nucleoside-triphosphatase activity | molecular\_function | 0.62701330817 | 0.896813730797 | 1.0 | 5 | cluster\_plot | 842 |
| 362 | GO:0044260 | cellular macromolecule metabolic process | biological\_process | 0.790418738198 | 0.905437597697 | 1.0 | 29 | cluster\_plot | 3874 |
| 363 | GO:0006629 | lipid metabolic process | biological\_process | 0.548989815057 | 0.908120054183 | 1.0 | 3 | cluster\_plot | 577 |
| 364 | GO:0016462 | pyrophosphatase activity | molecular\_function | 0.608231803548 | 0.910208247251 | 1.0 | 5 | cluster\_plot | 868 |
| 365 | GO:0016705 | oxidoreductase activity, acting on paired donors, with incorporation or reduction of molecular oxygen | molecular\_function | 0.580957585122 | 0.910337927296 | 1.0 | 4 | cluster\_plot | 727 |
| 366 | GO:0016818 | hydrolase activity, acting on acid anhydrides, in phosphorus-containing anhydrides | molecular\_function | 0.596548254779 | 0.9181134334 | 1.0 | 5 | cluster\_plot | 885 |
| 367 | GO:0006259 | DNA metabolic process | biological\_process | 0.398449211683 | 0.918340141421 | 1.0 | 1 | cluster\_plot | 265 |
| 368 | GO:0044237 | cellular metabolic process | biological\_process | 0.801397757299 | 0.922457861591 | 1.0 | 41 | cluster\_plot | 5402 |
| 369 | GO:0016817 | hydrolase activity, acting on acid anhydrides | molecular\_function | 0.585305105853 | 0.925393430508 | 1.0 | 5 | cluster\_plot | 902 |
| 370 | GO:0044710 | single-organism metabolic process | biological\_process | 0.759196441587 | 0.925579332785 | 1.0 | 25 | cluster\_plot | 3477 |
| 371 | GO:0051213 | dioxygenase activity | molecular\_function | 0.38396014944 | 0.925648870452 | 1.0 | 1 | cluster\_plot | 275 |
| 372 | GO:0006725 | cellular aromatic compound metabolic process | biological\_process | 0.637518738692 | 0.928705819585 | 1.0 | 8 | cluster\_plot | 1325 |
| 373 | GO:0009055 | electron carrier activity | molecular\_function | 0.502008119315 | 0.935623005493 | 1.0 | 3 | cluster\_plot | 631 |
| 374 | GO:0016757 | transferase activity, transferring glycosyl groups | molecular\_function | 0.500421995715 | 0.936477417306 | 1.0 | 3 | cluster\_plot | 633 |
| 375 | GO:0019538 | protein metabolic process | biological\_process | 0.735032671067 | 0.940229889976 | 1.0 | 23 | cluster\_plot | 3304 |
| 376 | GO:0006139 | nucleobase-containing compound metabolic process | biological\_process | 0.580159566461 | 0.942434085043 | 1.0 | 6 | cluster\_plot | 1092 |
| 377 | GO:1901360 | organic cyclic compound metabolic process | biological\_process | 0.613889773813 | 0.943863630058 | 1.0 | 8 | cluster\_plot | 1376 |
| 378 | GO:0034645 | cellular macromolecule biosynthetic process | biological\_process | 0.521427363436 | 0.94552171293 | 1.0 | 4 | cluster\_plot | 810 |
| 379 | GO:0043234 | protein complex | cellular\_component | 0.520784419708 | 0.945854938253 | 1.0 | 4 | cluster\_plot | 811 |
| 380 | GO:0008233 | peptidase activity | molecular\_function | 0.428353107894 | 0.945893894418 | 1.0 | 2 | cluster\_plot | 493 |
| 381 | GO:0005622 | intracellular | cellular\_component | 0.417347988521 | 0.95104155652 | 1.0 | 2 | cluster\_plot | 506 |
| 382 | GO:0003682 | chromatin binding | molecular\_function | 0.329965753425 | 0.951224253363 | 1.0 | 1 | cluster\_plot | 320 |
| 383 | GO:0019438 | aromatic compound biosynthetic process | biological\_process | 0.416524816946 | 0.951417438999 | 1.0 | 2 | cluster\_plot | 507 |
| 384 | GO:0005524 | ATP binding | molecular\_function | 0.679848956754 | 0.953152212886 | 1.0 | 16 | cluster\_plot | 2485 |
| 385 | GO:0055114 | oxidation-reduction process | biological\_process | 0.636078560819 | 0.953560339092 | 1.0 | 11 | cluster\_plot | 1826 |
| 386 | GO:0044464 | cell part | cellular\_component | 0.696594368341 | 0.954261672656 | 1.0 | 19 | cluster\_plot | 2880 |
| 387 | GO:0044424 | intracellular part | cellular\_component | 0.677124111236 | 0.954840151354 | 1.0 | 16 | cluster\_plot | 2495 |
| 388 | GO:0046983 | protein dimerization activity | molecular\_function | 0.320939334638 | 0.955164833555 | 1.0 | 1 | cluster\_plot | 329 |
| 389 | GO:0044271 | cellular nitrogen compound biosynthetic process | biological\_process | 0.406894185341 | 0.955717182338 | 1.0 | 2 | cluster\_plot | 519 |
| 390 | GO:0018130 | heterocycle biosynthetic process | biological\_process | 0.404555713011 | 0.956733510079 | 1.0 | 2 | cluster\_plot | 522 |
| 391 | GO:0019637 | organophosphate metabolic process | biological\_process | 0.311472097628 | 0.959169851005 | 1.0 | 1 | cluster\_plot | 339 |
| 392 | GO:0034641 | cellular nitrogen compound metabolic process | biological\_process | 0.567683016645 | 0.959541795183 | 1.0 | 7 | cluster\_plot | 1302 |
| 393 | GO:0046394 | carboxylic acid biosynthetic process | biological\_process | 0.306944886907 | 0.961035696308 | 1.0 | 1 | cluster\_plot | 344 |
| 394 | GO:0016053 | organic acid biosynthetic process | biological\_process | 0.306944886907 | 0.961035696308 | 1.0 | 1 | cluster\_plot | 344 |
| 395 | GO:0005525 | GTP binding | molecular\_function | 0.305170639005 | 0.961757858029 | 1.0 | 1 | cluster\_plot | 346 |
| 396 | GO:0032561 | guanyl ribonucleotide binding | molecular\_function | 0.305170639005 | 0.961757858029 | 1.0 | 1 | cluster\_plot | 346 |
| 397 | GO:0043170 | macromolecule metabolic process | biological\_process | 0.727030349524 | 0.963997571173 | 1.0 | 30 | cluster\_plot | 4357 |
| 398 | GO:0019001 | guanyl nucleotide binding | molecular\_function | 0.29827412739 | 0.964514694009 | 1.0 | 1 | cluster\_plot | 354 |
| 399 | GO:0006508 | proteolysis | biological\_process | 0.38396014944 | 0.965195960185 | 1.0 | 2 | cluster\_plot | 550 |
| 400 | GO:1901362 | organic cyclic compound biosynthetic process | biological\_process | 0.37113898452 | 0.970001935469 | 1.0 | 2 | cluster\_plot | 569 |
| 401 | GO:0016787 | hydrolase activity | molecular\_function | 0.652844705767 | 0.976917692691 | 1.0 | 19 | cluster\_plot | 3073 |
| 402 | GO:0035639 | purine ribonucleoside triphosphate binding | molecular\_function | 0.634056410678 | 0.979072185941 | 1.0 | 17 | cluster\_plot | 2831 |
| 403 | GO:0046483 | heterocycle metabolic process | biological\_process | 0.492639383029 | 0.980298711907 | 1.0 | 6 | cluster\_plot | 1286 |
| 404 | GO:0050662 | coenzyme binding | molecular\_function | 0.253211129726 | 0.98030070996 | 1.0 | 1 | cluster\_plot | 417 |
| 405 | GO:0006520 | cellular amino acid metabolic process | biological\_process | 0.247861598817 | 0.981887497774 | 1.0 | 1 | cluster\_plot | 426 |
| 406 | GO:0044249 | cellular biosynthetic process | biological\_process | 0.526300516366 | 0.982173742642 | 1.0 | 8 | cluster\_plot | 1605 |
| 407 | GO:0006807 | nitrogen compound metabolic process | biological\_process | 0.520143059586 | 0.983869820365 | 1.0 | 8 | cluster\_plot | 1624 |
| 408 | GO:1901576 | organic substance biosynthetic process | biological\_process | 0.514441125924 | 0.985336464875 | 1.0 | 8 | cluster\_plot | 1642 |
| 409 | GO:0044281 | small molecule metabolic process | biological\_process | 0.415704886204 | 0.985423410122 | 1.0 | 4 | cluster\_plot | 1016 |
| 410 | GO:0046914 | transition metal ion binding | molecular\_function | 0.508250498657 | 0.98681917828 | 1.0 | 8 | cluster\_plot | 1662 |
| 411 | GO:0019752 | carboxylic acid metabolic process | biological\_process | 0.313786154817 | 0.986846703664 | 1.0 | 2 | cluster\_plot | 673 |
| 412 | GO:0043436 | oxoacid metabolic process | biological\_process | 0.313786154817 | 0.986846703664 | 1.0 | 2 | cluster\_plot | 673 |
| 413 | GO:0006082 | organic acid metabolic process | biological\_process | 0.313320596724 | 0.986951599451 | 1.0 | 2 | cluster\_plot | 674 |
| 414 | GO:1901566 | organonitrogen compound biosynthetic process | biological\_process | 0.226585925098 | 0.987525978393 | 1.0 | 1 | cluster\_plot | 466 |
| 415 | GO:0046872 | metal ion binding | molecular\_function | 0.568623667873 | 0.988198279186 | 1.0 | 13 | cluster\_plot | 2414 |
| 416 | GO:0043169 | cation binding | molecular\_function | 0.563951328778 | 0.989221365003 | 1.0 | 13 | cluster\_plot | 2434 |
| 417 | GO:0030529 | ribonucleoprotein complex | cellular\_component | 0.21861085113 | 0.9893529132 | 1.0 | 1 | cluster\_plot | 483 |
| 418 | GO:0071704 | organic substance metabolic process | biological\_process | 0.687876489224 | 0.992391318294 | 1.0 | 42 | cluster\_plot | 6447 |
| 419 | GO:0048037 | cofactor binding | molecular\_function | 0.19884941826 | 0.993188527733 | 1.0 | 1 | cluster\_plot | 531 |
| 420 | GO:0032991 | macromolecular complex | cellular\_component | 0.405799543028 | 0.99322058969 | 1.0 | 5 | cluster\_plot | 1301 |
| 421 | GO:0008270 | zinc ion binding | molecular\_function | 0.333088457716 | 0.993291994321 | 1.0 | 3 | cluster\_plot | 951 |
| 422 | GO:0008152 | metabolic process | biological\_process | 0.718923846688 | 0.993598522182 | 1.0 | 62 | cluster\_plot | 9106 |
| 423 | GO:0003824 | catalytic activity | molecular\_function | 0.728631001253 | 0.993764300019 | 1.0 | 70 | cluster\_plot | 10144 |
| 424 | GO:0009058 | biosynthetic process | biological\_process | 0.457095416 | 0.995260955332 | 1.0 | 8 | cluster\_plot | 1848 |
| 425 | GO:0044238 | primary metabolic process | biological\_process | 0.656261622775 | 0.995744691394 | 1.0 | 38 | cluster\_plot | 6114 |
| 426 | GO:1901564 | organonitrogen compound metabolic process | biological\_process | 0.253515104672 | 0.996400187677 | 1.0 | 2 | cluster\_plot | 833 |
| 427 | GO:0010563 | negative regulation of phosphorus metabolic process | biological\_process | 0.0 | 1.0 | 1.0 | 0 | cluster\_plot | 3 |
| 428 | GO:0003755 | peptidyl-prolyl cis-trans isomerase activity | molecular\_function | 0.0 | 1.0 | 1.0 | 0 | cluster\_plot | 48 |
| 429 | GO:0004888 | transmembrane signaling receptor activity | molecular\_function | 0.0 | 1.0 | 1.0 | 0 | cluster\_plot | 41 |
| 430 | GO:0006426 | glycyl-tRNA aminoacylation | biological\_process | 0.0 | 1.0 | 1.0 | 0 | cluster\_plot | 3 |
| 431 | GO:0004526 | ribonuclease P activity | molecular\_function | 0.0 | 1.0 | 1.0 | 0 | cluster\_plot | 2 |
| 432 | GO:0009203 | ribonucleoside triphosphate catabolic process | biological\_process | 0.0 | 1.0 | 1.0 | 0 | cluster\_plot | 41 |
| 433 | GO:0017007 | protein-bilin linkage | biological\_process | 0.0 | 1.0 | 1.0 | 0 | cluster\_plot | 2 |
| 434 | GO:0006740 | NADPH regeneration | biological\_process | 0.0 | 1.0 | 1.0 | 0 | cluster\_plot | 15 |
| 435 | GO:0006890 | retrograde vesicle-mediated transport, Golgi to ER | biological\_process | 0.0 | 1.0 | 1.0 | 0 | cluster\_plot | 2 |
| 436 | GO:0004385 | guanylate kinase activity | molecular\_function | 0.0 | 1.0 | 1.0 | 0 | cluster\_plot | 6 |
| 437 | GO:0004371 | glycerone kinase activity | molecular\_function | 0.0 | 1.0 | 1.0 | 0 | cluster\_plot | 3 |
| 438 | GO:0000989 | transcription factor binding transcription factor activity | molecular\_function | 0.0 | 1.0 | 1.0 | 0 | cluster\_plot | 37 |
| 439 | GO:0005945 | 6-phosphofructokinase complex | cellular\_component | 0.0 | 1.0 | 1.0 | 0 | cluster\_plot | 15 |
| 440 | GO:0043244 | regulation of protein complex disassembly | biological\_process | 0.0 | 1.0 | 1.0 | 0 | cluster\_plot | 3 |
| 441 | GO:0044822 | poly(A) RNA binding | molecular\_function | 0.0 | 1.0 | 1.0 | 0 | cluster\_plot | 3 |
| 442 | GO:0051275 | beta-glucan catabolic process | biological\_process | 0.0 | 1.0 | 1.0 | 0 | cluster\_plot | 3 |
| 443 | GO:0015926 | glucosidase activity | molecular\_function | 0.0 | 1.0 | 1.0 | 0 | cluster\_plot | 13 |
| 444 | GO:0017148 | negative regulation of translation | biological\_process | 0.0 | 1.0 | 1.0 | 0 | cluster\_plot | 10 |
| 445 | GO:0004450 | isocitrate dehydrogenase (NADP+) activity | molecular\_function | 0.0 | 1.0 | 1.0 | 0 | cluster\_plot | 7 |
| 446 | GO:0016814 | hydrolase activity, acting on carbon-nitrogen (but not peptide) bonds, in cyclic amidines | molecular\_function | 0.0 | 1.0 | 1.0 | 0 | cluster\_plot | 20 |
| 447 | GO:0030176 | integral component of endoplasmic reticulum membrane | cellular\_component | 0.0 | 1.0 | 1.0 | 0 | cluster\_plot | 2 |
| 448 | GO:0042254 | ribosome biogenesis | biological\_process | 0.0 | 1.0 | 1.0 | 0 | cluster\_plot | 25 |
| 449 | GO:0023051 | regulation of signaling | biological\_process | 0.0 | 1.0 | 1.0 | 0 | cluster\_plot | 18 |
| 450 | GO:0033764 | steroid dehydrogenase activity, acting on the CH-OH group of donors, NAD or NADP as acceptor | molecular\_function | 0.0 | 1.0 | 1.0 | 0 | cluster\_plot | 15 |
| 451 | GO:0005666 | DNA-directed RNA polymerase III complex | cellular\_component | 0.0 | 1.0 | 1.0 | 0 | cluster\_plot | 6 |
| 452 | GO:2001020 | regulation of response to DNA damage stimulus | biological\_process | 0.0 | 1.0 | 1.0 | 0 | cluster\_plot | 2 |
| 453 | GO:0009209 | pyrimidine ribonucleoside triphosphate biosynthetic process | biological\_process | 0.0 | 1.0 | 1.0 | 0 | cluster\_plot | 7 |
| 454 | GO:0016429 | tRNA (adenine-N1-)-methyltransferase activity | molecular\_function | 0.0 | 1.0 | 1.0 | 0 | cluster\_plot | 3 |
| 455 | GO:0031248 | protein acetyltransferase complex | cellular\_component | 0.0 | 1.0 | 1.0 | 0 | cluster\_plot | 14 |
| 456 | GO:0045841 | negative regulation of mitotic metaphase/anaphase transition | biological\_process | 0.0 | 1.0 | 1.0 | 0 | cluster\_plot | 2 |
| 457 | GO:0006691 | leukotriene metabolic process | biological\_process | 0.0 | 1.0 | 1.0 | 0 | cluster\_plot | 3 |
| 458 | GO:0044433 | cytoplasmic vesicle part | cellular\_component | 0.0 | 1.0 | 1.0 | 0 | cluster\_plot | 42 |
| 459 | GO:0042445 | hormone metabolic process | biological\_process | 0.0 | 1.0 | 1.0 | 0 | cluster\_plot | 13 |
| 460 | GO:0043043 | peptide biosynthetic process | biological\_process | 0.0 | 1.0 | 1.0 | 0 | cluster\_plot | 13 |
| 461 | GO:0051169 | nuclear transport | biological\_process | 0.0 | 1.0 | 1.0 | 0 | cluster\_plot | 9 |
| 462 | GO:0005986 | sucrose biosynthetic process | biological\_process | 0.0 | 1.0 | 1.0 | 0 | cluster\_plot | 6 |
| 463 | GO:0017119 | Golgi transport complex | cellular\_component | 0.0 | 1.0 | 1.0 | 0 | cluster\_plot | 9 |
| 464 | GO:0008083 | growth factor activity | molecular\_function | 0.0 | 1.0 | 1.0 | 0 | cluster\_plot | 11 |
| 465 | GO:0004564 | beta-fructofuranosidase activity | molecular\_function | 0.0 | 1.0 | 1.0 | 0 | cluster\_plot | 8 |
| 466 | GO:0005672 | transcription factor TFIIA complex | cellular\_component | 0.0 | 1.0 | 1.0 | 0 | cluster\_plot | 2 |
| 467 | GO:0006418 | tRNA aminoacylation for protein translation | biological\_process | 0.0 | 1.0 | 1.0 | 0 | cluster\_plot | 84 |
| 468 | GO:0016272 | prefoldin complex | cellular\_component | 0.0 | 1.0 | 1.0 | 0 | cluster\_plot | 15 |
| 469 | GO:0008677 | 2-dehydropantoate 2-reductase activity | molecular\_function | 0.0 | 1.0 | 1.0 | 0 | cluster\_plot | 1 |
| 470 | GO:0000295 | adenine nucleotide transmembrane transporter activity | molecular\_function | 0.0 | 1.0 | 1.0 | 0 | cluster\_plot | 1 |
| 471 | GO:0004348 | glucosylceramidase activity | molecular\_function | 0.0 | 1.0 | 1.0 | 0 | cluster\_plot | 5 |
| 472 | GO:0006338 | chromatin remodeling | biological\_process | 0.0 | 1.0 | 1.0 | 0 | cluster\_plot | 5 |
| 473 | GO:0005092 | GDP-dissociation inhibitor activity | molecular\_function | 0.0 | 1.0 | 1.0 | 0 | cluster\_plot | 6 |
| 474 | GO:0046406 | magnesium protoporphyrin IX methyltransferase activity | molecular\_function | 0.0 | 1.0 | 1.0 | 0 | cluster\_plot | 3 |
| 475 | GO:0019307 | mannose biosynthetic process | biological\_process | 0.0 | 1.0 | 1.0 | 0 | cluster\_plot | 1 |
| 476 | GO:0015743 | malate transport | biological\_process | 0.0 | 1.0 | 1.0 | 0 | cluster\_plot | 30 |
| 477 | GO:0005849 | mRNA cleavage factor complex | cellular\_component | 0.0 | 1.0 | 1.0 | 0 | cluster\_plot | 3 |
| 478 | GO:0000347 | THO complex | cellular\_component | 0.0 | 1.0 | 1.0 | 0 | cluster\_plot | 4 |
| 479 | GO:0006021 | inositol biosynthetic process | biological\_process | 0.0 | 1.0 | 1.0 | 0 | cluster\_plot | 6 |
| 480 | GO:0004089 | carbonate dehydratase activity | molecular\_function | 0.0 | 1.0 | 1.0 | 0 | cluster\_plot | 10 |
| 481 | GO:0005253 | anion channel activity | molecular\_function | 0.0 | 1.0 | 1.0 | 0 | cluster\_plot | 13 |
| 482 | GO:0009527 | plastid outer membrane | cellular\_component | 0.0 | 1.0 | 1.0 | 0 | cluster\_plot | 2 |
| 483 | GO:0044437 | vacuolar part | cellular\_component | 0.0 | 1.0 | 1.0 | 0 | cluster\_plot | 8 |
| 484 | GO:0016765 | transferase activity, transferring alkyl or aryl (other than methyl) groups | molecular\_function | 0.0 | 1.0 | 1.0 | 0 | cluster\_plot | 58 |
| 485 | GO:1901606 | alpha-amino acid catabolic process | biological\_process | 0.0 | 1.0 | 1.0 | 0 | cluster\_plot | 27 |
| 486 | GO:0005674 | transcription factor TFIIF complex | cellular\_component | 0.0 | 1.0 | 1.0 | 0 | cluster\_plot | 4 |
| 487 | GO:0004451 | isocitrate lyase activity | molecular\_function | 0.0 | 1.0 | 1.0 | 0 | cluster\_plot | 2 |
| 488 | GO:0030117 | membrane coat | cellular\_component | 0.0 | 1.0 | 1.0 | 0 | cluster\_plot | 61 |
| 489 | GO:0042364 | water-soluble vitamin biosynthetic process | biological\_process | 0.0 | 1.0 | 1.0 | 0 | cluster\_plot | 32 |
| 490 | GO:0003774 | motor activity | molecular\_function | 0.0 | 1.0 | 1.0 | 0 | cluster\_plot | 117 |
| 491 | GO:0009072 | aromatic amino acid family metabolic process | biological\_process | 0.0 | 1.0 | 1.0 | 0 | cluster\_plot | 53 |
| 492 | GO:0043087 | regulation of GTPase activity | biological\_process | 0.0 | 1.0 | 1.0 | 0 | cluster\_plot | 51 |
| 493 | GO:0031167 | rRNA methylation | biological\_process | 0.0 | 1.0 | 1.0 | 0 | cluster\_plot | 1 |
| 494 | GO:0006428 | isoleucyl-tRNA aminoacylation | biological\_process | 0.0 | 1.0 | 1.0 | 0 | cluster\_plot | 4 |
| 495 | GO:0003830 | beta-1,4-mannosylglycoprotein 4-beta-N-acetylglucosaminyltransferase activity | molecular\_function | 0.0 | 1.0 | 1.0 | 0 | cluster\_plot | 3 |
| 496 | GO:0033014 | tetrapyrrole biosynthetic process | biological\_process | 0.0 | 1.0 | 1.0 | 0 | cluster\_plot | 48 |
| 497 | GO:0009446 | putrescine biosynthetic process | biological\_process | 0.0 | 1.0 | 1.0 | 0 | cluster\_plot | 2 |
| 498 | GO:0051903 | S-(hydroxymethyl)glutathione dehydrogenase activity | molecular\_function | 0.0 | 1.0 | 1.0 | 0 | cluster\_plot | 1 |
| 499 | GO:0003854 | 3-beta-hydroxy-delta5-steroid dehydrogenase activity | molecular\_function | 0.0 | 1.0 | 1.0 | 0 | cluster\_plot | 15 |
| 500 | GO:0007154 | cell communication | biological\_process | 0.0 | 1.0 | 1.0 | 0 | cluster\_plot | 20 |
| 501 | GO:0009152 | purine ribonucleotide biosynthetic process | biological\_process | 0.0 | 1.0 | 1.0 | 0 | cluster\_plot | 77 |
| 502 | GO:0005730 | nucleolus | cellular\_component | 0.0 | 1.0 | 1.0 | 0 | cluster\_plot | 9 |
| 503 | GO:1901292 | nucleoside phosphate catabolic process | biological\_process | 0.0 | 1.0 | 1.0 | 0 | cluster\_plot | 41 |
| 504 | GO:0070011 | peptidase activity, acting on L-amino acid peptides | molecular\_function | 0.0 | 1.0 | 1.0 | 0 | cluster\_plot | 472 |
| 505 | GO:0019905 | syntaxin binding | molecular\_function | 0.0 | 1.0 | 1.0 | 0 | cluster\_plot | 2 |
| 506 | GO:0006526 | arginine biosynthetic process | biological\_process | 0.0 | 1.0 | 1.0 | 0 | cluster\_plot | 16 |
| 507 | GO:0004474 | malate synthase activity | molecular\_function | 0.0 | 1.0 | 1.0 | 0 | cluster\_plot | 3 |
| 508 | GO:0000176 | nuclear exosome (RNase complex) | cellular\_component | 0.0 | 1.0 | 1.0 | 0 | cluster\_plot | 2 |
| 509 | GO:0000439 | core TFIIH complex | cellular\_component | 0.0 | 1.0 | 1.0 | 0 | cluster\_plot | 6 |
| 510 | GO:0032970 | regulation of actin filament-based process | biological\_process | 0.0 | 1.0 | 1.0 | 0 | cluster\_plot | 10 |
| 511 | GO:0004038 | allantoinase activity | molecular\_function | 0.0 | 1.0 | 1.0 | 0 | cluster\_plot | 2 |
| 512 | GO:1903047 | mitotic cell cycle process | biological\_process | 0.0 | 1.0 | 1.0 | 0 | cluster\_plot | 10 |
| 513 | GO:0032273 | positive regulation of protein polymerization | biological\_process | 0.0 | 1.0 | 1.0 | 0 | cluster\_plot | 4 |
| 514 | GO:0003880 | protein C-terminal carboxyl O-methyltransferase activity | molecular\_function | 0.0 | 1.0 | 1.0 | 0 | cluster\_plot | 1 |
| 515 | GO:0030976 | thiamine pyrophosphate binding | molecular\_function | 0.0 | 1.0 | 1.0 | 0 | cluster\_plot | 17 |
| 516 | GO:0019210 | kinase inhibitor activity | molecular\_function | 0.0 | 1.0 | 1.0 | 0 | cluster\_plot | 8 |
| 517 | GO:0052880 | oxidoreductase activity, acting on diphenols and related substances as donors, with copper protein as acceptor | molecular\_function | 0.0 | 1.0 | 1.0 | 0 | cluster\_plot | 2 |
| 518 | GO:0004096 | catalase activity | molecular\_function | 0.0 | 1.0 | 1.0 | 0 | cluster\_plot | 4 |
| 519 | GO:0000723 | telomere maintenance | biological\_process | 0.0 | 1.0 | 1.0 | 0 | cluster\_plot | 3 |
| 520 | GO:0006270 | DNA replication initiation | biological\_process | 0.0 | 1.0 | 1.0 | 0 | cluster\_plot | 5 |
| 521 | GO:0006221 | pyrimidine nucleotide biosynthetic process | biological\_process | 0.0 | 1.0 | 1.0 | 0 | cluster\_plot | 27 |
| 522 | GO:0090575 | RNA polymerase II transcription factor complex | cellular\_component | 0.0 | 1.0 | 1.0 | 0 | cluster\_plot | 15 |
| 523 | GO:0008238 | exopeptidase activity | molecular\_function | 0.0 | 1.0 | 1.0 | 0 | cluster\_plot | 68 |
| 524 | GO:0006848 | pyruvate transport | biological\_process | 0.0 | 1.0 | 1.0 | 0 | cluster\_plot | 6 |
| 525 | GO:0046373 | L-arabinose metabolic process | biological\_process | 0.0 | 1.0 | 1.0 | 0 | cluster\_plot | 1 |
| 526 | GO:0004828 | serine-tRNA ligase activity | molecular\_function | 0.0 | 1.0 | 1.0 | 0 | cluster\_plot | 3 |
| 527 | GO:0072521 | purine-containing compound metabolic process | biological\_process | 0.0 | 1.0 | 1.0 | 0 | cluster\_plot | 160 |
| 528 | GO:0003935 | GTP cyclohydrolase II activity | molecular\_function | 0.0 | 1.0 | 1.0 | 0 | cluster\_plot | 4 |
| 529 | GO:0034040 | lipid-transporting ATPase activity | molecular\_function | 0.0 | 1.0 | 1.0 | 0 | cluster\_plot | 1 |
| 530 | GO:0009143 | nucleoside triphosphate catabolic process | biological\_process | 0.0 | 1.0 | 1.0 | 0 | cluster\_plot | 41 |
| 531 | GO:0008534 | oxidized purine nucleobase lesion DNA N-glycosylase activity | molecular\_function | 0.0 | 1.0 | 1.0 | 0 | cluster\_plot | 3 |
| 532 | GO:0042559 | pteridine-containing compound biosynthetic process | biological\_process | 0.0 | 1.0 | 1.0 | 0 | cluster\_plot | 22 |
| 533 | GO:0007006 | mitochondrial membrane organization | biological\_process | 0.0 | 1.0 | 1.0 | 0 | cluster\_plot | 10 |
| 534 | GO:0030150 | protein import into mitochondrial matrix | biological\_process | 0.0 | 1.0 | 1.0 | 0 | cluster\_plot | 3 |
| 535 | GO:0015295 | solute:proton symporter activity | molecular\_function | 0.0 | 1.0 | 1.0 | 0 | cluster\_plot | 12 |
| 536 | GO:0008192 | RNA guanylyltransferase activity | molecular\_function | 0.0 | 1.0 | 1.0 | 0 | cluster\_plot | 4 |
| 537 | GO:0044453 | nuclear membrane part | cellular\_component | 0.0 | 1.0 | 1.0 | 0 | cluster\_plot | 1 |
| 538 | GO:0017150 | tRNA dihydrouridine synthase activity | molecular\_function | 0.0 | 1.0 | 1.0 | 0 | cluster\_plot | 6 |
| 539 | GO:0009116 | nucleoside metabolic process | biological\_process | 0.0 | 1.0 | 1.0 | 0 | cluster\_plot | 145 |
| 540 | GO:0016192 | vesicle-mediated transport | biological\_process | 0.0 | 1.0 | 1.0 | 0 | cluster\_plot | 202 |
| 541 | GO:0022604 | regulation of cell morphogenesis | biological\_process | 0.0 | 1.0 | 1.0 | 0 | cluster\_plot | 5 |
| 542 | GO:0045935 | positive regulation of nucleobase-containing compound metabolic process | biological\_process | 0.0 | 1.0 | 1.0 | 0 | cluster\_plot | 4 |
| 543 | GO:0046578 | regulation of Ras protein signal transduction | biological\_process | 0.0 | 1.0 | 1.0 | 0 | cluster\_plot | 11 |
| 544 | GO:0004047 | aminomethyltransferase activity | molecular\_function | 0.0 | 1.0 | 1.0 | 0 | cluster\_plot | 4 |
| 545 | GO:0050515 | 4-(cytidine 5'-diphospho)-2-C-methyl-D-erythritol kinase activity | molecular\_function | 0.0 | 1.0 | 1.0 | 0 | cluster\_plot | 1 |
| 546 | GO:0004579 | dolichyl-diphosphooligosaccharide-protein glycotransferase activity | molecular\_function | 0.0 | 1.0 | 1.0 | 0 | cluster\_plot | 10 |
| 547 | GO:0015020 | glucuronosyltransferase activity | molecular\_function | 0.0 | 1.0 | 1.0 | 0 | cluster\_plot | 6 |
| 548 | GO:0004252 | serine-type endopeptidase activity | molecular\_function | 0.0 | 1.0 | 1.0 | 0 | cluster\_plot | 140 |
| 549 | GO:0006022 | aminoglycan metabolic process | biological\_process | 0.0 | 1.0 | 1.0 | 0 | cluster\_plot | 18 |
| 550 | GO:0006914 | autophagy | biological\_process | 0.0 | 1.0 | 1.0 | 0 | cluster\_plot | 5 |
| 551 | GO:0030597 | RNA glycosylase activity | molecular\_function | 0.0 | 1.0 | 1.0 | 0 | cluster\_plot | 8 |
| 552 | GO:0006106 | fumarate metabolic process | biological\_process | 0.0 | 1.0 | 1.0 | 0 | cluster\_plot | 1 |
| 553 | GO:0015369 | calcium:proton antiporter activity | molecular\_function | 0.0 | 1.0 | 1.0 | 0 | cluster\_plot | 12 |
| 554 | GO:0005785 | signal recognition particle receptor complex | cellular\_component | 0.0 | 1.0 | 1.0 | 0 | cluster\_plot | 3 |
| 555 | GO:0008158 | hedgehog receptor activity | molecular\_function | 0.0 | 1.0 | 1.0 | 0 | cluster\_plot | 4 |
| 556 | GO:0050308 | sugar-phosphatase activity | molecular\_function | 0.0 | 1.0 | 1.0 | 0 | cluster\_plot | 17 |
| 557 | GO:0032535 | regulation of cellular component size | biological\_process | 0.0 | 1.0 | 1.0 | 0 | cluster\_plot | 10 |
| 558 | GO:0009317 | acetyl-CoA carboxylase complex | cellular\_component | 0.0 | 1.0 | 1.0 | 0 | cluster\_plot | 4 |
| 559 | GO:0045300 | acyl-[acyl-carrier-protein] desaturase activity | molecular\_function | 0.0 | 1.0 | 1.0 | 0 | cluster\_plot | 5 |
| 560 | GO:0006778 | porphyrin-containing compound metabolic process | biological\_process | 0.0 | 1.0 | 1.0 | 0 | cluster\_plot | 45 |
| 561 | GO:0016722 | oxidoreductase activity, oxidizing metal ions | molecular\_function | 0.0 | 1.0 | 1.0 | 0 | cluster\_plot | 1 |
| 562 | GO:0004668 | protein-arginine deiminase activity | molecular\_function | 0.0 | 1.0 | 1.0 | 0 | cluster\_plot | 2 |
| 563 | GO:0043022 | ribosome binding | molecular\_function | 0.0 | 1.0 | 1.0 | 0 | cluster\_plot | 6 |
| 564 | GO:0000221 | vacuolar proton-transporting V-type ATPase, V1 domain | cellular\_component | 0.0 | 1.0 | 1.0 | 0 | cluster\_plot | 1 |
| 565 | GO:0046939 | nucleotide phosphorylation | biological\_process | 0.0 | 1.0 | 1.0 | 0 | cluster\_plot | 25 |
| 566 | GO:0030130 | clathrin coat of trans-Golgi network vesicle | cellular\_component | 0.0 | 1.0 | 1.0 | 0 | cluster\_plot | 10 |
| 567 | GO:0044389 | small conjugating protein ligase binding | molecular\_function | 0.0 | 1.0 | 1.0 | 0 | cluster\_plot | 19 |
| 568 | GO:0015370 | solute:sodium symporter activity | molecular\_function | 0.0 | 1.0 | 1.0 | 0 | cluster\_plot | 11 |
| 569 | GO:0009102 | biotin biosynthetic process | biological\_process | 0.0 | 1.0 | 1.0 | 0 | cluster\_plot | 2 |
| 570 | GO:0034655 | nucleobase-containing compound catabolic process | biological\_process | 0.0 | 1.0 | 1.0 | 0 | cluster\_plot | 51 |
| 571 | GO:0005639 | integral component of nuclear inner membrane | cellular\_component | 0.0 | 1.0 | 1.0 | 0 | cluster\_plot | 1 |
| 572 | GO:1902600 | hydrogen ion transmembrane transport | biological\_process | 0.0 | 1.0 | 1.0 | 0 | cluster\_plot | 66 |
| 573 | GO:0006635 | fatty acid beta-oxidation | biological\_process | 0.0 | 1.0 | 1.0 | 0 | cluster\_plot | 7 |
| 574 | GO:0005669 | transcription factor TFIID complex | cellular\_component | 0.0 | 1.0 | 1.0 | 0 | cluster\_plot | 9 |
| 575 | GO:0006875 | cellular metal ion homeostasis | biological\_process | 0.0 | 1.0 | 1.0 | 0 | cluster\_plot | 6 |
| 576 | GO:0004527 | exonuclease activity | molecular\_function | 0.0 | 1.0 | 1.0 | 0 | cluster\_plot | 23 |
| 577 | GO:0044728 | DNA methylation or demethylation | biological\_process | 0.0 | 1.0 | 1.0 | 0 | cluster\_plot | 15 |
| 578 | GO:0031491 | nucleosome binding | molecular\_function | 0.0 | 1.0 | 1.0 | 0 | cluster\_plot | 3 |
| 579 | GO:0046034 | ATP metabolic process | biological\_process | 0.0 | 1.0 | 1.0 | 0 | cluster\_plot | 57 |
| 580 | GO:0006275 | regulation of DNA replication | biological\_process | 0.0 | 1.0 | 1.0 | 0 | cluster\_plot | 6 |
| 581 | GO:0004140 | dephospho-CoA kinase activity | molecular\_function | 0.0 | 1.0 | 1.0 | 0 | cluster\_plot | 1 |
| 582 | GO:1901476 | carbohydrate transporter activity | molecular\_function | 0.0 | 1.0 | 1.0 | 0 | cluster\_plot | 18 |
| 583 | GO:0006897 | endocytosis | biological\_process | 0.0 | 1.0 | 1.0 | 0 | cluster\_plot | 10 |
| 584 | GO:0009405 | pathogenesis | biological\_process | 0.0 | 1.0 | 1.0 | 0 | cluster\_plot | 1 |
| 585 | GO:0070408 | carbamoyl phosphate metabolic process | biological\_process | 0.0 | 1.0 | 1.0 | 0 | cluster\_plot | 3 |
| 586 | GO:0051053 | negative regulation of DNA metabolic process | biological\_process | 0.0 | 1.0 | 1.0 | 0 | cluster\_plot | 2 |
| 587 | GO:0009225 | nucleotide-sugar metabolic process | biological\_process | 0.0 | 1.0 | 1.0 | 0 | cluster\_plot | 3 |
| 588 | GO:0003779 | actin binding | molecular\_function | 0.0 | 1.0 | 1.0 | 0 | cluster\_plot | 34 |
| 589 | GO:0009435 | NAD biosynthetic process | biological\_process | 0.0 | 1.0 | 1.0 | 0 | cluster\_plot | 10 |
| 590 | GO:0015996 | chlorophyll catabolic process | biological\_process | 0.0 | 1.0 | 1.0 | 0 | cluster\_plot | 7 |
| 591 | GO:0019206 | nucleoside kinase activity | molecular\_function | 0.0 | 1.0 | 1.0 | 0 | cluster\_plot | 8 |
| 592 | GO:0034754 | cellular hormone metabolic process | biological\_process | 0.0 | 1.0 | 1.0 | 0 | cluster\_plot | 13 |
| 593 | GO:0034062 | RNA polymerase activity | molecular\_function | 0.0 | 1.0 | 1.0 | 0 | cluster\_plot | 94 |
| 594 | GO:0046083 | adenine metabolic process | biological\_process | 0.0 | 1.0 | 1.0 | 0 | cluster\_plot | 8 |
| 595 | GO:0008242 | omega peptidase activity | molecular\_function | 0.0 | 1.0 | 1.0 | 0 | cluster\_plot | 4 |
| 596 | GO:0004499 | N,N-dimethylaniline monooxygenase activity | molecular\_function | 0.0 | 1.0 | 1.0 | 0 | cluster\_plot | 34 |
| 597 | GO:0048284 | organelle fusion | biological\_process | 0.0 | 1.0 | 1.0 | 0 | cluster\_plot | 2 |
| 598 | GO:0004558 | alpha-glucosidase activity | molecular\_function | 0.0 | 1.0 | 1.0 | 0 | cluster\_plot | 8 |
| 599 | GO:0019363 | pyridine nucleotide biosynthetic process | biological\_process | 0.0 | 1.0 | 1.0 | 0 | cluster\_plot | 16 |
| 600 | GO:0036260 | RNA capping | biological\_process | 0.0 | 1.0 | 1.0 | 0 | cluster\_plot | 7 |
| 601 | GO:0007131 | reciprocal meiotic recombination | biological\_process | 0.0 | 1.0 | 1.0 | 0 | cluster\_plot | 1 |
| 602 | GO:0004399 | histidinol dehydrogenase activity | molecular\_function | 0.0 | 1.0 | 1.0 | 0 | cluster\_plot | 1 |
| 603 | GO:0016180 | snRNA processing | biological\_process | 0.0 | 1.0 | 1.0 | 0 | cluster\_plot | 1 |
| 604 | GO:0030329 | prenylcysteine metabolic process | biological\_process | 0.0 | 1.0 | 1.0 | 0 | cluster\_plot | 1 |
| 605 | GO:0004970 | ionotropic glutamate receptor activity | molecular\_function | 0.0 | 1.0 | 1.0 | 0 | cluster\_plot | 37 |
| 606 | GO:0009144 | purine nucleoside triphosphate metabolic process | biological\_process | 0.0 | 1.0 | 1.0 | 0 | cluster\_plot | 86 |
| 607 | GO:0006310 | DNA recombination | biological\_process | 0.0 | 1.0 | 1.0 | 0 | cluster\_plot | 21 |
| 608 | GO:0004357 | glutamate-cysteine ligase activity | molecular\_function | 0.0 | 1.0 | 1.0 | 0 | cluster\_plot | 4 |
| 609 | GO:1901135 | carbohydrate derivative metabolic process | biological\_process | 0.0 | 1.0 | 1.0 | 0 | cluster\_plot | 234 |
| 610 | GO:0044108 | cellular alcohol biosynthetic process | biological\_process | 0.0 | 1.0 | 1.0 | 0 | cluster\_plot | 3 |
| 611 | GO:0032266 | phosphatidylinositol-3-phosphate binding | molecular\_function | 0.0 | 1.0 | 1.0 | 0 | cluster\_plot | 1 |
| 612 | GO:0006564 | L-serine biosynthetic process | biological\_process | 0.0 | 1.0 | 1.0 | 0 | cluster\_plot | 9 |
| 613 | GO:0046556 | alpha-L-arabinofuranosidase activity | molecular\_function | 0.0 | 1.0 | 1.0 | 0 | cluster\_plot | 1 |
| 614 | GO:0016049 | cell growth | biological\_process | 0.0 | 1.0 | 1.0 | 0 | cluster\_plot | 17 |
| 615 | GO:1990104 | DNA bending complex | cellular\_component | 0.0 | 1.0 | 1.0 | 0 | cluster\_plot | 24 |
| 616 | GO:0045261 | proton-transporting ATP synthase complex, catalytic core F(1) | cellular\_component | 0.0 | 1.0 | 1.0 | 0 | cluster\_plot | 17 |
| 617 | GO:0000015 | phosphopyruvate hydratase complex | cellular\_component | 0.0 | 1.0 | 1.0 | 0 | cluster\_plot | 7 |
| 618 | GO:0016859 | cis-trans isomerase activity | molecular\_function | 0.0 | 1.0 | 1.0 | 0 | cluster\_plot | 48 |
| 619 | GO:0055029 | nuclear DNA-directed RNA polymerase complex | cellular\_component | 0.0 | 1.0 | 1.0 | 0 | cluster\_plot | 9 |
| 620 | GO:0006184 | GTP catabolic process | biological\_process | 0.0 | 1.0 | 1.0 | 0 | cluster\_plot | 22 |
| 621 | GO:0060089 | molecular transducer activity | molecular\_function | 0.0 | 1.0 | 1.0 | 0 | cluster\_plot | 141 |
| 622 | GO:0006367 | transcription initiation from RNA polymerase II promoter | biological\_process | 0.0 | 1.0 | 1.0 | 0 | cluster\_plot | 9 |
| 623 | GO:0045892 | negative regulation of transcription, DNA-templated | biological\_process | 0.0 | 1.0 | 1.0 | 0 | cluster\_plot | 8 |
| 624 | GO:0046961 | proton-transporting ATPase activity, rotational mechanism | molecular\_function | 0.0 | 1.0 | 1.0 | 0 | cluster\_plot | 24 |
| 625 | GO:0006659 | phosphatidylserine biosynthetic process | biological\_process | 0.0 | 1.0 | 1.0 | 0 | cluster\_plot | 2 |
| 626 | GO:0070818 | protoporphyrinogen oxidase activity | molecular\_function | 0.0 | 1.0 | 1.0 | 0 | cluster\_plot | 3 |
| 627 | GO:0016645 | oxidoreductase activity, acting on the CH-NH group of donors | molecular\_function | 0.0 | 1.0 | 1.0 | 0 | cluster\_plot | 29 |
| 628 | GO:0005665 | DNA-directed RNA polymerase II, core complex | cellular\_component | 0.0 | 1.0 | 1.0 | 0 | cluster\_plot | 3 |
| 629 | GO:0017169 | CDP-alcohol phosphatidyltransferase activity | molecular\_function | 0.0 | 1.0 | 1.0 | 0 | cluster\_plot | 1 |
| 630 | GO:0042083 | 5,10-methylenetetrahydrofolate-dependent methyltransferase activity | molecular\_function | 0.0 | 1.0 | 1.0 | 0 | cluster\_plot | 1 |
| 631 | GO:0009445 | putrescine metabolic process | biological\_process | 0.0 | 1.0 | 1.0 | 0 | cluster\_plot | 2 |
| 632 | GO:0046134 | pyrimidine nucleoside biosynthetic process | biological\_process | 0.0 | 1.0 | 1.0 | 0 | cluster\_plot | 12 |
| 633 | GO:0004455 | ketol-acid reductoisomerase activity | molecular\_function | 0.0 | 1.0 | 1.0 | 0 | cluster\_plot | 4 |
| 634 | GO:0006400 | tRNA modification | biological\_process | 0.0 | 1.0 | 1.0 | 0 | cluster\_plot | 16 |
| 635 | GO:0006614 | SRP-dependent cotranslational protein targeting to membrane | biological\_process | 0.0 | 1.0 | 1.0 | 0 | cluster\_plot | 17 |
| 636 | GO:0051783 | regulation of nuclear division | biological\_process | 0.0 | 1.0 | 1.0 | 0 | cluster\_plot | 5 |
| 637 | GO:0008616 | queuosine biosynthetic process | biological\_process | 0.0 | 1.0 | 1.0 | 0 | cluster\_plot | 3 |
| 638 | GO:0034472 | snRNA 3'-end processing | biological\_process | 0.0 | 1.0 | 1.0 | 0 | cluster\_plot | 1 |
| 639 | GO:0004845 | uracil phosphoribosyltransferase activity | molecular\_function | 0.0 | 1.0 | 1.0 | 0 | cluster\_plot | 1 |
| 640 | GO:0015036 | disulfide oxidoreductase activity | molecular\_function | 0.0 | 1.0 | 1.0 | 0 | cluster\_plot | 77 |
| 641 | GO:0009395 | phospholipid catabolic process | biological\_process | 0.0 | 1.0 | 1.0 | 0 | cluster\_plot | 1 |
| 642 | GO:0006543 | glutamine catabolic process | biological\_process | 0.0 | 1.0 | 1.0 | 0 | cluster\_plot | 3 |
| 643 | GO:0016863 | intramolecular oxidoreductase activity, transposing C=C bonds | molecular\_function | 0.0 | 1.0 | 1.0 | 0 | cluster\_plot | 5 |
| 644 | GO:0006887 | exocytosis | biological\_process | 0.0 | 1.0 | 1.0 | 0 | cluster\_plot | 40 |
| 645 | GO:0051060 | pullulanase activity | molecular\_function | 0.0 | 1.0 | 1.0 | 0 | cluster\_plot | 2 |
| 646 | GO:0006766 | vitamin metabolic process | biological\_process | 0.0 | 1.0 | 1.0 | 0 | cluster\_plot | 34 |
| 647 | GO:0005739 | mitochondrion | cellular\_component | 0.0 | 1.0 | 1.0 | 0 | cluster\_plot | 12 |
| 648 | GO:0019238 | cyclohydrolase activity | molecular\_function | 0.0 | 1.0 | 1.0 | 0 | cluster\_plot | 14 |
| 649 | GO:0019430 | removal of superoxide radicals | biological\_process | 0.0 | 1.0 | 1.0 | 0 | cluster\_plot | 1 |
| 650 | GO:0015718 | monocarboxylic acid transport | biological\_process | 0.0 | 1.0 | 1.0 | 0 | cluster\_plot | 6 |
| 651 | GO:0019856 | pyrimidine nucleobase biosynthetic process | biological\_process | 0.0 | 1.0 | 1.0 | 0 | cluster\_plot | 4 |
| 652 | GO:0005471 | ATP:ADP antiporter activity | molecular\_function | 0.0 | 1.0 | 1.0 | 0 | cluster\_plot | 1 |
| 653 | GO:0008373 | sialyltransferase activity | molecular\_function | 0.0 | 1.0 | 1.0 | 0 | cluster\_plot | 5 |
| 654 | GO:0030838 | positive regulation of actin filament polymerization | biological\_process | 0.0 | 1.0 | 1.0 | 0 | cluster\_plot | 4 |
| 655 | GO:0042401 | cellular biogenic amine biosynthetic process | biological\_process | 0.0 | 1.0 | 1.0 | 0 | cluster\_plot | 18 |
| 656 | GO:0044798 | nuclear transcription factor complex | cellular\_component | 0.0 | 1.0 | 1.0 | 0 | cluster\_plot | 15 |
| 657 | GO:0043138 | 3'-5' DNA helicase activity | molecular\_function | 0.0 | 1.0 | 1.0 | 0 | cluster\_plot | 5 |
| 658 | GO:0004190 | aspartic-type endopeptidase activity | molecular\_function | 0.0 | 1.0 | 1.0 | 0 | cluster\_plot | 25 |
| 659 | GO:0004198 | calcium-dependent cysteine-type endopeptidase activity | molecular\_function | 0.0 | 1.0 | 1.0 | 0 | cluster\_plot | 2 |
| 660 | GO:0004664 | prephenate dehydratase activity | molecular\_function | 0.0 | 1.0 | 1.0 | 0 | cluster\_plot | 8 |
| 661 | GO:0050307 | sucrose-phosphate phosphatase activity | molecular\_function | 0.0 | 1.0 | 1.0 | 0 | cluster\_plot | 6 |
| 662 | GO:0030312 | external encapsulating structure | cellular\_component | 0.0 | 1.0 | 1.0 | 0 | cluster\_plot | 137 |
| 663 | GO:0009890 | negative regulation of biosynthetic process | biological\_process | 0.0 | 1.0 | 1.0 | 0 | cluster\_plot | 20 |
| 664 | GO:0046037 | GMP metabolic process | biological\_process | 0.0 | 1.0 | 1.0 | 0 | cluster\_plot | 4 |
| 665 | GO:0044439 | peroxisomal part | cellular\_component | 0.0 | 1.0 | 1.0 | 0 | cluster\_plot | 11 |
| 666 | GO:0070409 | carbamoyl phosphate biosynthetic process | biological\_process | 0.0 | 1.0 | 1.0 | 0 | cluster\_plot | 3 |
| 667 | GO:0072350 | tricarboxylic acid metabolic process | biological\_process | 0.0 | 1.0 | 1.0 | 0 | cluster\_plot | 9 |
| 668 | GO:0009309 | amine biosynthetic process | biological\_process | 0.0 | 1.0 | 1.0 | 0 | cluster\_plot | 18 |
| 669 | GO:0009146 | purine nucleoside triphosphate catabolic process | biological\_process | 0.0 | 1.0 | 1.0 | 0 | cluster\_plot | 41 |
| 670 | GO:0016820 | hydrolase activity, acting on acid anhydrides, catalyzing transmembrane movement of substances | molecular\_function | 0.0 | 1.0 | 1.0 | 0 | cluster\_plot | 187 |
| 671 | GO:0015216 | purine nucleotide transmembrane transporter activity | molecular\_function | 0.0 | 1.0 | 1.0 | 0 | cluster\_plot | 1 |
| 672 | GO:0045980 | negative regulation of nucleotide metabolic process | biological\_process | 0.0 | 1.0 | 1.0 | 0 | cluster\_plot | 3 |
| 673 | GO:0008409 | 5'-3' exonuclease activity | molecular\_function | 0.0 | 1.0 | 1.0 | 0 | cluster\_plot | 6 |
| 674 | GO:0051090 | regulation of sequence-specific DNA binding transcription factor activity | biological\_process | 0.0 | 1.0 | 1.0 | 0 | cluster\_plot | 2 |
| 675 | GO:0004176 | ATP-dependent peptidase activity | molecular\_function | 0.0 | 1.0 | 1.0 | 0 | cluster\_plot | 15 |
| 676 | GO:0044451 | nucleoplasm part | cellular\_component | 0.0 | 1.0 | 1.0 | 0 | cluster\_plot | 61 |
| 677 | GO:0015980 | energy derivation by oxidation of organic compounds | biological\_process | 0.0 | 1.0 | 1.0 | 0 | cluster\_plot | 4 |
| 678 | GO:0015684 | ferrous iron transport | biological\_process | 0.0 | 1.0 | 1.0 | 0 | cluster\_plot | 3 |
| 679 | GO:0045226 | extracellular polysaccharide biosynthetic process | biological\_process | 0.0 | 1.0 | 1.0 | 0 | cluster\_plot | 7 |
| 680 | GO:0016157 | sucrose synthase activity | molecular\_function | 0.0 | 1.0 | 1.0 | 0 | cluster\_plot | 12 |
| 681 | GO:0000255 | allantoin metabolic process | biological\_process | 0.0 | 1.0 | 1.0 | 0 | cluster\_plot | 2 |
| 682 | GO:0051119 | sugar transmembrane transporter activity | molecular\_function | 0.0 | 1.0 | 1.0 | 0 | cluster\_plot | 18 |
| 683 | GO:0008277 | regulation of G-protein coupled receptor protein signaling pathway | biological\_process | 0.0 | 1.0 | 1.0 | 0 | cluster\_plot | 2 |
| 684 | GO:0004635 | phosphoribosyl-AMP cyclohydrolase activity | molecular\_function | 0.0 | 1.0 | 1.0 | 0 | cluster\_plot | 3 |
| 685 | GO:0015994 | chlorophyll metabolic process | biological\_process | 0.0 | 1.0 | 1.0 | 0 | cluster\_plot | 22 |
| 686 | GO:0004651 | polynucleotide 5'-phosphatase activity | molecular\_function | 0.0 | 1.0 | 1.0 | 0 | cluster\_plot | 1 |
| 687 | GO:0070887 | cellular response to chemical stimulus | biological\_process | 0.0 | 1.0 | 1.0 | 0 | cluster\_plot | 1 |
| 688 | GO:0046429 | 4-hydroxy-3-methylbut-2-en-1-yl diphosphate synthase activity | molecular\_function | 0.0 | 1.0 | 1.0 | 0 | cluster\_plot | 2 |
| 689 | GO:0004830 | tryptophan-tRNA ligase activity | molecular\_function | 0.0 | 1.0 | 1.0 | 0 | cluster\_plot | 2 |
| 690 | GO:0003849 | 3-deoxy-7-phosphoheptulonate synthase activity | molecular\_function | 0.0 | 1.0 | 1.0 | 0 | cluster\_plot | 5 |
| 691 | GO:0072599 | establishment of protein localization to endoplasmic reticulum | biological\_process | 0.0 | 1.0 | 1.0 | 0 | cluster\_plot | 17 |
| 692 | GO:0009263 | deoxyribonucleotide biosynthetic process | biological\_process | 0.0 | 1.0 | 1.0 | 0 | cluster\_plot | 3 |
| 693 | GO:0046173 | polyol biosynthetic process | biological\_process | 0.0 | 1.0 | 1.0 | 0 | cluster\_plot | 6 |
| 694 | GO:0051246 | regulation of protein metabolic process | biological\_process | 0.0 | 1.0 | 1.0 | 0 | cluster\_plot | 28 |
| 695 | GO:0004109 | coproporphyrinogen oxidase activity | molecular\_function | 0.0 | 1.0 | 1.0 | 0 | cluster\_plot | 1 |
| 696 | GO:0035384 | thioester biosynthetic process | biological\_process | 0.0 | 1.0 | 1.0 | 0 | cluster\_plot | 2 |
| 697 | GO:0044801 | single-organism membrane fusion | biological\_process | 0.0 | 1.0 | 1.0 | 0 | cluster\_plot | 2 |
| 698 | GO:0006741 | NADP biosynthetic process | biological\_process | 0.0 | 1.0 | 1.0 | 0 | cluster\_plot | 6 |
| 699 | GO:0019692 | deoxyribose phosphate metabolic process | biological\_process | 0.0 | 1.0 | 1.0 | 0 | cluster\_plot | 4 |
| 700 | GO:0006308 | DNA catabolic process | biological\_process | 0.0 | 1.0 | 1.0 | 0 | cluster\_plot | 4 |
| 701 | GO:0006414 | translational elongation | biological\_process | 0.0 | 1.0 | 1.0 | 0 | cluster\_plot | 53 |
| 702 | GO:0051336 | regulation of hydrolase activity | biological\_process | 0.0 | 1.0 | 1.0 | 0 | cluster\_plot | 52 |
| 703 | GO:0005732 | small nucleolar ribonucleoprotein complex | cellular\_component | 0.0 | 1.0 | 1.0 | 0 | cluster\_plot | 1 |
| 704 | GO:0007021 | tubulin complex assembly | biological\_process | 0.0 | 1.0 | 1.0 | 0 | cluster\_plot | 3 |
| 705 | GO:0045010 | actin nucleation | biological\_process | 0.0 | 1.0 | 1.0 | 0 | cluster\_plot | 4 |
| 706 | GO:0043650 | dicarboxylic acid biosynthetic process | biological\_process | 0.0 | 1.0 | 1.0 | 0 | cluster\_plot | 8 |
| 707 | GO:0008878 | glucose-1-phosphate adenylyltransferase activity | molecular\_function | 0.0 | 1.0 | 1.0 | 0 | cluster\_plot | 3 |
| 708 | GO:0009133 | nucleoside diphosphate biosynthetic process | biological\_process | 0.0 | 1.0 | 1.0 | 0 | cluster\_plot | 2 |
| 709 | GO:0004057 | arginyltransferase activity | molecular\_function | 0.0 | 1.0 | 1.0 | 0 | cluster\_plot | 2 |
| 710 | GO:0015914 | phospholipid transport | biological\_process | 0.0 | 1.0 | 1.0 | 0 | cluster\_plot | 17 |
| 711 | GO:0004803 | transposase activity | molecular\_function | 0.0 | 1.0 | 1.0 | 0 | cluster\_plot | 1 |
| 712 | GO:0015850 | organic hydroxy compound transport | biological\_process | 0.0 | 1.0 | 1.0 | 0 | cluster\_plot | 1 |
| 713 | GO:0071805 | potassium ion transmembrane transport | biological\_process | 0.0 | 1.0 | 1.0 | 0 | cluster\_plot | 23 |
| 714 | GO:0006549 | isoleucine metabolic process | biological\_process | 0.0 | 1.0 | 1.0 | 0 | cluster\_plot | 3 |
| 715 | GO:0016410 | N-acyltransferase activity | molecular\_function | 0.0 | 1.0 | 1.0 | 0 | cluster\_plot | 58 |
| 716 | GO:0004072 | aspartate kinase activity | molecular\_function | 0.0 | 1.0 | 1.0 | 0 | cluster\_plot | 3 |
| 717 | GO:0006032 | chitin catabolic process | biological\_process | 0.0 | 1.0 | 1.0 | 0 | cluster\_plot | 18 |
| 718 | GO:0015377 | cation:chloride symporter activity | molecular\_function | 0.0 | 1.0 | 1.0 | 0 | cluster\_plot | 2 |
| 719 | GO:0046112 | nucleobase biosynthetic process | biological\_process | 0.0 | 1.0 | 1.0 | 0 | cluster\_plot | 19 |
| 720 | GO:0005938 | cell cortex | cellular\_component | 0.0 | 1.0 | 1.0 | 0 | cluster\_plot | 2 |
| 721 | GO:0016108 | tetraterpenoid metabolic process | biological\_process | 0.0 | 1.0 | 1.0 | 0 | cluster\_plot | 9 |
| 722 | GO:0008216 | spermidine metabolic process | biological\_process | 0.0 | 1.0 | 1.0 | 0 | cluster\_plot | 6 |
| 723 | GO:0045727 | positive regulation of translation | biological\_process | 0.0 | 1.0 | 1.0 | 0 | cluster\_plot | 3 |
| 724 | GO:0004612 | phosphoenolpyruvate carboxykinase (ATP) activity | molecular\_function | 0.0 | 1.0 | 1.0 | 0 | cluster\_plot | 6 |
| 725 | GO:0000124 | SAGA complex | cellular\_component | 0.0 | 1.0 | 1.0 | 0 | cluster\_plot | 4 |
| 726 | GO:0006353 | DNA-templated transcription, termination | biological\_process | 0.0 | 1.0 | 1.0 | 0 | cluster\_plot | 2 |
| 727 | GO:0047429 | nucleoside-triphosphate diphosphatase activity | molecular\_function | 0.0 | 1.0 | 1.0 | 0 | cluster\_plot | 1 |
| 728 | GO:0008172 | S-methyltransferase activity | molecular\_function | 0.0 | 1.0 | 1.0 | 0 | cluster\_plot | 7 |
| 729 | GO:0046937 | phytochelatin metabolic process | biological\_process | 0.0 | 1.0 | 1.0 | 0 | cluster\_plot | 3 |
| 730 | GO:0005786 | signal recognition particle, endoplasmic reticulum targeting | cellular\_component | 0.0 | 1.0 | 1.0 | 0 | cluster\_plot | 1 |
| 731 | GO:1901568 | fatty acid derivative metabolic process | biological\_process | 0.0 | 1.0 | 1.0 | 0 | cluster\_plot | 3 |
| 732 | GO:0016748 | succinyltransferase activity | molecular\_function | 0.0 | 1.0 | 1.0 | 0 | cluster\_plot | 3 |
| 733 | GO:0003951 | NAD+ kinase activity | molecular\_function | 0.0 | 1.0 | 1.0 | 0 | cluster\_plot | 6 |
| 734 | GO:0000184 | nuclear-transcribed mRNA catabolic process, nonsense-mediated decay | biological\_process | 0.0 | 1.0 | 1.0 | 0 | cluster\_plot | 3 |
| 735 | GO:0043038 | amino acid activation | biological\_process | 0.0 | 1.0 | 1.0 | 0 | cluster\_plot | 87 |
| 736 | GO:0016197 | endosomal transport | biological\_process | 0.0 | 1.0 | 1.0 | 0 | cluster\_plot | 3 |
| 737 | GO:0016972 | thiol oxidase activity | molecular\_function | 0.0 | 1.0 | 1.0 | 0 | cluster\_plot | 4 |
| 738 | GO:0016743 | carboxyl- or carbamoyltransferase activity | molecular\_function | 0.0 | 1.0 | 1.0 | 0 | cluster\_plot | 5 |
| 739 | GO:0071265 | L-methionine biosynthetic process | biological\_process | 0.0 | 1.0 | 1.0 | 0 | cluster\_plot | 3 |
| 740 | GO:0006527 | arginine catabolic process | biological\_process | 0.0 | 1.0 | 1.0 | 0 | cluster\_plot | 1 |
| 741 | GO:0006281 | DNA repair | biological\_process | 0.0 | 1.0 | 1.0 | 0 | cluster\_plot | 128 |
| 742 | GO:0002098 | tRNA wobble uridine modification | biological\_process | 0.0 | 1.0 | 1.0 | 0 | cluster\_plot | 1 |
| 743 | GO:0004735 | pyrroline-5-carboxylate reductase activity | molecular\_function | 0.0 | 1.0 | 1.0 | 0 | cluster\_plot | 2 |
| 744 | GO:0051301 | cell division | biological\_process | 0.0 | 1.0 | 1.0 | 0 | cluster\_plot | 6 |
| 745 | GO:0006779 | porphyrin-containing compound biosynthetic process | biological\_process | 0.0 | 1.0 | 1.0 | 0 | cluster\_plot | 35 |
| 746 | GO:0008417 | fucosyltransferase activity | molecular\_function | 0.0 | 1.0 | 1.0 | 0 | cluster\_plot | 5 |
| 747 | GO:0009968 | negative regulation of signal transduction | biological\_process | 0.0 | 1.0 | 1.0 | 0 | cluster\_plot | 2 |
| 748 | GO:0010557 | positive regulation of macromolecule biosynthetic process | biological\_process | 0.0 | 1.0 | 1.0 | 0 | cluster\_plot | 7 |
| 749 | GO:0009221 | pyrimidine deoxyribonucleotide biosynthetic process | biological\_process | 0.0 | 1.0 | 1.0 | 0 | cluster\_plot | 3 |
| 750 | GO:0046051 | UTP metabolic process | biological\_process | 0.0 | 1.0 | 1.0 | 0 | cluster\_plot | 7 |
| 751 | GO:0007008 | outer mitochondrial membrane organization | biological\_process | 0.0 | 1.0 | 1.0 | 0 | cluster\_plot | 10 |
| 752 | GO:0008615 | pyridoxine biosynthetic process | biological\_process | 0.0 | 1.0 | 1.0 | 0 | cluster\_plot | 1 |
| 753 | GO:0016885 | ligase activity, forming carbon-carbon bonds | molecular\_function | 0.0 | 1.0 | 1.0 | 0 | cluster\_plot | 7 |
| 754 | GO:0008299 | isoprenoid biosynthetic process | biological\_process | 0.0 | 1.0 | 1.0 | 0 | cluster\_plot | 40 |
| 755 | GO:0008113 | peptide-methionine (S)-S-oxide reductase activity | molecular\_function | 0.0 | 1.0 | 1.0 | 0 | cluster\_plot | 5 |
| 756 | GO:0033925 | mannosyl-glycoprotein endo-beta-N-acetylglucosaminidase activity | molecular\_function | 0.0 | 1.0 | 1.0 | 0 | cluster\_plot | 3 |
| 757 | GO:0004812 | aminoacyl-tRNA ligase activity | molecular\_function | 0.0 | 1.0 | 1.0 | 0 | cluster\_plot | 84 |
| 758 | GO:0008418 | protein-N-terminal asparagine amidohydrolase activity | molecular\_function | 0.0 | 1.0 | 1.0 | 0 | cluster\_plot | 4 |
| 759 | GO:0018198 | peptidyl-cysteine modification | biological\_process | 0.0 | 1.0 | 1.0 | 0 | cluster\_plot | 1 |
| 760 | GO:0061505 | DNA topoisomerase II activity | molecular\_function | 0.0 | 1.0 | 1.0 | 0 | cluster\_plot | 11 |
| 761 | GO:0072351 | tricarboxylic acid biosynthetic process | biological\_process | 0.0 | 1.0 | 1.0 | 0 | cluster\_plot | 2 |
| 762 | GO:0043648 | dicarboxylic acid metabolic process | biological\_process | 0.0 | 1.0 | 1.0 | 0 | cluster\_plot | 44 |
| 763 | GO:0003909 | DNA ligase activity | molecular\_function | 0.0 | 1.0 | 1.0 | 0 | cluster\_plot | 4 |
| 764 | GO:0043566 | structure-specific DNA binding | molecular\_function | 0.0 | 1.0 | 1.0 | 0 | cluster\_plot | 28 |
| 765 | GO:0031301 | integral component of organelle membrane | cellular\_component | 0.0 | 1.0 | 1.0 | 0 | cluster\_plot | 15 |
| 766 | GO:0042325 | regulation of phosphorylation | biological\_process | 0.0 | 1.0 | 1.0 | 0 | cluster\_plot | 11 |
| 767 | GO:0008134 | transcription factor binding | molecular\_function | 0.0 | 1.0 | 1.0 | 0 | cluster\_plot | 10 |
| 768 | GO:0004560 | alpha-L-fucosidase activity | molecular\_function | 0.0 | 1.0 | 1.0 | 0 | cluster\_plot | 4 |
| 769 | GO:0008515 | sucrose transmembrane transporter activity | molecular\_function | 0.0 | 1.0 | 1.0 | 0 | cluster\_plot | 6 |
| 770 | GO:0006166 | purine ribonucleoside salvage | biological\_process | 0.0 | 1.0 | 1.0 | 0 | cluster\_plot | 2 |
| 771 | GO:0016823 | hydrolase activity, acting on acid carbon-carbon bonds, in ketonic substances | molecular\_function | 0.0 | 1.0 | 1.0 | 0 | cluster\_plot | 1 |
| 772 | GO:0010109 | regulation of photosynthesis | biological\_process | 0.0 | 1.0 | 1.0 | 0 | cluster\_plot | 4 |
| 773 | GO:0003689 | DNA clamp loader activity | molecular\_function | 0.0 | 1.0 | 1.0 | 0 | cluster\_plot | 5 |
| 774 | GO:0097354 | prenylation | biological\_process | 0.0 | 1.0 | 1.0 | 0 | cluster\_plot | 4 |
| 775 | GO:0071616 | acyl-CoA biosynthetic process | biological\_process | 0.0 | 1.0 | 1.0 | 0 | cluster\_plot | 2 |
| 776 | GO:0090329 | regulation of DNA-dependent DNA replication | biological\_process | 0.0 | 1.0 | 1.0 | 0 | cluster\_plot | 2 |
| 777 | GO:0003949 | 1-(5-phosphoribosyl)-5-[(5-phosphoribosylamino)methylideneamino]imidazole-4-carboxamide isomerase activity | molecular\_function | 0.0 | 1.0 | 1.0 | 0 | cluster\_plot | 3 |
| 778 | GO:0004523 | RNA-DNA hybrid ribonuclease activity | molecular\_function | 0.0 | 1.0 | 1.0 | 0 | cluster\_plot | 2 |
| 779 | GO:0004401 | histidinol-phosphatase activity | molecular\_function | 0.0 | 1.0 | 1.0 | 0 | cluster\_plot | 2 |
| 780 | GO:0004729 | oxygen-dependent protoporphyrinogen oxidase activity | molecular\_function | 0.0 | 1.0 | 1.0 | 0 | cluster\_plot | 3 |
| 781 | GO:0006364 | rRNA processing | biological\_process | 0.0 | 1.0 | 1.0 | 0 | cluster\_plot | 40 |
| 782 | GO:0004814 | arginine-tRNA ligase activity | molecular\_function | 0.0 | 1.0 | 1.0 | 0 | cluster\_plot | 1 |
| 783 | GO:0004392 | heme oxygenase (decyclizing) activity | molecular\_function | 0.0 | 1.0 | 1.0 | 0 | cluster\_plot | 3 |
| 784 | GO:0019207 | kinase regulator activity | molecular\_function | 0.0 | 1.0 | 1.0 | 0 | cluster\_plot | 17 |
| 785 | GO:0008898 | S-adenosylmethionine-homocysteine S-methyltransferase activity | molecular\_function | 0.0 | 1.0 | 1.0 | 0 | cluster\_plot | 4 |
| 786 | GO:0006694 | steroid biosynthetic process | biological\_process | 0.0 | 1.0 | 1.0 | 0 | cluster\_plot | 20 |
| 787 | GO:0004056 | argininosuccinate lyase activity | molecular\_function | 0.0 | 1.0 | 1.0 | 0 | cluster\_plot | 3 |
| 788 | GO:0015934 | large ribosomal subunit | cellular\_component | 0.0 | 1.0 | 1.0 | 0 | cluster\_plot | 32 |
| 789 | GO:0016116 | carotenoid metabolic process | biological\_process | 0.0 | 1.0 | 1.0 | 0 | cluster\_plot | 9 |
| 790 | GO:0008175 | tRNA methyltransferase activity | molecular\_function | 0.0 | 1.0 | 1.0 | 0 | cluster\_plot | 11 |
| 791 | GO:0009916 | alternative oxidase activity | molecular\_function | 0.0 | 1.0 | 1.0 | 0 | cluster\_plot | 5 |
| 792 | GO:0006915 | apoptotic process | biological\_process | 0.0 | 1.0 | 1.0 | 0 | cluster\_plot | 1 |
| 793 | GO:0010646 | regulation of cell communication | biological\_process | 0.0 | 1.0 | 1.0 | 0 | cluster\_plot | 18 |
| 794 | GO:0016719 | carotene 7,8-desaturase activity | molecular\_function | 0.0 | 1.0 | 1.0 | 0 | cluster\_plot | 1 |
| 795 | GO:0009259 | ribonucleotide metabolic process | biological\_process | 0.0 | 1.0 | 1.0 | 0 | cluster\_plot | 122 |
| 796 | GO:0006228 | UTP biosynthetic process | biological\_process | 0.0 | 1.0 | 1.0 | 0 | cluster\_plot | 7 |
| 797 | GO:0004484 | mRNA guanylyltransferase activity | molecular\_function | 0.0 | 1.0 | 1.0 | 0 | cluster\_plot | 3 |
| 798 | GO:0016647 | oxidoreductase activity, acting on the CH-NH group of donors, oxygen as acceptor | molecular\_function | 0.0 | 1.0 | 1.0 | 0 | cluster\_plot | 3 |
| 799 | GO:0045550 | geranylgeranyl reductase activity | molecular\_function | 0.0 | 1.0 | 1.0 | 0 | cluster\_plot | 1 |
| 800 | GO:0015079 | potassium ion transmembrane transporter activity | molecular\_function | 0.0 | 1.0 | 1.0 | 0 | cluster\_plot | 23 |
| 801 | GO:0016842 | amidine-lyase activity | molecular\_function | 0.0 | 1.0 | 1.0 | 0 | cluster\_plot | 4 |
| 802 | GO:0008176 | tRNA (guanine-N7-)-methyltransferase activity | molecular\_function | 0.0 | 1.0 | 1.0 | 0 | cluster\_plot | 4 |
| 803 | GO:0004129 | cytochrome-c oxidase activity | molecular\_function | 0.0 | 1.0 | 1.0 | 0 | cluster\_plot | 21 |
| 804 | GO:0009065 | glutamine family amino acid catabolic process | biological\_process | 0.0 | 1.0 | 1.0 | 0 | cluster\_plot | 8 |
| 805 | GO:0051056 | regulation of small GTPase mediated signal transduction | biological\_process | 0.0 | 1.0 | 1.0 | 0 | cluster\_plot | 12 |
| 806 | GO:0019277 | UDP-N-acetylgalactosamine biosynthetic process | biological\_process | 0.0 | 1.0 | 1.0 | 0 | cluster\_plot | 2 |
| 807 | GO:0042398 | cellular modified amino acid biosynthetic process | biological\_process | 0.0 | 1.0 | 1.0 | 0 | cluster\_plot | 44 |
| 808 | GO:0065004 | protein-DNA complex assembly | biological\_process | 0.0 | 1.0 | 1.0 | 0 | cluster\_plot | 32 |
| 809 | GO:0019900 | kinase binding | molecular\_function | 0.0 | 1.0 | 1.0 | 0 | cluster\_plot | 11 |
| 810 | GO:0004326 | tetrahydrofolylpolyglutamate synthase activity | molecular\_function | 0.0 | 1.0 | 1.0 | 0 | cluster\_plot | 4 |
| 811 | GO:0035556 | intracellular signal transduction | biological\_process | 0.0 | 1.0 | 1.0 | 0 | cluster\_plot | 129 |
| 812 | GO:0009360 | DNA polymerase III complex | cellular\_component | 0.0 | 1.0 | 1.0 | 0 | cluster\_plot | 6 |
| 813 | GO:0016903 | oxidoreductase activity, acting on the aldehyde or oxo group of donors | molecular\_function | 0.0 | 1.0 | 1.0 | 0 | cluster\_plot | 42 |
| 814 | GO:0009118 | regulation of nucleoside metabolic process | biological\_process | 0.0 | 1.0 | 1.0 | 0 | cluster\_plot | 51 |
| 815 | GO:0004571 | mannosyl-oligosaccharide 1,2-alpha-mannosidase activity | molecular\_function | 0.0 | 1.0 | 1.0 | 0 | cluster\_plot | 4 |
| 816 | GO:0005088 | Ras guanyl-nucleotide exchange factor activity | molecular\_function | 0.0 | 1.0 | 1.0 | 0 | cluster\_plot | 14 |
| 817 | GO:0015932 | nucleobase-containing compound transmembrane transporter activity | molecular\_function | 0.0 | 1.0 | 1.0 | 0 | cluster\_plot | 19 |
| 818 | GO:0030126 | COPI vesicle coat | cellular\_component | 0.0 | 1.0 | 1.0 | 0 | cluster\_plot | 8 |
| 819 | GO:0006282 | regulation of DNA repair | biological\_process | 0.0 | 1.0 | 1.0 | 0 | cluster\_plot | 2 |
| 820 | GO:0015977 | carbon fixation | biological\_process | 0.0 | 1.0 | 1.0 | 0 | cluster\_plot | 8 |
| 821 | GO:0009074 | aromatic amino acid family catabolic process | biological\_process | 0.0 | 1.0 | 1.0 | 0 | cluster\_plot | 4 |
| 822 | GO:0016822 | hydrolase activity, acting on acid carbon-carbon bonds | molecular\_function | 0.0 | 1.0 | 1.0 | 0 | cluster\_plot | 1 |
| 823 | GO:0004106 | chorismate mutase activity | molecular\_function | 0.0 | 1.0 | 1.0 | 0 | cluster\_plot | 4 |
| 824 | GO:0044743 | intracellular protein transmembrane import | biological\_process | 0.0 | 1.0 | 1.0 | 0 | cluster\_plot | 13 |
| 825 | GO:0070403 | NAD+ binding | molecular\_function | 0.0 | 1.0 | 1.0 | 0 | cluster\_plot | 3 |
| 826 | GO:0016744 | transferase activity, transferring aldehyde or ketonic groups | molecular\_function | 0.0 | 1.0 | 1.0 | 0 | cluster\_plot | 13 |
| 827 | GO:0016998 | cell wall macromolecule catabolic process | biological\_process | 0.0 | 1.0 | 1.0 | 0 | cluster\_plot | 55 |
| 828 | GO:1902222 | erythrose 4-phosphate/phosphoenolpyruvate family amino acid catabolic process | biological\_process | 0.0 | 1.0 | 1.0 | 0 | cluster\_plot | 4 |
| 829 | GO:0006821 | chloride transport | biological\_process | 0.0 | 1.0 | 1.0 | 0 | cluster\_plot | 13 |
| 830 | GO:0000303 | response to superoxide | biological\_process | 0.0 | 1.0 | 1.0 | 0 | cluster\_plot | 1 |
| 831 | GO:0003978 | UDP-glucose 4-epimerase activity | molecular\_function | 0.0 | 1.0 | 1.0 | 0 | cluster\_plot | 7 |
| 832 | GO:0043874 | acireductone synthase activity | molecular\_function | 0.0 | 1.0 | 1.0 | 0 | cluster\_plot | 1 |
| 833 | GO:0008360 | regulation of cell shape | biological\_process | 0.0 | 1.0 | 1.0 | 0 | cluster\_plot | 5 |
| 834 | GO:0022834 | ligand-gated channel activity | molecular\_function | 0.0 | 1.0 | 1.0 | 0 | cluster\_plot | 37 |
| 835 | GO:0016559 | peroxisome fission | biological\_process | 0.0 | 1.0 | 1.0 | 0 | cluster\_plot | 9 |
| 836 | GO:0030119 | AP-type membrane coat adaptor complex | cellular\_component | 0.0 | 1.0 | 1.0 | 0 | cluster\_plot | 23 |
| 837 | GO:0046040 | IMP metabolic process | biological\_process | 0.0 | 1.0 | 1.0 | 0 | cluster\_plot | 15 |
| 838 | GO:0044036 | cell wall macromolecule metabolic process | biological\_process | 0.0 | 1.0 | 1.0 | 0 | cluster\_plot | 55 |
| 839 | GO:0016620 | oxidoreductase activity, acting on the aldehyde or oxo group of donors, NAD or NADP as acceptor | molecular\_function | 0.0 | 1.0 | 1.0 | 0 | cluster\_plot | 33 |
| 840 | GO:0042724 | thiamine-containing compound biosynthetic process | biological\_process | 0.0 | 1.0 | 1.0 | 0 | cluster\_plot | 8 |
| 841 | GO:0000105 | histidine biosynthetic process | biological\_process | 0.0 | 1.0 | 1.0 | 0 | cluster\_plot | 17 |
| 842 | GO:0032879 | regulation of localization | biological\_process | 0.0 | 1.0 | 1.0 | 0 | cluster\_plot | 2 |
| 843 | GO:0003910 | DNA ligase (ATP) activity | molecular\_function | 0.0 | 1.0 | 1.0 | 0 | cluster\_plot | 4 |
| 844 | GO:0005758 | mitochondrial intermembrane space | cellular\_component | 0.0 | 1.0 | 1.0 | 0 | cluster\_plot | 2 |
| 845 | GO:0019566 | arabinose metabolic process | biological\_process | 0.0 | 1.0 | 1.0 | 0 | cluster\_plot | 1 |
| 846 | GO:1902582 | single-organism intracellular transport | biological\_process | 0.0 | 1.0 | 1.0 | 0 | cluster\_plot | 113 |
| 847 | GO:0034660 | ncRNA metabolic process | biological\_process | 0.0 | 1.0 | 1.0 | 0 | cluster\_plot | 183 |
| 848 | GO:0008170 | N-methyltransferase activity | molecular\_function | 0.0 | 1.0 | 1.0 | 0 | cluster\_plot | 25 |
| 849 | GO:0046916 | cellular transition metal ion homeostasis | biological\_process | 0.0 | 1.0 | 1.0 | 0 | cluster\_plot | 6 |
| 850 | GO:0009126 | purine nucleoside monophosphate metabolic process | biological\_process | 0.0 | 1.0 | 1.0 | 0 | cluster\_plot | 79 |
| 851 | GO:0008653 | lipopolysaccharide metabolic process | biological\_process | 0.0 | 1.0 | 1.0 | 0 | cluster\_plot | 2 |
| 852 | GO:0006784 | heme a biosynthetic process | biological\_process | 0.0 | 1.0 | 1.0 | 0 | cluster\_plot | 1 |
| 853 | GO:0019203 | carbohydrate phosphatase activity | molecular\_function | 0.0 | 1.0 | 1.0 | 0 | cluster\_plot | 17 |
| 854 | GO:0008474 | palmitoyl-(protein) hydrolase activity | molecular\_function | 0.0 | 1.0 | 1.0 | 0 | cluster\_plot | 4 |
| 855 | GO:0009607 | response to biotic stimulus | biological\_process | 0.0 | 1.0 | 1.0 | 0 | cluster\_plot | 71 |
| 856 | GO:0071555 | cell wall organization | biological\_process | 0.0 | 1.0 | 1.0 | 0 | cluster\_plot | 90 |
| 857 | GO:0000062 | fatty-acyl-CoA binding | molecular\_function | 0.0 | 1.0 | 1.0 | 0 | cluster\_plot | 5 |
| 858 | GO:0009186 | deoxyribonucleoside diphosphate metabolic process | biological\_process | 0.0 | 1.0 | 1.0 | 0 | cluster\_plot | 8 |
| 859 | GO:0043044 | ATP-dependent chromatin remodeling | biological\_process | 0.0 | 1.0 | 1.0 | 0 | cluster\_plot | 3 |
| 860 | GO:0018339 | peptidyl-L-beta-methylthioaspartic acid biosynthetic process from peptidyl-aspartic acid | biological\_process | 0.0 | 1.0 | 1.0 | 0 | cluster\_plot | 1 |
| 861 | GO:0017183 | peptidyl-diphthamide biosynthetic process from peptidyl-histidine | biological\_process | 0.0 | 1.0 | 1.0 | 0 | cluster\_plot | 4 |
| 862 | GO:0016895 | exodeoxyribonuclease activity, producing 5'-phosphomonoesters | molecular\_function | 0.0 | 1.0 | 1.0 | 0 | cluster\_plot | 2 |
| 863 | GO:0015939 | pantothenate metabolic process | biological\_process | 0.0 | 1.0 | 1.0 | 0 | cluster\_plot | 7 |
| 864 | GO:0015215 | nucleotide transmembrane transporter activity | molecular\_function | 0.0 | 1.0 | 1.0 | 0 | cluster\_plot | 10 |
| 865 | GO:0008685 | 2-C-methyl-D-erythritol 2,4-cyclodiphosphate synthase activity | molecular\_function | 0.0 | 1.0 | 1.0 | 0 | cluster\_plot | 2 |
| 866 | GO:0031124 | mRNA 3'-end processing | biological\_process | 0.0 | 1.0 | 1.0 | 0 | cluster\_plot | 3 |
| 867 | GO:0006479 | protein methylation | biological\_process | 0.0 | 1.0 | 1.0 | 0 | cluster\_plot | 26 |
| 868 | GO:0015940 | pantothenate biosynthetic process | biological\_process | 0.0 | 1.0 | 1.0 | 0 | cluster\_plot | 7 |
| 869 | GO:1902221 | erythrose 4-phosphate/phosphoenolpyruvate family amino acid metabolic process | biological\_process | 0.0 | 1.0 | 1.0 | 0 | cluster\_plot | 12 |
| 870 | GO:0009098 | leucine biosynthetic process | biological\_process | 0.0 | 1.0 | 1.0 | 0 | cluster\_plot | 4 |
| 871 | GO:0003922 | GMP synthase (glutamine-hydrolyzing) activity | molecular\_function | 0.0 | 1.0 | 1.0 | 0 | cluster\_plot | 4 |
| 872 | GO:0019400 | alditol metabolic process | biological\_process | 0.0 | 1.0 | 1.0 | 0 | cluster\_plot | 19 |
| 873 | GO:0031334 | positive regulation of protein complex assembly | biological\_process | 0.0 | 1.0 | 1.0 | 0 | cluster\_plot | 4 |
| 874 | GO:0015093 | ferrous iron transmembrane transporter activity | molecular\_function | 0.0 | 1.0 | 1.0 | 0 | cluster\_plot | 3 |
| 875 | GO:0008440 | inositol-1,4,5-trisphosphate 3-kinase activity | molecular\_function | 0.0 | 1.0 | 1.0 | 0 | cluster\_plot | 3 |
| 876 | GO:0031267 | small GTPase binding | molecular\_function | 0.0 | 1.0 | 1.0 | 0 | cluster\_plot | 19 |
| 877 | GO:0016636 | oxidoreductase activity, acting on the CH-CH group of donors, iron-sulfur protein as acceptor | molecular\_function | 0.0 | 1.0 | 1.0 | 0 | cluster\_plot | 2 |
| 878 | GO:0019213 | deacetylase activity | molecular\_function | 0.0 | 1.0 | 1.0 | 0 | cluster\_plot | 1 |
| 879 | GO:0016592 | mediator complex | cellular\_component | 0.0 | 1.0 | 1.0 | 0 | cluster\_plot | 21 |
| 880 | GO:0045005 | maintenance of fidelity involved in DNA-dependent DNA replication | biological\_process | 0.0 | 1.0 | 1.0 | 0 | cluster\_plot | 2 |
| 881 | GO:0009522 | photosystem I | cellular\_component | 0.0 | 1.0 | 1.0 | 0 | cluster\_plot | 16 |
| 882 | GO:0080134 | regulation of response to stress | biological\_process | 0.0 | 1.0 | 1.0 | 0 | cluster\_plot | 2 |
| 883 | GO:0005744 | mitochondrial inner membrane presequence translocase complex | cellular\_component | 0.0 | 1.0 | 1.0 | 0 | cluster\_plot | 1 |
| 884 | GO:0000139 | Golgi membrane | cellular\_component | 0.0 | 1.0 | 1.0 | 0 | cluster\_plot | 19 |
| 885 | GO:0007205 | protein kinase C-activating G-protein coupled receptor signaling pathway | biological\_process | 0.0 | 1.0 | 1.0 | 0 | cluster\_plot | 17 |
| 886 | GO:0004764 | shikimate 3-dehydrogenase (NADP+) activity | molecular\_function | 0.0 | 1.0 | 1.0 | 0 | cluster\_plot | 5 |
| 887 | GO:0042255 | ribosome assembly | biological\_process | 0.0 | 1.0 | 1.0 | 0 | cluster\_plot | 2 |
| 888 | GO:0006366 | transcription from RNA polymerase II promoter | biological\_process | 0.0 | 1.0 | 1.0 | 0 | cluster\_plot | 3 |
| 889 | GO:0016810 | hydrolase activity, acting on carbon-nitrogen (but not peptide) bonds | molecular\_function | 0.0 | 1.0 | 1.0 | 0 | cluster\_plot | 56 |
| 890 | GO:0004853 | uroporphyrinogen decarboxylase activity | molecular\_function | 0.0 | 1.0 | 1.0 | 0 | cluster\_plot | 5 |
| 891 | GO:0006207 | 'de novo' pyrimidine nucleobase biosynthetic process | biological\_process | 0.0 | 1.0 | 1.0 | 0 | cluster\_plot | 4 |
| 892 | GO:0043604 | amide biosynthetic process | biological\_process | 0.0 | 1.0 | 1.0 | 0 | cluster\_plot | 33 |
| 893 | GO:0004871 | signal transducer activity | molecular\_function | 0.0 | 1.0 | 1.0 | 0 | cluster\_plot | 141 |
| 894 | GO:0097506 | deaminated base DNA N-glycosylase activity | molecular\_function | 0.0 | 1.0 | 1.0 | 0 | cluster\_plot | 1 |
| 895 | GO:0045454 | cell redox homeostasis | biological\_process | 0.0 | 1.0 | 1.0 | 0 | cluster\_plot | 155 |
| 896 | GO:0005231 | excitatory extracellular ligand-gated ion channel activity | molecular\_function | 0.0 | 1.0 | 1.0 | 0 | cluster\_plot | 37 |
| 897 | GO:0003905 | alkylbase DNA N-glycosylase activity | molecular\_function | 0.0 | 1.0 | 1.0 | 0 | cluster\_plot | 10 |
| 898 | GO:0016421 | CoA carboxylase activity | molecular\_function | 0.0 | 1.0 | 1.0 | 0 | cluster\_plot | 7 |
| 899 | GO:0042357 | thiamine diphosphate metabolic process | biological\_process | 0.0 | 1.0 | 1.0 | 0 | cluster\_plot | 2 |
| 900 | GO:0005875 | microtubule associated complex | cellular\_component | 0.0 | 1.0 | 1.0 | 0 | cluster\_plot | 13 |
| 901 | GO:0005542 | folic acid binding | molecular\_function | 0.0 | 1.0 | 1.0 | 0 | cluster\_plot | 4 |
| 902 | GO:0040007 | growth | biological\_process | 0.0 | 1.0 | 1.0 | 0 | cluster\_plot | 17 |
| 903 | GO:0009226 | nucleotide-sugar biosynthetic process | biological\_process | 0.0 | 1.0 | 1.0 | 0 | cluster\_plot | 2 |
| 904 | GO:0032268 | regulation of cellular protein metabolic process | biological\_process | 0.0 | 1.0 | 1.0 | 0 | cluster\_plot | 26 |
| 905 | GO:0006231 | dTMP biosynthetic process | biological\_process | 0.0 | 1.0 | 1.0 | 0 | cluster\_plot | 1 |
| 906 | GO:0030288 | outer membrane-bounded periplasmic space | cellular\_component | 0.0 | 1.0 | 1.0 | 0 | cluster\_plot | 2 |
| 907 | GO:0016836 | hydro-lyase activity | molecular\_function | 0.0 | 1.0 | 1.0 | 0 | cluster\_plot | 54 |
| 908 | GO:0003938 | IMP dehydrogenase activity | molecular\_function | 0.0 | 1.0 | 1.0 | 0 | cluster\_plot | 3 |
| 909 | GO:0019201 | nucleotide kinase activity | molecular\_function | 0.0 | 1.0 | 1.0 | 0 | cluster\_plot | 28 |
| 910 | GO:0070001 | aspartic-type peptidase activity | molecular\_function | 0.0 | 1.0 | 1.0 | 0 | cluster\_plot | 25 |
| 911 | GO:0004520 | endodeoxyribonuclease activity | molecular\_function | 0.0 | 1.0 | 1.0 | 0 | cluster\_plot | 9 |
| 912 | GO:0009123 | nucleoside monophosphate metabolic process | biological\_process | 0.0 | 1.0 | 1.0 | 0 | cluster\_plot | 85 |
| 913 | GO:0071173 | spindle assembly checkpoint | biological\_process | 0.0 | 1.0 | 1.0 | 0 | cluster\_plot | 2 |
| 914 | GO:0003690 | double-stranded DNA binding | molecular\_function | 0.0 | 1.0 | 1.0 | 0 | cluster\_plot | 19 |
| 915 | GO:0016860 | intramolecular oxidoreductase activity | molecular\_function | 0.0 | 1.0 | 1.0 | 0 | cluster\_plot | 32 |
| 916 | GO:0008199 | ferric iron binding | molecular\_function | 0.0 | 1.0 | 1.0 | 0 | cluster\_plot | 9 |
| 917 | GO:0018202 | peptidyl-histidine modification | biological\_process | 0.0 | 1.0 | 1.0 | 0 | cluster\_plot | 4 |
| 918 | GO:0006222 | UMP biosynthetic process | biological\_process | 0.0 | 1.0 | 1.0 | 0 | cluster\_plot | 5 |
| 919 | GO:0019941 | modification-dependent protein catabolic process | biological\_process | 0.0 | 1.0 | 1.0 | 0 | cluster\_plot | 157 |
| 920 | GO:1901069 | guanosine-containing compound catabolic process | biological\_process | 0.0 | 1.0 | 1.0 | 0 | cluster\_plot | 22 |
| 921 | GO:0004609 | phosphatidylserine decarboxylase activity | molecular\_function | 0.0 | 1.0 | 1.0 | 0 | cluster\_plot | 1 |
| 922 | GO:1902680 | positive regulation of RNA biosynthetic process | biological\_process | 0.0 | 1.0 | 1.0 | 0 | cluster\_plot | 4 |
| 923 | GO:0045936 | negative regulation of phosphate metabolic process | biological\_process | 0.0 | 1.0 | 1.0 | 0 | cluster\_plot | 3 |
| 924 | GO:0051603 | proteolysis involved in cellular protein catabolic process | biological\_process | 0.0 | 1.0 | 1.0 | 0 | cluster\_plot | 183 |
| 925 | GO:0008017 | microtubule binding | molecular\_function | 0.0 | 1.0 | 1.0 | 0 | cluster\_plot | 116 |
| 926 | GO:0019419 | sulfate reduction | biological\_process | 0.0 | 1.0 | 1.0 | 0 | cluster\_plot | 3 |
| 927 | GO:0045240 | dihydrolipoyl dehydrogenase complex | cellular\_component | 0.0 | 1.0 | 1.0 | 0 | cluster\_plot | 3 |
| 928 | GO:0015930 | glutamate synthase activity | molecular\_function | 0.0 | 1.0 | 1.0 | 0 | cluster\_plot | 4 |
| 929 | GO:0009045 | xylose isomerase activity | molecular\_function | 0.0 | 1.0 | 1.0 | 0 | cluster\_plot | 2 |
| 930 | GO:0008734 | L-aspartate oxidase activity | molecular\_function | 0.0 | 1.0 | 1.0 | 0 | cluster\_plot | 2 |
| 931 | GO:0004000 | adenosine deaminase activity | molecular\_function | 0.0 | 1.0 | 1.0 | 0 | cluster\_plot | 1 |
| 932 | GO:0009085 | lysine biosynthetic process | biological\_process | 0.0 | 1.0 | 1.0 | 0 | cluster\_plot | 13 |
| 933 | GO:0009176 | pyrimidine deoxyribonucleoside monophosphate metabolic process | biological\_process | 0.0 | 1.0 | 1.0 | 0 | cluster\_plot | 1 |
| 934 | GO:0030983 | mismatched DNA binding | molecular\_function | 0.0 | 1.0 | 1.0 | 0 | cluster\_plot | 19 |
| 935 | GO:0003872 | 6-phosphofructokinase activity | molecular\_function | 0.0 | 1.0 | 1.0 | 0 | cluster\_plot | 15 |
| 936 | GO:0004497 | monooxygenase activity | molecular\_function | 0.0 | 1.0 | 1.0 | 0 | cluster\_plot | 63 |
| 937 | GO:0008835 | diaminohydroxyphosphoribosylaminopyrimidine deaminase activity | molecular\_function | 0.0 | 1.0 | 1.0 | 0 | cluster\_plot | 2 |
| 938 | GO:0009119 | ribonucleoside metabolic process | biological\_process | 0.0 | 1.0 | 1.0 | 0 | cluster\_plot | 120 |
| 939 | GO:0044423 | virion part | cellular\_component | 0.0 | 1.0 | 1.0 | 0 | cluster\_plot | 1 |
| 940 | GO:0006497 | protein lipidation | biological\_process | 0.0 | 1.0 | 1.0 | 0 | cluster\_plot | 18 |
| 941 | GO:0045859 | regulation of protein kinase activity | biological\_process | 0.0 | 1.0 | 1.0 | 0 | cluster\_plot | 11 |
| 942 | GO:0015095 | magnesium ion transmembrane transporter activity | molecular\_function | 0.0 | 1.0 | 1.0 | 0 | cluster\_plot | 16 |
| 943 | GO:0015748 | organophosphate ester transport | biological\_process | 0.0 | 1.0 | 1.0 | 0 | cluster\_plot | 26 |
| 944 | GO:0008081 | phosphoric diester hydrolase activity | molecular\_function | 0.0 | 1.0 | 1.0 | 0 | cluster\_plot | 36 |
| 945 | GO:0043100 | pyrimidine nucleobase salvage | biological\_process | 0.0 | 1.0 | 1.0 | 0 | cluster\_plot | 1 |
| 946 | GO:0008649 | rRNA methyltransferase activity | molecular\_function | 0.0 | 1.0 | 1.0 | 0 | cluster\_plot | 4 |
| 947 | GO:0016892 | endoribonuclease activity, producing 3'-phosphomonoesters | molecular\_function | 0.0 | 1.0 | 1.0 | 0 | cluster\_plot | 13 |
| 948 | GO:0033559 | unsaturated fatty acid metabolic process | biological\_process | 0.0 | 1.0 | 1.0 | 0 | cluster\_plot | 3 |
| 949 | GO:0016681 | oxidoreductase activity, acting on diphenols and related substances as donors, cytochrome as acceptor | molecular\_function | 0.0 | 1.0 | 1.0 | 0 | cluster\_plot | 3 |
| 950 | GO:0016896 | exoribonuclease activity, producing 5'-phosphomonoesters | molecular\_function | 0.0 | 1.0 | 1.0 | 0 | cluster\_plot | 1 |
| 951 | GO:0009125 | nucleoside monophosphate catabolic process | biological\_process | 0.0 | 1.0 | 1.0 | 0 | cluster\_plot | 19 |
| 952 | GO:0030163 | protein catabolic process | biological\_process | 0.0 | 1.0 | 1.0 | 0 | cluster\_plot | 41 |
| 953 | GO:0016307 | phosphatidylinositol phosphate kinase activity | molecular\_function | 0.0 | 1.0 | 1.0 | 0 | cluster\_plot | 20 |
| 954 | GO:0032880 | regulation of protein localization | biological\_process | 0.0 | 1.0 | 1.0 | 0 | cluster\_plot | 2 |
| 955 | GO:0030261 | chromosome condensation | biological\_process | 0.0 | 1.0 | 1.0 | 0 | cluster\_plot | 1 |
| 956 | GO:0004144 | diacylglycerol O-acyltransferase activity | molecular\_function | 0.0 | 1.0 | 1.0 | 0 | cluster\_plot | 10 |
| 957 | GO:0016632 | oxidoreductase activity, acting on the CH-CH group of donors, cytochrome as acceptor | molecular\_function | 0.0 | 1.0 | 1.0 | 0 | cluster\_plot | 2 |
| 958 | GO:0004817 | cysteine-tRNA ligase activity | molecular\_function | 0.0 | 1.0 | 1.0 | 0 | cluster\_plot | 4 |
| 959 | GO:0047632 | agmatine deiminase activity | molecular\_function | 0.0 | 1.0 | 1.0 | 0 | cluster\_plot | 2 |
| 960 | GO:0034023 | 5-(carboxyamino)imidazole ribonucleotide mutase activity | molecular\_function | 0.0 | 1.0 | 1.0 | 0 | cluster\_plot | 2 |
| 961 | GO:0019464 | glycine decarboxylation via glycine cleavage system | biological\_process | 0.0 | 1.0 | 1.0 | 0 | cluster\_plot | 4 |
| 962 | GO:0004003 | ATP-dependent DNA helicase activity | molecular\_function | 0.0 | 1.0 | 1.0 | 0 | cluster\_plot | 11 |
| 963 | GO:0005643 | nuclear pore | cellular\_component | 0.0 | 1.0 | 1.0 | 0 | cluster\_plot | 19 |
| 964 | GO:0008066 | glutamate receptor activity | molecular\_function | 0.0 | 1.0 | 1.0 | 0 | cluster\_plot | 37 |
| 965 | GO:0015301 | anion:anion antiporter activity | molecular\_function | 0.0 | 1.0 | 1.0 | 0 | cluster\_plot | 1 |
| 966 | GO:0004134 | 4-alpha-glucanotransferase activity | molecular\_function | 0.0 | 1.0 | 1.0 | 0 | cluster\_plot | 2 |
| 967 | GO:0009103 | lipopolysaccharide biosynthetic process | biological\_process | 0.0 | 1.0 | 1.0 | 0 | cluster\_plot | 2 |
| 968 | GO:0051998 | protein carboxyl O-methyltransferase activity | molecular\_function | 0.0 | 1.0 | 1.0 | 0 | cluster\_plot | 3 |
| 969 | GO:0006536 | glutamate metabolic process | biological\_process | 0.0 | 1.0 | 1.0 | 0 | cluster\_plot | 14 |
| 970 | GO:0000918 | barrier septum site selection | biological\_process | 0.0 | 1.0 | 1.0 | 0 | cluster\_plot | 3 |
| 971 | GO:0042440 | pigment metabolic process | biological\_process | 0.0 | 1.0 | 1.0 | 0 | cluster\_plot | 57 |
| 972 | GO:0032984 | macromolecular complex disassembly | biological\_process | 0.0 | 1.0 | 1.0 | 0 | cluster\_plot | 13 |
| 973 | GO:0009089 | lysine biosynthetic process via diaminopimelate | biological\_process | 0.0 | 1.0 | 1.0 | 0 | cluster\_plot | 13 |
| 974 | GO:0010333 | terpene synthase activity | molecular\_function | 0.0 | 1.0 | 1.0 | 0 | cluster\_plot | 53 |
| 975 | GO:0003887 | DNA-directed DNA polymerase activity | molecular\_function | 0.0 | 1.0 | 1.0 | 0 | cluster\_plot | 33 |
| 976 | GO:0006621 | protein retention in ER lumen | biological\_process | 0.0 | 1.0 | 1.0 | 0 | cluster\_plot | 11 |
| 977 | GO:0003746 | translation elongation factor activity | molecular\_function | 0.0 | 1.0 | 1.0 | 0 | cluster\_plot | 39 |
| 978 | GO:0031224 | intrinsic component of membrane | cellular\_component | 0.0 | 1.0 | 1.0 | 0 | cluster\_plot | 6 |
| 979 | GO:0009690 | cytokinin metabolic process | biological\_process | 0.0 | 1.0 | 1.0 | 0 | cluster\_plot | 13 |
| 980 | GO:0047800 | cysteamine dioxygenase activity | molecular\_function | 0.0 | 1.0 | 1.0 | 0 | cluster\_plot | 11 |
| 981 | GO:0004379 | glycylpeptide N-tetradecanoyltransferase activity | molecular\_function | 0.0 | 1.0 | 1.0 | 0 | cluster\_plot | 2 |
| 982 | GO:0004629 | phospholipase C activity | molecular\_function | 0.0 | 1.0 | 1.0 | 0 | cluster\_plot | 6 |
| 983 | GO:0042723 | thiamine-containing compound metabolic process | biological\_process | 0.0 | 1.0 | 1.0 | 0 | cluster\_plot | 10 |
| 984 | GO:0006265 | DNA topological change | biological\_process | 0.0 | 1.0 | 1.0 | 0 | cluster\_plot | 17 |
| 985 | GO:0003712 | transcription cofactor activity | molecular\_function | 0.0 | 1.0 | 1.0 | 0 | cluster\_plot | 37 |
| 986 | GO:0019866 | organelle inner membrane | cellular\_component | 0.0 | 1.0 | 1.0 | 0 | cluster\_plot | 17 |
| 987 | GO:0034470 | ncRNA processing | biological\_process | 0.0 | 1.0 | 1.0 | 0 | cluster\_plot | 99 |
| 988 | GO:0004019 | adenylosuccinate synthase activity | molecular\_function | 0.0 | 1.0 | 1.0 | 0 | cluster\_plot | 1 |
| 989 | GO:0016114 | terpenoid biosynthetic process | biological\_process | 0.0 | 1.0 | 1.0 | 0 | cluster\_plot | 19 |
| 990 | GO:0016615 | malate dehydrogenase activity | molecular\_function | 0.0 | 1.0 | 1.0 | 0 | cluster\_plot | 28 |
| 991 | GO:0051172 | negative regulation of nitrogen compound metabolic process | biological\_process | 0.0 | 1.0 | 1.0 | 0 | cluster\_plot | 13 |
| 992 | GO:0047334 | diphosphate-fructose-6-phosphate 1-phosphotransferase activity | molecular\_function | 0.0 | 1.0 | 1.0 | 0 | cluster\_plot | 3 |
| 993 | GO:2000113 | negative regulation of cellular macromolecule biosynthetic process | biological\_process | 0.0 | 1.0 | 1.0 | 0 | cluster\_plot | 20 |
| 994 | GO:0009205 | purine ribonucleoside triphosphate metabolic process | biological\_process | 0.0 | 1.0 | 1.0 | 0 | cluster\_plot | 86 |
| 995 | GO:0033866 | nucleoside bisphosphate biosynthetic process | biological\_process | 0.0 | 1.0 | 1.0 | 0 | cluster\_plot | 2 |
| 996 | GO:0046422 | violaxanthin de-epoxidase activity | molecular\_function | 0.0 | 1.0 | 1.0 | 0 | cluster\_plot | 1 |
| 997 | GO:0030248 | cellulose binding | molecular\_function | 0.0 | 1.0 | 1.0 | 0 | cluster\_plot | 1 |
| 998 | GO:0004322 | ferroxidase activity | molecular\_function | 0.0 | 1.0 | 1.0 | 0 | cluster\_plot | 1 |
| 999 | GO:0015294 | solute:cation symporter activity | molecular\_function | 0.0 | 1.0 | 1.0 | 0 | cluster\_plot | 25 |
| 1000 | GO:0009790 | embryo development | biological\_process | 0.0 | 1.0 | 1.0 | 0 | cluster\_plot | 5 |
| 1001 | GO:0030598 | rRNA N-glycosylase activity | molecular\_function | 0.0 | 1.0 | 1.0 | 0 | cluster\_plot | 8 |
| 1002 | GO:0043628 | ncRNA 3'-end processing | biological\_process | 0.0 | 1.0 | 1.0 | 0 | cluster\_plot | 1 |
| 1003 | GO:0042318 | penicillin biosynthetic process | biological\_process | 0.0 | 1.0 | 1.0 | 0 | cluster\_plot | 1 |
| 1004 | GO:0098599 | palmitoyl hydrolase activity | molecular\_function | 0.0 | 1.0 | 1.0 | 0 | cluster\_plot | 4 |
| 1005 | GO:0031325 | positive regulation of cellular metabolic process | biological\_process | 0.0 | 1.0 | 1.0 | 0 | cluster\_plot | 8 |
| 1006 | GO:0009896 | positive regulation of catabolic process | biological\_process | 0.0 | 1.0 | 1.0 | 0 | cluster\_plot | 1 |
| 1007 | GO:0031324 | negative regulation of cellular metabolic process | biological\_process | 0.0 | 1.0 | 1.0 | 0 | cluster\_plot | 23 |
| 1008 | GO:0004018 | N6-(1,2-dicarboxyethyl)AMP AMP-lyase (fumarate-forming) activity | molecular\_function | 0.0 | 1.0 | 1.0 | 0 | cluster\_plot | 1 |
| 1009 | GO:0016151 | nickel cation binding | molecular\_function | 0.0 | 1.0 | 1.0 | 0 | cluster\_plot | 6 |
| 1010 | GO:0019478 | D-amino acid catabolic process | biological\_process | 0.0 | 1.0 | 1.0 | 0 | cluster\_plot | 1 |
| 1011 | GO:0044093 | positive regulation of molecular function | biological\_process | 0.0 | 1.0 | 1.0 | 0 | cluster\_plot | 2 |
| 1012 | GO:0000149 | SNARE binding | molecular\_function | 0.0 | 1.0 | 1.0 | 0 | cluster\_plot | 2 |
| 1013 | GO:0015276 | ligand-gated ion channel activity | molecular\_function | 0.0 | 1.0 | 1.0 | 0 | cluster\_plot | 37 |
| 1014 | GO:0006772 | thiamine metabolic process | biological\_process | 0.0 | 1.0 | 1.0 | 0 | cluster\_plot | 10 |
| 1015 | GO:0033554 | cellular response to stress | biological\_process | 0.0 | 1.0 | 1.0 | 0 | cluster\_plot | 134 |
| 1016 | GO:0003879 | ATP phosphoribosyltransferase activity | molecular\_function | 0.0 | 1.0 | 1.0 | 0 | cluster\_plot | 3 |
| 1017 | GO:0015851 | nucleobase transport | biological\_process | 0.0 | 1.0 | 1.0 | 0 | cluster\_plot | 1 |
| 1018 | GO:0010817 | regulation of hormone levels | biological\_process | 0.0 | 1.0 | 1.0 | 0 | cluster\_plot | 13 |
| 1019 | GO:0004742 | dihydrolipoyllysine-residue acetyltransferase activity | molecular\_function | 0.0 | 1.0 | 1.0 | 0 | cluster\_plot | 1 |
| 1020 | GO:0046390 | ribose phosphate biosynthetic process | biological\_process | 0.0 | 1.0 | 1.0 | 0 | cluster\_plot | 82 |
| 1021 | GO:0003883 | CTP synthase activity | molecular\_function | 0.0 | 1.0 | 1.0 | 0 | cluster\_plot | 10 |
| 1022 | GO:0051704 | multi-organism process | biological\_process | 0.0 | 1.0 | 1.0 | 0 | cluster\_plot | 2 |
| 1023 | GO:0008033 | tRNA processing | biological\_process | 0.0 | 1.0 | 1.0 | 0 | cluster\_plot | 61 |
| 1024 | GO:0009067 | aspartate family amino acid biosynthetic process | biological\_process | 0.0 | 1.0 | 1.0 | 0 | cluster\_plot | 33 |
| 1025 | GO:0015081 | sodium ion transmembrane transporter activity | molecular\_function | 0.0 | 1.0 | 1.0 | 0 | cluster\_plot | 16 |
| 1026 | GO:0034032 | purine nucleoside bisphosphate metabolic process | biological\_process | 0.0 | 1.0 | 1.0 | 0 | cluster\_plot | 12 |
| 1027 | GO:0033179 | proton-transporting V-type ATPase, V0 domain | cellular\_component | 0.0 | 1.0 | 1.0 | 0 | cluster\_plot | 18 |
| 1028 | GO:0015935 | small ribosomal subunit | cellular\_component | 0.0 | 1.0 | 1.0 | 0 | cluster\_plot | 33 |
| 1029 | GO:0004048 | anthranilate phosphoribosyltransferase activity | molecular\_function | 0.0 | 1.0 | 1.0 | 0 | cluster\_plot | 1 |
| 1030 | GO:0009628 | response to abiotic stimulus | biological\_process | 0.0 | 1.0 | 1.0 | 0 | cluster\_plot | 20 |
| 1031 | GO:0005544 | calcium-dependent phospholipid binding | molecular\_function | 0.0 | 1.0 | 1.0 | 0 | cluster\_plot | 13 |
| 1032 | GO:0004121 | cystathionine beta-lyase activity | molecular\_function | 0.0 | 1.0 | 1.0 | 0 | cluster\_plot | 1 |
| 1033 | GO:0071941 | nitrogen cycle metabolic process | biological\_process | 0.0 | 1.0 | 1.0 | 0 | cluster\_plot | 2 |
| 1034 | GO:0032403 | protein complex binding | molecular\_function | 0.0 | 1.0 | 1.0 | 0 | cluster\_plot | 127 |
| 1035 | GO:0046385 | deoxyribose phosphate biosynthetic process | biological\_process | 0.0 | 1.0 | 1.0 | 0 | cluster\_plot | 3 |
| 1036 | GO:0015018 | galactosylgalactosylxylosylprotein 3-beta-glucuronosyltransferase activity | molecular\_function | 0.0 | 1.0 | 1.0 | 0 | cluster\_plot | 6 |
| 1037 | GO:1902100 | negative regulation of metaphase/anaphase transition of cell cycle | biological\_process | 0.0 | 1.0 | 1.0 | 0 | cluster\_plot | 2 |
| 1038 | GO:0019901 | protein kinase binding | molecular\_function | 0.0 | 1.0 | 1.0 | 0 | cluster\_plot | 11 |
| 1039 | GO:0030811 | regulation of nucleotide catabolic process | biological\_process | 0.0 | 1.0 | 1.0 | 0 | cluster\_plot | 51 |
| 1040 | GO:0016642 | oxidoreductase activity, acting on the CH-NH2 group of donors, disulfide as acceptor | molecular\_function | 0.0 | 1.0 | 1.0 | 0 | cluster\_plot | 3 |
| 1041 | GO:0044550 | secondary metabolite biosynthetic process | biological\_process | 0.0 | 1.0 | 1.0 | 0 | cluster\_plot | 4 |
| 1042 | GO:0015988 | energy coupled proton transmembrane transport, against electrochemical gradient | biological\_process | 0.0 | 1.0 | 1.0 | 0 | cluster\_plot | 38 |
| 1043 | GO:0000079 | regulation of cyclin-dependent protein serine/threonine kinase activity | biological\_process | 0.0 | 1.0 | 1.0 | 0 | cluster\_plot | 11 |
| 1044 | GO:0008135 | translation factor activity, nucleic acid binding | molecular\_function | 0.0 | 1.0 | 1.0 | 0 | cluster\_plot | 100 |
| 1045 | GO:0030272 | 5-formyltetrahydrofolate cyclo-ligase activity | molecular\_function | 0.0 | 1.0 | 1.0 | 0 | cluster\_plot | 3 |
| 1046 | GO:0001932 | regulation of protein phosphorylation | biological\_process | 0.0 | 1.0 | 1.0 | 0 | cluster\_plot | 11 |
| 1047 | GO:0008252 | nucleotidase activity | molecular\_function | 0.0 | 1.0 | 1.0 | 0 | cluster\_plot | 4 |
| 1048 | GO:0006596 | polyamine biosynthetic process | biological\_process | 0.0 | 1.0 | 1.0 | 0 | cluster\_plot | 8 |
| 1049 | GO:0006164 | purine nucleotide biosynthetic process | biological\_process | 0.0 | 1.0 | 1.0 | 0 | cluster\_plot | 89 |
| 1050 | GO:0034708 | methyltransferase complex | cellular\_component | 0.0 | 1.0 | 1.0 | 0 | cluster\_plot | 3 |
| 1051 | GO:0005086 | ARF guanyl-nucleotide exchange factor activity | molecular\_function | 0.0 | 1.0 | 1.0 | 0 | cluster\_plot | 11 |
| 1052 | GO:0033180 | proton-transporting V-type ATPase, V1 domain | cellular\_component | 0.0 | 1.0 | 1.0 | 0 | cluster\_plot | 7 |
| 1053 | GO:0000152 | nuclear ubiquitin ligase complex | cellular\_component | 0.0 | 1.0 | 1.0 | 0 | cluster\_plot | 7 |
| 1054 | GO:0080135 | regulation of cellular response to stress | biological\_process | 0.0 | 1.0 | 1.0 | 0 | cluster\_plot | 2 |
| 1055 | GO:0010629 | negative regulation of gene expression | biological\_process | 0.0 | 1.0 | 1.0 | 0 | cluster\_plot | 19 |
| 1056 | GO:0005099 | Ras GTPase activator activity | molecular\_function | 0.0 | 1.0 | 1.0 | 0 | cluster\_plot | 27 |
| 1057 | GO:0044282 | small molecule catabolic process | biological\_process | 0.0 | 1.0 | 1.0 | 0 | cluster\_plot | 49 |
| 1058 | GO:0016639 | oxidoreductase activity, acting on the CH-NH2 group of donors, NAD or NADP as acceptor | molecular\_function | 0.0 | 1.0 | 1.0 | 0 | cluster\_plot | 3 |
| 1059 | GO:0015296 | anion:cation symporter activity | molecular\_function | 0.0 | 1.0 | 1.0 | 0 | cluster\_plot | 13 |
| 1060 | GO:0008375 | acetylglucosaminyltransferase activity | molecular\_function | 0.0 | 1.0 | 1.0 | 0 | cluster\_plot | 61 |
| 1061 | GO:0048529 | magnesium-protoporphyrin IX monomethyl ester (oxidative) cyclase activity | molecular\_function | 0.0 | 1.0 | 1.0 | 0 | cluster\_plot | 2 |
| 1062 | GO:0008840 | 4-hydroxy-tetrahydrodipicolinate synthase | molecular\_function | 0.0 | 1.0 | 1.0 | 0 | cluster\_plot | 1 |
| 1063 | GO:0016877 | ligase activity, forming carbon-sulfur bonds | molecular\_function | 0.0 | 1.0 | 1.0 | 0 | cluster\_plot | 2 |
| 1064 | GO:0048519 | negative regulation of biological process | biological\_process | 0.0 | 1.0 | 1.0 | 0 | cluster\_plot | 48 |
| 1065 | GO:0000776 | kinetochore | cellular\_component | 0.0 | 1.0 | 1.0 | 0 | cluster\_plot | 3 |
| 1066 | GO:0046496 | nicotinamide nucleotide metabolic process | biological\_process | 0.0 | 1.0 | 1.0 | 0 | cluster\_plot | 31 |
| 1067 | GO:0018738 | S-formylglutathione hydrolase activity | molecular\_function | 0.0 | 1.0 | 1.0 | 0 | cluster\_plot | 2 |
| 1068 | GO:0010608 | posttranscriptional regulation of gene expression | biological\_process | 0.0 | 1.0 | 1.0 | 0 | cluster\_plot | 15 |
| 1069 | GO:1901988 | negative regulation of cell cycle phase transition | biological\_process | 0.0 | 1.0 | 1.0 | 0 | cluster\_plot | 2 |
| 1070 | GO:0016829 | lyase activity | molecular\_function | 0.0 | 1.0 | 1.0 | 0 | cluster\_plot | 267 |
| 1071 | GO:0006777 | Mo-molybdopterin cofactor biosynthetic process | biological\_process | 0.0 | 1.0 | 1.0 | 0 | cluster\_plot | 10 |
| 1072 | GO:0070567 | cytidylyltransferase activity | molecular\_function | 0.0 | 1.0 | 1.0 | 0 | cluster\_plot | 2 |
| 1073 | GO:0006612 | protein targeting to membrane | biological\_process | 0.0 | 1.0 | 1.0 | 0 | cluster\_plot | 17 |
| 1074 | GO:0003868 | 4-hydroxyphenylpyruvate dioxygenase activity | molecular\_function | 0.0 | 1.0 | 1.0 | 0 | cluster\_plot | 2 |
| 1075 | GO:0043096 | purine nucleobase salvage | biological\_process | 0.0 | 1.0 | 1.0 | 0 | cluster\_plot | 8 |
| 1076 | GO:0030417 | nicotianamine metabolic process | biological\_process | 0.0 | 1.0 | 1.0 | 0 | cluster\_plot | 2 |
| 1077 | GO:0015217 | ADP transmembrane transporter activity | molecular\_function | 0.0 | 1.0 | 1.0 | 0 | cluster\_plot | 1 |
| 1078 | GO:0000910 | cytokinesis | biological\_process | 0.0 | 1.0 | 1.0 | 0 | cluster\_plot | 11 |
| 1079 | GO:1901570 | fatty acid derivative biosynthetic process | biological\_process | 0.0 | 1.0 | 1.0 | 0 | cluster\_plot | 3 |
| 1080 | GO:0008312 | 7S RNA binding | molecular\_function | 0.0 | 1.0 | 1.0 | 0 | cluster\_plot | 13 |
| 1081 | GO:0016796 | exonuclease activity, active with either ribo- or deoxyribonucleic acids and producing 5'-phosphomonoesters | molecular\_function | 0.0 | 1.0 | 1.0 | 0 | cluster\_plot | 3 |
| 1082 | GO:0005451 | monovalent cation:proton antiporter activity | molecular\_function | 0.0 | 1.0 | 1.0 | 0 | cluster\_plot | 5 |
| 1083 | GO:0008236 | serine-type peptidase activity | molecular\_function | 0.0 | 1.0 | 1.0 | 0 | cluster\_plot | 212 |
| 1084 | GO:0008676 | 3-deoxy-8-phosphooctulonate synthase activity | molecular\_function | 0.0 | 1.0 | 1.0 | 0 | cluster\_plot | 2 |
| 1085 | GO:0004149 | dihydrolipoyllysine-residue succinyltransferase activity | molecular\_function | 0.0 | 1.0 | 1.0 | 0 | cluster\_plot | 3 |
| 1086 | GO:0006551 | leucine metabolic process | biological\_process | 0.0 | 1.0 | 1.0 | 0 | cluster\_plot | 4 |
| 1087 | GO:0018205 | peptidyl-lysine modification | biological\_process | 0.0 | 1.0 | 1.0 | 0 | cluster\_plot | 5 |
| 1088 | GO:0009235 | cobalamin metabolic process | biological\_process | 0.0 | 1.0 | 1.0 | 0 | cluster\_plot | 1 |
| 1089 | GO:0051049 | regulation of transport | biological\_process | 0.0 | 1.0 | 1.0 | 0 | cluster\_plot | 2 |
| 1090 | GO:0006401 | RNA catabolic process | biological\_process | 0.0 | 1.0 | 1.0 | 0 | cluster\_plot | 6 |
| 1091 | GO:0004077 | biotin-[acetyl-CoA-carboxylase] ligase activity | molecular\_function | 0.0 | 1.0 | 1.0 | 0 | cluster\_plot | 3 |
| 1092 | GO:0009265 | 2'-deoxyribonucleotide biosynthetic process | biological\_process | 0.0 | 1.0 | 1.0 | 0 | cluster\_plot | 3 |
| 1093 | GO:0044255 | cellular lipid metabolic process | biological\_process | 0.0 | 1.0 | 1.0 | 0 | cluster\_plot | 292 |
| 1094 | GO:0017070 | U6 snRNA binding | molecular\_function | 0.0 | 1.0 | 1.0 | 0 | cluster\_plot | 2 |
| 1095 | GO:0015099 | nickel cation transmembrane transporter activity | molecular\_function | 0.0 | 1.0 | 1.0 | 0 | cluster\_plot | 2 |
| 1096 | GO:0052736 | beta-glucanase activity | molecular\_function | 0.0 | 1.0 | 1.0 | 0 | cluster\_plot | 10 |
| 1097 | GO:0016781 | phosphotransferase activity, paired acceptors | molecular\_function | 0.0 | 1.0 | 1.0 | 0 | cluster\_plot | 6 |
| 1098 | GO:0003841 | 1-acylglycerol-3-phosphate O-acyltransferase activity | molecular\_function | 0.0 | 1.0 | 1.0 | 0 | cluster\_plot | 2 |
| 1099 | GO:0055082 | cellular chemical homeostasis | biological\_process | 0.0 | 1.0 | 1.0 | 0 | cluster\_plot | 6 |
| 1100 | GO:0045263 | proton-transporting ATP synthase complex, coupling factor F(o) | cellular\_component | 0.0 | 1.0 | 1.0 | 0 | cluster\_plot | 11 |
| 1101 | GO:0017144 | drug metabolic process | biological\_process | 0.0 | 1.0 | 1.0 | 0 | cluster\_plot | 1 |
| 1102 | GO:0043207 | response to external biotic stimulus | biological\_process | 0.0 | 1.0 | 1.0 | 0 | cluster\_plot | 1 |
| 1103 | GO:0006007 | glucose catabolic process | biological\_process | 0.0 | 1.0 | 1.0 | 0 | cluster\_plot | 17 |
| 1104 | GO:0044434 | chloroplast part | cellular\_component | 0.0 | 1.0 | 1.0 | 0 | cluster\_plot | 2 |
| 1105 | GO:0004512 | inositol-3-phosphate synthase activity | molecular\_function | 0.0 | 1.0 | 1.0 | 0 | cluster\_plot | 6 |
| 1106 | GO:0044391 | ribosomal subunit | cellular\_component | 0.0 | 1.0 | 1.0 | 0 | cluster\_plot | 65 |
| 1107 | GO:0008115 | sarcosine oxidase activity | molecular\_function | 0.0 | 1.0 | 1.0 | 0 | cluster\_plot | 3 |
| 1108 | GO:0046116 | queuosine metabolic process | biological\_process | 0.0 | 1.0 | 1.0 | 0 | cluster\_plot | 3 |
| 1109 | GO:0004634 | phosphopyruvate hydratase activity | molecular\_function | 0.0 | 1.0 | 1.0 | 0 | cluster\_plot | 7 |
| 1110 | GO:0016265 | death | biological\_process | 0.0 | 1.0 | 1.0 | 0 | cluster\_plot | 1 |
| 1111 | GO:1900542 | regulation of purine nucleotide metabolic process | biological\_process | 0.0 | 1.0 | 1.0 | 0 | cluster\_plot | 51 |
| 1112 | GO:0072488 | ammonium transmembrane transport | biological\_process | 0.0 | 1.0 | 1.0 | 0 | cluster\_plot | 11 |
| 1113 | GO:0050790 | regulation of catalytic activity | biological\_process | 0.0 | 1.0 | 1.0 | 0 | cluster\_plot | 137 |
| 1114 | GO:0071496 | cellular response to external stimulus | biological\_process | 0.0 | 1.0 | 1.0 | 0 | cluster\_plot | 7 |
| 1115 | GO:0070461 | SAGA-type complex | cellular\_component | 0.0 | 1.0 | 1.0 | 0 | cluster\_plot | 12 |
| 1116 | GO:0004602 | glutathione peroxidase activity | molecular\_function | 0.0 | 1.0 | 1.0 | 0 | cluster\_plot | 12 |
| 1117 | GO:0090407 | organophosphate biosynthetic process | biological\_process | 0.0 | 1.0 | 1.0 | 0 | cluster\_plot | 195 |
| 1118 | GO:0016864 | intramolecular oxidoreductase activity, transposing S-S bonds | molecular\_function | 0.0 | 1.0 | 1.0 | 0 | cluster\_plot | 1 |
| 1119 | GO:0009654 | photosystem II oxygen evolving complex | cellular\_component | 0.0 | 1.0 | 1.0 | 0 | cluster\_plot | 33 |
| 1120 | GO:0006333 | chromatin assembly or disassembly | biological\_process | 0.0 | 1.0 | 1.0 | 0 | cluster\_plot | 5 |
| 1121 | GO:0048280 | vesicle fusion with Golgi apparatus | biological\_process | 0.0 | 1.0 | 1.0 | 0 | cluster\_plot | 2 |
| 1122 | GO:0004073 | aspartate-semialdehyde dehydrogenase activity | molecular\_function | 0.0 | 1.0 | 1.0 | 0 | cluster\_plot | 1 |
| 1123 | GO:0007005 | mitochondrion organization | biological\_process | 0.0 | 1.0 | 1.0 | 0 | cluster\_plot | 20 |
| 1124 | GO:0005886 | plasma membrane | cellular\_component | 0.0 | 1.0 | 1.0 | 0 | cluster\_plot | 5 |
| 1125 | GO:0016149 | translation release factor activity, codon specific | molecular\_function | 0.0 | 1.0 | 1.0 | 0 | cluster\_plot | 12 |
| 1126 | GO:0051002 | ligase activity, forming nitrogen-metal bonds | molecular\_function | 0.0 | 1.0 | 1.0 | 0 | cluster\_plot | 6 |
| 1127 | GO:0006223 | uracil salvage | biological\_process | 0.0 | 1.0 | 1.0 | 0 | cluster\_plot | 1 |
| 1128 | GO:0016844 | strictosidine synthase activity | molecular\_function | 0.0 | 1.0 | 1.0 | 0 | cluster\_plot | 13 |
| 1129 | GO:0006357 | regulation of transcription from RNA polymerase II promoter | biological\_process | 0.0 | 1.0 | 1.0 | 0 | cluster\_plot | 20 |
| 1130 | GO:0004641 | phosphoribosylformylglycinamidine cyclo-ligase activity | molecular\_function | 0.0 | 1.0 | 1.0 | 0 | cluster\_plot | 3 |
| 1131 | GO:0015038 | glutathione disulfide oxidoreductase activity | molecular\_function | 0.0 | 1.0 | 1.0 | 0 | cluster\_plot | 2 |
| 1132 | GO:1901981 | phosphatidylinositol phosphate binding | molecular\_function | 0.0 | 1.0 | 1.0 | 0 | cluster\_plot | 1 |
| 1133 | GO:0010921 | regulation of phosphatase activity | biological\_process | 0.0 | 1.0 | 1.0 | 0 | cluster\_plot | 1 |
| 1134 | GO:0080019 | fatty-acyl-CoA reductase (alcohol-forming) activity | molecular\_function | 0.0 | 1.0 | 1.0 | 0 | cluster\_plot | 7 |
| 1135 | GO:0000154 | rRNA modification | biological\_process | 0.0 | 1.0 | 1.0 | 0 | cluster\_plot | 4 |
| 1136 | GO:0006633 | fatty acid biosynthetic process | biological\_process | 0.0 | 1.0 | 1.0 | 0 | cluster\_plot | 123 |
| 1137 | GO:0022803 | passive transmembrane transporter activity | molecular\_function | 0.0 | 1.0 | 1.0 | 0 | cluster\_plot | 76 |
| 1138 | GO:0009173 | pyrimidine ribonucleoside monophosphate metabolic process | biological\_process | 0.0 | 1.0 | 1.0 | 0 | cluster\_plot | 5 |
| 1139 | GO:0009260 | ribonucleotide biosynthetic process | biological\_process | 0.0 | 1.0 | 1.0 | 0 | cluster\_plot | 82 |
| 1140 | GO:0045116 | protein neddylation | biological\_process | 0.0 | 1.0 | 1.0 | 0 | cluster\_plot | 2 |
| 1141 | GO:0004794 | L-threonine ammonia-lyase activity | molecular\_function | 0.0 | 1.0 | 1.0 | 0 | cluster\_plot | 2 |
| 1142 | GO:0016775 | phosphotransferase activity, nitrogenous group as acceptor | molecular\_function | 0.0 | 1.0 | 1.0 | 0 | cluster\_plot | 25 |
| 1143 | GO:0016838 | carbon-oxygen lyase activity, acting on phosphates | molecular\_function | 0.0 | 1.0 | 1.0 | 0 | cluster\_plot | 59 |
| 1144 | GO:0003848 | 2-amino-4-hydroxy-6-hydroxymethyldihydropteridine diphosphokinase activity | molecular\_function | 0.0 | 1.0 | 1.0 | 0 | cluster\_plot | 2 |
| 1145 | GO:0009130 | pyrimidine nucleoside monophosphate biosynthetic process | biological\_process | 0.0 | 1.0 | 1.0 | 0 | cluster\_plot | 6 |
| 1146 | GO:0006220 | pyrimidine nucleotide metabolic process | biological\_process | 0.0 | 1.0 | 1.0 | 0 | cluster\_plot | 28 |
| 1147 | GO:0004435 | phosphatidylinositol phospholipase C activity | molecular\_function | 0.0 | 1.0 | 1.0 | 0 | cluster\_plot | 6 |
| 1148 | GO:0008097 | 5S rRNA binding | molecular\_function | 0.0 | 1.0 | 1.0 | 0 | cluster\_plot | 2 |
| 1149 | GO:0006904 | vesicle docking involved in exocytosis | biological\_process | 0.0 | 1.0 | 1.0 | 0 | cluster\_plot | 13 |
| 1150 | GO:0055086 | nucleobase-containing small molecule metabolic process | biological\_process | 0.0 | 1.0 | 1.0 | 0 | cluster\_plot | 262 |
| 1151 | GO:0005496 | steroid binding | molecular\_function | 0.0 | 1.0 | 1.0 | 0 | cluster\_plot | 3 |
| 1152 | GO:0044242 | cellular lipid catabolic process | biological\_process | 0.0 | 1.0 | 1.0 | 0 | cluster\_plot | 8 |
| 1153 | GO:0007049 | cell cycle | biological\_process | 0.0 | 1.0 | 1.0 | 0 | cluster\_plot | 13 |
| 1154 | GO:1901070 | guanosine-containing compound biosynthetic process | biological\_process | 0.0 | 1.0 | 1.0 | 0 | cluster\_plot | 11 |
| 1155 | GO:0010498 | proteasomal protein catabolic process | biological\_process | 0.0 | 1.0 | 1.0 | 0 | cluster\_plot | 8 |
| 1156 | GO:0043666 | regulation of phosphoprotein phosphatase activity | biological\_process | 0.0 | 1.0 | 1.0 | 0 | cluster\_plot | 1 |
| 1157 | GO:0009233 | menaquinone metabolic process | biological\_process | 0.0 | 1.0 | 1.0 | 0 | cluster\_plot | 1 |
| 1158 | GO:0016071 | mRNA metabolic process | biological\_process | 0.0 | 1.0 | 1.0 | 0 | cluster\_plot | 54 |
| 1159 | GO:1901659 | glycosyl compound biosynthetic process | biological\_process | 0.0 | 1.0 | 1.0 | 0 | cluster\_plot | 80 |
| 1160 | GO:0016072 | rRNA metabolic process | biological\_process | 0.0 | 1.0 | 1.0 | 0 | cluster\_plot | 40 |
| 1161 | GO:0009432 | SOS response | biological\_process | 0.0 | 1.0 | 1.0 | 0 | cluster\_plot | 5 |
| 1162 | GO:0005742 | mitochondrial outer membrane translocase complex | cellular\_component | 0.0 | 1.0 | 1.0 | 0 | cluster\_plot | 12 |
| 1163 | GO:0016888 | endodeoxyribonuclease activity, producing 5'-phosphomonoesters | molecular\_function | 0.0 | 1.0 | 1.0 | 0 | cluster\_plot | 6 |
| 1164 | GO:0033121 | regulation of purine nucleotide catabolic process | biological\_process | 0.0 | 1.0 | 1.0 | 0 | cluster\_plot | 51 |
| 1165 | GO:0042546 | cell wall biogenesis | biological\_process | 0.0 | 1.0 | 1.0 | 0 | cluster\_plot | 2 |
| 1166 | GO:0001671 | ATPase activator activity | molecular\_function | 0.0 | 1.0 | 1.0 | 0 | cluster\_plot | 2 |
| 1167 | GO:0006631 | fatty acid metabolic process | biological\_process | 0.0 | 1.0 | 1.0 | 0 | cluster\_plot | 139 |
| 1168 | GO:0006189 | 'de novo' IMP biosynthetic process | biological\_process | 0.0 | 1.0 | 1.0 | 0 | cluster\_plot | 11 |
| 1169 | GO:0006233 | dTDP biosynthetic process | biological\_process | 0.0 | 1.0 | 1.0 | 0 | cluster\_plot | 2 |
| 1170 | GO:0098589 | membrane region | cellular\_component | 0.0 | 1.0 | 1.0 | 0 | cluster\_plot | 18 |
| 1171 | GO:0016851 | magnesium chelatase activity | molecular\_function | 0.0 | 1.0 | 1.0 | 0 | cluster\_plot | 6 |
| 1172 | GO:0008237 | metallopeptidase activity | molecular\_function | 0.0 | 1.0 | 1.0 | 0 | cluster\_plot | 76 |
| 1173 | GO:0031225 | anchored component of membrane | cellular\_component | 0.0 | 1.0 | 1.0 | 0 | cluster\_plot | 19 |
| 1174 | GO:0004427 | inorganic diphosphatase activity | molecular\_function | 0.0 | 1.0 | 1.0 | 0 | cluster\_plot | 21 |
| 1175 | GO:0043102 | amino acid salvage | biological\_process | 0.0 | 1.0 | 1.0 | 0 | cluster\_plot | 2 |
| 1176 | GO:0008146 | sulfotransferase activity | molecular\_function | 0.0 | 1.0 | 1.0 | 0 | cluster\_plot | 8 |
| 1177 | GO:0019357 | nicotinate nucleotide biosynthetic process | biological\_process | 0.0 | 1.0 | 1.0 | 0 | cluster\_plot | 2 |
| 1178 | GO:0042594 | response to starvation | biological\_process | 0.0 | 1.0 | 1.0 | 0 | cluster\_plot | 2 |
| 1179 | GO:0033588 | Elongator holoenzyme complex | cellular\_component | 0.0 | 1.0 | 1.0 | 0 | cluster\_plot | 1 |
| 1180 | GO:0000784 | nuclear chromosome, telomeric region | cellular\_component | 0.0 | 1.0 | 1.0 | 0 | cluster\_plot | 1 |
| 1181 | GO:0004749 | ribose phosphate diphosphokinase activity | molecular\_function | 0.0 | 1.0 | 1.0 | 0 | cluster\_plot | 3 |
| 1182 | GO:0006555 | methionine metabolic process | biological\_process | 0.0 | 1.0 | 1.0 | 0 | cluster\_plot | 12 |
| 1183 | GO:0042586 | peptide deformylase activity | molecular\_function | 0.0 | 1.0 | 1.0 | 0 | cluster\_plot | 4 |
| 1184 | GO:0006979 | response to oxidative stress | biological\_process | 0.0 | 1.0 | 1.0 | 0 | cluster\_plot | 128 |
| 1185 | GO:0045735 | nutrient reservoir activity | molecular\_function | 0.0 | 1.0 | 1.0 | 0 | cluster\_plot | 56 |
| 1186 | GO:0015986 | ATP synthesis coupled proton transport | biological\_process | 0.0 | 1.0 | 1.0 | 0 | cluster\_plot | 35 |
| 1187 | GO:0006661 | phosphatidylinositol biosynthetic process | biological\_process | 0.0 | 1.0 | 1.0 | 0 | cluster\_plot | 18 |
| 1188 | GO:0015675 | nickel cation transport | biological\_process | 0.0 | 1.0 | 1.0 | 0 | cluster\_plot | 2 |
| 1189 | GO:0003987 | acetate-CoA ligase activity | molecular\_function | 0.0 | 1.0 | 1.0 | 0 | cluster\_plot | 2 |
| 1190 | GO:0016671 | oxidoreductase activity, acting on a sulfur group of donors, disulfide as acceptor | molecular\_function | 0.0 | 1.0 | 1.0 | 0 | cluster\_plot | 15 |
| 1191 | GO:0010638 | positive regulation of organelle organization | biological\_process | 0.0 | 1.0 | 1.0 | 0 | cluster\_plot | 4 |
| 1192 | GO:0045786 | negative regulation of cell cycle | biological\_process | 0.0 | 1.0 | 1.0 | 0 | cluster\_plot | 10 |
| 1193 | GO:0072524 | pyridine-containing compound metabolic process | biological\_process | 0.0 | 1.0 | 1.0 | 0 | cluster\_plot | 39 |
| 1194 | GO:0000781 | chromosome, telomeric region | cellular\_component | 0.0 | 1.0 | 1.0 | 0 | cluster\_plot | 1 |
| 1195 | GO:0016670 | oxidoreductase activity, acting on a sulfur group of donors, oxygen as acceptor | molecular\_function | 0.0 | 1.0 | 1.0 | 0 | cluster\_plot | 5 |
| 1196 | GO:0004478 | methionine adenosyltransferase activity | molecular\_function | 0.0 | 1.0 | 1.0 | 0 | cluster\_plot | 8 |
| 1197 | GO:0030870 | Mre11 complex | cellular\_component | 0.0 | 1.0 | 1.0 | 0 | cluster\_plot | 1 |
| 1198 | GO:0006165 | nucleoside diphosphate phosphorylation | biological\_process | 0.0 | 1.0 | 1.0 | 0 | cluster\_plot | 7 |
| 1199 | GO:0016780 | phosphotransferase activity, for other substituted phosphate groups | molecular\_function | 0.0 | 1.0 | 1.0 | 0 | cluster\_plot | 16 |
| 1200 | GO:0009220 | pyrimidine ribonucleotide biosynthetic process | biological\_process | 0.0 | 1.0 | 1.0 | 0 | cluster\_plot | 12 |
| 1201 | GO:0048278 | vesicle docking | biological\_process | 0.0 | 1.0 | 1.0 | 0 | cluster\_plot | 17 |
| 1202 | GO:0033615 | mitochondrial proton-transporting ATP synthase complex assembly | biological\_process | 0.0 | 1.0 | 1.0 | 0 | cluster\_plot | 2 |
| 1203 | GO:0009975 | cyclase activity | molecular\_function | 0.0 | 1.0 | 1.0 | 0 | cluster\_plot | 3 |
| 1204 | GO:0000155 | phosphorelay sensor kinase activity | molecular\_function | 0.0 | 1.0 | 1.0 | 0 | cluster\_plot | 25 |
| 1205 | GO:0008446 | GDP-mannose 4,6-dehydratase activity | molecular\_function | 0.0 | 1.0 | 1.0 | 0 | cluster\_plot | 1 |
| 1206 | GO:0004549 | tRNA-specific ribonuclease activity | molecular\_function | 0.0 | 1.0 | 1.0 | 0 | cluster\_plot | 3 |
| 1207 | GO:0000077 | DNA damage checkpoint | biological\_process | 0.0 | 1.0 | 1.0 | 0 | cluster\_plot | 2 |
| 1208 | GO:0032269 | negative regulation of cellular protein metabolic process | biological\_process | 0.0 | 1.0 | 1.0 | 0 | cluster\_plot | 10 |
| 1209 | GO:0016861 | intramolecular oxidoreductase activity, interconverting aldoses and ketoses | molecular\_function | 0.0 | 1.0 | 1.0 | 0 | cluster\_plot | 26 |
| 1210 | GO:0017025 | TBP-class protein binding | molecular\_function | 0.0 | 1.0 | 1.0 | 0 | cluster\_plot | 10 |
| 1211 | GO:0008080 | N-acetyltransferase activity | molecular\_function | 0.0 | 1.0 | 1.0 | 0 | cluster\_plot | 56 |
| 1212 | GO:0009207 | purine ribonucleoside triphosphate catabolic process | biological\_process | 0.0 | 1.0 | 1.0 | 0 | cluster\_plot | 41 |
| 1213 | GO:0019887 | protein kinase regulator activity | molecular\_function | 0.0 | 1.0 | 1.0 | 0 | cluster\_plot | 17 |
| 1214 | GO:0008028 | monocarboxylic acid transmembrane transporter activity | molecular\_function | 0.0 | 1.0 | 1.0 | 0 | cluster\_plot | 11 |
| 1215 | GO:0031968 | organelle outer membrane | cellular\_component | 0.0 | 1.0 | 1.0 | 0 | cluster\_plot | 13 |
| 1216 | GO:0046923 | ER retention sequence binding | molecular\_function | 0.0 | 1.0 | 1.0 | 0 | cluster\_plot | 11 |
| 1217 | GO:0006511 | ubiquitin-dependent protein catabolic process | biological\_process | 0.0 | 1.0 | 1.0 | 0 | cluster\_plot | 157 |
| 1218 | GO:0005244 | voltage-gated ion channel activity | molecular\_function | 0.0 | 1.0 | 1.0 | 0 | cluster\_plot | 13 |
| 1219 | GO:0030654 | beta-lactam antibiotic biosynthetic process | biological\_process | 0.0 | 1.0 | 1.0 | 0 | cluster\_plot | 1 |
| 1220 | GO:0005853 | eukaryotic translation elongation factor 1 complex | cellular\_component | 0.0 | 1.0 | 1.0 | 0 | cluster\_plot | 7 |
| 1221 | GO:0005834 | heterotrimeric G-protein complex | cellular\_component | 0.0 | 1.0 | 1.0 | 0 | cluster\_plot | 7 |
| 1222 | GO:0032506 | cytokinetic process | biological\_process | 0.0 | 1.0 | 1.0 | 0 | cluster\_plot | 6 |
| 1223 | GO:0008441 | 3'(2'),5'-bisphosphate nucleotidase activity | molecular\_function | 0.0 | 1.0 | 1.0 | 0 | cluster\_plot | 4 |
| 1224 | GO:0033218 | amide binding | molecular\_function | 0.0 | 1.0 | 1.0 | 0 | cluster\_plot | 12 |
| 1225 | GO:0004823 | leucine-tRNA ligase activity | molecular\_function | 0.0 | 1.0 | 1.0 | 0 | cluster\_plot | 4 |
| 1226 | GO:0005388 | calcium-transporting ATPase activity | molecular\_function | 0.0 | 1.0 | 1.0 | 0 | cluster\_plot | 26 |
| 1227 | GO:0008643 | carbohydrate transport | biological\_process | 0.0 | 1.0 | 1.0 | 0 | cluster\_plot | 18 |
| 1228 | GO:0043413 | macromolecule glycosylation | biological\_process | 0.0 | 1.0 | 1.0 | 0 | cluster\_plot | 61 |
| 1229 | GO:0005750 | mitochondrial respiratory chain complex III | cellular\_component | 0.0 | 1.0 | 1.0 | 0 | cluster\_plot | 5 |
| 1230 | GO:0042176 | regulation of protein catabolic process | biological\_process | 0.0 | 1.0 | 1.0 | 0 | cluster\_plot | 2 |
| 1231 | GO:0052861 | glucan endo-1,3-beta-glucanase activity, C-3 substituted reducing group | molecular\_function | 0.0 | 1.0 | 1.0 | 0 | cluster\_plot | 10 |
| 1232 | GO:0006561 | proline biosynthetic process | biological\_process | 0.0 | 1.0 | 1.0 | 0 | cluster\_plot | 6 |
| 1233 | GO:0007018 | microtubule-based movement | biological\_process | 0.0 | 1.0 | 1.0 | 0 | cluster\_plot | 97 |
| 1234 | GO:0005956 | protein kinase CK2 complex | cellular\_component | 0.0 | 1.0 | 1.0 | 0 | cluster\_plot | 7 |
| 1235 | GO:0042575 | DNA polymerase complex | cellular\_component | 0.0 | 1.0 | 1.0 | 0 | cluster\_plot | 6 |
| 1236 | GO:0004316 | 3-oxoacyl-[acyl-carrier-protein] reductase (NADPH) activity | molecular\_function | 0.0 | 1.0 | 1.0 | 0 | cluster\_plot | 2 |
| 1237 | GO:0006163 | purine nucleotide metabolic process | biological\_process | 0.0 | 1.0 | 1.0 | 0 | cluster\_plot | 135 |
| 1238 | GO:0004576 | oligosaccharyl transferase activity | molecular\_function | 0.0 | 1.0 | 1.0 | 0 | cluster\_plot | 12 |
| 1239 | GO:0008763 | UDP-N-acetylmuramate-L-alanine ligase activity | molecular\_function | 0.0 | 1.0 | 1.0 | 0 | cluster\_plot | 3 |
| 1240 | GO:0072330 | monocarboxylic acid biosynthetic process | biological\_process | 0.0 | 1.0 | 1.0 | 0 | cluster\_plot | 128 |
| 1241 | GO:0006560 | proline metabolic process | biological\_process | 0.0 | 1.0 | 1.0 | 0 | cluster\_plot | 10 |
| 1242 | GO:0006824 | cobalt ion transport | biological\_process | 0.0 | 1.0 | 1.0 | 0 | cluster\_plot | 2 |
| 1243 | GO:0051187 | cofactor catabolic process | biological\_process | 0.0 | 1.0 | 1.0 | 0 | cluster\_plot | 7 |
| 1244 | GO:0006754 | ATP biosynthetic process | biological\_process | 0.0 | 1.0 | 1.0 | 0 | cluster\_plot | 49 |
| 1245 | GO:0046933 | proton-transporting ATP synthase activity, rotational mechanism | molecular\_function | 0.0 | 1.0 | 1.0 | 0 | cluster\_plot | 24 |
| 1246 | GO:1901137 | carbohydrate derivative biosynthetic process | biological\_process | 0.0 | 1.0 | 1.0 | 0 | cluster\_plot | 130 |
| 1247 | GO:0008444 | CDP-diacylglycerol-glycerol-3-phosphate 3-phosphatidyltransferase activity | molecular\_function | 0.0 | 1.0 | 1.0 | 0 | cluster\_plot | 1 |
| 1248 | GO:0005787 | signal peptidase complex | cellular\_component | 0.0 | 1.0 | 1.0 | 0 | cluster\_plot | 10 |
| 1249 | GO:0016226 | iron-sulfur cluster assembly | biological\_process | 0.0 | 1.0 | 1.0 | 0 | cluster\_plot | 20 |
| 1250 | GO:0030418 | nicotianamine biosynthetic process | biological\_process | 0.0 | 1.0 | 1.0 | 0 | cluster\_plot | 2 |
| 1251 | GO:0005746 | mitochondrial respiratory chain | cellular\_component | 0.0 | 1.0 | 1.0 | 0 | cluster\_plot | 3 |
| 1252 | GO:0043039 | tRNA aminoacylation | biological\_process | 0.0 | 1.0 | 1.0 | 0 | cluster\_plot | 87 |
| 1253 | GO:0004818 | glutamate-tRNA ligase activity | molecular\_function | 0.0 | 1.0 | 1.0 | 0 | cluster\_plot | 3 |
| 1254 | GO:0030041 | actin filament polymerization | biological\_process | 0.0 | 1.0 | 1.0 | 0 | cluster\_plot | 1 |
| 1255 | GO:0009219 | pyrimidine deoxyribonucleotide metabolic process | biological\_process | 0.0 | 1.0 | 1.0 | 0 | cluster\_plot | 4 |
| 1256 | GO:0005801 | cis-Golgi network | cellular\_component | 0.0 | 1.0 | 1.0 | 0 | cluster\_plot | 11 |
| 1257 | GO:0009161 | ribonucleoside monophosphate metabolic process | biological\_process | 0.0 | 1.0 | 1.0 | 0 | cluster\_plot | 84 |
| 1258 | GO:0046834 | lipid phosphorylation | biological\_process | 0.0 | 1.0 | 1.0 | 0 | cluster\_plot | 13 |
| 1259 | GO:0004315 | 3-oxoacyl-[acyl-carrier-protein] synthase activity | molecular\_function | 0.0 | 1.0 | 1.0 | 0 | cluster\_plot | 3 |
| 1260 | GO:0006665 | sphingolipid metabolic process | biological\_process | 0.0 | 1.0 | 1.0 | 0 | cluster\_plot | 8 |
| 1261 | GO:0031327 | negative regulation of cellular biosynthetic process | biological\_process | 0.0 | 1.0 | 1.0 | 0 | cluster\_plot | 20 |
| 1262 | GO:0019139 | cytokinin dehydrogenase activity | molecular\_function | 0.0 | 1.0 | 1.0 | 0 | cluster\_plot | 13 |
| 1263 | GO:0072338 | cellular lactam metabolic process | biological\_process | 0.0 | 1.0 | 1.0 | 0 | cluster\_plot | 1 |
| 1264 | GO:0008308 | voltage-gated anion channel activity | molecular\_function | 0.0 | 1.0 | 1.0 | 0 | cluster\_plot | 13 |
| 1265 | GO:0072594 | establishment of protein localization to organelle | biological\_process | 0.0 | 1.0 | 1.0 | 0 | cluster\_plot | 29 |
| 1266 | GO:1902531 | regulation of intracellular signal transduction | biological\_process | 0.0 | 1.0 | 1.0 | 0 | cluster\_plot | 12 |
| 1267 | GO:0003857 | 3-hydroxyacyl-CoA dehydrogenase activity | molecular\_function | 0.0 | 1.0 | 1.0 | 0 | cluster\_plot | 4 |
| 1268 | GO:0072329 | monocarboxylic acid catabolic process | biological\_process | 0.0 | 1.0 | 1.0 | 0 | cluster\_plot | 9 |
| 1269 | GO:0016837 | carbon-oxygen lyase activity, acting on polysaccharides | molecular\_function | 0.0 | 1.0 | 1.0 | 0 | cluster\_plot | 3 |
| 1270 | GO:0004864 | protein phosphatase inhibitor activity | molecular\_function | 0.0 | 1.0 | 1.0 | 0 | cluster\_plot | 1 |
| 1271 | GO:0009052 | pentose-phosphate shunt, non-oxidative branch | biological\_process | 0.0 | 1.0 | 1.0 | 0 | cluster\_plot | 4 |
| 1272 | GO:0044427 | chromosomal part | cellular\_component | 0.0 | 1.0 | 1.0 | 0 | cluster\_plot | 59 |
| 1273 | GO:0004665 | prephenate dehydrogenase (NADP+) activity | molecular\_function | 0.0 | 1.0 | 1.0 | 0 | cluster\_plot | 4 |
| 1274 | GO:0046912 | transferase activity, transferring acyl groups, acyl groups converted into alkyl on transfer | molecular\_function | 0.0 | 1.0 | 1.0 | 0 | cluster\_plot | 14 |
| 1275 | GO:0004824 | lysine-tRNA ligase activity | molecular\_function | 0.0 | 1.0 | 1.0 | 0 | cluster\_plot | 3 |
| 1276 | GO:0006597 | spermine biosynthetic process | biological\_process | 0.0 | 1.0 | 1.0 | 0 | cluster\_plot | 5 |
| 1277 | GO:0050311 | sulfite reductase (ferredoxin) activity | molecular\_function | 0.0 | 1.0 | 1.0 | 0 | cluster\_plot | 3 |
| 1278 | GO:0009211 | pyrimidine deoxyribonucleoside triphosphate metabolic process | biological\_process | 0.0 | 1.0 | 1.0 | 0 | cluster\_plot | 1 |
| 1279 | GO:0004671 | protein C-terminal S-isoprenylcysteine carboxyl O-methyltransferase activity | molecular\_function | 0.0 | 1.0 | 1.0 | 0 | cluster\_plot | 1 |
| 1280 | GO:0009139 | pyrimidine nucleoside diphosphate biosynthetic process | biological\_process | 0.0 | 1.0 | 1.0 | 0 | cluster\_plot | 2 |
| 1281 | GO:0046128 | purine ribonucleoside metabolic process | biological\_process | 0.0 | 1.0 | 1.0 | 0 | cluster\_plot | 112 |
| 1282 | GO:0046160 | heme a metabolic process | biological\_process | 0.0 | 1.0 | 1.0 | 0 | cluster\_plot | 1 |
| 1283 | GO:0046049 | UMP metabolic process | biological\_process | 0.0 | 1.0 | 1.0 | 0 | cluster\_plot | 5 |
| 1284 | GO:0006471 | protein ADP-ribosylation | biological\_process | 0.0 | 1.0 | 1.0 | 0 | cluster\_plot | 5 |
| 1285 | GO:0070201 | regulation of establishment of protein localization | biological\_process | 0.0 | 1.0 | 1.0 | 0 | cluster\_plot | 2 |
| 1286 | GO:0003855 | 3-dehydroquinate dehydratase activity | molecular\_function | 0.0 | 1.0 | 1.0 | 0 | cluster\_plot | 5 |
| 1287 | GO:0005984 | disaccharide metabolic process | biological\_process | 0.0 | 1.0 | 1.0 | 0 | cluster\_plot | 57 |
| 1288 | GO:0009138 | pyrimidine nucleoside diphosphate metabolic process | biological\_process | 0.0 | 1.0 | 1.0 | 0 | cluster\_plot | 2 |
| 1289 | GO:0033176 | proton-transporting V-type ATPase complex | cellular\_component | 0.0 | 1.0 | 1.0 | 0 | cluster\_plot | 7 |
| 1290 | GO:0004055 | argininosuccinate synthase activity | molecular\_function | 0.0 | 1.0 | 1.0 | 0 | cluster\_plot | 3 |
| 1291 | GO:1901293 | nucleoside phosphate biosynthetic process | biological\_process | 0.0 | 1.0 | 1.0 | 0 | cluster\_plot | 133 |
| 1292 | GO:0006432 | phenylalanyl-tRNA aminoacylation | biological\_process | 0.0 | 1.0 | 1.0 | 0 | cluster\_plot | 7 |
| 1293 | GO:0043062 | extracellular structure organization | biological\_process | 0.0 | 1.0 | 1.0 | 0 | cluster\_plot | 17 |
| 1294 | GO:0008131 | primary amine oxidase activity | molecular\_function | 0.0 | 1.0 | 1.0 | 0 | cluster\_plot | 15 |
| 1295 | GO:0016042 | lipid catabolic process | biological\_process | 0.0 | 1.0 | 1.0 | 0 | cluster\_plot | 13 |
| 1296 | GO:0043632 | modification-dependent macromolecule catabolic process | biological\_process | 0.0 | 1.0 | 1.0 | 0 | cluster\_plot | 157 |
| 1297 | GO:0004573 | mannosyl-oligosaccharide glucosidase activity | molecular\_function | 0.0 | 1.0 | 1.0 | 0 | cluster\_plot | 2 |
| 1298 | GO:0010035 | response to inorganic substance | biological\_process | 0.0 | 1.0 | 1.0 | 0 | cluster\_plot | 13 |
| 1299 | GO:0004160 | dihydroxy-acid dehydratase activity | molecular\_function | 0.0 | 1.0 | 1.0 | 0 | cluster\_plot | 1 |
| 1300 | GO:0030880 | RNA polymerase complex | cellular\_component | 0.0 | 1.0 | 1.0 | 0 | cluster\_plot | 9 |
| 1301 | GO:0042803 | protein homodimerization activity | molecular\_function | 0.0 | 1.0 | 1.0 | 0 | cluster\_plot | 11 |
| 1302 | GO:0010604 | positive regulation of macromolecule metabolic process | biological\_process | 0.0 | 1.0 | 1.0 | 0 | cluster\_plot | 7 |
| 1303 | GO:0009496 | plastoquinol--plastocyanin reductase activity | molecular\_function | 0.0 | 1.0 | 1.0 | 0 | cluster\_plot | 2 |
| 1304 | GO:0003856 | 3-dehydroquinate synthase activity | molecular\_function | 0.0 | 1.0 | 1.0 | 0 | cluster\_plot | 1 |
| 1305 | GO:0010024 | phytochromobilin biosynthetic process | biological\_process | 0.0 | 1.0 | 1.0 | 0 | cluster\_plot | 2 |
| 1306 | GO:0019682 | glyceraldehyde-3-phosphate metabolic process | biological\_process | 0.0 | 1.0 | 1.0 | 0 | cluster\_plot | 2 |
| 1307 | GO:0009251 | glucan catabolic process | biological\_process | 0.0 | 1.0 | 1.0 | 0 | cluster\_plot | 3 |
| 1308 | GO:0042256 | mature ribosome assembly | biological\_process | 0.0 | 1.0 | 1.0 | 0 | cluster\_plot | 2 |
| 1309 | GO:0008883 | glutamyl-tRNA reductase activity | molecular\_function | 0.0 | 1.0 | 1.0 | 0 | cluster\_plot | 5 |
| 1310 | GO:0016882 | cyclo-ligase activity | molecular\_function | 0.0 | 1.0 | 1.0 | 0 | cluster\_plot | 6 |
| 1311 | GO:0042170 | plastid membrane | cellular\_component | 0.0 | 1.0 | 1.0 | 0 | cluster\_plot | 2 |
| 1312 | GO:0005216 | ion channel activity | molecular\_function | 0.0 | 1.0 | 1.0 | 0 | cluster\_plot | 76 |
| 1313 | GO:0009538 | photosystem I reaction center | cellular\_component | 0.0 | 1.0 | 1.0 | 0 | cluster\_plot | 8 |
| 1314 | GO:0007033 | vacuole organization | biological\_process | 0.0 | 1.0 | 1.0 | 0 | cluster\_plot | 1 |
| 1315 | GO:0005057 | receptor signaling protein activity | molecular\_function | 0.0 | 1.0 | 1.0 | 0 | cluster\_plot | 55 |
| 1316 | GO:0008508 | bile acid:sodium symporter activity | molecular\_function | 0.0 | 1.0 | 1.0 | 0 | cluster\_plot | 11 |
| 1317 | GO:0046486 | glycerolipid metabolic process | biological\_process | 0.0 | 1.0 | 1.0 | 0 | cluster\_plot | 66 |
| 1318 | GO:0019843 | rRNA binding | molecular\_function | 0.0 | 1.0 | 1.0 | 0 | cluster\_plot | 21 |
| 1319 | GO:0048878 | chemical homeostasis | biological\_process | 0.0 | 1.0 | 1.0 | 0 | cluster\_plot | 11 |
| 1320 | GO:0019829 | cation-transporting ATPase activity | molecular\_function | 0.0 | 1.0 | 1.0 | 0 | cluster\_plot | 117 |
| 1321 | GO:0051248 | negative regulation of protein metabolic process | biological\_process | 0.0 | 1.0 | 1.0 | 0 | cluster\_plot | 10 |
| 1322 | GO:0046554 | malate dehydrogenase (NADP+) activity | molecular\_function | 0.0 | 1.0 | 1.0 | 0 | cluster\_plot | 3 |
| 1323 | GO:0008204 | ergosterol metabolic process | biological\_process | 0.0 | 1.0 | 1.0 | 0 | cluster\_plot | 3 |
| 1324 | GO:0000049 | tRNA binding | molecular\_function | 0.0 | 1.0 | 1.0 | 0 | cluster\_plot | 13 |
| 1325 | GO:0048193 | Golgi vesicle transport | biological\_process | 0.0 | 1.0 | 1.0 | 0 | cluster\_plot | 52 |
| 1326 | GO:0008612 | peptidyl-lysine modification to peptidyl-hypusine | biological\_process | 0.0 | 1.0 | 1.0 | 0 | cluster\_plot | 5 |
| 1327 | GO:0008531 | riboflavin kinase activity | molecular\_function | 0.0 | 1.0 | 1.0 | 0 | cluster\_plot | 1 |
| 1328 | GO:0015766 | disaccharide transport | biological\_process | 0.0 | 1.0 | 1.0 | 0 | cluster\_plot | 6 |
| 1329 | GO:0006431 | methionyl-tRNA aminoacylation | biological\_process | 0.0 | 1.0 | 1.0 | 0 | cluster\_plot | 3 |
| 1330 | GO:0008037 | cell recognition | biological\_process | 0.0 | 1.0 | 1.0 | 0 | cluster\_plot | 115 |
| 1331 | GO:0005977 | glycogen metabolic process | biological\_process | 0.0 | 1.0 | 1.0 | 0 | cluster\_plot | 3 |
| 1332 | GO:0009156 | ribonucleoside monophosphate biosynthetic process | biological\_process | 0.0 | 1.0 | 1.0 | 0 | cluster\_plot | 76 |
| 1333 | GO:0046658 | anchored component of plasma membrane | cellular\_component | 0.0 | 1.0 | 1.0 | 0 | cluster\_plot | 2 |
| 1334 | GO:0004813 | alanine-tRNA ligase activity | molecular\_function | 0.0 | 1.0 | 1.0 | 0 | cluster\_plot | 5 |
| 1335 | GO:0005346 | purine ribonucleotide transmembrane transporter activity | molecular\_function | 0.0 | 1.0 | 1.0 | 0 | cluster\_plot | 1 |
| 1336 | GO:0003989 | acetyl-CoA carboxylase activity | molecular\_function | 0.0 | 1.0 | 1.0 | 0 | cluster\_plot | 7 |
| 1337 | GO:0006420 | arginyl-tRNA aminoacylation | biological\_process | 0.0 | 1.0 | 1.0 | 0 | cluster\_plot | 1 |
| 1338 | GO:0030245 | cellulose catabolic process | biological\_process | 0.0 | 1.0 | 1.0 | 0 | cluster\_plot | 3 |
| 1339 | GO:0004819 | glutamine-tRNA ligase activity | molecular\_function | 0.0 | 1.0 | 1.0 | 0 | cluster\_plot | 3 |
| 1340 | GO:0006750 | glutathione biosynthetic process | biological\_process | 0.0 | 1.0 | 1.0 | 0 | cluster\_plot | 7 |
| 1341 | GO:0034755 | iron ion transmembrane transport | biological\_process | 0.0 | 1.0 | 1.0 | 0 | cluster\_plot | 1 |
| 1342 | GO:0071804 | cellular potassium ion transport | biological\_process | 0.0 | 1.0 | 1.0 | 0 | cluster\_plot | 23 |
| 1343 | GO:0000996 | core DNA-dependent RNA polymerase binding promoter specificity activity | molecular\_function | 0.0 | 1.0 | 1.0 | 0 | cluster\_plot | 12 |
| 1344 | GO:0035299 | inositol pentakisphosphate 2-kinase activity | molecular\_function | 0.0 | 1.0 | 1.0 | 0 | cluster\_plot | 2 |
| 1345 | GO:0016756 | glutathione gamma-glutamylcysteinyltransferase activity | molecular\_function | 0.0 | 1.0 | 1.0 | 0 | cluster\_plot | 3 |
| 1346 | GO:0032507 | maintenance of protein location in cell | biological\_process | 0.0 | 1.0 | 1.0 | 0 | cluster\_plot | 13 |
| 1347 | GO:0006081 | cellular aldehyde metabolic process | biological\_process | 0.0 | 1.0 | 1.0 | 0 | cluster\_plot | 14 |
| 1348 | GO:0046493 | lipid A metabolic process | biological\_process | 0.0 | 1.0 | 1.0 | 0 | cluster\_plot | 3 |
| 1349 | GO:0004852 | uroporphyrinogen-III synthase activity | molecular\_function | 0.0 | 1.0 | 1.0 | 0 | cluster\_plot | 3 |
| 1350 | GO:0071900 | regulation of protein serine/threonine kinase activity | biological\_process | 0.0 | 1.0 | 1.0 | 0 | cluster\_plot | 11 |
| 1351 | GO:0003997 | acyl-CoA oxidase activity | molecular\_function | 0.0 | 1.0 | 1.0 | 0 | cluster\_plot | 7 |
| 1352 | GO:0018580 | nitronate monooxygenase activity | molecular\_function | 0.0 | 1.0 | 1.0 | 0 | cluster\_plot | 2 |
| 1353 | GO:0051087 | chaperone binding | molecular\_function | 0.0 | 1.0 | 1.0 | 0 | cluster\_plot | 24 |
| 1354 | GO:0009199 | ribonucleoside triphosphate metabolic process | biological\_process | 0.0 | 1.0 | 1.0 | 0 | cluster\_plot | 86 |
| 1355 | GO:0006487 | protein N-linked glycosylation | biological\_process | 0.0 | 1.0 | 1.0 | 0 | cluster\_plot | 9 |
| 1356 | GO:0006974 | cellular response to DNA damage stimulus | biological\_process | 0.0 | 1.0 | 1.0 | 0 | cluster\_plot | 131 |
| 1357 | GO:0009132 | nucleoside diphosphate metabolic process | biological\_process | 0.0 | 1.0 | 1.0 | 0 | cluster\_plot | 15 |
| 1358 | GO:0006813 | potassium ion transport | biological\_process | 0.0 | 1.0 | 1.0 | 0 | cluster\_plot | 26 |
| 1359 | GO:0046131 | pyrimidine ribonucleoside metabolic process | biological\_process | 0.0 | 1.0 | 1.0 | 0 | cluster\_plot | 12 |
| 1360 | GO:0006571 | tyrosine biosynthetic process | biological\_process | 0.0 | 1.0 | 1.0 | 0 | cluster\_plot | 4 |
| 1361 | GO:0006188 | IMP biosynthetic process | biological\_process | 0.0 | 1.0 | 1.0 | 0 | cluster\_plot | 15 |
| 1362 | GO:0006452 | translational frameshifting | biological\_process | 0.0 | 1.0 | 1.0 | 0 | cluster\_plot | 3 |
| 1363 | GO:0071822 | protein complex subunit organization | biological\_process | 0.0 | 1.0 | 1.0 | 0 | cluster\_plot | 93 |
| 1364 | GO:0004601 | peroxidase activity | molecular\_function | 0.0 | 1.0 | 1.0 | 0 | cluster\_plot | 142 |
| 1365 | GO:0032502 | developmental process | biological\_process | 0.0 | 1.0 | 1.0 | 0 | cluster\_plot | 21 |
| 1366 | GO:0070838 | divalent metal ion transport | biological\_process | 0.0 | 1.0 | 1.0 | 0 | cluster\_plot | 57 |
| 1367 | GO:0004636 | phosphoribosyl-ATP diphosphatase activity | molecular\_function | 0.0 | 1.0 | 1.0 | 0 | cluster\_plot | 3 |
| 1368 | GO:0005779 | integral component of peroxisomal membrane | cellular\_component | 0.0 | 1.0 | 1.0 | 0 | cluster\_plot | 11 |
| 1369 | GO:0015693 | magnesium ion transport | biological\_process | 0.0 | 1.0 | 1.0 | 0 | cluster\_plot | 16 |
| 1370 | GO:0072600 | establishment of protein localization to Golgi | biological\_process | 0.0 | 1.0 | 1.0 | 0 | cluster\_plot | 3 |
| 1371 | GO:0015985 | energy coupled proton transport, down electrochemical gradient | biological\_process | 0.0 | 1.0 | 1.0 | 0 | cluster\_plot | 35 |
| 1372 | GO:0004425 | indole-3-glycerol-phosphate synthase activity | molecular\_function | 0.0 | 1.0 | 1.0 | 0 | cluster\_plot | 1 |
| 1373 | GO:0004556 | alpha-amylase activity | molecular\_function | 0.0 | 1.0 | 1.0 | 0 | cluster\_plot | 10 |
| 1374 | GO:0030301 | cholesterol transport | biological\_process | 0.0 | 1.0 | 1.0 | 0 | cluster\_plot | 1 |
| 1375 | GO:0032259 | methylation | biological\_process | 0.0 | 1.0 | 1.0 | 0 | cluster\_plot | 65 |
| 1376 | GO:0032012 | regulation of ARF protein signal transduction | biological\_process | 0.0 | 1.0 | 1.0 | 0 | cluster\_plot | 11 |
| 1377 | GO:0016831 | carboxy-lyase activity | molecular\_function | 0.0 | 1.0 | 1.0 | 0 | cluster\_plot | 50 |
| 1378 | GO:0016872 | intramolecular lyase activity | molecular\_function | 0.0 | 1.0 | 1.0 | 0 | cluster\_plot | 19 |
| 1379 | GO:0006144 | purine nucleobase metabolic process | biological\_process | 0.0 | 1.0 | 1.0 | 0 | cluster\_plot | 15 |
| 1380 | GO:0051174 | regulation of phosphorus metabolic process | biological\_process | 0.0 | 1.0 | 1.0 | 0 | cluster\_plot | 66 |
| 1381 | GO:0016655 | oxidoreductase activity, acting on NAD(P)H, quinone or similar compound as acceptor | molecular\_function | 0.0 | 1.0 | 1.0 | 0 | cluster\_plot | 13 |
| 1382 | GO:0044767 | single-organism developmental process | biological\_process | 0.0 | 1.0 | 1.0 | 0 | cluster\_plot | 21 |
| 1383 | GO:0016876 | ligase activity, forming aminoacyl-tRNA and related compounds | molecular\_function | 0.0 | 1.0 | 1.0 | 0 | cluster\_plot | 87 |
| 1384 | GO:0042455 | ribonucleoside biosynthetic process | biological\_process | 0.0 | 1.0 | 1.0 | 0 | cluster\_plot | 80 |
| 1385 | GO:0006606 | protein import into nucleus | biological\_process | 0.0 | 1.0 | 1.0 | 0 | cluster\_plot | 9 |
| 1386 | GO:0005102 | receptor binding | molecular\_function | 0.0 | 1.0 | 1.0 | 0 | cluster\_plot | 11 |
| 1387 | GO:0045184 | establishment of protein localization | biological\_process | 0.0 | 1.0 | 1.0 | 0 | cluster\_plot | 247 |
| 1388 | GO:0009229 | thiamine diphosphate biosynthetic process | biological\_process | 0.0 | 1.0 | 1.0 | 0 | cluster\_plot | 2 |
| 1389 | GO:0004170 | dUTP diphosphatase activity | molecular\_function | 0.0 | 1.0 | 1.0 | 0 | cluster\_plot | 1 |
| 1390 | GO:0019288 | isopentenyl diphosphate biosynthetic process, methylerythritol 4-phosphate pathway | biological\_process | 0.0 | 1.0 | 1.0 | 0 | cluster\_plot | 2 |
| 1391 | GO:0031461 | cullin-RING ubiquitin ligase complex | cellular\_component | 0.0 | 1.0 | 1.0 | 0 | cluster\_plot | 26 |
| 1392 | GO:0009174 | pyrimidine ribonucleoside monophosphate biosynthetic process | biological\_process | 0.0 | 1.0 | 1.0 | 0 | cluster\_plot | 5 |
| 1393 | GO:0046488 | phosphatidylinositol metabolic process | biological\_process | 0.0 | 1.0 | 1.0 | 0 | cluster\_plot | 55 |
| 1394 | GO:0015085 | calcium ion transmembrane transporter activity | molecular\_function | 0.0 | 1.0 | 1.0 | 0 | cluster\_plot | 38 |
| 1395 | GO:0008108 | UDP-glucose:hexose-1-phosphate uridylyltransferase activity | molecular\_function | 0.0 | 1.0 | 1.0 | 0 | cluster\_plot | 1 |
| 1396 | GO:0017056 | structural constituent of nuclear pore | molecular\_function | 0.0 | 1.0 | 1.0 | 0 | cluster\_plot | 2 |
| 1397 | GO:0051338 | regulation of transferase activity | biological\_process | 0.0 | 1.0 | 1.0 | 0 | cluster\_plot | 11 |
| 1398 | GO:0043173 | nucleotide salvage | biological\_process | 0.0 | 1.0 | 1.0 | 0 | cluster\_plot | 2 |
| 1399 | GO:0032318 | regulation of Ras GTPase activity | biological\_process | 0.0 | 1.0 | 1.0 | 0 | cluster\_plot | 51 |
| 1400 | GO:0030291 | protein serine/threonine kinase inhibitor activity | molecular\_function | 0.0 | 1.0 | 1.0 | 0 | cluster\_plot | 8 |
| 1401 | GO:0004834 | tryptophan synthase activity | molecular\_function | 0.0 | 1.0 | 1.0 | 0 | cluster\_plot | 9 |
| 1402 | GO:0015672 | monovalent inorganic cation transport | biological\_process | 0.0 | 1.0 | 1.0 | 0 | cluster\_plot | 157 |
| 1403 | GO:0045934 | negative regulation of nucleobase-containing compound metabolic process | biological\_process | 0.0 | 1.0 | 1.0 | 0 | cluster\_plot | 13 |
| 1404 | GO:0016630 | protochlorophyllide reductase activity | molecular\_function | 0.0 | 1.0 | 1.0 | 0 | cluster\_plot | 2 |
| 1405 | GO:0009117 | nucleotide metabolic process | biological\_process | 0.0 | 1.0 | 1.0 | 0 | cluster\_plot | 211 |
| 1406 | GO:0004488 | methylenetetrahydrofolate dehydrogenase (NADP+) activity | molecular\_function | 0.0 | 1.0 | 1.0 | 0 | cluster\_plot | 5 |
| 1407 | GO:0000042 | protein targeting to Golgi | biological\_process | 0.0 | 1.0 | 1.0 | 0 | cluster\_plot | 3 |
| 1408 | GO:0015144 | carbohydrate transmembrane transporter activity | molecular\_function | 0.0 | 1.0 | 1.0 | 0 | cluster\_plot | 18 |
| 1409 | GO:0005991 | trehalose metabolic process | biological\_process | 0.0 | 1.0 | 1.0 | 0 | cluster\_plot | 22 |
| 1410 | GO:0019395 | fatty acid oxidation | biological\_process | 0.0 | 1.0 | 1.0 | 0 | cluster\_plot | 7 |
| 1411 | GO:1901990 | regulation of mitotic cell cycle phase transition | biological\_process | 0.0 | 1.0 | 1.0 | 0 | cluster\_plot | 5 |
| 1412 | GO:0046451 | diaminopimelate metabolic process | biological\_process | 0.0 | 1.0 | 1.0 | 0 | cluster\_plot | 13 |
| 1413 | GO:0071669 | plant-type cell wall organization or biogenesis | biological\_process | 0.0 | 1.0 | 1.0 | 0 | cluster\_plot | 1 |
| 1414 | GO:0035091 | phosphatidylinositol binding | molecular\_function | 0.0 | 1.0 | 1.0 | 0 | cluster\_plot | 14 |
| 1415 | GO:0009039 | urease activity | molecular\_function | 0.0 | 1.0 | 1.0 | 0 | cluster\_plot | 1 |
| 1416 | GO:0051235 | maintenance of location | biological\_process | 0.0 | 1.0 | 1.0 | 0 | cluster\_plot | 13 |
| 1417 | GO:0031328 | positive regulation of cellular biosynthetic process | biological\_process | 0.0 | 1.0 | 1.0 | 0 | cluster\_plot | 7 |
| 1418 | GO:0009064 | glutamine family amino acid metabolic process | biological\_process | 0.0 | 1.0 | 1.0 | 0 | cluster\_plot | 54 |
| 1419 | GO:0051726 | regulation of cell cycle | biological\_process | 0.0 | 1.0 | 1.0 | 0 | cluster\_plot | 27 |
| 1420 | GO:0005874 | microtubule | cellular\_component | 0.0 | 1.0 | 1.0 | 0 | cluster\_plot | 3 |
| 1421 | GO:0006241 | CTP biosynthetic process | biological\_process | 0.0 | 1.0 | 1.0 | 0 | cluster\_plot | 7 |
| 1422 | GO:0008614 | pyridoxine metabolic process | biological\_process | 0.0 | 1.0 | 1.0 | 0 | cluster\_plot | 1 |
| 1423 | GO:0019509 | L-methionine salvage from methylthioadenosine | biological\_process | 0.0 | 1.0 | 1.0 | 0 | cluster\_plot | 2 |
| 1424 | GO:0030036 | actin cytoskeleton organization | biological\_process | 0.0 | 1.0 | 1.0 | 0 | cluster\_plot | 14 |
| 1425 | GO:0048034 | heme O biosynthetic process | biological\_process | 0.0 | 1.0 | 1.0 | 0 | cluster\_plot | 2 |
| 1426 | GO:0016117 | carotenoid biosynthetic process | biological\_process | 0.0 | 1.0 | 1.0 | 0 | cluster\_plot | 9 |
| 1427 | GO:0006072 | glycerol-3-phosphate metabolic process | biological\_process | 0.0 | 1.0 | 1.0 | 0 | cluster\_plot | 12 |
| 1428 | GO:0000162 | tryptophan biosynthetic process | biological\_process | 0.0 | 1.0 | 1.0 | 0 | cluster\_plot | 8 |
| 1429 | GO:0009113 | purine nucleobase biosynthetic process | biological\_process | 0.0 | 1.0 | 1.0 | 0 | cluster\_plot | 15 |
| 1430 | GO:0045047 | protein targeting to ER | biological\_process | 0.0 | 1.0 | 1.0 | 0 | cluster\_plot | 17 |
| 1431 | GO:0004417 | hydroxyethylthiazole kinase activity | molecular\_function | 0.0 | 1.0 | 1.0 | 0 | cluster\_plot | 2 |
| 1432 | GO:0007155 | cell adhesion | biological\_process | 0.0 | 1.0 | 1.0 | 0 | cluster\_plot | 4 |
| 1433 | GO:1903046 | meiotic cell cycle process | biological\_process | 0.0 | 1.0 | 1.0 | 0 | cluster\_plot | 6 |
| 1434 | GO:0071174 | mitotic spindle checkpoint | biological\_process | 0.0 | 1.0 | 1.0 | 0 | cluster\_plot | 2 |
| 1435 | GO:0010038 | response to metal ion | biological\_process | 0.0 | 1.0 | 1.0 | 0 | cluster\_plot | 5 |
| 1436 | GO:0018298 | protein-chromophore linkage | biological\_process | 0.0 | 1.0 | 1.0 | 0 | cluster\_plot | 8 |
| 1437 | GO:0009452 | 7-methylguanosine RNA capping | biological\_process | 0.0 | 1.0 | 1.0 | 0 | cluster\_plot | 7 |
| 1438 | GO:0031145 | anaphase-promoting complex-dependent proteasomal ubiquitin-dependent protein catabolic process | biological\_process | 0.0 | 1.0 | 1.0 | 0 | cluster\_plot | 2 |
| 1439 | GO:0004358 | glutamate N-acetyltransferase activity | molecular\_function | 0.0 | 1.0 | 1.0 | 0 | cluster\_plot | 3 |
| 1440 | GO:0046434 | organophosphate catabolic process | biological\_process | 0.0 | 1.0 | 1.0 | 0 | cluster\_plot | 51 |
| 1441 | GO:0000445 | THO complex part of transcription export complex | cellular\_component | 0.0 | 1.0 | 1.0 | 0 | cluster\_plot | 4 |
| 1442 | GO:0043085 | positive regulation of catalytic activity | biological\_process | 0.0 | 1.0 | 1.0 | 0 | cluster\_plot | 2 |
| 1443 | GO:0019276 | UDP-N-acetylgalactosamine metabolic process | biological\_process | 0.0 | 1.0 | 1.0 | 0 | cluster\_plot | 2 |
| 1444 | GO:0051302 | regulation of cell division | biological\_process | 0.0 | 1.0 | 1.0 | 0 | cluster\_plot | 6 |
| 1445 | GO:0031667 | response to nutrient levels | biological\_process | 0.0 | 1.0 | 1.0 | 0 | cluster\_plot | 2 |
| 1446 | GO:0008725 | DNA-3-methyladenine glycosylase activity | molecular\_function | 0.0 | 1.0 | 1.0 | 0 | cluster\_plot | 10 |
| 1447 | GO:0043189 | H4/H2A histone acetyltransferase complex | cellular\_component | 0.0 | 1.0 | 1.0 | 0 | cluster\_plot | 2 |
| 1448 | GO:0070204 | 2-succinyl-5-enolpyruvyl-6-hydroxy-3-cyclohexene-1-carboxylic-acid synthase activity | molecular\_function | 0.0 | 1.0 | 1.0 | 0 | cluster\_plot | 1 |
| 1449 | GO:0065008 | regulation of biological quality | biological\_process | 0.0 | 1.0 | 1.0 | 0 | cluster\_plot | 212 |
| 1450 | GO:0051082 | unfolded protein binding | molecular\_function | 0.0 | 1.0 | 1.0 | 0 | cluster\_plot | 71 |
| 1451 | GO:0043414 | macromolecule methylation | biological\_process | 0.0 | 1.0 | 1.0 | 0 | cluster\_plot | 49 |
| 1452 | GO:0015995 | chlorophyll biosynthetic process | biological\_process | 0.0 | 1.0 | 1.0 | 0 | cluster\_plot | 15 |
| 1453 | GO:0000702 | oxidized base lesion DNA N-glycosylase activity | molecular\_function | 0.0 | 1.0 | 1.0 | 0 | cluster\_plot | 3 |
| 1454 | GO:0004827 | proline-tRNA ligase activity | molecular\_function | 0.0 | 1.0 | 1.0 | 0 | cluster\_plot | 4 |
| 1455 | GO:0010628 | positive regulation of gene expression | biological\_process | 0.0 | 1.0 | 1.0 | 0 | cluster\_plot | 4 |
| 1456 | GO:0032324 | molybdopterin cofactor biosynthetic process | biological\_process | 0.0 | 1.0 | 1.0 | 0 | cluster\_plot | 10 |
| 1457 | GO:0043933 | macromolecular complex subunit organization | biological\_process | 0.0 | 1.0 | 1.0 | 0 | cluster\_plot | 98 |
| 1458 | GO:0030915 | Smc5-Smc6 complex | cellular\_component | 0.0 | 1.0 | 1.0 | 0 | cluster\_plot | 1 |
| 1459 | GO:0000160 | phosphorelay signal transduction system | biological\_process | 0.0 | 1.0 | 1.0 | 0 | cluster\_plot | 64 |
| 1460 | GO:0016279 | protein-lysine N-methyltransferase activity | molecular\_function | 0.0 | 1.0 | 1.0 | 0 | cluster\_plot | 18 |
| 1461 | GO:0009312 | oligosaccharide biosynthetic process | biological\_process | 0.0 | 1.0 | 1.0 | 0 | cluster\_plot | 29 |
| 1462 | GO:0019365 | pyridine nucleotide salvage | biological\_process | 0.0 | 1.0 | 1.0 | 0 | cluster\_plot | 2 |
| 1463 | GO:0003876 | AMP deaminase activity | molecular\_function | 0.0 | 1.0 | 1.0 | 0 | cluster\_plot | 3 |
| 1464 | GO:0016901 | oxidoreductase activity, acting on the CH-OH group of donors, quinone or similar compound as acceptor | molecular\_function | 0.0 | 1.0 | 1.0 | 0 | cluster\_plot | 5 |
| 1465 | GO:0004647 | phosphoserine phosphatase activity | molecular\_function | 0.0 | 1.0 | 1.0 | 0 | cluster\_plot | 1 |
| 1466 | GO:0032182 | small conjugating protein binding | molecular\_function | 0.0 | 1.0 | 1.0 | 0 | cluster\_plot | 1 |
| 1467 | GO:0042625 | ATPase activity, coupled to transmembrane movement of ions | molecular\_function | 0.0 | 1.0 | 1.0 | 0 | cluster\_plot | 117 |
| 1468 | GO:0033043 | regulation of organelle organization | biological\_process | 0.0 | 1.0 | 1.0 | 0 | cluster\_plot | 15 |
| 1469 | GO:0050664 | oxidoreductase activity, acting on NAD(P)H, oxygen as acceptor | molecular\_function | 0.0 | 1.0 | 1.0 | 0 | cluster\_plot | 11 |
| 1470 | GO:0035383 | thioester metabolic process | biological\_process | 0.0 | 1.0 | 1.0 | 0 | cluster\_plot | 2 |
| 1471 | GO:0004652 | polynucleotide adenylyltransferase activity | molecular\_function | 0.0 | 1.0 | 1.0 | 0 | cluster\_plot | 9 |
| 1472 | GO:0042168 | heme metabolic process | biological\_process | 0.0 | 1.0 | 1.0 | 0 | cluster\_plot | 11 |
| 1473 | GO:0008422 | beta-glucosidase activity | molecular\_function | 0.0 | 1.0 | 1.0 | 0 | cluster\_plot | 3 |
| 1474 | GO:0043419 | urea catabolic process | biological\_process | 0.0 | 1.0 | 1.0 | 0 | cluster\_plot | 1 |
| 1475 | GO:0051537 | 2 iron, 2 sulfur cluster binding | molecular\_function | 0.0 | 1.0 | 1.0 | 0 | cluster\_plot | 22 |
| 1476 | GO:0072522 | purine-containing compound biosynthetic process | biological\_process | 0.0 | 1.0 | 1.0 | 0 | cluster\_plot | 114 |
| 1477 | GO:0030170 | pyridoxal phosphate binding | molecular\_function | 0.0 | 1.0 | 1.0 | 0 | cluster\_plot | 89 |
| 1478 | GO:0005740 | mitochondrial envelope | cellular\_component | 0.0 | 1.0 | 1.0 | 0 | cluster\_plot | 7 |
| 1479 | GO:0005516 | calmodulin binding | molecular\_function | 0.0 | 1.0 | 1.0 | 0 | cluster\_plot | 28 |
| 1480 | GO:0009408 | response to heat | biological\_process | 0.0 | 1.0 | 1.0 | 0 | cluster\_plot | 5 |
| 1481 | GO:0009620 | response to fungus | biological\_process | 0.0 | 1.0 | 1.0 | 0 | cluster\_plot | 1 |
| 1482 | GO:0009142 | nucleoside triphosphate biosynthetic process | biological\_process | 0.0 | 1.0 | 1.0 | 0 | cluster\_plot | 56 |
| 1483 | GO:0008154 | actin polymerization or depolymerization | biological\_process | 0.0 | 1.0 | 1.0 | 0 | cluster\_plot | 1 |
| 1484 | GO:0009583 | detection of light stimulus | biological\_process | 0.0 | 1.0 | 1.0 | 0 | cluster\_plot | 8 |
| 1485 | GO:0006850 | mitochondrial pyruvate transport | biological\_process | 0.0 | 1.0 | 1.0 | 0 | cluster\_plot | 6 |
| 1486 | GO:0006885 | regulation of pH | biological\_process | 0.0 | 1.0 | 1.0 | 0 | cluster\_plot | 5 |
| 1487 | GO:0009097 | isoleucine biosynthetic process | biological\_process | 0.0 | 1.0 | 1.0 | 0 | cluster\_plot | 3 |
| 1488 | GO:0051499 | D-aminoacyl-tRNA deacylase activity | molecular\_function | 0.0 | 1.0 | 1.0 | 0 | cluster\_plot | 1 |
| 1489 | GO:0042450 | arginine biosynthetic process via ornithine | biological\_process | 0.0 | 1.0 | 1.0 | 0 | cluster\_plot | 3 |
| 1490 | GO:0000107 | imidazoleglycerol-phosphate synthase activity | molecular\_function | 0.0 | 1.0 | 1.0 | 0 | cluster\_plot | 1 |
| 1491 | GO:0009581 | detection of external stimulus | biological\_process | 0.0 | 1.0 | 1.0 | 0 | cluster\_plot | 8 |
| 1492 | GO:0019860 | uracil metabolic process | biological\_process | 0.0 | 1.0 | 1.0 | 0 | cluster\_plot | 1 |
| 1493 | GO:0070003 | threonine-type peptidase activity | molecular\_function | 0.0 | 1.0 | 1.0 | 0 | cluster\_plot | 42 |
| 1494 | GO:0006637 | acyl-CoA metabolic process | biological\_process | 0.0 | 1.0 | 1.0 | 0 | cluster\_plot | 2 |
| 1495 | GO:0003968 | RNA-directed RNA polymerase activity | molecular\_function | 0.0 | 1.0 | 1.0 | 0 | cluster\_plot | 9 |
| 1496 | GO:0015293 | symporter activity | molecular\_function | 0.0 | 1.0 | 1.0 | 0 | cluster\_plot | 25 |
| 1497 | GO:0043492 | ATPase activity, coupled to movement of substances | molecular\_function | 0.0 | 1.0 | 1.0 | 0 | cluster\_plot | 178 |
| 1498 | GO:0019107 | myristoyltransferase activity | molecular\_function | 0.0 | 1.0 | 1.0 | 0 | cluster\_plot | 2 |
| 1499 | GO:0003916 | DNA topoisomerase activity | molecular\_function | 0.0 | 1.0 | 1.0 | 0 | cluster\_plot | 17 |
| 1500 | GO:0008060 | ARF GTPase activator activity | molecular\_function | 0.0 | 1.0 | 1.0 | 0 | cluster\_plot | 28 |
| 1501 | GO:0015031 | protein transport | biological\_process | 0.0 | 1.0 | 1.0 | 0 | cluster\_plot | 240 |
| 1502 | GO:0004148 | dihydrolipoyl dehydrogenase activity | molecular\_function | 0.0 | 1.0 | 1.0 | 0 | cluster\_plot | 1 |
| 1503 | GO:0009088 | threonine biosynthetic process | biological\_process | 0.0 | 1.0 | 1.0 | 0 | cluster\_plot | 1 |
| 1504 | GO:0042054 | histone methyltransferase activity | molecular\_function | 0.0 | 1.0 | 1.0 | 0 | cluster\_plot | 18 |
| 1505 | GO:1902493 | acetyltransferase complex | cellular\_component | 0.0 | 1.0 | 1.0 | 0 | cluster\_plot | 14 |
| 1506 | GO:0046132 | pyrimidine ribonucleoside biosynthetic process | biological\_process | 0.0 | 1.0 | 1.0 | 0 | cluster\_plot | 12 |
| 1507 | GO:0004820 | glycine-tRNA ligase activity | molecular\_function | 0.0 | 1.0 | 1.0 | 0 | cluster\_plot | 3 |
| 1508 | GO:0006278 | RNA-dependent DNA replication | biological\_process | 0.0 | 1.0 | 1.0 | 0 | cluster\_plot | 10 |
| 1509 | GO:0070402 | NADPH binding | molecular\_function | 0.0 | 1.0 | 1.0 | 0 | cluster\_plot | 5 |
| 1510 | GO:0016634 | oxidoreductase activity, acting on the CH-CH group of donors, oxygen as acceptor | molecular\_function | 0.0 | 1.0 | 1.0 | 0 | cluster\_plot | 15 |
| 1511 | GO:0051020 | GTPase binding | molecular\_function | 0.0 | 1.0 | 1.0 | 0 | cluster\_plot | 19 |
| 1512 | GO:0009196 | pyrimidine deoxyribonucleoside diphosphate metabolic process | biological\_process | 0.0 | 1.0 | 1.0 | 0 | cluster\_plot | 2 |
| 1513 | GO:0006424 | glutamyl-tRNA aminoacylation | biological\_process | 0.0 | 1.0 | 1.0 | 0 | cluster\_plot | 3 |
| 1514 | GO:0006304 | DNA modification | biological\_process | 0.0 | 1.0 | 1.0 | 0 | cluster\_plot | 15 |
| 1515 | GO:0030580 | quinone cofactor methyltransferase activity | molecular\_function | 0.0 | 1.0 | 1.0 | 0 | cluster\_plot | 2 |
| 1516 | GO:0015368 | calcium:cation antiporter activity | molecular\_function | 0.0 | 1.0 | 1.0 | 0 | cluster\_plot | 12 |
| 1517 | GO:0006388 | tRNA splicing, via endonucleolytic cleavage and ligation | biological\_process | 0.0 | 1.0 | 1.0 | 0 | cluster\_plot | 2 |
| 1518 | GO:0008107 | galactoside 2-alpha-L-fucosyltransferase activity | molecular\_function | 0.0 | 1.0 | 1.0 | 0 | cluster\_plot | 2 |
| 1519 | GO:0043086 | negative regulation of catalytic activity | biological\_process | 0.0 | 1.0 | 1.0 | 0 | cluster\_plot | 71 |
| 1520 | GO:1901991 | negative regulation of mitotic cell cycle phase transition | biological\_process | 0.0 | 1.0 | 1.0 | 0 | cluster\_plot | 2 |
| 1521 | GO:0008915 | lipid-A-disaccharide synthase activity | molecular\_function | 0.0 | 1.0 | 1.0 | 0 | cluster\_plot | 1 |
| 1522 | GO:0000276 | mitochondrial proton-transporting ATP synthase complex, coupling factor F(o) | cellular\_component | 0.0 | 1.0 | 1.0 | 0 | cluster\_plot | 9 |
| 1523 | GO:0047134 | protein-disulfide reductase activity | molecular\_function | 0.0 | 1.0 | 1.0 | 0 | cluster\_plot | 8 |
| 1524 | GO:1901657 | glycosyl compound metabolic process | biological\_process | 0.0 | 1.0 | 1.0 | 0 | cluster\_plot | 145 |
| 1525 | GO:0000428 | DNA-directed RNA polymerase complex | cellular\_component | 0.0 | 1.0 | 1.0 | 0 | cluster\_plot | 9 |
| 1526 | GO:0030523 | dihydrolipoamide S-acyltransferase activity | molecular\_function | 0.0 | 1.0 | 1.0 | 0 | cluster\_plot | 1 |
| 1527 | GO:0030118 | clathrin coat | cellular\_component | 0.0 | 1.0 | 1.0 | 0 | cluster\_plot | 10 |
| 1528 | GO:0004644 | phosphoribosylglycinamide formyltransferase activity | molecular\_function | 0.0 | 1.0 | 1.0 | 0 | cluster\_plot | 2 |
| 1529 | GO:0015922 | aspartate oxidase activity | molecular\_function | 0.0 | 1.0 | 1.0 | 0 | cluster\_plot | 2 |
| 1530 | GO:0008295 | spermidine biosynthetic process | biological\_process | 0.0 | 1.0 | 1.0 | 0 | cluster\_plot | 6 |
| 1531 | GO:0031969 | chloroplast membrane | cellular\_component | 0.0 | 1.0 | 1.0 | 0 | cluster\_plot | 2 |
| 1532 | GO:0004516 | nicotinate phosphoribosyltransferase activity | molecular\_function | 0.0 | 1.0 | 1.0 | 0 | cluster\_plot | 2 |
| 1533 | GO:0046700 | heterocycle catabolic process | biological\_process | 0.0 | 1.0 | 1.0 | 0 | cluster\_plot | 64 |
| 1534 | GO:0000394 | RNA splicing, via endonucleolytic cleavage and ligation | biological\_process | 0.0 | 1.0 | 1.0 | 0 | cluster\_plot | 2 |
| 1535 | GO:0016208 | AMP binding | molecular\_function | 0.0 | 1.0 | 1.0 | 0 | cluster\_plot | 2 |
| 1536 | GO:0016709 | oxidoreductase activity, acting on paired donors, with incorporation or reduction of molecular oxygen, NAD(P)H as one donor, and incorporation of one atom of oxygen | molecular\_function | 0.0 | 1.0 | 1.0 | 0 | cluster\_plot | 54 |
| 1537 | GO:0043140 | ATP-dependent 3'-5' DNA helicase activity | molecular\_function | 0.0 | 1.0 | 1.0 | 0 | cluster\_plot | 5 |
| 1538 | GO:0044430 | cytoskeletal part | cellular\_component | 0.0 | 1.0 | 1.0 | 0 | cluster\_plot | 58 |
| 1539 | GO:1901987 | regulation of cell cycle phase transition | biological\_process | 0.0 | 1.0 | 1.0 | 0 | cluster\_plot | 5 |
| 1540 | GO:0072595 | maintenance of protein localization in organelle | biological\_process | 0.0 | 1.0 | 1.0 | 0 | cluster\_plot | 11 |
| 1541 | GO:0072339 | cellular lactam biosynthetic process | biological\_process | 0.0 | 1.0 | 1.0 | 0 | cluster\_plot | 1 |
| 1542 | GO:0000988 | protein binding transcription factor activity | molecular\_function | 0.0 | 1.0 | 1.0 | 0 | cluster\_plot | 49 |
| 1543 | GO:0005839 | proteasome core complex | cellular\_component | 0.0 | 1.0 | 1.0 | 0 | cluster\_plot | 42 |
| 1544 | GO:0006461 | protein complex assembly | biological\_process | 0.0 | 1.0 | 1.0 | 0 | cluster\_plot | 77 |
| 1545 | GO:0015780 | nucleotide-sugar transport | biological\_process | 0.0 | 1.0 | 1.0 | 0 | cluster\_plot | 9 |
| 1546 | GO:0070283 | radical SAM enzyme activity | molecular\_function | 0.0 | 1.0 | 1.0 | 0 | cluster\_plot | 6 |
| 1547 | GO:0048544 | recognition of pollen | biological\_process | 0.0 | 1.0 | 1.0 | 0 | cluster\_plot | 115 |
| 1548 | GO:0044428 | nuclear part | cellular\_component | 0.0 | 1.0 | 1.0 | 0 | cluster\_plot | 135 |
| 1549 | GO:0005198 | structural molecule activity | molecular\_function | 0.0 | 1.0 | 1.0 | 0 | cluster\_plot | 517 |
| 1550 | GO:0031127 | alpha-(1,2)-fucosyltransferase activity | molecular\_function | 0.0 | 1.0 | 1.0 | 0 | cluster\_plot | 2 |
| 1551 | GO:0008124 | 4-alpha-hydroxytetrahydrobiopterin dehydratase activity | molecular\_function | 0.0 | 1.0 | 1.0 | 0 | cluster\_plot | 3 |
| 1552 | GO:0033743 | peptide-methionine (R)-S-oxide reductase activity | molecular\_function | 0.0 | 1.0 | 1.0 | 0 | cluster\_plot | 6 |
| 1553 | GO:0022832 | voltage-gated channel activity | molecular\_function | 0.0 | 1.0 | 1.0 | 0 | cluster\_plot | 13 |
| 1554 | GO:0030942 | endoplasmic reticulum signal peptide binding | molecular\_function | 0.0 | 1.0 | 1.0 | 0 | cluster\_plot | 1 |
| 1555 | GO:0009094 | L-phenylalanine biosynthetic process | biological\_process | 0.0 | 1.0 | 1.0 | 0 | cluster\_plot | 8 |
| 1556 | GO:0047750 | cholestenol delta-isomerase activity | molecular\_function | 0.0 | 1.0 | 1.0 | 0 | cluster\_plot | 2 |
| 1557 | GO:0005094 | Rho GDP-dissociation inhibitor activity | molecular\_function | 0.0 | 1.0 | 1.0 | 0 | cluster\_plot | 6 |
| 1558 | GO:0005680 | anaphase-promoting complex | cellular\_component | 0.0 | 1.0 | 1.0 | 0 | cluster\_plot | 7 |
| 1559 | GO:0008144 | drug binding | molecular\_function | 0.0 | 1.0 | 1.0 | 0 | cluster\_plot | 1 |
| 1560 | GO:0042026 | protein refolding | biological\_process | 0.0 | 1.0 | 1.0 | 0 | cluster\_plot | 8 |
| 1561 | GO:0004375 | glycine dehydrogenase (decarboxylating) activity | molecular\_function | 0.0 | 1.0 | 1.0 | 0 | cluster\_plot | 3 |
| 1562 | GO:0046835 | carbohydrate phosphorylation | biological\_process | 0.0 | 1.0 | 1.0 | 0 | cluster\_plot | 1 |
| 1563 | GO:0030127 | COPII vesicle coat | cellular\_component | 0.0 | 1.0 | 1.0 | 0 | cluster\_plot | 24 |
| 1564 | GO:0004418 | hydroxymethylbilane synthase activity | molecular\_function | 0.0 | 1.0 | 1.0 | 0 | cluster\_plot | 1 |
| 1565 | GO:0006098 | pentose-phosphate shunt | biological\_process | 0.0 | 1.0 | 1.0 | 0 | cluster\_plot | 15 |
| 1566 | GO:0009891 | positive regulation of biosynthetic process | biological\_process | 0.0 | 1.0 | 1.0 | 0 | cluster\_plot | 7 |
| 1567 | GO:0044769 | ATPase activity, coupled to transmembrane movement of ions, rotational mechanism | molecular\_function | 0.0 | 1.0 | 1.0 | 0 | cluster\_plot | 33 |
| 1568 | GO:0019104 | DNA N-glycosylase activity | molecular\_function | 0.0 | 1.0 | 1.0 | 0 | cluster\_plot | 14 |
| 1569 | GO:0004673 | protein histidine kinase activity | molecular\_function | 0.0 | 1.0 | 1.0 | 0 | cluster\_plot | 25 |
| 1570 | GO:1902495 | transmembrane transporter complex | cellular\_component | 0.0 | 1.0 | 1.0 | 0 | cluster\_plot | 5 |
| 1571 | GO:0006085 | acetyl-CoA biosynthetic process | biological\_process | 0.0 | 1.0 | 1.0 | 0 | cluster\_plot | 2 |
| 1572 | GO:0001682 | tRNA 5'-leader removal | biological\_process | 0.0 | 1.0 | 1.0 | 0 | cluster\_plot | 2 |
| 1573 | GO:0017000 | antibiotic biosynthetic process | biological\_process | 0.0 | 1.0 | 1.0 | 0 | cluster\_plot | 1 |
| 1574 | GO:0046522 | S-methyl-5-thioribose kinase activity | molecular\_function | 0.0 | 1.0 | 1.0 | 0 | cluster\_plot | 4 |
| 1575 | GO:0004674 | protein serine/threonine kinase activity | molecular\_function | 0.0 | 1.0 | 1.0 | 0 | cluster\_plot | 38 |
| 1576 | GO:0045040 | protein import into mitochondrial outer membrane | biological\_process | 0.0 | 1.0 | 1.0 | 0 | cluster\_plot | 10 |
| 1577 | GO:0019898 | extrinsic component of membrane | cellular\_component | 0.0 | 1.0 | 1.0 | 0 | cluster\_plot | 29 |
| 1578 | GO:0004789 | thiamine-phosphate diphosphorylase activity | molecular\_function | 0.0 | 1.0 | 1.0 | 0 | cluster\_plot | 4 |
| 1579 | GO:0051205 | protein insertion into membrane | biological\_process | 0.0 | 1.0 | 1.0 | 0 | cluster\_plot | 7 |
| 1580 | GO:0042147 | retrograde transport, endosome to Golgi | biological\_process | 0.0 | 1.0 | 1.0 | 0 | cluster\_plot | 3 |
| 1581 | GO:0009154 | purine ribonucleotide catabolic process | biological\_process | 0.0 | 1.0 | 1.0 | 0 | cluster\_plot | 41 |
| 1582 | GO:0034440 | lipid oxidation | biological\_process | 0.0 | 1.0 | 1.0 | 0 | cluster\_plot | 7 |
| 1583 | GO:0072341 | modified amino acid binding | molecular\_function | 0.0 | 1.0 | 1.0 | 0 | cluster\_plot | 4 |
| 1584 | GO:0043243 | positive regulation of protein complex disassembly | biological\_process | 0.0 | 1.0 | 1.0 | 0 | cluster\_plot | 3 |
| 1585 | GO:1902562 | H4 histone acetyltransferase complex | cellular\_component | 0.0 | 1.0 | 1.0 | 0 | cluster\_plot | 2 |
| 1586 | GO:0016799 | hydrolase activity, hydrolyzing N-glycosyl compounds | molecular\_function | 0.0 | 1.0 | 1.0 | 0 | cluster\_plot | 22 |
| 1587 | GO:0001716 | L-amino-acid oxidase activity | molecular\_function | 0.0 | 1.0 | 1.0 | 0 | cluster\_plot | 2 |
| 1588 | GO:0006437 | tyrosyl-tRNA aminoacylation | biological\_process | 0.0 | 1.0 | 1.0 | 0 | cluster\_plot | 2 |
| 1589 | GO:0036440 | citrate synthase activity | molecular\_function | 0.0 | 1.0 | 1.0 | 0 | cluster\_plot | 2 |
| 1590 | GO:0006112 | energy reserve metabolic process | biological\_process | 0.0 | 1.0 | 1.0 | 0 | cluster\_plot | 3 |
| 1591 | GO:0004739 | pyruvate dehydrogenase (acetyl-transferring) activity | molecular\_function | 0.0 | 1.0 | 1.0 | 0 | cluster\_plot | 1 |
| 1592 | GO:0035437 | maintenance of protein localization in endoplasmic reticulum | biological\_process | 0.0 | 1.0 | 1.0 | 0 | cluster\_plot | 11 |
| 1593 | GO:0008839 | 4-hydroxy-tetrahydrodipicolinate reductase | molecular\_function | 0.0 | 1.0 | 1.0 | 0 | cluster\_plot | 3 |
| 1594 | GO:0006419 | alanyl-tRNA aminoacylation | biological\_process | 0.0 | 1.0 | 1.0 | 0 | cluster\_plot | 5 |
| 1595 | GO:0015991 | ATP hydrolysis coupled proton transport | biological\_process | 0.0 | 1.0 | 1.0 | 0 | cluster\_plot | 38 |
| 1596 | GO:0046487 | glyoxylate metabolic process | biological\_process | 0.0 | 1.0 | 1.0 | 0 | cluster\_plot | 3 |
| 1597 | GO:0033202 | DNA helicase complex | cellular\_component | 0.0 | 1.0 | 1.0 | 0 | cluster\_plot | 4 |
| 1598 | GO:0006586 | indolalkylamine metabolic process | biological\_process | 0.0 | 1.0 | 1.0 | 0 | cluster\_plot | 16 |
| 1599 | GO:0004746 | riboflavin synthase activity | molecular\_function | 0.0 | 1.0 | 1.0 | 0 | cluster\_plot | 3 |
| 1600 | GO:0006417 | regulation of translation | biological\_process | 0.0 | 1.0 | 1.0 | 0 | cluster\_plot | 15 |
| 1601 | GO:0000808 | origin recognition complex | cellular\_component | 0.0 | 1.0 | 1.0 | 0 | cluster\_plot | 9 |
| 1602 | GO:0000172 | ribonuclease MRP complex | cellular\_component | 0.0 | 1.0 | 1.0 | 0 | cluster\_plot | 1 |
| 1603 | GO:0016812 | hydrolase activity, acting on carbon-nitrogen (but not peptide) bonds, in cyclic amides | molecular\_function | 0.0 | 1.0 | 1.0 | 0 | cluster\_plot | 2 |
| 1604 | GO:0019204 | nucleotide phosphatase activity | molecular\_function | 0.0 | 1.0 | 1.0 | 0 | cluster\_plot | 1 |
| 1605 | GO:0006545 | glycine biosynthetic process | biological\_process | 0.0 | 1.0 | 1.0 | 0 | cluster\_plot | 1 |
| 1606 | GO:0004638 | phosphoribosylaminoimidazole carboxylase activity | molecular\_function | 0.0 | 1.0 | 1.0 | 0 | cluster\_plot | 2 |
| 1607 | GO:0046938 | phytochelatin biosynthetic process | biological\_process | 0.0 | 1.0 | 1.0 | 0 | cluster\_plot | 3 |
| 1608 | GO:0048522 | positive regulation of cellular process | biological\_process | 0.0 | 1.0 | 1.0 | 0 | cluster\_plot | 12 |
| 1609 | GO:0003729 | mRNA binding | molecular\_function | 0.0 | 1.0 | 1.0 | 0 | cluster\_plot | 3 |
| 1610 | GO:0006839 | mitochondrial transport | biological\_process | 0.0 | 1.0 | 1.0 | 0 | cluster\_plot | 19 |
| 1611 | GO:0004816 | asparagine-tRNA ligase activity | molecular\_function | 0.0 | 1.0 | 1.0 | 0 | cluster\_plot | 6 |
| 1612 | GO:0016992 | lipoate synthase activity | molecular\_function | 0.0 | 1.0 | 1.0 | 0 | cluster\_plot | 4 |
| 1613 | GO:0006783 | heme biosynthetic process | biological\_process | 0.0 | 1.0 | 1.0 | 0 | cluster\_plot | 8 |
| 1614 | GO:0031966 | mitochondrial membrane | cellular\_component | 0.0 | 1.0 | 1.0 | 0 | cluster\_plot | 29 |
| 1615 | GO:0004402 | histone acetyltransferase activity | molecular\_function | 0.0 | 1.0 | 1.0 | 0 | cluster\_plot | 8 |
| 1616 | GO:0070588 | calcium ion transmembrane transport | biological\_process | 0.0 | 1.0 | 1.0 | 0 | cluster\_plot | 24 |
| 1617 | GO:0007050 | cell cycle arrest | biological\_process | 0.0 | 1.0 | 1.0 | 0 | cluster\_plot | 8 |
| 1618 | GO:0004619 | phosphoglycerate mutase activity | molecular\_function | 0.0 | 1.0 | 1.0 | 0 | cluster\_plot | 2 |
| 1619 | GO:0005694 | chromosome | cellular\_component | 0.0 | 1.0 | 1.0 | 0 | cluster\_plot | 27 |
| 1620 | GO:0006177 | GMP biosynthetic process | biological\_process | 0.0 | 1.0 | 1.0 | 0 | cluster\_plot | 4 |
| 1621 | GO:0003871 | 5-methyltetrahydropteroyltriglutamate-homocysteine S-methyltransferase activity | molecular\_function | 0.0 | 1.0 | 1.0 | 0 | cluster\_plot | 3 |
| 1622 | GO:0045132 | meiotic chromosome segregation | biological\_process | 0.0 | 1.0 | 1.0 | 0 | cluster\_plot | 5 |
| 1623 | GO:0008290 | F-actin capping protein complex | cellular\_component | 0.0 | 1.0 | 1.0 | 0 | cluster\_plot | 6 |
| 1624 | GO:0035825 | reciprocal DNA recombination | biological\_process | 0.0 | 1.0 | 1.0 | 0 | cluster\_plot | 1 |
| 1625 | GO:0008565 | protein transporter activity | molecular\_function | 0.0 | 1.0 | 1.0 | 0 | cluster\_plot | 19 |
| 1626 | GO:0016725 | oxidoreductase activity, acting on CH or CH2 groups | molecular\_function | 0.0 | 1.0 | 1.0 | 0 | cluster\_plot | 3 |
| 1627 | GO:0016050 | vesicle organization | biological\_process | 0.0 | 1.0 | 1.0 | 0 | cluster\_plot | 2 |
| 1628 | GO:0005381 | iron ion transmembrane transporter activity | molecular\_function | 0.0 | 1.0 | 1.0 | 0 | cluster\_plot | 4 |
| 1629 | GO:0009158 | ribonucleoside monophosphate catabolic process | biological\_process | 0.0 | 1.0 | 1.0 | 0 | cluster\_plot | 19 |
| 1630 | GO:0016778 | diphosphotransferase activity | molecular\_function | 0.0 | 1.0 | 1.0 | 0 | cluster\_plot | 7 |
| 1631 | GO:0006818 | hydrogen transport | biological\_process | 0.0 | 1.0 | 1.0 | 0 | cluster\_plot | 83 |
| 1632 | GO:0008318 | protein prenyltransferase activity | molecular\_function | 0.0 | 1.0 | 1.0 | 0 | cluster\_plot | 4 |
| 1633 | GO:0009041 | uridylate kinase activity | molecular\_function | 0.0 | 1.0 | 1.0 | 0 | cluster\_plot | 2 |
| 1634 | GO:0008213 | protein alkylation | biological\_process | 0.0 | 1.0 | 1.0 | 0 | cluster\_plot | 26 |
| 1635 | GO:0000145 | exocyst | cellular\_component | 0.0 | 1.0 | 1.0 | 0 | cluster\_plot | 40 |
| 1636 | GO:0040029 | regulation of gene expression, epigenetic | biological\_process | 0.0 | 1.0 | 1.0 | 0 | cluster\_plot | 15 |
| 1637 | GO:0016054 | organic acid catabolic process | biological\_process | 0.0 | 1.0 | 1.0 | 0 | cluster\_plot | 37 |
| 1638 | GO:0008430 | selenium binding | molecular\_function | 0.0 | 1.0 | 1.0 | 0 | cluster\_plot | 4 |
| 1639 | GO:0050113 | inositol oxygenase activity | molecular\_function | 0.0 | 1.0 | 1.0 | 0 | cluster\_plot | 9 |
| 1640 | GO:0052862 | glucan endo-1,4-beta-glucanase activity, C-3 substituted reducing group | molecular\_function | 0.0 | 1.0 | 1.0 | 0 | cluster\_plot | 10 |
| 1641 | GO:0019136 | deoxynucleoside kinase activity | molecular\_function | 0.0 | 1.0 | 1.0 | 0 | cluster\_plot | 6 |
| 1642 | GO:0009306 | protein secretion | biological\_process | 0.0 | 1.0 | 1.0 | 0 | cluster\_plot | 1 |
| 1643 | GO:0006525 | arginine metabolic process | biological\_process | 0.0 | 1.0 | 1.0 | 0 | cluster\_plot | 19 |
| 1644 | GO:0008792 | arginine decarboxylase activity | molecular\_function | 0.0 | 1.0 | 1.0 | 0 | cluster\_plot | 1 |
| 1645 | GO:0043228 | non-membrane-bounded organelle | cellular\_component | 0.0 | 1.0 | 1.0 | 0 | cluster\_plot | 485 |
| 1646 | GO:0061135 | endopeptidase regulator activity | molecular\_function | 0.0 | 1.0 | 1.0 | 0 | cluster\_plot | 22 |
| 1647 | GO:0009066 | aspartate family amino acid metabolic process | biological\_process | 0.0 | 1.0 | 1.0 | 0 | cluster\_plot | 37 |
| 1648 | GO:0009311 | oligosaccharide metabolic process | biological\_process | 0.0 | 1.0 | 1.0 | 0 | cluster\_plot | 62 |
| 1649 | GO:0004325 | ferrochelatase activity | molecular\_function | 0.0 | 1.0 | 1.0 | 0 | cluster\_plot | 5 |
| 1650 | GO:0007275 | multicellular organismal development | biological\_process | 0.0 | 1.0 | 1.0 | 0 | cluster\_plot | 16 |
| 1651 | GO:0050832 | defense response to fungus | biological\_process | 0.0 | 1.0 | 1.0 | 0 | cluster\_plot | 1 |
| 1652 | GO:0018197 | peptidyl-aspartic acid modification | biological\_process | 0.0 | 1.0 | 1.0 | 0 | cluster\_plot | 1 |
| 1653 | GO:0051493 | regulation of cytoskeleton organization | biological\_process | 0.0 | 1.0 | 1.0 | 0 | cluster\_plot | 10 |
| 1654 | GO:0042316 | penicillin metabolic process | biological\_process | 0.0 | 1.0 | 1.0 | 0 | cluster\_plot | 1 |
| 1655 | GO:0036338 | viral membrane | cellular\_component | 0.0 | 1.0 | 1.0 | 0 | cluster\_plot | 1 |
| 1656 | GO:0016667 | oxidoreductase activity, acting on a sulfur group of donors | molecular\_function | 0.0 | 1.0 | 1.0 | 0 | cluster\_plot | 110 |
| 1657 | GO:0030695 | GTPase regulator activity | molecular\_function | 0.0 | 1.0 | 1.0 | 0 | cluster\_plot | 65 |
| 1658 | GO:0042545 | cell wall modification | biological\_process | 0.0 | 1.0 | 1.0 | 0 | cluster\_plot | 89 |
| 1659 | GO:0098518 | polynucleotide phosphatase activity | molecular\_function | 0.0 | 1.0 | 1.0 | 0 | cluster\_plot | 1 |
| 1660 | GO:0008184 | glycogen phosphorylase activity | molecular\_function | 0.0 | 1.0 | 1.0 | 0 | cluster\_plot | 4 |
| 1661 | GO:0004329 | formate-tetrahydrofolate ligase activity | molecular\_function | 0.0 | 1.0 | 1.0 | 0 | cluster\_plot | 2 |
| 1662 | GO:0051649 | establishment of localization in cell | biological\_process | 0.0 | 1.0 | 1.0 | 0 | cluster\_plot | 283 |
| 1663 | GO:0016846 | carbon-sulfur lyase activity | molecular\_function | 0.0 | 1.0 | 1.0 | 0 | cluster\_plot | 20 |
| 1664 | GO:0030832 | regulation of actin filament length | biological\_process | 0.0 | 1.0 | 1.0 | 0 | cluster\_plot | 10 |
| 1665 | GO:0042451 | purine nucleoside biosynthetic process | biological\_process | 0.0 | 1.0 | 1.0 | 0 | cluster\_plot | 72 |
| 1666 | GO:0009082 | branched-chain amino acid biosynthetic process | biological\_process | 0.0 | 1.0 | 1.0 | 0 | cluster\_plot | 17 |
| 1667 | GO:0043623 | cellular protein complex assembly | biological\_process | 0.0 | 1.0 | 1.0 | 0 | cluster\_plot | 30 |
| 1668 | GO:0042171 | lysophosphatidic acid acyltransferase activity | molecular\_function | 0.0 | 1.0 | 1.0 | 0 | cluster\_plot | 2 |
| 1669 | GO:0006436 | tryptophanyl-tRNA aminoacylation | biological\_process | 0.0 | 1.0 | 1.0 | 0 | cluster\_plot | 2 |
| 1670 | GO:0045252 | oxoglutarate dehydrogenase complex | cellular\_component | 0.0 | 1.0 | 1.0 | 0 | cluster\_plot | 3 |
| 1671 | GO:0046294 | formaldehyde catabolic process | biological\_process | 0.0 | 1.0 | 1.0 | 0 | cluster\_plot | 2 |
| 1672 | GO:0004143 | diacylglycerol kinase activity | molecular\_function | 0.0 | 1.0 | 1.0 | 0 | cluster\_plot | 17 |
| 1673 | GO:0003952 | NAD+ synthase (glutamine-hydrolyzing) activity | molecular\_function | 0.0 | 1.0 | 1.0 | 0 | cluster\_plot | 2 |
| 1674 | GO:0017004 | cytochrome complex assembly | biological\_process | 0.0 | 1.0 | 1.0 | 0 | cluster\_plot | 5 |
| 1675 | GO:0009267 | cellular response to starvation | biological\_process | 0.0 | 1.0 | 1.0 | 0 | cluster\_plot | 2 |
| 1676 | GO:0070925 | organelle assembly | biological\_process | 0.0 | 1.0 | 1.0 | 0 | cluster\_plot | 4 |
| 1677 | GO:0051051 | negative regulation of transport | biological\_process | 0.0 | 1.0 | 1.0 | 0 | cluster\_plot | 2 |
| 1678 | GO:0005667 | transcription factor complex | cellular\_component | 0.0 | 1.0 | 1.0 | 0 | cluster\_plot | 24 |
| 1679 | GO:0000228 | nuclear chromosome | cellular\_component | 0.0 | 1.0 | 1.0 | 0 | cluster\_plot | 7 |
| 1680 | GO:0005840 | ribosome | cellular\_component | 0.0 | 1.0 | 1.0 | 0 | cluster\_plot | 417 |
| 1681 | GO:0030623 | U5 snRNA binding | molecular\_function | 0.0 | 1.0 | 1.0 | 0 | cluster\_plot | 1 |
| 1682 | GO:0003684 | damaged DNA binding | molecular\_function | 0.0 | 1.0 | 1.0 | 0 | cluster\_plot | 24 |
| 1683 | GO:0043241 | protein complex disassembly | biological\_process | 0.0 | 1.0 | 1.0 | 0 | cluster\_plot | 13 |
| 1684 | GO:0003713 | transcription coactivator activity | molecular\_function | 0.0 | 1.0 | 1.0 | 0 | cluster\_plot | 5 |
| 1685 | GO:0000301 | retrograde transport, vesicle recycling within Golgi | biological\_process | 0.0 | 1.0 | 1.0 | 0 | cluster\_plot | 3 |
| 1686 | GO:0006370 | 7-methylguanosine mRNA capping | biological\_process | 0.0 | 1.0 | 1.0 | 0 | cluster\_plot | 3 |
| 1687 | GO:0009415 | response to water | biological\_process | 0.0 | 1.0 | 1.0 | 0 | cluster\_plot | 7 |
| 1688 | GO:0008519 | ammonium transmembrane transporter activity | molecular\_function | 0.0 | 1.0 | 1.0 | 0 | cluster\_plot | 18 |
| 1689 | GO:0006720 | isoprenoid metabolic process | biological\_process | 0.0 | 1.0 | 1.0 | 0 | cluster\_plot | 40 |
| 1690 | GO:0004496 | mevalonate kinase activity | molecular\_function | 0.0 | 1.0 | 1.0 | 0 | cluster\_plot | 1 |
| 1691 | GO:0006040 | amino sugar metabolic process | biological\_process | 0.0 | 1.0 | 1.0 | 0 | cluster\_plot | 21 |
| 1692 | GO:0006672 | ceramide metabolic process | biological\_process | 0.0 | 1.0 | 1.0 | 0 | cluster\_plot | 3 |
| 1693 | GO:0005047 | signal recognition particle binding | molecular\_function | 0.0 | 1.0 | 1.0 | 0 | cluster\_plot | 3 |
| 1694 | GO:0006071 | glycerol metabolic process | biological\_process | 0.0 | 1.0 | 1.0 | 0 | cluster\_plot | 19 |
| 1695 | GO:0006909 | phagocytosis | biological\_process | 0.0 | 1.0 | 1.0 | 0 | cluster\_plot | 6 |
| 1696 | GO:0007186 | G-protein coupled receptor signaling pathway | biological\_process | 0.0 | 1.0 | 1.0 | 0 | cluster\_plot | 32 |
| 1697 | GO:0033015 | tetrapyrrole catabolic process | biological\_process | 0.0 | 1.0 | 1.0 | 0 | cluster\_plot | 7 |
| 1698 | GO:0000956 | nuclear-transcribed mRNA catabolic process | biological\_process | 0.0 | 1.0 | 1.0 | 0 | cluster\_plot | 3 |
| 1699 | GO:0009314 | response to radiation | biological\_process | 0.0 | 1.0 | 1.0 | 0 | cluster\_plot | 8 |
| 1700 | GO:0022411 | cellular component disassembly | biological\_process | 0.0 | 1.0 | 1.0 | 0 | cluster\_plot | 13 |
| 1701 | GO:0006438 | valyl-tRNA aminoacylation | biological\_process | 0.0 | 1.0 | 1.0 | 0 | cluster\_plot | 2 |
| 1702 | GO:0016627 | oxidoreductase activity, acting on the CH-CH group of donors | molecular\_function | 0.0 | 1.0 | 1.0 | 0 | cluster\_plot | 54 |
| 1703 | GO:0043631 | RNA polyadenylation | biological\_process | 0.0 | 1.0 | 1.0 | 0 | cluster\_plot | 12 |
| 1704 | GO:0005337 | nucleoside transmembrane transporter activity | molecular\_function | 0.0 | 1.0 | 1.0 | 0 | cluster\_plot | 9 |
| 1705 | GO:0006891 | intra-Golgi vesicle-mediated transport | biological\_process | 0.0 | 1.0 | 1.0 | 0 | cluster\_plot | 7 |
| 1706 | GO:0019184 | nonribosomal peptide biosynthetic process | biological\_process | 0.0 | 1.0 | 1.0 | 0 | cluster\_plot | 7 |
| 1707 | GO:0043545 | molybdopterin cofactor metabolic process | biological\_process | 0.0 | 1.0 | 1.0 | 0 | cluster\_plot | 10 |
| 1708 | GO:0016423 | tRNA (guanine) methyltransferase activity | molecular\_function | 0.0 | 1.0 | 1.0 | 0 | cluster\_plot | 8 |
| 1709 | GO:0006030 | chitin metabolic process | biological\_process | 0.0 | 1.0 | 1.0 | 0 | cluster\_plot | 18 |
| 1710 | GO:0009231 | riboflavin biosynthetic process | biological\_process | 0.0 | 1.0 | 1.0 | 0 | cluster\_plot | 13 |
| 1711 | GO:0009443 | pyridoxal 5'-phosphate salvage | biological\_process | 0.0 | 1.0 | 1.0 | 0 | cluster\_plot | 1 |
| 1712 | GO:0016126 | sterol biosynthetic process | biological\_process | 0.0 | 1.0 | 1.0 | 0 | cluster\_plot | 3 |
| 1713 | GO:0006605 | protein targeting | biological\_process | 0.0 | 1.0 | 1.0 | 0 | cluster\_plot | 31 |
| 1714 | GO:0048610 | cellular process involved in reproduction | biological\_process | 0.0 | 1.0 | 1.0 | 0 | cluster\_plot | 121 |
| 1715 | GO:0006570 | tyrosine metabolic process | biological\_process | 0.0 | 1.0 | 1.0 | 0 | cluster\_plot | 5 |
| 1716 | GO:0019239 | deaminase activity | molecular\_function | 0.0 | 1.0 | 1.0 | 0 | cluster\_plot | 11 |
| 1717 | GO:0016161 | beta-amylase activity | molecular\_function | 0.0 | 1.0 | 1.0 | 0 | cluster\_plot | 31 |
| 1718 | GO:0003777 | microtubule motor activity | molecular\_function | 0.0 | 1.0 | 1.0 | 0 | cluster\_plot | 97 |
| 1719 | GO:0046149 | pigment catabolic process | biological\_process | 0.0 | 1.0 | 1.0 | 0 | cluster\_plot | 7 |
| 1720 | GO:0006140 | regulation of nucleotide metabolic process | biological\_process | 0.0 | 1.0 | 1.0 | 0 | cluster\_plot | 54 |
| 1721 | GO:0006450 | regulation of translational fidelity | biological\_process | 0.0 | 1.0 | 1.0 | 0 | cluster\_plot | 2 |
| 1722 | GO:0006768 | biotin metabolic process | biological\_process | 0.0 | 1.0 | 1.0 | 0 | cluster\_plot | 2 |
| 1723 | GO:0004221 | ubiquitin thiolesterase activity | molecular\_function | 0.0 | 1.0 | 1.0 | 0 | cluster\_plot | 66 |
| 1724 | GO:0006434 | seryl-tRNA aminoacylation | biological\_process | 0.0 | 1.0 | 1.0 | 0 | cluster\_plot | 3 |
| 1725 | GO:0016459 | myosin complex | cellular\_component | 0.0 | 1.0 | 1.0 | 0 | cluster\_plot | 20 |
| 1726 | GO:0071451 | cellular response to superoxide | biological\_process | 0.0 | 1.0 | 1.0 | 0 | cluster\_plot | 1 |
| 1727 | GO:0006289 | nucleotide-excision repair | biological\_process | 0.0 | 1.0 | 1.0 | 0 | cluster\_plot | 20 |
| 1728 | GO:0009218 | pyrimidine ribonucleotide metabolic process | biological\_process | 0.0 | 1.0 | 1.0 | 0 | cluster\_plot | 12 |
| 1729 | GO:0016651 | oxidoreductase activity, acting on NAD(P)H | molecular\_function | 0.0 | 1.0 | 1.0 | 0 | cluster\_plot | 48 |
| 1730 | GO:0006787 | porphyrin-containing compound catabolic process | biological\_process | 0.0 | 1.0 | 1.0 | 0 | cluster\_plot | 7 |
| 1731 | GO:0004867 | serine-type endopeptidase inhibitor activity | molecular\_function | 0.0 | 1.0 | 1.0 | 0 | cluster\_plot | 8 |
| 1732 | GO:0008937 | ferredoxin-NAD(P) reductase activity | molecular\_function | 0.0 | 1.0 | 1.0 | 0 | cluster\_plot | 1 |
| 1733 | GO:0006152 | purine nucleoside catabolic process | biological\_process | 0.0 | 1.0 | 1.0 | 0 | cluster\_plot | 41 |
| 1734 | GO:0015662 | ATPase activity, coupled to transmembrane movement of ions, phosphorylative mechanism | molecular\_function | 0.0 | 1.0 | 1.0 | 0 | cluster\_plot | 27 |
| 1735 | GO:0051052 | regulation of DNA metabolic process | biological\_process | 0.0 | 1.0 | 1.0 | 0 | cluster\_plot | 11 |
| 1736 | GO:0016311 | dephosphorylation | biological\_process | 0.0 | 1.0 | 1.0 | 0 | cluster\_plot | 41 |
| 1737 | GO:0006862 | nucleotide transport | biological\_process | 0.0 | 1.0 | 1.0 | 0 | cluster\_plot | 9 |
| 1738 | GO:0015211 | purine nucleoside transmembrane transporter activity | molecular\_function | 0.0 | 1.0 | 1.0 | 0 | cluster\_plot | 1 |
| 1739 | GO:0090305 | nucleic acid phosphodiester bond hydrolysis | biological\_process | 0.0 | 1.0 | 1.0 | 0 | cluster\_plot | 1 |
| 1740 | GO:0008378 | galactosyltransferase activity | molecular\_function | 0.0 | 1.0 | 1.0 | 0 | cluster\_plot | 35 |
| 1741 | GO:0006730 | one-carbon metabolic process | biological\_process | 0.0 | 1.0 | 1.0 | 0 | cluster\_plot | 2 |
| 1742 | GO:0006558 | L-phenylalanine metabolic process | biological\_process | 0.0 | 1.0 | 1.0 | 0 | cluster\_plot | 12 |
| 1743 | GO:0007010 | cytoskeleton organization | biological\_process | 0.0 | 1.0 | 1.0 | 0 | cluster\_plot | 45 |
| 1744 | GO:0008138 | protein tyrosine/serine/threonine phosphatase activity | molecular\_function | 0.0 | 1.0 | 1.0 | 0 | cluster\_plot | 31 |
| 1745 | GO:0005663 | DNA replication factor C complex | cellular\_component | 0.0 | 1.0 | 1.0 | 0 | cluster\_plot | 5 |
| 1746 | GO:0030904 | retromer complex | cellular\_component | 0.0 | 1.0 | 1.0 | 0 | cluster\_plot | 2 |
| 1747 | GO:0003756 | protein disulfide isomerase activity | molecular\_function | 0.0 | 1.0 | 1.0 | 0 | cluster\_plot | 1 |
| 1748 | GO:0015205 | nucleobase transmembrane transporter activity | molecular\_function | 0.0 | 1.0 | 1.0 | 0 | cluster\_plot | 1 |
| 1749 | GO:1901136 | carbohydrate derivative catabolic process | biological\_process | 0.0 | 1.0 | 1.0 | 0 | cluster\_plot | 68 |
| 1750 | GO:0030337 | DNA polymerase processivity factor activity | molecular\_function | 0.0 | 1.0 | 1.0 | 0 | cluster\_plot | 3 |
| 1751 | GO:0070569 | uridylyltransferase activity | molecular\_function | 0.0 | 1.0 | 1.0 | 0 | cluster\_plot | 1 |
| 1752 | GO:0006559 | L-phenylalanine catabolic process | biological\_process | 0.0 | 1.0 | 1.0 | 0 | cluster\_plot | 4 |
| 1753 | GO:0043549 | regulation of kinase activity | biological\_process | 0.0 | 1.0 | 1.0 | 0 | cluster\_plot | 11 |
| 1754 | GO:0008610 | lipid biosynthetic process | biological\_process | 0.0 | 1.0 | 1.0 | 0 | cluster\_plot | 246 |
| 1755 | GO:0003954 | NADH dehydrogenase activity | molecular\_function | 0.0 | 1.0 | 1.0 | 0 | cluster\_plot | 9 |
| 1756 | GO:0016646 | oxidoreductase activity, acting on the CH-NH group of donors, NAD or NADP as acceptor | molecular\_function | 0.0 | 1.0 | 1.0 | 0 | cluster\_plot | 9 |
| 1757 | GO:0009124 | nucleoside monophosphate biosynthetic process | biological\_process | 0.0 | 1.0 | 1.0 | 0 | cluster\_plot | 77 |
| 1758 | GO:0098542 | defense response to other organism | biological\_process | 0.0 | 1.0 | 1.0 | 0 | cluster\_plot | 1 |
| 1759 | GO:0005751 | mitochondrial respiratory chain complex IV | cellular\_component | 0.0 | 1.0 | 1.0 | 0 | cluster\_plot | 5 |
| 1760 | GO:0009127 | purine nucleoside monophosphate biosynthetic process | biological\_process | 0.0 | 1.0 | 1.0 | 0 | cluster\_plot | 71 |
| 1761 | GO:0051129 | negative regulation of cellular component organization | biological\_process | 0.0 | 1.0 | 1.0 | 0 | cluster\_plot | 2 |
| 1762 | GO:0042773 | ATP synthesis coupled electron transport | biological\_process | 0.0 | 1.0 | 1.0 | 0 | cluster\_plot | 2 |
| 1763 | GO:0006334 | nucleosome assembly | biological\_process | 0.0 | 1.0 | 1.0 | 0 | cluster\_plot | 29 |
| 1764 | GO:0005048 | signal sequence binding | molecular\_function | 0.0 | 1.0 | 1.0 | 0 | cluster\_plot | 12 |
| 1765 | GO:0072528 | pyrimidine-containing compound biosynthetic process | biological\_process | 0.0 | 1.0 | 1.0 | 0 | cluster\_plot | 38 |
| 1766 | GO:0003904 | deoxyribodipyrimidine photo-lyase activity | molecular\_function | 0.0 | 1.0 | 1.0 | 0 | cluster\_plot | 1 |
| 1767 | GO:0004107 | chorismate synthase activity | molecular\_function | 0.0 | 1.0 | 1.0 | 0 | cluster\_plot | 5 |
| 1768 | GO:0009536 | plastid | cellular\_component | 0.0 | 1.0 | 1.0 | 0 | cluster\_plot | 21 |
| 1769 | GO:0016469 | proton-transporting two-sector ATPase complex | cellular\_component | 0.0 | 1.0 | 1.0 | 0 | cluster\_plot | 7 |
| 1770 | GO:0003984 | acetolactate synthase activity | molecular\_function | 0.0 | 1.0 | 1.0 | 0 | cluster\_plot | 5 |
| 1771 | GO:0015605 | organophosphate ester transmembrane transporter activity | molecular\_function | 0.0 | 1.0 | 1.0 | 0 | cluster\_plot | 10 |
| 1772 | GO:0003735 | structural constituent of ribosome | molecular\_function | 0.0 | 1.0 | 1.0 | 0 | cluster\_plot | 445 |
| 1773 | GO:0009448 | gamma-aminobutyric acid metabolic process | biological\_process | 0.0 | 1.0 | 1.0 | 0 | cluster\_plot | 2 |
| 1774 | GO:0003995 | acyl-CoA dehydrogenase activity | molecular\_function | 0.0 | 1.0 | 1.0 | 0 | cluster\_plot | 16 |
| 1775 | GO:0004298 | threonine-type endopeptidase activity | molecular\_function | 0.0 | 1.0 | 1.0 | 0 | cluster\_plot | 42 |
| 1776 | GO:0005509 | calcium ion binding | molecular\_function | 0.0 | 1.0 | 1.0 | 0 | cluster\_plot | 246 |
| 1777 | GO:0009201 | ribonucleoside triphosphate biosynthetic process | biological\_process | 0.0 | 1.0 | 1.0 | 0 | cluster\_plot | 56 |
| 1778 | GO:0004733 | pyridoxamine-phosphate oxidase activity | molecular\_function | 0.0 | 1.0 | 1.0 | 0 | cluster\_plot | 1 |
| 1779 | GO:0004872 | receptor activity | molecular\_function | 0.0 | 1.0 | 1.0 | 0 | cluster\_plot | 67 |
| 1780 | GO:0060249 | anatomical structure homeostasis | biological\_process | 0.0 | 1.0 | 1.0 | 0 | cluster\_plot | 3 |
| 1781 | GO:0044273 | sulfur compound catabolic process | biological\_process | 0.0 | 1.0 | 1.0 | 0 | cluster\_plot | 1 |
| 1782 | GO:0017009 | protein-phycocyanobilin linkage | biological\_process | 0.0 | 1.0 | 1.0 | 0 | cluster\_plot | 2 |
| 1783 | GO:0008219 | cell death | biological\_process | 0.0 | 1.0 | 1.0 | 0 | cluster\_plot | 1 |
| 1784 | GO:0047661 | amino-acid racemase activity | molecular\_function | 0.0 | 1.0 | 1.0 | 0 | cluster\_plot | 3 |
| 1785 | GO:0006749 | glutathione metabolic process | biological\_process | 0.0 | 1.0 | 1.0 | 0 | cluster\_plot | 15 |
| 1786 | GO:0008169 | C-methyltransferase activity | molecular\_function | 0.0 | 1.0 | 1.0 | 0 | cluster\_plot | 2 |
| 1787 | GO:0009197 | pyrimidine deoxyribonucleoside diphosphate biosynthetic process | biological\_process | 0.0 | 1.0 | 1.0 | 0 | cluster\_plot | 2 |
| 1788 | GO:0004643 | phosphoribosylaminoimidazolecarboxamide formyltransferase activity | molecular\_function | 0.0 | 1.0 | 1.0 | 0 | cluster\_plot | 7 |
| 1789 | GO:0050793 | regulation of developmental process | biological\_process | 0.0 | 1.0 | 1.0 | 0 | cluster\_plot | 5 |
| 1790 | GO:0004640 | phosphoribosylanthranilate isomerase activity | molecular\_function | 0.0 | 1.0 | 1.0 | 0 | cluster\_plot | 1 |
| 1791 | GO:0008250 | oligosaccharyltransferase complex | cellular\_component | 0.0 | 1.0 | 1.0 | 0 | cluster\_plot | 3 |
| 1792 | GO:0044275 | cellular carbohydrate catabolic process | biological\_process | 0.0 | 1.0 | 1.0 | 0 | cluster\_plot | 12 |
| 1793 | GO:0004185 | serine-type carboxypeptidase activity | molecular\_function | 0.0 | 1.0 | 1.0 | 0 | cluster\_plot | 46 |
| 1794 | GO:0005347 | ATP transmembrane transporter activity | molecular\_function | 0.0 | 1.0 | 1.0 | 0 | cluster\_plot | 1 |
| 1795 | GO:0042816 | vitamin B6 metabolic process | biological\_process | 0.0 | 1.0 | 1.0 | 0 | cluster\_plot | 1 |
| 1796 | GO:0004657 | proline dehydrogenase activity | molecular\_function | 0.0 | 1.0 | 1.0 | 0 | cluster\_plot | 4 |
| 1797 | GO:0006122 | mitochondrial electron transport, ubiquinol to cytochrome c | biological\_process | 0.0 | 1.0 | 1.0 | 0 | cluster\_plot | 5 |
| 1798 | GO:0006195 | purine nucleotide catabolic process | biological\_process | 0.0 | 1.0 | 1.0 | 0 | cluster\_plot | 41 |
| 1799 | GO:0042822 | pyridoxal phosphate metabolic process | biological\_process | 0.0 | 1.0 | 1.0 | 0 | cluster\_plot | 7 |
| 1800 | GO:0000179 | rRNA (adenine-N6,N6-)-dimethyltransferase activity | molecular\_function | 0.0 | 1.0 | 1.0 | 0 | cluster\_plot | 3 |
| 1801 | GO:0003942 | N-acetyl-gamma-glutamyl-phosphate reductase activity | molecular\_function | 0.0 | 1.0 | 1.0 | 0 | cluster\_plot | 7 |
| 1802 | GO:0036361 | racemase activity, acting on amino acids and derivatives | molecular\_function | 0.0 | 1.0 | 1.0 | 0 | cluster\_plot | 5 |
| 1803 | GO:0070603 | SWI/SNF superfamily-type complex | cellular\_component | 0.0 | 1.0 | 1.0 | 0 | cluster\_plot | 4 |
| 1804 | GO:0006449 | regulation of translational termination | biological\_process | 0.0 | 1.0 | 1.0 | 0 | cluster\_plot | 3 |
| 1805 | GO:0004822 | isoleucine-tRNA ligase activity | molecular\_function | 0.0 | 1.0 | 1.0 | 0 | cluster\_plot | 4 |
| 1806 | GO:0004044 | amidophosphoribosyltransferase activity | molecular\_function | 0.0 | 1.0 | 1.0 | 0 | cluster\_plot | 5 |
| 1807 | GO:0005402 | cation:sugar symporter activity | molecular\_function | 0.0 | 1.0 | 1.0 | 0 | cluster\_plot | 12 |
| 1808 | GO:0004725 | protein tyrosine phosphatase activity | molecular\_function | 0.0 | 1.0 | 1.0 | 0 | cluster\_plot | 9 |
| 1809 | GO:0045229 | external encapsulating structure organization | biological\_process | 0.0 | 1.0 | 1.0 | 0 | cluster\_plot | 90 |
| 1810 | GO:0004470 | malic enzyme activity | molecular\_function | 0.0 | 1.0 | 1.0 | 0 | cluster\_plot | 14 |
| 1811 | GO:0046416 | D-amino acid metabolic process | biological\_process | 0.0 | 1.0 | 1.0 | 0 | cluster\_plot | 1 |
| 1812 | GO:0004832 | valine-tRNA ligase activity | molecular\_function | 0.0 | 1.0 | 1.0 | 0 | cluster\_plot | 2 |
| 1813 | GO:0016878 | acid-thiol ligase activity | molecular\_function | 0.0 | 1.0 | 1.0 | 0 | cluster\_plot | 2 |
| 1814 | GO:0051651 | maintenance of location in cell | biological\_process | 0.0 | 1.0 | 1.0 | 0 | cluster\_plot | 13 |
| 1815 | GO:0044444 | cytoplasmic part | cellular\_component | 0.0 | 1.0 | 1.0 | 0 | cluster\_plot | 907 |
| 1816 | GO:0004400 | histidinol-phosphate transaminase activity | molecular\_function | 0.0 | 1.0 | 1.0 | 0 | cluster\_plot | 4 |
| 1817 | GO:0016791 | phosphatase activity | molecular\_function | 0.0 | 1.0 | 1.0 | 0 | cluster\_plot | 113 |
| 1818 | GO:0016458 | gene silencing | biological\_process | 0.0 | 1.0 | 1.0 | 0 | cluster\_plot | 11 |
| 1819 | GO:0036459 | ubiquitinyl hydrolase activity | molecular\_function | 0.0 | 1.0 | 1.0 | 0 | cluster\_plot | 66 |
| 1820 | GO:0071826 | ribonucleoprotein complex subunit organization | biological\_process | 0.0 | 1.0 | 1.0 | 0 | cluster\_plot | 2 |
| 1821 | GO:0032955 | regulation of barrier septum assembly | biological\_process | 0.0 | 1.0 | 1.0 | 0 | cluster\_plot | 1 |
| 1822 | GO:0044445 | cytosolic part | cellular\_component | 0.0 | 1.0 | 1.0 | 0 | cluster\_plot | 23 |
| 1823 | GO:0003933 | GTP cyclohydrolase activity | molecular\_function | 0.0 | 1.0 | 1.0 | 0 | cluster\_plot | 4 |
| 1824 | GO:0018193 | peptidyl-amino acid modification | biological\_process | 0.0 | 1.0 | 1.0 | 0 | cluster\_plot | 16 |
| 1825 | GO:0004721 | phosphoprotein phosphatase activity | molecular\_function | 0.0 | 1.0 | 1.0 | 0 | cluster\_plot | 49 |
| 1826 | GO:0048038 | quinone binding | molecular\_function | 0.0 | 1.0 | 1.0 | 0 | cluster\_plot | 24 |
| 1827 | GO:0046073 | dTMP metabolic process | biological\_process | 0.0 | 1.0 | 1.0 | 0 | cluster\_plot | 1 |
| 1828 | GO:0004491 | methylmalonate-semialdehyde dehydrogenase (acylating) activity | molecular\_function | 0.0 | 1.0 | 1.0 | 0 | cluster\_plot | 1 |
| 1829 | GO:0005230 | extracellular ligand-gated ion channel activity | molecular\_function | 0.0 | 1.0 | 1.0 | 0 | cluster\_plot | 37 |
| 1830 | GO:0006323 | DNA packaging | biological\_process | 0.0 | 1.0 | 1.0 | 0 | cluster\_plot | 1 |
| 1831 | GO:0034450 | ubiquitin-ubiquitin ligase activity | molecular\_function | 0.0 | 1.0 | 1.0 | 0 | cluster\_plot | 4 |
| 1832 | GO:0047623 | adenosine-phosphate deaminase activity | molecular\_function | 0.0 | 1.0 | 1.0 | 0 | cluster\_plot | 3 |
| 1833 | GO:0003964 | RNA-directed DNA polymerase activity | molecular\_function | 0.0 | 1.0 | 1.0 | 0 | cluster\_plot | 10 |
| 1834 | GO:0006481 | C-terminal protein methylation | biological\_process | 0.0 | 1.0 | 1.0 | 0 | cluster\_plot | 1 |
| 1835 | GO:0006913 | nucleocytoplasmic transport | biological\_process | 0.0 | 1.0 | 1.0 | 0 | cluster\_plot | 9 |
| 1836 | GO:0045185 | maintenance of protein location | biological\_process | 0.0 | 1.0 | 1.0 | 0 | cluster\_plot | 13 |
| 1837 | GO:0000075 | cell cycle checkpoint | biological\_process | 0.0 | 1.0 | 1.0 | 0 | cluster\_plot | 4 |
| 1838 | GO:0006470 | protein dephosphorylation | biological\_process | 0.0 | 1.0 | 1.0 | 0 | cluster\_plot | 40 |
| 1839 | GO:0007059 | chromosome segregation | biological\_process | 0.0 | 1.0 | 1.0 | 0 | cluster\_plot | 6 |
| 1840 | GO:0006302 | double-strand break repair | biological\_process | 0.0 | 1.0 | 1.0 | 0 | cluster\_plot | 1 |
| 1841 | GO:0006099 | tricarboxylic acid cycle | biological\_process | 0.0 | 1.0 | 1.0 | 0 | cluster\_plot | 25 |
| 1842 | GO:0004869 | cysteine-type endopeptidase inhibitor activity | molecular\_function | 0.0 | 1.0 | 1.0 | 0 | cluster\_plot | 11 |
| 1843 | GO:0006595 | polyamine metabolic process | biological\_process | 0.0 | 1.0 | 1.0 | 0 | cluster\_plot | 8 |
| 1844 | GO:0046292 | formaldehyde metabolic process | biological\_process | 0.0 | 1.0 | 1.0 | 0 | cluster\_plot | 2 |
| 1845 | GO:0006397 | mRNA processing | biological\_process | 0.0 | 1.0 | 1.0 | 0 | cluster\_plot | 48 |
| 1846 | GO:0016782 | transferase activity, transferring sulfur-containing groups | molecular\_function | 0.0 | 1.0 | 1.0 | 0 | cluster\_plot | 17 |
| 1847 | GO:0006576 | cellular biogenic amine metabolic process | biological\_process | 0.0 | 1.0 | 1.0 | 0 | cluster\_plot | 26 |
| 1848 | GO:0006767 | water-soluble vitamin metabolic process | biological\_process | 0.0 | 1.0 | 1.0 | 0 | cluster\_plot | 34 |
| 1849 | GO:0044205 | 'de novo' UMP biosynthetic process | biological\_process | 0.0 | 1.0 | 1.0 | 0 | cluster\_plot | 1 |
| 1850 | GO:0034314 | Arp2/3 complex-mediated actin nucleation | biological\_process | 0.0 | 1.0 | 1.0 | 0 | cluster\_plot | 4 |
| 1851 | GO:0030071 | regulation of mitotic metaphase/anaphase transition | biological\_process | 0.0 | 1.0 | 1.0 | 0 | cluster\_plot | 5 |
| 1852 | GO:0006413 | translational initiation | biological\_process | 0.0 | 1.0 | 1.0 | 0 | cluster\_plot | 40 |
| 1853 | GO:0015074 | DNA integration | biological\_process | 0.0 | 1.0 | 1.0 | 0 | cluster\_plot | 3 |
| 1854 | GO:0004809 | tRNA (guanine-N2-)-methyltransferase activity | molecular\_function | 0.0 | 1.0 | 1.0 | 0 | cluster\_plot | 4 |
| 1855 | GO:0006873 | cellular ion homeostasis | biological\_process | 0.0 | 1.0 | 1.0 | 0 | cluster\_plot | 6 |
| 1856 | GO:0004489 | methylenetetrahydrofolate reductase (NAD(P)H) activity | molecular\_function | 0.0 | 1.0 | 1.0 | 0 | cluster\_plot | 1 |
| 1857 | GO:0006383 | transcription from RNA polymerase III promoter | biological\_process | 0.0 | 1.0 | 1.0 | 0 | cluster\_plot | 6 |
| 1858 | GO:0048033 | heme o metabolic process | biological\_process | 0.0 | 1.0 | 1.0 | 0 | cluster\_plot | 2 |
| 1859 | GO:0005777 | peroxisome | cellular\_component | 0.0 | 1.0 | 1.0 | 0 | cluster\_plot | 8 |
| 1860 | GO:0016411 | acylglycerol O-acyltransferase activity | molecular\_function | 0.0 | 1.0 | 1.0 | 0 | cluster\_plot | 12 |
| 1861 | GO:0008987 | quinolinate synthetase A activity | molecular\_function | 0.0 | 1.0 | 1.0 | 0 | cluster\_plot | 2 |
| 1862 | GO:0016813 | hydrolase activity, acting on carbon-nitrogen (but not peptide) bonds, in linear amidines | molecular\_function | 0.0 | 1.0 | 1.0 | 0 | cluster\_plot | 5 |
| 1863 | GO:0043232 | intracellular non-membrane-bounded organelle | cellular\_component | 0.0 | 1.0 | 1.0 | 0 | cluster\_plot | 485 |
| 1864 | GO:0006200 | ATP catabolic process | biological\_process | 0.0 | 1.0 | 1.0 | 0 | cluster\_plot | 19 |
| 1865 | GO:0043527 | tRNA methyltransferase complex | cellular\_component | 0.0 | 1.0 | 1.0 | 0 | cluster\_plot | 3 |
| 1866 | GO:0008641 | small protein activating enzyme activity | molecular\_function | 0.0 | 1.0 | 1.0 | 0 | cluster\_plot | 7 |
| 1867 | GO:0015037 | peptide disulfide oxidoreductase activity | molecular\_function | 0.0 | 1.0 | 1.0 | 0 | cluster\_plot | 2 |
| 1868 | GO:0032954 | regulation of cytokinetic process | biological\_process | 0.0 | 1.0 | 1.0 | 0 | cluster\_plot | 1 |
| 1869 | GO:0001510 | RNA methylation | biological\_process | 0.0 | 1.0 | 1.0 | 0 | cluster\_plot | 8 |
| 1870 | GO:0006544 | glycine metabolic process | biological\_process | 0.0 | 1.0 | 1.0 | 0 | cluster\_plot | 28 |
| 1871 | GO:0042578 | phosphoric ester hydrolase activity | molecular\_function | 0.0 | 1.0 | 1.0 | 0 | cluster\_plot | 168 |
| 1872 | GO:0015002 | heme-copper terminal oxidase activity | molecular\_function | 0.0 | 1.0 | 1.0 | 0 | cluster\_plot | 21 |
| 1873 | GO:0006542 | glutamine biosynthetic process | biological\_process | 0.0 | 1.0 | 1.0 | 0 | cluster\_plot | 6 |
| 1874 | GO:0019673 | GDP-mannose metabolic process | biological\_process | 0.0 | 1.0 | 1.0 | 0 | cluster\_plot | 1 |
| 1875 | GO:0070071 | proton-transporting two-sector ATPase complex assembly | biological\_process | 0.0 | 1.0 | 1.0 | 0 | cluster\_plot | 5 |
| 1876 | GO:0004829 | threonine-tRNA ligase activity | molecular\_function | 0.0 | 1.0 | 1.0 | 0 | cluster\_plot | 3 |
| 1877 | GO:0031399 | regulation of protein modification process | biological\_process | 0.0 | 1.0 | 1.0 | 0 | cluster\_plot | 11 |
| 1878 | GO:0006575 | cellular modified amino acid metabolic process | biological\_process | 0.0 | 1.0 | 1.0 | 0 | cluster\_plot | 58 |
| 1879 | GO:0008442 | 3-hydroxyisobutyrate dehydrogenase activity | molecular\_function | 0.0 | 1.0 | 1.0 | 0 | cluster\_plot | 1 |
| 1880 | GO:0004591 | oxoglutarate dehydrogenase (succinyl-transferring) activity | molecular\_function | 0.0 | 1.0 | 1.0 | 0 | cluster\_plot | 2 |
| 1881 | GO:0033013 | tetrapyrrole metabolic process | biological\_process | 0.0 | 1.0 | 1.0 | 0 | cluster\_plot | 58 |
| 1882 | GO:1902679 | negative regulation of RNA biosynthetic process | biological\_process | 0.0 | 1.0 | 1.0 | 0 | cluster\_plot | 8 |
| 1883 | GO:0015101 | organic cation transmembrane transporter activity | molecular\_function | 0.0 | 1.0 | 1.0 | 0 | cluster\_plot | 18 |
| 1884 | GO:0017069 | snRNA binding | molecular\_function | 0.0 | 1.0 | 1.0 | 0 | cluster\_plot | 2 |
| 1885 | GO:0004866 | endopeptidase inhibitor activity | molecular\_function | 0.0 | 1.0 | 1.0 | 0 | cluster\_plot | 22 |
| 1886 | GO:0004124 | cysteine synthase activity | molecular\_function | 0.0 | 1.0 | 1.0 | 0 | cluster\_plot | 5 |
| 1887 | GO:0034477 | U6 snRNA 3'-end processing | biological\_process | 0.0 | 1.0 | 1.0 | 0 | cluster\_plot | 1 |
| 1888 | GO:0003913 | DNA photolyase activity | molecular\_function | 0.0 | 1.0 | 1.0 | 0 | cluster\_plot | 8 |
| 1889 | GO:0051287 | NAD binding | molecular\_function | 0.0 | 1.0 | 1.0 | 0 | cluster\_plot | 67 |
| 1890 | GO:0005856 | cytoskeleton | cellular\_component | 0.0 | 1.0 | 1.0 | 0 | cluster\_plot | 18 |
| 1891 | GO:0016868 | intramolecular transferase activity, phosphotransferases | molecular\_function | 0.0 | 1.0 | 1.0 | 0 | cluster\_plot | 15 |
| 1892 | GO:0008283 | cell proliferation | biological\_process | 0.0 | 1.0 | 1.0 | 0 | cluster\_plot | 11 |
| 1893 | GO:0019627 | urea metabolic process | biological\_process | 0.0 | 1.0 | 1.0 | 0 | cluster\_plot | 2 |
| 1894 | GO:0004616 | phosphogluconate dehydrogenase (decarboxylating) activity | molecular\_function | 0.0 | 1.0 | 1.0 | 0 | cluster\_plot | 10 |
| 1895 | GO:0016684 | oxidoreductase activity, acting on peroxide as acceptor | molecular\_function | 0.0 | 1.0 | 1.0 | 0 | cluster\_plot | 142 |
| 1896 | GO:0004748 | ribonucleoside-diphosphate reductase activity, thioredoxin disulfide as acceptor | molecular\_function | 0.0 | 1.0 | 1.0 | 0 | cluster\_plot | 1 |
| 1897 | GO:0016857 | racemase and epimerase activity, acting on carbohydrates and derivatives | molecular\_function | 0.0 | 1.0 | 1.0 | 0 | cluster\_plot | 10 |
| 1898 | GO:0030173 | integral component of Golgi membrane | cellular\_component | 0.0 | 1.0 | 1.0 | 0 | cluster\_plot | 1 |
| 1899 | GO:0004476 | mannose-6-phosphate isomerase activity | molecular\_function | 0.0 | 1.0 | 1.0 | 0 | cluster\_plot | 5 |
| 1900 | GO:0006379 | mRNA cleavage | biological\_process | 0.0 | 1.0 | 1.0 | 0 | cluster\_plot | 1 |
| 1901 | GO:0006069 | ethanol oxidation | biological\_process | 0.0 | 1.0 | 1.0 | 0 | cluster\_plot | 1 |
| 1902 | GO:0051130 | positive regulation of cellular component organization | biological\_process | 0.0 | 1.0 | 1.0 | 0 | cluster\_plot | 7 |
| 1903 | GO:0000775 | chromosome, centromeric region | cellular\_component | 0.0 | 1.0 | 1.0 | 0 | cluster\_plot | 10 |
| 1904 | GO:0006826 | iron ion transport | biological\_process | 0.0 | 1.0 | 1.0 | 0 | cluster\_plot | 4 |
| 1905 | GO:0008173 | RNA methyltransferase activity | molecular\_function | 0.0 | 1.0 | 1.0 | 0 | cluster\_plot | 29 |
| 1906 | GO:0006448 | regulation of translational elongation | biological\_process | 0.0 | 1.0 | 1.0 | 0 | cluster\_plot | 7 |
| 1907 | GO:0008654 | phospholipid biosynthetic process | biological\_process | 0.0 | 1.0 | 1.0 | 0 | cluster\_plot | 40 |
| 1908 | GO:2000104 | negative regulation of DNA-dependent DNA replication | biological\_process | 0.0 | 1.0 | 1.0 | 0 | cluster\_plot | 2 |
| 1909 | GO:0000922 | spindle pole | cellular\_component | 0.0 | 1.0 | 1.0 | 0 | cluster\_plot | 12 |
| 1910 | GO:0031227 | intrinsic component of endoplasmic reticulum membrane | cellular\_component | 0.0 | 1.0 | 1.0 | 0 | cluster\_plot | 6 |
| 1911 | GO:0046129 | purine ribonucleoside biosynthetic process | biological\_process | 0.0 | 1.0 | 1.0 | 0 | cluster\_plot | 72 |
| 1912 | GO:0009157 | deoxyribonucleoside monophosphate biosynthetic process | biological\_process | 0.0 | 1.0 | 1.0 | 0 | cluster\_plot | 1 |
| 1913 | GO:0006422 | aspartyl-tRNA aminoacylation | biological\_process | 0.0 | 1.0 | 1.0 | 0 | cluster\_plot | 1 |
| 1914 | GO:0043626 | PCNA complex | cellular\_component | 0.0 | 1.0 | 1.0 | 0 | cluster\_plot | 3 |
| 1915 | GO:0016708 | oxidoreductase activity, acting on paired donors, with incorporation or reduction of molecular oxygen, NAD(P)H as one donor, and incorporation of two atoms of oxygen into one donor | molecular\_function | 0.0 | 1.0 | 1.0 | 0 | cluster\_plot | 1 |
| 1916 | GO:0015696 | ammonium transport | biological\_process | 0.0 | 1.0 | 1.0 | 0 | cluster\_plot | 18 |
| 1917 | GO:1901068 | guanosine-containing compound metabolic process | biological\_process | 0.0 | 1.0 | 1.0 | 0 | cluster\_plot | 38 |
| 1918 | GO:0046653 | tetrahydrofolate metabolic process | biological\_process | 0.0 | 1.0 | 1.0 | 0 | cluster\_plot | 3 |
| 1919 | GO:0010948 | negative regulation of cell cycle process | biological\_process | 0.0 | 1.0 | 1.0 | 0 | cluster\_plot | 2 |
| 1920 | GO:0016811 | hydrolase activity, acting on carbon-nitrogen (but not peptide) bonds, in linear amides | molecular\_function | 0.0 | 1.0 | 1.0 | 0 | cluster\_plot | 17 |
| 1921 | GO:0009247 | glycolipid biosynthetic process | biological\_process | 0.0 | 1.0 | 1.0 | 0 | cluster\_plot | 26 |
| 1922 | GO:0045839 | negative regulation of mitosis | biological\_process | 0.0 | 1.0 | 1.0 | 0 | cluster\_plot | 2 |
| 1923 | GO:0046456 | icosanoid biosynthetic process | biological\_process | 0.0 | 1.0 | 1.0 | 0 | cluster\_plot | 3 |
| 1924 | GO:0010558 | negative regulation of macromolecule biosynthetic process | biological\_process | 0.0 | 1.0 | 1.0 | 0 | cluster\_plot | 20 |
| 1925 | GO:0004112 | cyclic-nucleotide phosphodiesterase activity | molecular\_function | 0.0 | 1.0 | 1.0 | 0 | cluster\_plot | 5 |
| 1926 | GO:0004097 | catechol oxidase activity | molecular\_function | 0.0 | 1.0 | 1.0 | 0 | cluster\_plot | 5 |
| 1927 | GO:0008061 | chitin binding | molecular\_function | 0.0 | 1.0 | 1.0 | 0 | cluster\_plot | 3 |
| 1928 | GO:0006928 | cellular component movement | biological\_process | 0.0 | 1.0 | 1.0 | 0 | cluster\_plot | 97 |
| 1929 | GO:0007067 | mitotic nuclear division | biological\_process | 0.0 | 1.0 | 1.0 | 0 | cluster\_plot | 8 |
| 1930 | GO:0065002 | intracellular protein transmembrane transport | biological\_process | 0.0 | 1.0 | 1.0 | 0 | cluster\_plot | 13 |
| 1931 | GO:0031123 | RNA 3'-end processing | biological\_process | 0.0 | 1.0 | 1.0 | 0 | cluster\_plot | 13 |
| 1932 | GO:0018410 | C-terminal protein amino acid modification | biological\_process | 0.0 | 1.0 | 1.0 | 0 | cluster\_plot | 1 |
| 1933 | GO:0000226 | microtubule cytoskeleton organization | biological\_process | 0.0 | 1.0 | 1.0 | 0 | cluster\_plot | 23 |
| 1934 | GO:0046408 | chlorophyll synthetase activity | molecular\_function | 0.0 | 1.0 | 1.0 | 0 | cluster\_plot | 3 |
| 1935 | GO:0043687 | post-translational protein modification | biological\_process | 0.0 | 1.0 | 1.0 | 0 | cluster\_plot | 1 |
| 1936 | GO:0016776 | phosphotransferase activity, phosphate group as acceptor | molecular\_function | 0.0 | 1.0 | 1.0 | 0 | cluster\_plot | 36 |
| 1937 | GO:0070085 | glycosylation | biological\_process | 0.0 | 1.0 | 1.0 | 0 | cluster\_plot | 77 |
| 1938 | GO:0004532 | exoribonuclease activity | molecular\_function | 0.0 | 1.0 | 1.0 | 0 | cluster\_plot | 1 |
| 1939 | GO:0003917 | DNA topoisomerase type I activity | molecular\_function | 0.0 | 1.0 | 1.0 | 0 | cluster\_plot | 5 |
| 1940 | GO:0004181 | metallocarboxypeptidase activity | molecular\_function | 0.0 | 1.0 | 1.0 | 0 | cluster\_plot | 2 |
| 1941 | GO:0005743 | mitochondrial inner membrane | cellular\_component | 0.0 | 1.0 | 1.0 | 0 | cluster\_plot | 17 |
| 1942 | GO:0006108 | malate metabolic process | biological\_process | 0.0 | 1.0 | 1.0 | 0 | cluster\_plot | 14 |
| 1943 | GO:0004654 | polyribonucleotide nucleotidyltransferase activity | molecular\_function | 0.0 | 1.0 | 1.0 | 0 | cluster\_plot | 3 |
| 1944 | GO:0009095 | aromatic amino acid family biosynthetic process, prephenate pathway | biological\_process | 0.0 | 1.0 | 1.0 | 0 | cluster\_plot | 12 |
| 1945 | GO:0004459 | L-lactate dehydrogenase activity | molecular\_function | 0.0 | 1.0 | 1.0 | 0 | cluster\_plot | 2 |
| 1946 | GO:0004788 | thiamine diphosphokinase activity | molecular\_function | 0.0 | 1.0 | 1.0 | 0 | cluster\_plot | 2 |
| 1947 | GO:0007034 | vacuolar transport | biological\_process | 0.0 | 1.0 | 1.0 | 0 | cluster\_plot | 2 |
| 1948 | GO:0031163 | metallo-sulfur cluster assembly | biological\_process | 0.0 | 1.0 | 1.0 | 0 | cluster\_plot | 20 |
| 1949 | GO:0055080 | cation homeostasis | biological\_process | 0.0 | 1.0 | 1.0 | 0 | cluster\_plot | 11 |
| 1950 | GO:0019720 | Mo-molybdopterin cofactor metabolic process | biological\_process | 0.0 | 1.0 | 1.0 | 0 | cluster\_plot | 10 |
| 1951 | GO:0005783 | endoplasmic reticulum | cellular\_component | 0.0 | 1.0 | 1.0 | 0 | cluster\_plot | 38 |
| 1952 | GO:0016405 | CoA-ligase activity | molecular\_function | 0.0 | 1.0 | 1.0 | 0 | cluster\_plot | 2 |
| 1953 | GO:0006529 | asparagine biosynthetic process | biological\_process | 0.0 | 1.0 | 1.0 | 0 | cluster\_plot | 10 |
| 1954 | GO:0042286 | glutamate-1-semialdehyde 2,1-aminomutase activity | molecular\_function | 0.0 | 1.0 | 1.0 | 0 | cluster\_plot | 1 |
| 1955 | GO:0000098 | sulfur amino acid catabolic process | biological\_process | 0.0 | 1.0 | 1.0 | 0 | cluster\_plot | 1 |
| 1956 | GO:0003950 | NAD+ ADP-ribosyltransferase activity | molecular\_function | 0.0 | 1.0 | 1.0 | 0 | cluster\_plot | 6 |
| 1957 | GO:0051128 | regulation of cellular component organization | biological\_process | 0.0 | 1.0 | 1.0 | 0 | cluster\_plot | 24 |
| 1958 | GO:0051258 | protein polymerization | biological\_process | 0.0 | 1.0 | 1.0 | 0 | cluster\_plot | 16 |
| 1959 | GO:0004133 | glycogen debranching enzyme activity | molecular\_function | 0.0 | 1.0 | 1.0 | 0 | cluster\_plot | 2 |
| 1960 | GO:0046524 | sucrose-phosphate synthase activity | molecular\_function | 0.0 | 1.0 | 1.0 | 0 | cluster\_plot | 7 |
| 1961 | GO:0004719 | protein-L-isoaspartate (D-aspartate) O-methyltransferase activity | molecular\_function | 0.0 | 1.0 | 1.0 | 0 | cluster\_plot | 2 |
| 1962 | GO:0005618 | cell wall | cellular\_component | 0.0 | 1.0 | 1.0 | 0 | cluster\_plot | 137 |
| 1963 | GO:0034614 | cellular response to reactive oxygen species | biological\_process | 0.0 | 1.0 | 1.0 | 0 | cluster\_plot | 1 |
| 1964 | GO:0008079 | translation termination factor activity | molecular\_function | 0.0 | 1.0 | 1.0 | 0 | cluster\_plot | 13 |
| 1965 | GO:1902593 | single-organism nuclear import | biological\_process | 0.0 | 1.0 | 1.0 | 0 | cluster\_plot | 9 |
| 1966 | GO:0015444 | magnesium-importing ATPase activity | molecular\_function | 0.0 | 1.0 | 1.0 | 0 | cluster\_plot | 1 |
| 1967 | GO:0006808 | regulation of nitrogen utilization | biological\_process | 0.0 | 1.0 | 1.0 | 0 | cluster\_plot | 2 |
| 1968 | GO:0060590 | ATPase regulator activity | molecular\_function | 0.0 | 1.0 | 1.0 | 0 | cluster\_plot | 9 |
| 1969 | GO:0006553 | lysine metabolic process | biological\_process | 0.0 | 1.0 | 1.0 | 0 | cluster\_plot | 13 |
| 1970 | GO:0008831 | dTDP-4-dehydrorhamnose reductase activity | molecular\_function | 0.0 | 1.0 | 1.0 | 0 | cluster\_plot | 7 |
| 1971 | GO:0009206 | purine ribonucleoside triphosphate biosynthetic process | biological\_process | 0.0 | 1.0 | 1.0 | 0 | cluster\_plot | 56 |
| 1972 | GO:0070008 | serine-type exopeptidase activity | molecular\_function | 0.0 | 1.0 | 1.0 | 0 | cluster\_plot | 46 |
| 1973 | GO:0006505 | GPI anchor metabolic process | biological\_process | 0.0 | 1.0 | 1.0 | 0 | cluster\_plot | 22 |
| 1974 | GO:0009071 | serine family amino acid catabolic process | biological\_process | 0.0 | 1.0 | 1.0 | 0 | cluster\_plot | 14 |
| 1975 | GO:0046348 | amino sugar catabolic process | biological\_process | 0.0 | 1.0 | 1.0 | 0 | cluster\_plot | 18 |
| 1976 | GO:0012511 | monolayer-surrounded lipid storage body | cellular\_component | 0.0 | 1.0 | 1.0 | 0 | cluster\_plot | 11 |
| 1977 | GO:0046185 | aldehyde catabolic process | biological\_process | 0.0 | 1.0 | 1.0 | 0 | cluster\_plot | 2 |
| 1978 | GO:0008193 | tRNA guanylyltransferase activity | molecular\_function | 0.0 | 1.0 | 1.0 | 0 | cluster\_plot | 1 |
| 1979 | GO:0016530 | metallochaperone activity | molecular\_function | 0.0 | 1.0 | 1.0 | 0 | cluster\_plot | 3 |
| 1980 | GO:0004156 | dihydropteroate synthase activity | molecular\_function | 0.0 | 1.0 | 1.0 | 0 | cluster\_plot | 3 |
| 1981 | GO:1901700 | response to oxygen-containing compound | biological\_process | 0.0 | 1.0 | 1.0 | 0 | cluster\_plot | 8 |
| 1982 | GO:0006568 | tryptophan metabolic process | biological\_process | 0.0 | 1.0 | 1.0 | 0 | cluster\_plot | 16 |
| 1983 | GO:0005548 | phospholipid transporter activity | molecular\_function | 0.0 | 1.0 | 1.0 | 0 | cluster\_plot | 17 |
| 1984 | GO:1901616 | organic hydroxy compound catabolic process | biological\_process | 0.0 | 1.0 | 1.0 | 0 | cluster\_plot | 9 |
| 1985 | GO:0030003 | cellular cation homeostasis | biological\_process | 0.0 | 1.0 | 1.0 | 0 | cluster\_plot | 6 |
| 1986 | GO:0006083 | acetate metabolic process | biological\_process | 0.0 | 1.0 | 1.0 | 0 | cluster\_plot | 2 |
| 1987 | GO:0009162 | deoxyribonucleoside monophosphate metabolic process | biological\_process | 0.0 | 1.0 | 1.0 | 0 | cluster\_plot | 1 |
| 1988 | GO:0006541 | glutamine metabolic process | biological\_process | 0.0 | 1.0 | 1.0 | 0 | cluster\_plot | 15 |
| 1989 | GO:0005789 | endoplasmic reticulum membrane | cellular\_component | 0.0 | 1.0 | 1.0 | 0 | cluster\_plot | 18 |
| 1990 | GO:0008408 | 3'-5' exonuclease activity | molecular\_function | 0.0 | 1.0 | 1.0 | 0 | cluster\_plot | 14 |
| 1991 | GO:0010508 | positive regulation of autophagy | biological\_process | 0.0 | 1.0 | 1.0 | 0 | cluster\_plot | 1 |
| 1992 | GO:0009129 | pyrimidine nucleoside monophosphate metabolic process | biological\_process | 0.0 | 1.0 | 1.0 | 0 | cluster\_plot | 6 |
| 1993 | GO:0004791 | thioredoxin-disulfide reductase activity | molecular\_function | 0.0 | 1.0 | 1.0 | 0 | cluster\_plot | 1 |
| 1994 | GO:0008380 | RNA splicing | biological\_process | 0.0 | 1.0 | 1.0 | 0 | cluster\_plot | 10 |
| 1995 | GO:0006102 | isocitrate metabolic process | biological\_process | 0.0 | 1.0 | 1.0 | 0 | cluster\_plot | 7 |
| 1996 | GO:0019200 | carbohydrate kinase activity | molecular\_function | 0.0 | 1.0 | 1.0 | 0 | cluster\_plot | 19 |
| 1997 | GO:0005351 | sugar:proton symporter activity | molecular\_function | 0.0 | 1.0 | 1.0 | 0 | cluster\_plot | 12 |
| 1998 | GO:0016433 | rRNA (adenine) methyltransferase activity | molecular\_function | 0.0 | 1.0 | 1.0 | 0 | cluster\_plot | 3 |
| 1999 | GO:0004351 | glutamate decarboxylase activity | molecular\_function | 0.0 | 1.0 | 1.0 | 0 | cluster\_plot | 6 |
| 2000 | GO:0023021 | termination of signal transduction | biological\_process | 0.0 | 1.0 | 1.0 | 0 | cluster\_plot | 2 |
| 2001 | GO:0006814 | sodium ion transport | biological\_process | 0.0 | 1.0 | 1.0 | 0 | cluster\_plot | 30 |
| 2002 | GO:0060589 | nucleoside-triphosphatase regulator activity | molecular\_function | 0.0 | 1.0 | 1.0 | 0 | cluster\_plot | 74 |
| 2003 | GO:0008837 | diaminopimelate epimerase activity | molecular\_function | 0.0 | 1.0 | 1.0 | 0 | cluster\_plot | 3 |
| 2004 | GO:0008215 | spermine metabolic process | biological\_process | 0.0 | 1.0 | 1.0 | 0 | cluster\_plot | 5 |
| 2005 | GO:0070568 | guanylyltransferase activity | molecular\_function | 0.0 | 1.0 | 1.0 | 0 | cluster\_plot | 4 |
| 2006 | GO:0004861 | cyclin-dependent protein serine/threonine kinase inhibitor activity | molecular\_function | 0.0 | 1.0 | 1.0 | 0 | cluster\_plot | 8 |
| 2007 | GO:0008448 | N-acetylglucosamine-6-phosphate deacetylase activity | molecular\_function | 0.0 | 1.0 | 1.0 | 0 | cluster\_plot | 1 |
| 2008 | GO:0016835 | carbon-oxygen lyase activity | molecular\_function | 0.0 | 1.0 | 1.0 | 0 | cluster\_plot | 124 |
| 2009 | GO:0004650 | polygalacturonase activity | molecular\_function | 0.0 | 1.0 | 1.0 | 0 | cluster\_plot | 75 |
| 2010 | GO:0004639 | phosphoribosylaminoimidazolesuccinocarboxamide synthase activity | molecular\_function | 0.0 | 1.0 | 1.0 | 0 | cluster\_plot | 1 |
| 2011 | GO:0046500 | S-adenosylmethionine metabolic process | biological\_process | 0.0 | 1.0 | 1.0 | 0 | cluster\_plot | 8 |
| 2012 | GO:0009168 | purine ribonucleoside monophosphate biosynthetic process | biological\_process | 0.0 | 1.0 | 1.0 | 0 | cluster\_plot | 71 |
| 2013 | GO:0071617 | lysophospholipid acyltransferase activity | molecular\_function | 0.0 | 1.0 | 1.0 | 0 | cluster\_plot | 2 |
| 2014 | GO:0023057 | negative regulation of signaling | biological\_process | 0.0 | 1.0 | 1.0 | 0 | cluster\_plot | 2 |
| 2015 | GO:0031570 | DNA integrity checkpoint | biological\_process | 0.0 | 1.0 | 1.0 | 0 | cluster\_plot | 2 |
| 2016 | GO:0004611 | phosphoenolpyruvate carboxykinase activity | molecular\_function | 0.0 | 1.0 | 1.0 | 0 | cluster\_plot | 14 |
| 2017 | GO:0004462 | lactoylglutathione lyase activity | molecular\_function | 0.0 | 1.0 | 1.0 | 0 | cluster\_plot | 9 |
| 2018 | GO:0009976 | tocopherol cyclase activity | molecular\_function | 0.0 | 1.0 | 1.0 | 0 | cluster\_plot | 3 |
| 2019 | GO:0004825 | methionine-tRNA ligase activity | molecular\_function | 0.0 | 1.0 | 1.0 | 0 | cluster\_plot | 3 |
| 2020 | GO:0004020 | adenylylsulfate kinase activity | molecular\_function | 0.0 | 1.0 | 1.0 | 0 | cluster\_plot | 6 |
| 2021 | GO:0016751 | S-succinyltransferase activity | molecular\_function | 0.0 | 1.0 | 1.0 | 0 | cluster\_plot | 3 |
| 2022 | GO:0016769 | transferase activity, transferring nitrogenous groups | molecular\_function | 0.0 | 1.0 | 1.0 | 0 | cluster\_plot | 31 |
| 2023 | GO:0000786 | nucleosome | cellular\_component | 0.0 | 1.0 | 1.0 | 0 | cluster\_plot | 24 |
| 2024 | GO:0003864 | 3-methyl-2-oxobutanoate hydroxymethyltransferase activity | molecular\_function | 0.0 | 1.0 | 1.0 | 0 | cluster\_plot | 4 |
| 2025 | GO:0030060 | L-malate dehydrogenase activity | molecular\_function | 0.0 | 1.0 | 1.0 | 0 | cluster\_plot | 11 |
| 2026 | GO:0019362 | pyridine nucleotide metabolic process | biological\_process | 0.0 | 1.0 | 1.0 | 0 | cluster\_plot | 31 |
| 2027 | GO:0006690 | icosanoid metabolic process | biological\_process | 0.0 | 1.0 | 1.0 | 0 | cluster\_plot | 3 |
| 2028 | GO:0044448 | cell cortex part | cellular\_component | 0.0 | 1.0 | 1.0 | 0 | cluster\_plot | 40 |
| 2029 | GO:0005247 | voltage-gated chloride channel activity | molecular\_function | 0.0 | 1.0 | 1.0 | 0 | cluster\_plot | 13 |
| 2030 | GO:0044106 | cellular amine metabolic process | biological\_process | 0.0 | 1.0 | 1.0 | 0 | cluster\_plot | 27 |
| 2031 | GO:0009394 | 2'-deoxyribonucleotide metabolic process | biological\_process | 0.0 | 1.0 | 1.0 | 0 | cluster\_plot | 4 |
| 2032 | GO:0016676 | oxidoreductase activity, acting on a heme group of donors, oxygen as acceptor | molecular\_function | 0.0 | 1.0 | 1.0 | 0 | cluster\_plot | 21 |
| 2033 | GO:0008479 | queuine tRNA-ribosyltransferase activity | molecular\_function | 0.0 | 1.0 | 1.0 | 0 | cluster\_plot | 3 |
| 2034 | GO:0022900 | electron transport chain | biological\_process | 0.0 | 1.0 | 1.0 | 0 | cluster\_plot | 22 |
| 2035 | GO:0046474 | glycerophospholipid biosynthetic process | biological\_process | 0.0 | 1.0 | 1.0 | 0 | cluster\_plot | 20 |
| 2036 | GO:0048478 | replication fork protection | biological\_process | 0.0 | 1.0 | 1.0 | 0 | cluster\_plot | 2 |
| 2037 | GO:0003825 | alpha,alpha-trehalose-phosphate synthase (UDP-forming) activity | molecular\_function | 0.0 | 1.0 | 1.0 | 0 | cluster\_plot | 2 |
| 2038 | GO:0097346 | INO80-type complex | cellular\_component | 0.0 | 1.0 | 1.0 | 0 | cluster\_plot | 4 |
| 2039 | GO:0034030 | ribonucleoside bisphosphate biosynthetic process | biological\_process | 0.0 | 1.0 | 1.0 | 0 | cluster\_plot | 2 |
| 2040 | GO:0022603 | regulation of anatomical structure morphogenesis | biological\_process | 0.0 | 1.0 | 1.0 | 0 | cluster\_plot | 5 |
| 2041 | GO:0098588 | bounding membrane of organelle | cellular\_component | 0.0 | 1.0 | 1.0 | 0 | cluster\_plot | 50 |
| 2042 | GO:0010340 | carboxyl-O-methyltransferase activity | molecular\_function | 0.0 | 1.0 | 1.0 | 0 | cluster\_plot | 3 |
| 2043 | GO:0051247 | positive regulation of protein metabolic process | biological\_process | 0.0 | 1.0 | 1.0 | 0 | cluster\_plot | 3 |
| 2044 | GO:0004514 | nicotinate-nucleotide diphosphorylase (carboxylating) activity | molecular\_function | 0.0 | 1.0 | 1.0 | 0 | cluster\_plot | 1 |
| 2045 | GO:0008047 | enzyme activator activity | molecular\_function | 0.0 | 1.0 | 1.0 | 0 | cluster\_plot | 57 |
| 2046 | GO:0051707 | response to other organism | biological\_process | 0.0 | 1.0 | 1.0 | 0 | cluster\_plot | 1 |
| 2047 | GO:0046903 | secretion | biological\_process | 0.0 | 1.0 | 1.0 | 0 | cluster\_plot | 41 |
| 2048 | GO:0038032 | termination of G-protein coupled receptor signaling pathway | biological\_process | 0.0 | 1.0 | 1.0 | 0 | cluster\_plot | 2 |
| 2049 | GO:0009966 | regulation of signal transduction | biological\_process | 0.0 | 1.0 | 1.0 | 0 | cluster\_plot | 18 |
| 2050 | GO:0000375 | RNA splicing, via transesterification reactions | biological\_process | 0.0 | 1.0 | 1.0 | 0 | cluster\_plot | 6 |
| 2051 | GO:0004350 | glutamate-5-semialdehyde dehydrogenase activity | molecular\_function | 0.0 | 1.0 | 1.0 | 0 | cluster\_plot | 2 |
| 2052 | GO:0051202 | phytochromobilin metabolic process | biological\_process | 0.0 | 1.0 | 1.0 | 0 | cluster\_plot | 2 |
| 2053 | GO:0008655 | pyrimidine-containing compound salvage | biological\_process | 0.0 | 1.0 | 1.0 | 0 | cluster\_plot | 1 |
| 2054 | GO:0031967 | organelle envelope | cellular\_component | 0.0 | 1.0 | 1.0 | 0 | cluster\_plot | 7 |
| 2055 | GO:0044438 | microbody part | cellular\_component | 0.0 | 1.0 | 1.0 | 0 | cluster\_plot | 11 |
| 2056 | GO:0016229 | steroid dehydrogenase activity | molecular\_function | 0.0 | 1.0 | 1.0 | 0 | cluster\_plot | 15 |
| 2057 | GO:0032501 | multicellular organismal process | biological\_process | 0.0 | 1.0 | 1.0 | 0 | cluster\_plot | 16 |
| 2058 | GO:0018342 | protein prenylation | biological\_process | 0.0 | 1.0 | 1.0 | 0 | cluster\_plot | 4 |
| 2059 | GO:0004555 | alpha,alpha-trehalase activity | molecular\_function | 0.0 | 1.0 | 1.0 | 0 | cluster\_plot | 2 |
| 2060 | GO:0009189 | deoxyribonucleoside diphosphate biosynthetic process | biological\_process | 0.0 | 1.0 | 1.0 | 0 | cluster\_plot | 2 |
| 2061 | GO:0006506 | GPI anchor biosynthetic process | biological\_process | 0.0 | 1.0 | 1.0 | 0 | cluster\_plot | 18 |
| 2062 | GO:0009148 | pyrimidine nucleoside triphosphate biosynthetic process | biological\_process | 0.0 | 1.0 | 1.0 | 0 | cluster\_plot | 7 |
| 2063 | GO:0004066 | asparagine synthase (glutamine-hydrolyzing) activity | molecular\_function | 0.0 | 1.0 | 1.0 | 0 | cluster\_plot | 10 |
| 2064 | GO:0007088 | regulation of mitosis | biological\_process | 0.0 | 1.0 | 1.0 | 0 | cluster\_plot | 5 |
| 2065 | GO:0004372 | glycine hydroxymethyltransferase activity | molecular\_function | 0.0 | 1.0 | 1.0 | 0 | cluster\_plot | 13 |
| 2066 | GO:0033897 | ribonuclease T2 activity | molecular\_function | 0.0 | 1.0 | 1.0 | 0 | cluster\_plot | 12 |
| 2067 | GO:0004799 | thymidylate synthase activity | molecular\_function | 0.0 | 1.0 | 1.0 | 0 | cluster\_plot | 1 |
| 2068 | GO:0016073 | snRNA metabolic process | biological\_process | 0.0 | 1.0 | 1.0 | 0 | cluster\_plot | 1 |
| 2069 | GO:0042727 | flavin-containing compound biosynthetic process | biological\_process | 0.0 | 1.0 | 1.0 | 0 | cluster\_plot | 13 |
| 2070 | GO:0015157 | oligosaccharide transmembrane transporter activity | molecular\_function | 0.0 | 1.0 | 1.0 | 0 | cluster\_plot | 6 |
| 2071 | GO:0008963 | phospho-N-acetylmuramoyl-pentapeptide-transferase activity | molecular\_function | 0.0 | 1.0 | 1.0 | 0 | cluster\_plot | 3 |
| 2072 | GO:0006886 | intracellular protein transport | biological\_process | 0.0 | 1.0 | 1.0 | 0 | cluster\_plot | 198 |
| 2073 | GO:0019867 | outer membrane | cellular\_component | 0.0 | 1.0 | 1.0 | 0 | cluster\_plot | 21 |
| 2074 | GO:0000280 | nuclear division | biological\_process | 0.0 | 1.0 | 1.0 | 0 | cluster\_plot | 8 |
| 2075 | GO:0051224 | negative regulation of protein transport | biological\_process | 0.0 | 1.0 | 1.0 | 0 | cluster\_plot | 2 |
| 2076 | GO:0008703 | 5-amino-6-(5-phosphoribosylamino)uracil reductase activity | molecular\_function | 0.0 | 1.0 | 1.0 | 0 | cluster\_plot | 1 |
| 2077 | GO:0009208 | pyrimidine ribonucleoside triphosphate metabolic process | biological\_process | 0.0 | 1.0 | 1.0 | 0 | cluster\_plot | 7 |
| 2078 | GO:0004798 | thymidylate kinase activity | molecular\_function | 0.0 | 1.0 | 1.0 | 0 | cluster\_plot | 2 |
| 2079 | GO:0004801 | sedoheptulose-7-phosphate:D-glyceraldehyde-3-phosphate glyceronetransferase activity | molecular\_function | 0.0 | 1.0 | 1.0 | 0 | cluster\_plot | 2 |
| 2080 | GO:0008428 | ribonuclease inhibitor activity | molecular\_function | 0.0 | 1.0 | 1.0 | 0 | cluster\_plot | 2 |
| 2081 | GO:0005085 | guanyl-nucleotide exchange factor activity | molecular\_function | 0.0 | 1.0 | 1.0 | 0 | cluster\_plot | 25 |
| 2082 | GO:0051539 | 4 iron, 4 sulfur cluster binding | molecular\_function | 0.0 | 1.0 | 1.0 | 0 | cluster\_plot | 22 |
| 2083 | GO:0032270 | positive regulation of cellular protein metabolic process | biological\_process | 0.0 | 1.0 | 1.0 | 0 | cluster\_plot | 3 |
| 2084 | GO:0031668 | cellular response to extracellular stimulus | biological\_process | 0.0 | 1.0 | 1.0 | 0 | cluster\_plot | 7 |
| 2085 | GO:0004310 | farnesyl-diphosphate farnesyltransferase activity | molecular\_function | 0.0 | 1.0 | 1.0 | 0 | cluster\_plot | 2 |
| 2086 | GO:0044599 | AP-5 adaptor complex | cellular\_component | 0.0 | 1.0 | 1.0 | 0 | cluster\_plot | 2 |
| 2087 | GO:0061025 | membrane fusion | biological\_process | 0.0 | 1.0 | 1.0 | 0 | cluster\_plot | 2 |
| 2088 | GO:0035303 | regulation of dephosphorylation | biological\_process | 0.0 | 1.0 | 1.0 | 0 | cluster\_plot | 1 |
| 2089 | GO:0030414 | peptidase inhibitor activity | molecular\_function | 0.0 | 1.0 | 1.0 | 0 | cluster\_plot | 22 |
| 2090 | GO:0030120 | vesicle coat | cellular\_component | 0.0 | 1.0 | 1.0 | 0 | cluster\_plot | 42 |
| 2091 | GO:0004197 | cysteine-type endopeptidase activity | molecular\_function | 0.0 | 1.0 | 1.0 | 0 | cluster\_plot | 23 |
| 2092 | GO:0030833 | regulation of actin filament polymerization | biological\_process | 0.0 | 1.0 | 1.0 | 0 | cluster\_plot | 10 |
| 2093 | GO:0008234 | cysteine-type peptidase activity | molecular\_function | 0.0 | 1.0 | 1.0 | 0 | cluster\_plot | 94 |
| 2094 | GO:0016538 | cyclin-dependent protein serine/threonine kinase regulator activity | molecular\_function | 0.0 | 1.0 | 1.0 | 0 | cluster\_plot | 10 |
| 2095 | GO:0045277 | respiratory chain complex IV | cellular\_component | 0.0 | 1.0 | 1.0 | 0 | cluster\_plot | 5 |
| 2096 | GO:0016211 | ammonia ligase activity | molecular\_function | 0.0 | 1.0 | 1.0 | 0 | cluster\_plot | 6 |
| 2097 | GO:0030328 | prenylcysteine catabolic process | biological\_process | 0.0 | 1.0 | 1.0 | 0 | cluster\_plot | 1 |
| 2098 | GO:0048285 | organelle fission | biological\_process | 0.0 | 1.0 | 1.0 | 0 | cluster\_plot | 17 |
| 2099 | GO:0000398 | mRNA splicing, via spliceosome | biological\_process | 0.0 | 1.0 | 1.0 | 0 | cluster\_plot | 6 |
| 2100 | GO:0004017 | adenylate kinase activity | molecular\_function | 0.0 | 1.0 | 1.0 | 0 | cluster\_plot | 3 |
| 2101 | GO:0016668 | oxidoreductase activity, acting on a sulfur group of donors, NAD(P) as acceptor | molecular\_function | 0.0 | 1.0 | 1.0 | 0 | cluster\_plot | 12 |
| 2102 | GO:0034508 | centromere complex assembly | biological\_process | 0.0 | 1.0 | 1.0 | 0 | cluster\_plot | 3 |
| 2103 | GO:0009605 | response to external stimulus | biological\_process | 0.0 | 1.0 | 1.0 | 0 | cluster\_plot | 16 |
| 2104 | GO:0046854 | phosphatidylinositol phosphorylation | biological\_process | 0.0 | 1.0 | 1.0 | 0 | cluster\_plot | 13 |
| 2105 | GO:0006835 | dicarboxylic acid transport | biological\_process | 0.0 | 1.0 | 1.0 | 0 | cluster\_plot | 30 |
| 2106 | GO:0004529 | exodeoxyribonuclease activity | molecular\_function | 0.0 | 1.0 | 1.0 | 0 | cluster\_plot | 2 |
| 2107 | GO:0016841 | ammonia-lyase activity | molecular\_function | 0.0 | 1.0 | 1.0 | 0 | cluster\_plot | 6 |
| 2108 | GO:0016255 | attachment of GPI anchor to protein | biological\_process | 0.0 | 1.0 | 1.0 | 0 | cluster\_plot | 6 |
| 2109 | GO:0009013 | succinate-semialdehyde dehydrogenase [NAD(P)+] activity | molecular\_function | 0.0 | 1.0 | 1.0 | 0 | cluster\_plot | 2 |
| 2110 | GO:0031300 | intrinsic component of organelle membrane | cellular\_component | 0.0 | 1.0 | 1.0 | 0 | cluster\_plot | 6 |
| 2111 | GO:0006067 | ethanol metabolic process | biological\_process | 0.0 | 1.0 | 1.0 | 0 | cluster\_plot | 1 |
| 2112 | GO:0009991 | response to extracellular stimulus | biological\_process | 0.0 | 1.0 | 1.0 | 0 | cluster\_plot | 7 |
| 2113 | GO:0046146 | tetrahydrobiopterin metabolic process | biological\_process | 0.0 | 1.0 | 1.0 | 0 | cluster\_plot | 3 |
| 2114 | GO:0000377 | RNA splicing, via transesterification reactions with bulged adenosine as nucleophile | biological\_process | 0.0 | 1.0 | 1.0 | 0 | cluster\_plot | 6 |
| 2115 | GO:0052803 | imidazole-containing compound metabolic process | biological\_process | 0.0 | 1.0 | 1.0 | 0 | cluster\_plot | 17 |
| 2116 | GO:0009894 | regulation of catabolic process | biological\_process | 0.0 | 1.0 | 1.0 | 0 | cluster\_plot | 54 |
| 2117 | GO:1901658 | glycosyl compound catabolic process | biological\_process | 0.0 | 1.0 | 1.0 | 0 | cluster\_plot | 41 |
| 2118 | GO:0008824 | cyanate hydratase activity | molecular\_function | 0.0 | 1.0 | 1.0 | 0 | cluster\_plot | 1 |
| 2119 | GO:0022836 | gated channel activity | molecular\_function | 0.0 | 1.0 | 1.0 | 0 | cluster\_plot | 50 |
| 2120 | GO:0050242 | pyruvate, phosphate dikinase activity | molecular\_function | 0.0 | 1.0 | 1.0 | 0 | cluster\_plot | 6 |
| 2121 | GO:0006879 | cellular iron ion homeostasis | biological\_process | 0.0 | 1.0 | 1.0 | 0 | cluster\_plot | 6 |
| 2122 | GO:0004659 | prenyltransferase activity | molecular\_function | 0.0 | 1.0 | 1.0 | 0 | cluster\_plot | 19 |
| 2123 | GO:0009169 | purine ribonucleoside monophosphate catabolic process | biological\_process | 0.0 | 1.0 | 1.0 | 0 | cluster\_plot | 19 |
| 2124 | GO:0044107 | cellular alcohol metabolic process | biological\_process | 0.0 | 1.0 | 1.0 | 0 | cluster\_plot | 3 |
| 2125 | GO:0030259 | lipid glycosylation | biological\_process | 0.0 | 1.0 | 1.0 | 0 | cluster\_plot | 16 |
| 2126 | GO:0006168 | adenine salvage | biological\_process | 0.0 | 1.0 | 1.0 | 0 | cluster\_plot | 8 |
| 2127 | GO:0007031 | peroxisome organization | biological\_process | 0.0 | 1.0 | 1.0 | 0 | cluster\_plot | 3 |
| 2128 | GO:0050897 | cobalt ion binding | molecular\_function | 0.0 | 1.0 | 1.0 | 0 | cluster\_plot | 4 |
| 2129 | GO:0006000 | fructose metabolic process | biological\_process | 0.0 | 1.0 | 1.0 | 0 | cluster\_plot | 3 |
| 2130 | GO:0008483 | transaminase activity | molecular\_function | 0.0 | 1.0 | 1.0 | 0 | cluster\_plot | 31 |
| 2131 | GO:0030688 | preribosome, small subunit precursor | cellular\_component | 0.0 | 1.0 | 1.0 | 0 | cluster\_plot | 3 |
| 2132 | GO:0004416 | hydroxyacylglutathione hydrolase activity | molecular\_function | 0.0 | 1.0 | 1.0 | 0 | cluster\_plot | 2 |
| 2133 | GO:0016724 | oxidoreductase activity, oxidizing metal ions, oxygen as acceptor | molecular\_function | 0.0 | 1.0 | 1.0 | 0 | cluster\_plot | 1 |
| 2134 | GO:0004177 | aminopeptidase activity | molecular\_function | 0.0 | 1.0 | 1.0 | 0 | cluster\_plot | 16 |
| 2135 | GO:0016635 | oxidoreductase activity, acting on the CH-CH group of donors, quinone or related compound as acceptor | molecular\_function | 0.0 | 1.0 | 1.0 | 0 | cluster\_plot | 4 |
| 2136 | GO:0015770 | sucrose transport | biological\_process | 0.0 | 1.0 | 1.0 | 0 | cluster\_plot | 6 |
| 2137 | GO:0046930 | pore complex | cellular\_component | 0.0 | 1.0 | 1.0 | 0 | cluster\_plot | 19 |
| 2138 | GO:0003885 | D-arabinono-1,4-lactone oxidase activity | molecular\_function | 0.0 | 1.0 | 1.0 | 0 | cluster\_plot | 5 |
| 2139 | GO:0005992 | trehalose biosynthetic process | biological\_process | 0.0 | 1.0 | 1.0 | 0 | cluster\_plot | 20 |
| 2140 | GO:0006306 | DNA methylation | biological\_process | 0.0 | 1.0 | 1.0 | 0 | cluster\_plot | 15 |
| 2141 | GO:1901072 | glucosamine-containing compound catabolic process | biological\_process | 0.0 | 1.0 | 1.0 | 0 | cluster\_plot | 18 |
| 2142 | GO:0030896 | checkpoint clamp complex | cellular\_component | 0.0 | 1.0 | 1.0 | 0 | cluster\_plot | 2 |
| 2143 | GO:0046490 | isopentenyl diphosphate metabolic process | biological\_process | 0.0 | 1.0 | 1.0 | 0 | cluster\_plot | 2 |
| 2144 | GO:0006636 | unsaturated fatty acid biosynthetic process | biological\_process | 0.0 | 1.0 | 1.0 | 0 | cluster\_plot | 3 |
| 2145 | GO:0065003 | macromolecular complex assembly | biological\_process | 0.0 | 1.0 | 1.0 | 0 | cluster\_plot | 82 |
| 2146 | GO:0016742 | hydroxymethyl-, formyl- and related transferase activity | molecular\_function | 0.0 | 1.0 | 1.0 | 0 | cluster\_plot | 32 |
| 2147 | GO:0016728 | oxidoreductase activity, acting on CH or CH2 groups, disulfide as acceptor | molecular\_function | 0.0 | 1.0 | 1.0 | 0 | cluster\_plot | 1 |
| 2148 | GO:0009266 | response to temperature stimulus | biological\_process | 0.0 | 1.0 | 1.0 | 0 | cluster\_plot | 5 |
| 2149 | GO:0009678 | hydrogen-translocating pyrophosphatase activity | molecular\_function | 0.0 | 1.0 | 1.0 | 0 | cluster\_plot | 9 |
| 2150 | GO:0016854 | racemase and epimerase activity | molecular\_function | 0.0 | 1.0 | 1.0 | 0 | cluster\_plot | 15 |
| 2151 | GO:0006421 | asparaginyl-tRNA aminoacylation | biological\_process | 0.0 | 1.0 | 1.0 | 0 | cluster\_plot | 6 |
| 2152 | GO:0004449 | isocitrate dehydrogenase (NAD+) activity | molecular\_function | 0.0 | 1.0 | 1.0 | 0 | cluster\_plot | 3 |
| 2153 | GO:0042393 | histone binding | molecular\_function | 0.0 | 1.0 | 1.0 | 0 | cluster\_plot | 28 |
| 2154 | GO:0022838 | substrate-specific channel activity | molecular\_function | 0.0 | 1.0 | 1.0 | 0 | cluster\_plot | 76 |
| 2155 | GO:0018196 | peptidyl-asparagine modification | biological\_process | 0.0 | 1.0 | 1.0 | 0 | cluster\_plot | 6 |
| 2156 | GO:0032940 | secretion by cell | biological\_process | 0.0 | 1.0 | 1.0 | 0 | cluster\_plot | 41 |
| 2157 | GO:0006643 | membrane lipid metabolic process | biological\_process | 0.0 | 1.0 | 1.0 | 0 | cluster\_plot | 38 |
| 2158 | GO:0055065 | metal ion homeostasis | biological\_process | 0.0 | 1.0 | 1.0 | 0 | cluster\_plot | 6 |
| 2159 | GO:0005083 | small GTPase regulator activity | molecular\_function | 0.0 | 1.0 | 1.0 | 0 | cluster\_plot | 65 |
| 2160 | GO:0004163 | diphosphomevalonate decarboxylase activity | molecular\_function | 0.0 | 1.0 | 1.0 | 0 | cluster\_plot | 1 |
| 2161 | GO:0009707 | chloroplast outer membrane | cellular\_component | 0.0 | 1.0 | 1.0 | 0 | cluster\_plot | 2 |
| 2162 | GO:0019427 | acetyl-CoA biosynthetic process from acetate | biological\_process | 0.0 | 1.0 | 1.0 | 0 | cluster\_plot | 2 |
| 2163 | GO:0010277 | chlorophyllide a oxygenase [overall] activity | molecular\_function | 0.0 | 1.0 | 1.0 | 0 | cluster\_plot | 7 |
| 2164 | GO:0004367 | glycerol-3-phosphate dehydrogenase [NAD+] activity | molecular\_function | 0.0 | 1.0 | 1.0 | 0 | cluster\_plot | 9 |
| 2165 | GO:0009416 | response to light stimulus | biological\_process | 0.0 | 1.0 | 1.0 | 0 | cluster\_plot | 8 |
| 2166 | GO:0019370 | leukotriene biosynthetic process | biological\_process | 0.0 | 1.0 | 1.0 | 0 | cluster\_plot | 3 |
| 2167 | GO:0072523 | purine-containing compound catabolic process | biological\_process | 0.0 | 1.0 | 1.0 | 0 | cluster\_plot | 41 |
| 2168 | GO:0005788 | endoplasmic reticulum lumen | cellular\_component | 0.0 | 1.0 | 1.0 | 0 | cluster\_plot | 1 |
| 2169 | GO:0043461 | proton-transporting ATP synthase complex assembly | biological\_process | 0.0 | 1.0 | 1.0 | 0 | cluster\_plot | 5 |
| 2170 | GO:0009150 | purine ribonucleotide metabolic process | biological\_process | 0.0 | 1.0 | 1.0 | 0 | cluster\_plot | 117 |
| 2171 | GO:0052646 | alditol phosphate metabolic process | biological\_process | 0.0 | 1.0 | 1.0 | 0 | cluster\_plot | 14 |
| 2172 | GO:0004575 | sucrose alpha-glucosidase activity | molecular\_function | 0.0 | 1.0 | 1.0 | 0 | cluster\_plot | 8 |
| 2173 | GO:0071554 | cell wall organization or biogenesis | biological\_process | 0.0 | 1.0 | 1.0 | 0 | cluster\_plot | 92 |
| 2174 | GO:0071266 | 'de novo' L-methionine biosynthetic process | biological\_process | 0.0 | 1.0 | 1.0 | 0 | cluster\_plot | 1 |
| 2175 | GO:0016415 | octanoyltransferase activity | molecular\_function | 0.0 | 1.0 | 1.0 | 0 | cluster\_plot | 3 |
| 2176 | GO:0004550 | nucleoside diphosphate kinase activity | molecular\_function | 0.0 | 1.0 | 1.0 | 0 | cluster\_plot | 7 |
| 2177 | GO:0006433 | prolyl-tRNA aminoacylation | biological\_process | 0.0 | 1.0 | 1.0 | 0 | cluster\_plot | 4 |
| 2178 | GO:0019212 | phosphatase inhibitor activity | molecular\_function | 0.0 | 1.0 | 1.0 | 0 | cluster\_plot | 1 |
| 2179 | GO:0016763 | transferase activity, transferring pentosyl groups | molecular\_function | 0.0 | 1.0 | 1.0 | 0 | cluster\_plot | 34 |
| 2180 | GO:0000256 | allantoin catabolic process | biological\_process | 0.0 | 1.0 | 1.0 | 0 | cluster\_plot | 2 |
| 2181 | GO:0006020 | inositol metabolic process | biological\_process | 0.0 | 1.0 | 1.0 | 0 | cluster\_plot | 15 |
| 2182 | GO:0044446 | intracellular organelle part | cellular\_component | 0.0 | 1.0 | 1.0 | 0 | cluster\_plot | 532 |
| 2183 | GO:0016701 | oxidoreductase activity, acting on single donors with incorporation of molecular oxygen | molecular\_function | 0.0 | 1.0 | 1.0 | 0 | cluster\_plot | 61 |
| 2184 | GO:0006729 | tetrahydrobiopterin biosynthetic process | biological\_process | 0.0 | 1.0 | 1.0 | 0 | cluster\_plot | 3 |
| 2185 | GO:0016894 | endonuclease activity, active with either ribo- or deoxyribonucleic acids and producing 3'-phosphomonoesters | molecular\_function | 0.0 | 1.0 | 1.0 | 0 | cluster\_plot | 13 |
| 2186 | GO:0031974 | membrane-enclosed lumen | cellular\_component | 0.0 | 1.0 | 1.0 | 0 | cluster\_plot | 9 |
| 2187 | GO:0006313 | transposition, DNA-mediated | biological\_process | 0.0 | 1.0 | 1.0 | 0 | cluster\_plot | 1 |
| 2188 | GO:0019320 | hexose catabolic process | biological\_process | 0.0 | 1.0 | 1.0 | 0 | cluster\_plot | 17 |
| 2189 | GO:0015928 | fucosidase activity | molecular\_function | 0.0 | 1.0 | 1.0 | 0 | cluster\_plot | 4 |
| 2190 | GO:0034061 | DNA polymerase activity | molecular\_function | 0.0 | 1.0 | 1.0 | 0 | cluster\_plot | 43 |
| 2191 | GO:0033177 | proton-transporting two-sector ATPase complex, proton-transporting domain | cellular\_component | 0.0 | 1.0 | 1.0 | 0 | cluster\_plot | 30 |
| 2192 | GO:0044092 | negative regulation of molecular function | biological\_process | 0.0 | 1.0 | 1.0 | 0 | cluster\_plot | 71 |
| 2193 | GO:1901269 | lipooligosaccharide metabolic process | biological\_process | 0.0 | 1.0 | 1.0 | 0 | cluster\_plot | 3 |
| 2194 | GO:0004592 | pantoate-beta-alanine ligase activity | molecular\_function | 0.0 | 1.0 | 1.0 | 0 | cluster\_plot | 2 |
| 2195 | GO:0008484 | sulfuric ester hydrolase activity | molecular\_function | 0.0 | 1.0 | 1.0 | 0 | cluster\_plot | 3 |
| 2196 | GO:0016160 | amylase activity | molecular\_function | 0.0 | 1.0 | 1.0 | 0 | cluster\_plot | 41 |
| 2197 | GO:0009107 | lipoate biosynthetic process | biological\_process | 0.0 | 1.0 | 1.0 | 0 | cluster\_plot | 7 |
| 2198 | GO:0009507 | chloroplast | cellular\_component | 0.0 | 1.0 | 1.0 | 0 | cluster\_plot | 21 |
| 2199 | GO:0005815 | microtubule organizing center | cellular\_component | 0.0 | 1.0 | 1.0 | 0 | cluster\_plot | 12 |
| 2200 | GO:0042651 | thylakoid membrane | cellular\_component | 0.0 | 1.0 | 1.0 | 0 | cluster\_plot | 8 |
| 2201 | GO:0030570 | pectate lyase activity | molecular\_function | 0.0 | 1.0 | 1.0 | 0 | cluster\_plot | 3 |
| 2202 | GO:0044087 | regulation of cellular component biogenesis | biological\_process | 0.0 | 1.0 | 1.0 | 0 | cluster\_plot | 11 |
| 2203 | GO:0045744 | negative regulation of G-protein coupled receptor protein signaling pathway | biological\_process | 0.0 | 1.0 | 1.0 | 0 | cluster\_plot | 2 |
| 2204 | GO:0004362 | glutathione-disulfide reductase activity | molecular\_function | 0.0 | 1.0 | 1.0 | 0 | cluster\_plot | 2 |
| 2205 | GO:0043101 | purine-containing compound salvage | biological\_process | 0.0 | 1.0 | 1.0 | 0 | cluster\_plot | 10 |
| 2206 | GO:0010506 | regulation of autophagy | biological\_process | 0.0 | 1.0 | 1.0 | 0 | cluster\_plot | 1 |
| 2207 | GO:0008760 | UDP-N-acetylglucosamine 1-carboxyvinyltransferase activity | molecular\_function | 0.0 | 1.0 | 1.0 | 0 | cluster\_plot | 2 |
| 2208 | GO:0009261 | ribonucleotide catabolic process | biological\_process | 0.0 | 1.0 | 1.0 | 0 | cluster\_plot | 41 |
| 2209 | GO:0015937 | coenzyme A biosynthetic process | biological\_process | 0.0 | 1.0 | 1.0 | 0 | cluster\_plot | 2 |
| 2210 | GO:0043254 | regulation of protein complex assembly | biological\_process | 0.0 | 1.0 | 1.0 | 0 | cluster\_plot | 10 |
| 2211 | GO:0005759 | mitochondrial matrix | cellular\_component | 0.0 | 1.0 | 1.0 | 0 | cluster\_plot | 6 |
| 2212 | GO:0004152 | dihydroorotate dehydrogenase activity | molecular\_function | 0.0 | 1.0 | 1.0 | 0 | cluster\_plot | 4 |
| 2213 | GO:0004860 | protein kinase inhibitor activity | molecular\_function | 0.0 | 1.0 | 1.0 | 0 | cluster\_plot | 8 |
| 2214 | GO:0003899 | DNA-directed RNA polymerase activity | molecular\_function | 0.0 | 1.0 | 1.0 | 0 | cluster\_plot | 85 |
| 2215 | GO:0051253 | negative regulation of RNA metabolic process | biological\_process | 0.0 | 1.0 | 1.0 | 0 | cluster\_plot | 8 |
| 2216 | GO:0098687 | chromosomal region | cellular\_component | 0.0 | 1.0 | 1.0 | 0 | cluster\_plot | 1 |
| 2217 | GO:0015924 | mannosyl-oligosaccharide mannosidase activity | molecular\_function | 0.0 | 1.0 | 1.0 | 0 | cluster\_plot | 4 |
| 2218 | GO:0008443 | phosphofructokinase activity | molecular\_function | 0.0 | 1.0 | 1.0 | 0 | cluster\_plot | 18 |
| 2219 | GO:0015772 | oligosaccharide transport | biological\_process | 0.0 | 1.0 | 1.0 | 0 | cluster\_plot | 6 |
| 2220 | GO:0042558 | pteridine-containing compound metabolic process | biological\_process | 0.0 | 1.0 | 1.0 | 0 | cluster\_plot | 27 |
| 2221 | GO:0044435 | plastid part | cellular\_component | 0.0 | 1.0 | 1.0 | 0 | cluster\_plot | 2 |
| 2222 | GO:0016717 | oxidoreductase activity, acting on paired donors, with oxidation of a pair of donors resulting in the reduction of molecular oxygen to two molecules of water | molecular\_function | 0.0 | 1.0 | 1.0 | 0 | cluster\_plot | 16 |
| 2223 | GO:0043094 | cellular metabolic compound salvage | biological\_process | 0.0 | 1.0 | 1.0 | 0 | cluster\_plot | 16 |
| 2224 | GO:0006591 | ornithine metabolic process | biological\_process | 0.0 | 1.0 | 1.0 | 0 | cluster\_plot | 3 |
| 2225 | GO:0006378 | mRNA polyadenylation | biological\_process | 0.0 | 1.0 | 1.0 | 0 | cluster\_plot | 3 |
| 2226 | GO:0034599 | cellular response to oxidative stress | biological\_process | 0.0 | 1.0 | 1.0 | 0 | cluster\_plot | 1 |
| 2227 | GO:0042626 | ATPase activity, coupled to transmembrane movement of substances | molecular\_function | 0.0 | 1.0 | 1.0 | 0 | cluster\_plot | 178 |
| 2228 | GO:0051003 | ligase activity, forming nitrogen-metal bonds, forming coordination complexes | molecular\_function | 0.0 | 1.0 | 1.0 | 0 | cluster\_plot | 6 |
| 2229 | GO:0016571 | histone methylation | biological\_process | 0.0 | 1.0 | 1.0 | 0 | cluster\_plot | 16 |
| 2230 | GO:0005089 | Rho guanyl-nucleotide exchange factor activity | molecular\_function | 0.0 | 1.0 | 1.0 | 0 | cluster\_plot | 14 |
| 2231 | GO:0050661 | NADP binding | molecular\_function | 0.0 | 1.0 | 1.0 | 0 | cluster\_plot | 68 |
| 2232 | GO:0006906 | vesicle fusion | biological\_process | 0.0 | 1.0 | 1.0 | 0 | cluster\_plot | 2 |
| 2233 | GO:0005234 | extracellular-glutamate-gated ion channel activity | molecular\_function | 0.0 | 1.0 | 1.0 | 0 | cluster\_plot | 37 |
| 2234 | GO:0019031 | viral envelope | cellular\_component | 0.0 | 1.0 | 1.0 | 0 | cluster\_plot | 1 |
| 2235 | GO:0044707 | single-multicellular organism process | biological\_process | 0.0 | 1.0 | 1.0 | 0 | cluster\_plot | 16 |
| 2236 | GO:0004422 | hypoxanthine phosphoribosyltransferase activity | molecular\_function | 0.0 | 1.0 | 1.0 | 0 | cluster\_plot | 2 |
| 2237 | GO:0015992 | proton transport | biological\_process | 0.0 | 1.0 | 1.0 | 0 | cluster\_plot | 83 |
| 2238 | GO:0009163 | nucleoside biosynthetic process | biological\_process | 0.0 | 1.0 | 1.0 | 0 | cluster\_plot | 80 |
| 2239 | GO:0004014 | adenosylmethionine decarboxylase activity | molecular\_function | 0.0 | 1.0 | 1.0 | 0 | cluster\_plot | 5 |
| 2240 | GO:0015969 | guanosine tetraphosphate metabolic process | biological\_process | 0.0 | 1.0 | 1.0 | 0 | cluster\_plot | 5 |
| 2241 | GO:0004180 | carboxypeptidase activity | molecular\_function | 0.0 | 1.0 | 1.0 | 0 | cluster\_plot | 48 |
| 2242 | GO:0043233 | organelle lumen | cellular\_component | 0.0 | 1.0 | 1.0 | 0 | cluster\_plot | 7 |
| 2243 | GO:0046907 | intracellular transport | biological\_process | 0.0 | 1.0 | 1.0 | 0 | cluster\_plot | 242 |
| 2244 | GO:0004645 | phosphorylase activity | molecular\_function | 0.0 | 1.0 | 1.0 | 0 | cluster\_plot | 4 |
| 2245 | GO:0046039 | GTP metabolic process | biological\_process | 0.0 | 1.0 | 1.0 | 0 | cluster\_plot | 29 |
| 2246 | GO:0010648 | negative regulation of cell communication | biological\_process | 0.0 | 1.0 | 1.0 | 0 | cluster\_plot | 2 |
| 2247 | GO:0018271 | biotin-protein ligase activity | molecular\_function | 0.0 | 1.0 | 1.0 | 0 | cluster\_plot | 3 |
| 2248 | GO:0009057 | macromolecule catabolic process | biological\_process | 0.0 | 1.0 | 1.0 | 0 | cluster\_plot | 252 |
| 2249 | GO:0009165 | nucleotide biosynthetic process | biological\_process | 0.0 | 1.0 | 1.0 | 0 | cluster\_plot | 130 |
| 2250 | GO:0015087 | cobalt ion transmembrane transporter activity | molecular\_function | 0.0 | 1.0 | 1.0 | 0 | cluster\_plot | 2 |
| 2251 | GO:0015740 | C4-dicarboxylate transport | biological\_process | 0.0 | 1.0 | 1.0 | 0 | cluster\_plot | 30 |
| 2252 | GO:0004421 | hydroxymethylglutaryl-CoA synthase activity | molecular\_function | 0.0 | 1.0 | 1.0 | 0 | cluster\_plot | 2 |
| 2253 | GO:0046080 | dUTP metabolic process | biological\_process | 0.0 | 1.0 | 1.0 | 0 | cluster\_plot | 1 |
| 2254 | GO:0001076 | RNA polymerase II transcription factor binding transcription factor activity | molecular\_function | 0.0 | 1.0 | 1.0 | 0 | cluster\_plot | 21 |
| 2255 | GO:0004536 | deoxyribonuclease activity | molecular\_function | 0.0 | 1.0 | 1.0 | 0 | cluster\_plot | 11 |
| 2256 | GO:0042819 | vitamin B6 biosynthetic process | biological\_process | 0.0 | 1.0 | 1.0 | 0 | cluster\_plot | 1 |
| 2257 | GO:0046351 | disaccharide biosynthetic process | biological\_process | 0.0 | 1.0 | 1.0 | 0 | cluster\_plot | 26 |
| 2258 | GO:0003980 | UDP-glucose:glycoprotein glucosyltransferase activity | molecular\_function | 0.0 | 1.0 | 1.0 | 0 | cluster\_plot | 2 |
| 2259 | GO:0044455 | mitochondrial membrane part | cellular\_component | 0.0 | 1.0 | 1.0 | 0 | cluster\_plot | 39 |
| 2260 | GO:0003873 | 6-phosphofructo-2-kinase activity | molecular\_function | 0.0 | 1.0 | 1.0 | 0 | cluster\_plot | 3 |
| 2261 | GO:0070013 | intracellular organelle lumen | cellular\_component | 0.0 | 1.0 | 1.0 | 0 | cluster\_plot | 7 |
| 2262 | GO:0006788 | heme oxidation | biological\_process | 0.0 | 1.0 | 1.0 | 0 | cluster\_plot | 3 |
| 2263 | GO:0006399 | tRNA metabolic process | biological\_process | 0.0 | 1.0 | 1.0 | 0 | cluster\_plot | 145 |
| 2264 | GO:0006644 | phospholipid metabolic process | biological\_process | 0.0 | 1.0 | 1.0 | 0 | cluster\_plot | 78 |
| 2265 | GO:0009029 | tetraacyldisaccharide 4'-kinase activity | molecular\_function | 0.0 | 1.0 | 1.0 | 0 | cluster\_plot | 1 |
| 2266 | GO:0009147 | pyrimidine nucleoside triphosphate metabolic process | biological\_process | 0.0 | 1.0 | 1.0 | 0 | cluster\_plot | 8 |
| 2267 | GO:0000247 | C-8 sterol isomerase activity | molecular\_function | 0.0 | 1.0 | 1.0 | 0 | cluster\_plot | 3 |
| 2268 | GO:0052592 | oxidoreductase activity, acting on CH or CH2 groups, with an iron-sulfur protein as acceptor | molecular\_function | 0.0 | 1.0 | 1.0 | 0 | cluster\_plot | 2 |
| 2269 | GO:0033124 | regulation of GTP catabolic process | biological\_process | 0.0 | 1.0 | 1.0 | 0 | cluster\_plot | 51 |
| 2270 | GO:0006206 | pyrimidine nucleobase metabolic process | biological\_process | 0.0 | 1.0 | 1.0 | 0 | cluster\_plot | 5 |
| 2271 | GO:0005852 | eukaryotic translation initiation factor 3 complex | cellular\_component | 0.0 | 1.0 | 1.0 | 0 | cluster\_plot | 11 |
| 2272 | GO:0006696 | ergosterol biosynthetic process | biological\_process | 0.0 | 1.0 | 1.0 | 0 | cluster\_plot | 3 |
| 2273 | GO:0030677 | ribonuclease P complex | cellular\_component | 0.0 | 1.0 | 1.0 | 0 | cluster\_plot | 1 |
| 2274 | GO:0004335 | galactokinase activity | molecular\_function | 0.0 | 1.0 | 1.0 | 0 | cluster\_plot | 1 |
| 2275 | GO:0016426 | tRNA (adenine) methyltransferase activity | molecular\_function | 0.0 | 1.0 | 1.0 | 0 | cluster\_plot | 3 |
| 2276 | GO:0000990 | core RNA polymerase binding transcription factor activity | molecular\_function | 0.0 | 1.0 | 1.0 | 0 | cluster\_plot | 12 |
| 2277 | GO:0045901 | positive regulation of translational elongation | biological\_process | 0.0 | 1.0 | 1.0 | 0 | cluster\_plot | 3 |
| 2278 | GO:0044815 | DNA packaging complex | cellular\_component | 0.0 | 1.0 | 1.0 | 0 | cluster\_plot | 24 |
| 2279 | GO:0002161 | aminoacyl-tRNA editing activity | molecular\_function | 0.0 | 1.0 | 1.0 | 0 | cluster\_plot | 6 |
| 2280 | GO:0019842 | vitamin binding | molecular\_function | 0.0 | 1.0 | 1.0 | 0 | cluster\_plot | 21 |
| 2281 | GO:0004363 | glutathione synthase activity | molecular\_function | 0.0 | 1.0 | 1.0 | 0 | cluster\_plot | 2 |
| 2282 | GO:0004356 | glutamate-ammonia ligase activity | molecular\_function | 0.0 | 1.0 | 1.0 | 0 | cluster\_plot | 6 |
| 2283 | GO:0048856 | anatomical structure development | biological\_process | 0.0 | 1.0 | 1.0 | 0 | cluster\_plot | 5 |
| 2284 | GO:0035312 | 5'-3' exodeoxyribonuclease activity | molecular\_function | 0.0 | 1.0 | 1.0 | 0 | cluster\_plot | 2 |
| 2285 | GO:0042435 | indole-containing compound biosynthetic process | biological\_process | 0.0 | 1.0 | 1.0 | 0 | cluster\_plot | 8 |
| 2286 | GO:0036442 | hydrogen-exporting ATPase activity | molecular\_function | 0.0 | 1.0 | 1.0 | 0 | cluster\_plot | 24 |
| 2287 | GO:0046365 | monosaccharide catabolic process | biological\_process | 0.0 | 1.0 | 1.0 | 0 | cluster\_plot | 17 |
| 2288 | GO:0042219 | cellular modified amino acid catabolic process | biological\_process | 0.0 | 1.0 | 1.0 | 0 | cluster\_plot | 1 |
| 2289 | GO:1901071 | glucosamine-containing compound metabolic process | biological\_process | 0.0 | 1.0 | 1.0 | 0 | cluster\_plot | 19 |
| 2290 | GO:0045017 | glycerolipid biosynthetic process | biological\_process | 0.0 | 1.0 | 1.0 | 0 | cluster\_plot | 29 |
| 2291 | GO:0008171 | O-methyltransferase activity | molecular\_function | 0.0 | 1.0 | 1.0 | 0 | cluster\_plot | 47 |
| 2292 | GO:0008198 | ferrous iron binding | molecular\_function | 0.0 | 1.0 | 1.0 | 0 | cluster\_plot | 3 |
| 2293 | GO:0016471 | vacuolar proton-transporting V-type ATPase complex | cellular\_component | 0.0 | 1.0 | 1.0 | 0 | cluster\_plot | 7 |
| 2294 | GO:0003840 | gamma-glutamyltransferase activity | molecular\_function | 0.0 | 1.0 | 1.0 | 0 | cluster\_plot | 6 |
| 2295 | GO:0008686 | 3,4-dihydroxy-2-butanone-4-phosphate synthase activity | molecular\_function | 0.0 | 1.0 | 1.0 | 0 | cluster\_plot | 3 |
| 2296 | GO:0004844 | uracil DNA N-glycosylase activity | molecular\_function | 0.0 | 1.0 | 1.0 | 0 | cluster\_plot | 1 |
| 2297 | GO:0034308 | primary alcohol metabolic process | biological\_process | 0.0 | 1.0 | 1.0 | 0 | cluster\_plot | 1 |
| 2298 | GO:0044432 | endoplasmic reticulum part | cellular\_component | 0.0 | 1.0 | 1.0 | 0 | cluster\_plot | 50 |
| 2299 | GO:0009177 | pyrimidine deoxyribonucleoside monophosphate biosynthetic process | biological\_process | 0.0 | 1.0 | 1.0 | 0 | cluster\_plot | 1 |
| 2300 | GO:0032934 | sterol binding | molecular\_function | 0.0 | 1.0 | 1.0 | 0 | cluster\_plot | 3 |
| 2301 | GO:0016641 | oxidoreductase activity, acting on the CH-NH2 group of donors, oxygen as acceptor | molecular\_function | 0.0 | 1.0 | 1.0 | 0 | cluster\_plot | 18 |
| 2302 | GO:0047746 | chlorophyllase activity | molecular\_function | 0.0 | 1.0 | 1.0 | 0 | cluster\_plot | 7 |
| 2303 | GO:0050136 | NADH dehydrogenase (quinone) activity | molecular\_function | 0.0 | 1.0 | 1.0 | 0 | cluster\_plot | 9 |
| 2304 | GO:0005885 | Arp2/3 protein complex | cellular\_component | 0.0 | 1.0 | 1.0 | 0 | cluster\_plot | 4 |
| 2305 | GO:0004012 | phospholipid-translocating ATPase activity | molecular\_function | 0.0 | 1.0 | 1.0 | 0 | cluster\_plot | 17 |
| 2306 | GO:0016875 | ligase activity, forming carbon-oxygen bonds | molecular\_function | 0.0 | 1.0 | 1.0 | 0 | cluster\_plot | 87 |
| 2307 | GO:0005681 | spliceosomal complex | cellular\_component | 0.0 | 1.0 | 1.0 | 0 | cluster\_plot | 8 |
| 2308 | GO:0046497 | nicotinate nucleotide metabolic process | biological\_process | 0.0 | 1.0 | 1.0 | 0 | cluster\_plot | 2 |
| 2309 | GO:0000774 | adenyl-nucleotide exchange factor activity | molecular\_function | 0.0 | 1.0 | 1.0 | 0 | cluster\_plot | 7 |
| 2310 | GO:0046364 | monosaccharide biosynthetic process | biological\_process | 0.0 | 1.0 | 1.0 | 0 | cluster\_plot | 11 |
| 2311 | GO:0015399 | primary active transmembrane transporter activity | molecular\_function | 0.0 | 1.0 | 1.0 | 0 | cluster\_plot | 194 |
| 2312 | GO:0000917 | barrier septum assembly | biological\_process | 0.0 | 1.0 | 1.0 | 0 | cluster\_plot | 3 |
| 2313 | GO:0003743 | translation initiation factor activity | molecular\_function | 0.0 | 1.0 | 1.0 | 0 | cluster\_plot | 48 |
| 2314 | GO:1901565 | organonitrogen compound catabolic process | biological\_process | 0.0 | 1.0 | 1.0 | 0 | cluster\_plot | 99 |
| 2315 | GO:0006284 | base-excision repair | biological\_process | 0.0 | 1.0 | 1.0 | 0 | cluster\_plot | 32 |
| 2316 | GO:0051259 | protein oligomerization | biological\_process | 0.0 | 1.0 | 1.0 | 0 | cluster\_plot | 16 |
| 2317 | GO:0010181 | FMN binding | molecular\_function | 0.0 | 1.0 | 1.0 | 0 | cluster\_plot | 36 |
| 2318 | GO:0030029 | actin filament-based process | biological\_process | 0.0 | 1.0 | 1.0 | 0 | cluster\_plot | 14 |
| 2319 | GO:0009584 | detection of visible light | biological\_process | 0.0 | 1.0 | 1.0 | 0 | cluster\_plot | 8 |
| 2320 | GO:0005811 | lipid particle | cellular\_component | 0.0 | 1.0 | 1.0 | 0 | cluster\_plot | 11 |
| 2321 | GO:0030132 | clathrin coat of coated pit | cellular\_component | 0.0 | 1.0 | 1.0 | 0 | cluster\_plot | 10 |
| 2322 | GO:0000178 | exosome (RNase complex) | cellular\_component | 0.0 | 1.0 | 1.0 | 0 | cluster\_plot | 4 |
| 2323 | GO:0004070 | aspartate carbamoyltransferase activity | molecular\_function | 0.0 | 1.0 | 1.0 | 0 | cluster\_plot | 1 |
| 2324 | GO:0017057 | 6-phosphogluconolactonase activity | molecular\_function | 0.0 | 1.0 | 1.0 | 0 | cluster\_plot | 3 |
| 2325 | GO:0046174 | polyol catabolic process | biological\_process | 0.0 | 1.0 | 1.0 | 0 | cluster\_plot | 9 |
| 2326 | GO:0004311 | farnesyltranstransferase activity | molecular\_function | 0.0 | 1.0 | 1.0 | 0 | cluster\_plot | 4 |
| 2327 | GO:0004457 | lactate dehydrogenase activity | molecular\_function | 0.0 | 1.0 | 1.0 | 0 | cluster\_plot | 2 |
| 2328 | GO:0051784 | negative regulation of nuclear division | biological\_process | 0.0 | 1.0 | 1.0 | 0 | cluster\_plot | 2 |
| 2329 | GO:0007017 | microtubule-based process | biological\_process | 0.0 | 1.0 | 1.0 | 0 | cluster\_plot | 133 |
| 2330 | GO:0004590 | orotidine-5'-phosphate decarboxylase activity | molecular\_function | 0.0 | 1.0 | 1.0 | 0 | cluster\_plot | 1 |
| 2331 | GO:0004146 | dihydrofolate reductase activity | molecular\_function | 0.0 | 1.0 | 1.0 | 0 | cluster\_plot | 1 |
| 2332 | GO:0051139 | metal ion:proton antiporter activity | molecular\_function | 0.0 | 1.0 | 1.0 | 0 | cluster\_plot | 12 |
| 2333 | GO:0055067 | monovalent inorganic cation homeostasis | biological\_process | 0.0 | 1.0 | 1.0 | 0 | cluster\_plot | 5 |
| 2334 | GO:0030125 | clathrin vesicle coat | cellular\_component | 0.0 | 1.0 | 1.0 | 0 | cluster\_plot | 10 |
| 2335 | GO:0006402 | mRNA catabolic process | biological\_process | 0.0 | 1.0 | 1.0 | 0 | cluster\_plot | 6 |
| 2336 | GO:0018024 | histone-lysine N-methyltransferase activity | molecular\_function | 0.0 | 1.0 | 1.0 | 0 | cluster\_plot | 18 |
| 2337 | GO:0009262 | deoxyribonucleotide metabolic process | biological\_process | 0.0 | 1.0 | 1.0 | 0 | cluster\_plot | 4 |
| 2338 | GO:0019205 | nucleobase-containing compound kinase activity | molecular\_function | 0.0 | 1.0 | 1.0 | 0 | cluster\_plot | 47 |
| 2339 | GO:0003906 | DNA-(apurinic or apyrimidinic site) lyase activity | molecular\_function | 0.0 | 1.0 | 1.0 | 0 | cluster\_plot | 3 |
| 2340 | GO:0016886 | ligase activity, forming phosphoric ester bonds | molecular\_function | 0.0 | 1.0 | 1.0 | 0 | cluster\_plot | 4 |
| 2341 | GO:0019310 | inositol catabolic process | biological\_process | 0.0 | 1.0 | 1.0 | 0 | cluster\_plot | 9 |
| 2342 | GO:0009396 | folic acid-containing compound biosynthetic process | biological\_process | 0.0 | 1.0 | 1.0 | 0 | cluster\_plot | 19 |
| 2343 | GO:0009450 | gamma-aminobutyric acid catabolic process | biological\_process | 0.0 | 1.0 | 1.0 | 0 | cluster\_plot | 2 |
| 2344 | GO:0012501 | programmed cell death | biological\_process | 0.0 | 1.0 | 1.0 | 0 | cluster\_plot | 1 |
| 2345 | GO:0031090 | organelle membrane | cellular\_component | 0.0 | 1.0 | 1.0 | 0 | cluster\_plot | 68 |
| 2346 | GO:0051495 | positive regulation of cytoskeleton organization | biological\_process | 0.0 | 1.0 | 1.0 | 0 | cluster\_plot | 4 |
| 2347 | GO:1990351 | transporter complex | cellular\_component | 0.0 | 1.0 | 1.0 | 0 | cluster\_plot | 5 |
| 2348 | GO:0016833 | oxo-acid-lyase activity | molecular\_function | 0.0 | 1.0 | 1.0 | 0 | cluster\_plot | 12 |
| 2349 | GO:0006888 | ER to Golgi vesicle-mediated transport | biological\_process | 0.0 | 1.0 | 1.0 | 0 | cluster\_plot | 35 |
| 2350 | GO:0000502 | proteasome complex | cellular\_component | 0.0 | 1.0 | 1.0 | 0 | cluster\_plot | 2 |
| 2351 | GO:0042555 | MCM complex | cellular\_component | 0.0 | 1.0 | 1.0 | 0 | cluster\_plot | 3 |
| 2352 | GO:0009664 | plant-type cell wall organization | biological\_process | 0.0 | 1.0 | 1.0 | 0 | cluster\_plot | 1 |
| 2353 | GO:0006771 | riboflavin metabolic process | biological\_process | 0.0 | 1.0 | 1.0 | 0 | cluster\_plot | 13 |
| 2354 | GO:0010605 | negative regulation of macromolecule metabolic process | biological\_process | 0.0 | 1.0 | 1.0 | 0 | cluster\_plot | 31 |
| 2355 | GO:0046219 | indolalkylamine biosynthetic process | biological\_process | 0.0 | 1.0 | 1.0 | 0 | cluster\_plot | 8 |
| 2356 | GO:0016755 | transferase activity, transferring amino-acyl groups | molecular\_function | 0.0 | 1.0 | 1.0 | 0 | cluster\_plot | 11 |
| 2357 | GO:0046148 | pigment biosynthetic process | biological\_process | 0.0 | 1.0 | 1.0 | 0 | cluster\_plot | 47 |
| 2358 | GO:0046417 | chorismate metabolic process | biological\_process | 0.0 | 1.0 | 1.0 | 0 | cluster\_plot | 2 |
| 2359 | GO:0046072 | dTDP metabolic process | biological\_process | 0.0 | 1.0 | 1.0 | 0 | cluster\_plot | 2 |
| 2360 | GO:0003993 | acid phosphatase activity | molecular\_function | 0.0 | 1.0 | 1.0 | 0 | cluster\_plot | 16 |
| 2361 | GO:0009086 | methionine biosynthetic process | biological\_process | 0.0 | 1.0 | 1.0 | 0 | cluster\_plot | 11 |
| 2362 | GO:1901891 | regulation of cell septum assembly | biological\_process | 0.0 | 1.0 | 1.0 | 0 | cluster\_plot | 1 |
| 2363 | GO:0032313 | regulation of Rab GTPase activity | biological\_process | 0.0 | 1.0 | 1.0 | 0 | cluster\_plot | 23 |
| 2364 | GO:1901701 | cellular response to oxygen-containing compound | biological\_process | 0.0 | 1.0 | 1.0 | 0 | cluster\_plot | 1 |
| 2365 | GO:0006658 | phosphatidylserine metabolic process | biological\_process | 0.0 | 1.0 | 1.0 | 0 | cluster\_plot | 2 |
| 2366 | GO:0005887 | integral component of plasma membrane | cellular\_component | 0.0 | 1.0 | 1.0 | 0 | cluster\_plot | 6 |
| 2367 | GO:0004108 | citrate (Si)-synthase activity | molecular\_function | 0.0 | 1.0 | 1.0 | 0 | cluster\_plot | 2 |
| 2368 | GO:0042742 | defense response to bacterium | biological\_process | 0.0 | 1.0 | 1.0 | 0 | cluster\_plot | 1 |
| 2369 | GO:0016783 | sulfurtransferase activity | molecular\_function | 0.0 | 1.0 | 1.0 | 0 | cluster\_plot | 9 |
| 2370 | GO:0008495 | protoheme IX farnesyltransferase activity | molecular\_function | 0.0 | 1.0 | 1.0 | 0 | cluster\_plot | 2 |
| 2371 | GO:0007094 | mitotic spindle assembly checkpoint | biological\_process | 0.0 | 1.0 | 1.0 | 0 | cluster\_plot | 2 |
| 2372 | GO:0016802 | trialkylsulfonium hydrolase activity | molecular\_function | 0.0 | 1.0 | 1.0 | 0 | cluster\_plot | 2 |
| 2373 | GO:0006753 | nucleoside phosphate metabolic process | biological\_process | 0.0 | 1.0 | 1.0 | 0 | cluster\_plot | 220 |
| 2374 | GO:0019899 | enzyme binding | molecular\_function | 0.0 | 1.0 | 1.0 | 0 | cluster\_plot | 49 |
| 2375 | GO:0008977 | prephenate dehydrogenase activity | molecular\_function | 0.0 | 1.0 | 1.0 | 0 | cluster\_plot | 4 |
| 2376 | GO:0015631 | tubulin binding | molecular\_function | 0.0 | 1.0 | 1.0 | 0 | cluster\_plot | 116 |
| 2377 | GO:0072525 | pyridine-containing compound biosynthetic process | biological\_process | 0.0 | 1.0 | 1.0 | 0 | cluster\_plot | 24 |
| 2378 | GO:0008320 | protein transmembrane transporter activity | molecular\_function | 0.0 | 1.0 | 1.0 | 0 | cluster\_plot | 7 |
| 2379 | GO:0009617 | response to bacterium | biological\_process | 0.0 | 1.0 | 1.0 | 0 | cluster\_plot | 1 |
| 2380 | GO:0044431 | Golgi apparatus part | cellular\_component | 0.0 | 1.0 | 1.0 | 0 | cluster\_plot | 55 |
| 2381 | GO:0034728 | nucleosome organization | biological\_process | 0.0 | 1.0 | 1.0 | 0 | cluster\_plot | 29 |
| 2382 | GO:0008757 | S-adenosylmethionine-dependent methyltransferase activity | molecular\_function | 0.0 | 1.0 | 1.0 | 0 | cluster\_plot | 47 |
| 2383 | GO:0004424 | imidazoleglycerol-phosphate dehydratase activity | molecular\_function | 0.0 | 1.0 | 1.0 | 0 | cluster\_plot | 2 |
| 2384 | GO:0033862 | UMP kinase activity | molecular\_function | 0.0 | 1.0 | 1.0 | 0 | cluster\_plot | 2 |
| 2385 | GO:0034035 | purine ribonucleoside bisphosphate metabolic process | biological\_process | 0.0 | 1.0 | 1.0 | 0 | cluster\_plot | 5 |
| 2386 | GO:0044454 | nuclear chromosome part | cellular\_component | 0.0 | 1.0 | 1.0 | 0 | cluster\_plot | 10 |
| 2387 | GO:0004150 | dihydroneopterin aldolase activity | molecular\_function | 0.0 | 1.0 | 1.0 | 0 | cluster\_plot | 2 |
| 2388 | GO:0043624 | cellular protein complex disassembly | biological\_process | 0.0 | 1.0 | 1.0 | 0 | cluster\_plot | 13 |
| 2389 | GO:0006650 | glycerophospholipid metabolic process | biological\_process | 0.0 | 1.0 | 1.0 | 0 | cluster\_plot | 57 |
| 2390 | GO:0030488 | tRNA methylation | biological\_process | 0.0 | 1.0 | 1.0 | 0 | cluster\_plot | 3 |
| 2391 | GO:0006816 | calcium ion transport | biological\_process | 0.0 | 1.0 | 1.0 | 0 | cluster\_plot | 38 |
| 2392 | GO:0071203 | WASH complex | cellular\_component | 0.0 | 1.0 | 1.0 | 0 | cluster\_plot | 6 |
| 2393 | GO:0031011 | Ino80 complex | cellular\_component | 0.0 | 1.0 | 1.0 | 0 | cluster\_plot | 4 |
| 2394 | GO:0005664 | nuclear origin of replication recognition complex | cellular\_component | 0.0 | 1.0 | 1.0 | 0 | cluster\_plot | 3 |
| 2395 | GO:0008092 | cytoskeletal protein binding | molecular\_function | 0.0 | 1.0 | 1.0 | 0 | cluster\_plot | 150 |
| 2396 | GO:0008425 | 2-polyprenyl-6-methoxy-1,4-benzoquinone methyltransferase activity | molecular\_function | 0.0 | 1.0 | 1.0 | 0 | cluster\_plot | 2 |
| 2397 | GO:0004334 | fumarylacetoacetase activity | molecular\_function | 0.0 | 1.0 | 1.0 | 0 | cluster\_plot | 1 |
| 2398 | GO:0090151 | establishment of protein localization to mitochondrial membrane | biological\_process | 0.0 | 1.0 | 1.0 | 0 | cluster\_plot | 10 |
| 2399 | GO:0006305 | DNA alkylation | biological\_process | 0.0 | 1.0 | 1.0 | 0 | cluster\_plot | 15 |
| 2400 | GO:0008137 | NADH dehydrogenase (ubiquinone) activity | molecular\_function | 0.0 | 1.0 | 1.0 | 0 | cluster\_plot | 9 |
| 2401 | GO:0015491 | cation:cation antiporter activity | molecular\_function | 0.0 | 1.0 | 1.0 | 0 | cluster\_plot | 17 |
| 2402 | GO:0051223 | regulation of protein transport | biological\_process | 0.0 | 1.0 | 1.0 | 0 | cluster\_plot | 2 |
| 2403 | GO:0044030 | regulation of DNA methylation | biological\_process | 0.0 | 1.0 | 1.0 | 0 | cluster\_plot | 3 |
| 2404 | GO:0004617 | phosphoglycerate dehydrogenase activity | molecular\_function | 0.0 | 1.0 | 1.0 | 0 | cluster\_plot | 5 |
| 2405 | GO:0004164 | diphthine synthase activity | molecular\_function | 0.0 | 1.0 | 1.0 | 0 | cluster\_plot | 1 |
| 2406 | GO:0016843 | amine-lyase activity | molecular\_function | 0.0 | 1.0 | 1.0 | 0 | cluster\_plot | 13 |
| 2407 | GO:0005097 | Rab GTPase activator activity | molecular\_function | 0.0 | 1.0 | 1.0 | 0 | cluster\_plot | 25 |
| 2408 | GO:0031683 | G-protein beta/gamma-subunit complex binding | molecular\_function | 0.0 | 1.0 | 1.0 | 0 | cluster\_plot | 8 |
| 2409 | GO:0009439 | cyanate metabolic process | biological\_process | 0.0 | 1.0 | 1.0 | 0 | cluster\_plot | 1 |
| 2410 | GO:0050080 | malonyl-CoA decarboxylase activity | molecular\_function | 0.0 | 1.0 | 1.0 | 0 | cluster\_plot | 1 |
| 2411 | GO:0004370 | glycerol kinase activity | molecular\_function | 0.0 | 1.0 | 1.0 | 0 | cluster\_plot | 3 |
| 2412 | GO:0044796 | DNA polymerase processivity factor complex | cellular\_component | 0.0 | 1.0 | 1.0 | 0 | cluster\_plot | 3 |
| 2413 | GO:0010285 | L,L-diaminopimelate aminotransferase activity | molecular\_function | 0.0 | 1.0 | 1.0 | 0 | cluster\_plot | 4 |
| 2414 | GO:0009316 | 3-isopropylmalate dehydratase complex | cellular\_component | 0.0 | 1.0 | 1.0 | 0 | cluster\_plot | 1 |
| 2415 | GO:0004588 | orotate phosphoribosyltransferase activity | molecular\_function | 0.0 | 1.0 | 1.0 | 0 | cluster\_plot | 1 |
| 2416 | GO:0044247 | cellular polysaccharide catabolic process | biological\_process | 0.0 | 1.0 | 1.0 | 0 | cluster\_plot | 3 |
| 2417 | GO:0004333 | fumarate hydratase activity | molecular\_function | 0.0 | 1.0 | 1.0 | 0 | cluster\_plot | 1 |
| 2418 | GO:0006094 | gluconeogenesis | biological\_process | 0.0 | 1.0 | 1.0 | 0 | cluster\_plot | 10 |
| 2419 | GO:0007093 | mitotic cell cycle checkpoint | biological\_process | 0.0 | 1.0 | 1.0 | 0 | cluster\_plot | 2 |
| 2420 | GO:0019359 | nicotinamide nucleotide biosynthetic process | biological\_process | 0.0 | 1.0 | 1.0 | 0 | cluster\_plot | 16 |
| 2421 | GO:0006664 | glycolipid metabolic process | biological\_process | 0.0 | 1.0 | 1.0 | 0 | cluster\_plot | 30 |
| 2422 | GO:0006415 | translational termination | biological\_process | 0.0 | 1.0 | 1.0 | 0 | cluster\_plot | 13 |
| 2423 | GO:0006097 | glyoxylate cycle | biological\_process | 0.0 | 1.0 | 1.0 | 0 | cluster\_plot | 3 |
| 2424 | GO:0004620 | phospholipase activity | molecular\_function | 0.0 | 1.0 | 1.0 | 0 | cluster\_plot | 7 |
| 2425 | GO:0016849 | phosphorus-oxygen lyase activity | molecular\_function | 0.0 | 1.0 | 1.0 | 0 | cluster\_plot | 2 |
| 2426 | GO:0009349 | riboflavin synthase complex | cellular\_component | 0.0 | 1.0 | 1.0 | 0 | cluster\_plot | 1 |
| 2427 | GO:0031047 | gene silencing by RNA | biological\_process | 0.0 | 1.0 | 1.0 | 0 | cluster\_plot | 11 |
| 2428 | GO:0009893 | positive regulation of metabolic process | biological\_process | 0.0 | 1.0 | 1.0 | 0 | cluster\_plot | 8 |
| 2429 | GO:0008478 | pyridoxal kinase activity | molecular\_function | 0.0 | 1.0 | 1.0 | 0 | cluster\_plot | 1 |
| 2430 | GO:0006486 | protein glycosylation | biological\_process | 0.0 | 1.0 | 1.0 | 0 | cluster\_plot | 61 |
| 2431 | GO:0019008 | molybdopterin synthase complex | cellular\_component | 0.0 | 1.0 | 1.0 | 0 | cluster\_plot | 1 |
| 2432 | GO:0033875 | ribonucleoside bisphosphate metabolic process | biological\_process | 0.0 | 1.0 | 1.0 | 0 | cluster\_plot | 12 |
| 2433 | GO:0050801 | ion homeostasis | biological\_process | 0.0 | 1.0 | 1.0 | 0 | cluster\_plot | 11 |
| 2434 | GO:0005985 | sucrose metabolic process | biological\_process | 0.0 | 1.0 | 1.0 | 0 | cluster\_plot | 35 |
| 2435 | GO:0004045 | aminoacyl-tRNA hydrolase activity | molecular\_function | 0.0 | 1.0 | 1.0 | 0 | cluster\_plot | 11 |
| 2436 | GO:0034033 | purine nucleoside bisphosphate biosynthetic process | biological\_process | 0.0 | 1.0 | 1.0 | 0 | cluster\_plot | 2 |
| 2437 | GO:0016880 | acid-ammonia (or amide) ligase activity | molecular\_function | 0.0 | 1.0 | 1.0 | 0 | cluster\_plot | 6 |
| 2438 | GO:0015936 | coenzyme A metabolic process | biological\_process | 0.0 | 1.0 | 1.0 | 0 | cluster\_plot | 7 |
| 2439 | GO:0006425 | glutaminyl-tRNA aminoacylation | biological\_process | 0.0 | 1.0 | 1.0 | 0 | cluster\_plot | 3 |
| 2440 | GO:0009611 | response to wounding | biological\_process | 0.0 | 1.0 | 1.0 | 0 | cluster\_plot | 8 |
| 2441 | GO:0031071 | cysteine desulfurase activity | molecular\_function | 0.0 | 1.0 | 1.0 | 0 | cluster\_plot | 3 |
| 2442 | GO:0016869 | intramolecular transferase activity, transferring amino groups | molecular\_function | 0.0 | 1.0 | 1.0 | 0 | cluster\_plot | 1 |
| 2443 | GO:0008536 | Ran GTPase binding | molecular\_function | 0.0 | 1.0 | 1.0 | 0 | cluster\_plot | 19 |
| 2444 | GO:0031329 | regulation of cellular catabolic process | biological\_process | 0.0 | 1.0 | 1.0 | 0 | cluster\_plot | 52 |
| 2445 | GO:0044462 | external encapsulating structure part | cellular\_component | 0.0 | 1.0 | 1.0 | 0 | cluster\_plot | 2 |
| 2446 | GO:0017171 | serine hydrolase activity | molecular\_function | 0.0 | 1.0 | 1.0 | 0 | cluster\_plot | 212 |
| 2447 | GO:0016703 | oxidoreductase activity, acting on single donors with incorporation of molecular oxygen, incorporation of one atom of oxygen (internal monooxygenases or internal mixed function oxidases) | molecular\_function | 0.0 | 1.0 | 1.0 | 0 | cluster\_plot | 9 |
| 2448 | GO:0004345 | glucose-6-phosphate dehydrogenase activity | molecular\_function | 0.0 | 1.0 | 1.0 | 0 | cluster\_plot | 7 |
| 2449 | GO:0048523 | negative regulation of cellular process | biological\_process | 0.0 | 1.0 | 1.0 | 0 | cluster\_plot | 35 |
| 2450 | GO:0030151 | molybdenum ion binding | molecular\_function | 0.0 | 1.0 | 1.0 | 0 | cluster\_plot | 8 |
| 2451 | GO:0010564 | regulation of cell cycle process | biological\_process | 0.0 | 1.0 | 1.0 | 0 | cluster\_plot | 6 |
| 2452 | GO:0006739 | NADP metabolic process | biological\_process | 0.0 | 1.0 | 1.0 | 0 | cluster\_plot | 21 |
| 2453 | GO:0004349 | glutamate 5-kinase activity | molecular\_function | 0.0 | 1.0 | 1.0 | 0 | cluster\_plot | 2 |
| 2454 | GO:0046349 | amino sugar biosynthetic process | biological\_process | 0.0 | 1.0 | 1.0 | 0 | cluster\_plot | 2 |
| 2455 | GO:0009145 | purine nucleoside triphosphate biosynthetic process | biological\_process | 0.0 | 1.0 | 1.0 | 0 | cluster\_plot | 56 |
| 2456 | GO:0008964 | phosphoenolpyruvate carboxylase activity | molecular\_function | 0.0 | 1.0 | 1.0 | 0 | cluster\_plot | 8 |
| 2457 | GO:0016979 | lipoate-protein ligase activity | molecular\_function | 0.0 | 1.0 | 1.0 | 0 | cluster\_plot | 4 |
| 2458 | GO:0016675 | oxidoreductase activity, acting on a heme group of donors | molecular\_function | 0.0 | 1.0 | 1.0 | 0 | cluster\_plot | 21 |
| 2459 | GO:0016832 | aldehyde-lyase activity | molecular\_function | 0.0 | 1.0 | 1.0 | 0 | cluster\_plot | 14 |
| 2460 | GO:0051189 | prosthetic group metabolic process | biological\_process | 0.0 | 1.0 | 1.0 | 0 | cluster\_plot | 10 |
| 2461 | GO:0048583 | regulation of response to stimulus | biological\_process | 0.0 | 1.0 | 1.0 | 0 | cluster\_plot | 20 |
| 2462 | GO:0022884 | macromolecule transmembrane transporter activity | molecular\_function | 0.0 | 1.0 | 1.0 | 0 | cluster\_plot | 7 |
| 2463 | GO:0006952 | defense response | biological\_process | 0.0 | 1.0 | 1.0 | 0 | cluster\_plot | 131 |
| 2464 | GO:0006556 | S-adenosylmethionine biosynthetic process | biological\_process | 0.0 | 1.0 | 1.0 | 0 | cluster\_plot | 8 |
| 2465 | GO:0003747 | translation release factor activity | molecular\_function | 0.0 | 1.0 | 1.0 | 0 | cluster\_plot | 13 |
| 2466 | GO:0016129 | phytosteroid biosynthetic process | biological\_process | 0.0 | 1.0 | 1.0 | 0 | cluster\_plot | 3 |
| 2467 | GO:0034357 | photosynthetic membrane | cellular\_component | 0.0 | 1.0 | 1.0 | 0 | cluster\_plot | 8 |
| 2468 | GO:0022607 | cellular component assembly | biological\_process | 0.0 | 1.0 | 1.0 | 0 | cluster\_plot | 106 |
| 2469 | GO:0042823 | pyridoxal phosphate biosynthetic process | biological\_process | 0.0 | 1.0 | 1.0 | 0 | cluster\_plot | 7 |
| 2470 | GO:0016762 | xyloglucan:xyloglucosyl transferase activity | molecular\_function | 0.0 | 1.0 | 1.0 | 0 | cluster\_plot | 47 |
| 2471 | GO:0007264 | small GTPase mediated signal transduction | biological\_process | 0.0 | 1.0 | 1.0 | 0 | cluster\_plot | 109 |
| 2472 | GO:0019202 | amino acid kinase activity | molecular\_function | 0.0 | 1.0 | 1.0 | 0 | cluster\_plot | 8 |
| 2473 | GO:0031012 | extracellular matrix | cellular\_component | 0.0 | 1.0 | 1.0 | 0 | cluster\_plot | 8 |
| 2474 | GO:0010215 | cellulose microfibril organization | biological\_process | 0.0 | 1.0 | 1.0 | 0 | cluster\_plot | 17 |
| 2475 | GO:0016482 | cytoplasmic transport | biological\_process | 0.0 | 1.0 | 1.0 | 0 | cluster\_plot | 77 |
| 2476 | GO:0008094 | DNA-dependent ATPase activity | molecular\_function | 0.0 | 1.0 | 1.0 | 0 | cluster\_plot | 29 |
| 2477 | GO:0006084 | acetyl-CoA metabolic process | biological\_process | 0.0 | 1.0 | 1.0 | 0 | cluster\_plot | 2 |
| 2478 | GO:0046184 | aldehyde biosynthetic process | biological\_process | 0.0 | 1.0 | 1.0 | 0 | cluster\_plot | 7 |
| 2479 | GO:0016790 | thiolester hydrolase activity | molecular\_function | 0.0 | 1.0 | 1.0 | 0 | cluster\_plot | 84 |
| 2480 | GO:0071103 | DNA conformation change | biological\_process | 0.0 | 1.0 | 1.0 | 0 | cluster\_plot | 18 |
| 2481 | GO:0046130 | purine ribonucleoside catabolic process | biological\_process | 0.0 | 1.0 | 1.0 | 0 | cluster\_plot | 41 |
| 2482 | GO:0030198 | extracellular matrix organization | biological\_process | 0.0 | 1.0 | 1.0 | 0 | cluster\_plot | 17 |
| 2483 | GO:0019321 | pentose metabolic process | biological\_process | 0.0 | 1.0 | 1.0 | 0 | cluster\_plot | 5 |
| 2484 | GO:0030145 | manganese ion binding | molecular\_function | 0.0 | 1.0 | 1.0 | 0 | cluster\_plot | 9 |
| 2485 | GO:0004448 | isocitrate dehydrogenase activity | molecular\_function | 0.0 | 1.0 | 1.0 | 0 | cluster\_plot | 10 |
| 2486 | GO:0044270 | cellular nitrogen compound catabolic process | biological\_process | 0.0 | 1.0 | 1.0 | 0 | cluster\_plot | 61 |
| 2487 | GO:0016673 | oxidoreductase activity, acting on a sulfur group of donors, iron-sulfur protein as acceptor | molecular\_function | 0.0 | 1.0 | 1.0 | 0 | cluster\_plot | 3 |
| 2488 | GO:0042726 | flavin-containing compound metabolic process | biological\_process | 0.0 | 1.0 | 1.0 | 0 | cluster\_plot | 13 |
| 2489 | GO:0004849 | uridine kinase activity | molecular\_function | 0.0 | 1.0 | 1.0 | 0 | cluster\_plot | 2 |
| 2490 | GO:0006429 | leucyl-tRNA aminoacylation | biological\_process | 0.0 | 1.0 | 1.0 | 0 | cluster\_plot | 4 |
| 2491 | GO:1901681 | sulfur compound binding | molecular\_function | 0.0 | 1.0 | 1.0 | 0 | cluster\_plot | 22 |
| 2492 | GO:0008064 | regulation of actin polymerization or depolymerization | biological\_process | 0.0 | 1.0 | 1.0 | 0 | cluster\_plot | 10 |
| 2493 | GO:0043130 | ubiquitin binding | molecular\_function | 0.0 | 1.0 | 1.0 | 0 | cluster\_plot | 1 |
| 2494 | GO:0042802 | identical protein binding | molecular\_function | 0.0 | 1.0 | 1.0 | 0 | cluster\_plot | 83 |
| 2495 | GO:0031975 | envelope | cellular\_component | 0.0 | 1.0 | 1.0 | 0 | cluster\_plot | 7 |
| 2496 | GO:0051260 | protein homooligomerization | biological\_process | 0.0 | 1.0 | 1.0 | 0 | cluster\_plot | 14 |
| 2497 | GO:0019319 | hexose biosynthetic process | biological\_process | 0.0 | 1.0 | 1.0 | 0 | cluster\_plot | 11 |
| 2498 | GO:0017016 | Ras GTPase binding | molecular\_function | 0.0 | 1.0 | 1.0 | 0 | cluster\_plot | 19 |
| 2499 | GO:0015405 | P-P-bond-hydrolysis-driven transmembrane transporter activity | molecular\_function | 0.0 | 1.0 | 1.0 | 0 | cluster\_plot | 194 |
| 2500 | GO:0004049 | anthranilate synthase activity | molecular\_function | 0.0 | 1.0 | 1.0 | 0 | cluster\_plot | 5 |
| 2501 | GO:0065009 | regulation of molecular function | biological\_process | 0.0 | 1.0 | 1.0 | 0 | cluster\_plot | 139 |
| 2502 | GO:0004807 | triose-phosphate isomerase activity | molecular\_function | 0.0 | 1.0 | 1.0 | 0 | cluster\_plot | 7 |
| 2503 | GO:0018279 | protein N-linked glycosylation via asparagine | biological\_process | 0.0 | 1.0 | 1.0 | 0 | cluster\_plot | 6 |
| 2504 | GO:0009245 | lipid A biosynthetic process | biological\_process | 0.0 | 1.0 | 1.0 | 0 | cluster\_plot | 3 |
| 2505 | GO:0009234 | menaquinone biosynthetic process | biological\_process | 0.0 | 1.0 | 1.0 | 0 | cluster\_plot | 1 |
| 2506 | GO:0051173 | positive regulation of nitrogen compound metabolic process | biological\_process | 0.0 | 1.0 | 1.0 | 0 | cluster\_plot | 4 |
| 2507 | GO:0009166 | nucleotide catabolic process | biological\_process | 0.0 | 1.0 | 1.0 | 0 | cluster\_plot | 41 |
| 2508 | GO:0009240 | isopentenyl diphosphate biosynthetic process | biological\_process | 0.0 | 1.0 | 1.0 | 0 | cluster\_plot | 2 |
| 2509 | GO:0007166 | cell surface receptor signaling pathway | biological\_process | 0.0 | 1.0 | 1.0 | 0 | cluster\_plot | 32 |
| 2510 | GO:0004738 | pyruvate dehydrogenase activity | molecular\_function | 0.0 | 1.0 | 1.0 | 0 | cluster\_plot | 1 |
| 2511 | GO:0005795 | Golgi stack | cellular\_component | 0.0 | 1.0 | 1.0 | 0 | cluster\_plot | 8 |
| 2512 | GO:0003725 | double-stranded RNA binding | molecular\_function | 0.0 | 1.0 | 1.0 | 0 | cluster\_plot | 2 |
| 2513 | GO:0043733 | DNA-3-methylbase glycosylase activity | molecular\_function | 0.0 | 1.0 | 1.0 | 0 | cluster\_plot | 10 |
| 2514 | GO:0009128 | purine nucleoside monophosphate catabolic process | biological\_process | 0.0 | 1.0 | 1.0 | 0 | cluster\_plot | 19 |
| 2515 | GO:0031970 | organelle envelope lumen | cellular\_component | 0.0 | 1.0 | 1.0 | 0 | cluster\_plot | 2 |
| 2516 | GO:0071806 | protein transmembrane transport | biological\_process | 0.0 | 1.0 | 1.0 | 0 | cluster\_plot | 13 |
| 2517 | GO:0009063 | cellular amino acid catabolic process | biological\_process | 0.0 | 1.0 | 1.0 | 0 | cluster\_plot | 30 |
| 2518 | GO:0009308 | amine metabolic process | biological\_process | 0.0 | 1.0 | 1.0 | 0 | cluster\_plot | 55 |
| 2519 | GO:0022618 | ribonucleoprotein complex assembly | biological\_process | 0.0 | 1.0 | 1.0 | 0 | cluster\_plot | 2 |
| 2520 | GO:0006012 | galactose metabolic process | biological\_process | 0.0 | 1.0 | 1.0 | 0 | cluster\_plot | 9 |
| 2521 | GO:0001101 | response to acid | biological\_process | 0.0 | 1.0 | 1.0 | 0 | cluster\_plot | 7 |
| 2522 | GO:0000156 | phosphorelay response regulator activity | molecular\_function | 0.0 | 1.0 | 1.0 | 0 | cluster\_plot | 55 |
| 2523 | GO:0017003 | protein-heme linkage | biological\_process | 0.0 | 1.0 | 1.0 | 0 | cluster\_plot | 1 |
| 2524 | GO:0032200 | telomere organization | biological\_process | 0.0 | 1.0 | 1.0 | 0 | cluster\_plot | 3 |
| 2525 | GO:0048500 | signal recognition particle | cellular\_component | 0.0 | 1.0 | 1.0 | 0 | cluster\_plot | 13 |
| 2526 | GO:0005338 | nucleotide-sugar transmembrane transporter activity | molecular\_function | 0.0 | 1.0 | 1.0 | 0 | cluster\_plot | 9 |
| 2527 | GO:0016730 | oxidoreductase activity, acting on iron-sulfur proteins as donors | molecular\_function | 0.0 | 1.0 | 1.0 | 0 | cluster\_plot | 1 |
| 2528 | GO:0070069 | cytochrome complex | cellular\_component | 0.0 | 1.0 | 1.0 | 0 | cluster\_plot | 11 |
| 2529 | GO:1990204 | oxidoreductase complex | cellular\_component | 0.0 | 1.0 | 1.0 | 0 | cluster\_plot | 49 |
| 2530 | GO:0015918 | sterol transport | biological\_process | 0.0 | 1.0 | 1.0 | 0 | cluster\_plot | 1 |
| 2531 | GO:0004826 | phenylalanine-tRNA ligase activity | molecular\_function | 0.0 | 1.0 | 1.0 | 0 | cluster\_plot | 7 |
| 2532 | GO:0051920 | peroxiredoxin activity | molecular\_function | 0.0 | 1.0 | 1.0 | 0 | cluster\_plot | 5 |
| 2533 | GO:0000175 | 3'-5'-exoribonuclease activity | molecular\_function | 0.0 | 1.0 | 1.0 | 0 | cluster\_plot | 1 |
| 2534 | GO:0004158 | dihydroorotate oxidase activity | molecular\_function | 0.0 | 1.0 | 1.0 | 0 | cluster\_plot | 4 |
| 2535 | GO:0043467 | regulation of generation of precursor metabolites and energy | biological\_process | 0.0 | 1.0 | 1.0 | 0 | cluster\_plot | 4 |
| 2536 | GO:0016598 | protein arginylation | biological\_process | 0.0 | 1.0 | 1.0 | 0 | cluster\_plot | 2 |
| 2537 | GO:0004637 | phosphoribosylamine-glycine ligase activity | molecular\_function | 0.0 | 1.0 | 1.0 | 0 | cluster\_plot | 2 |
| 2538 | GO:0009167 | purine ribonucleoside monophosphate metabolic process | biological\_process | 0.0 | 1.0 | 1.0 | 0 | cluster\_plot | 79 |
| 2539 | GO:0045239 | tricarboxylic acid cycle enzyme complex | cellular\_component | 0.0 | 1.0 | 1.0 | 0 | cluster\_plot | 4 |
| 2540 | GO:0003852 | 2-isopropylmalate synthase activity | molecular\_function | 0.0 | 1.0 | 1.0 | 0 | cluster\_plot | 3 |
| 2541 | GO:0003678 | DNA helicase activity | molecular\_function | 0.0 | 1.0 | 1.0 | 0 | cluster\_plot | 20 |
| 2542 | GO:0009011 | starch synthase activity | molecular\_function | 0.0 | 1.0 | 1.0 | 0 | cluster\_plot | 9 |
| 2543 | GO:0008235 | metalloexopeptidase activity | molecular\_function | 0.0 | 1.0 | 1.0 | 0 | cluster\_plot | 8 |
| 2544 | GO:0032196 | transposition | biological\_process | 0.0 | 1.0 | 1.0 | 0 | cluster\_plot | 1 |
| 2545 | GO:0016531 | copper chaperone activity | molecular\_function | 0.0 | 1.0 | 1.0 | 0 | cluster\_plot | 2 |
| 2546 | GO:0016702 | oxidoreductase activity, acting on single donors with incorporation of molecular oxygen, incorporation of two atoms of oxygen | molecular\_function | 0.0 | 1.0 | 1.0 | 0 | cluster\_plot | 43 |
| 2547 | GO:0009141 | nucleoside triphosphate metabolic process | biological\_process | 0.0 | 1.0 | 1.0 | 0 | cluster\_plot | 87 |
| 2548 | GO:0019773 | proteasome core complex, alpha-subunit complex | cellular\_component | 0.0 | 1.0 | 1.0 | 0 | cluster\_plot | 16 |
| 2549 | GO:0009200 | deoxyribonucleoside triphosphate metabolic process | biological\_process | 0.0 | 1.0 | 1.0 | 0 | cluster\_plot | 1 |
| 2550 | GO:0070469 | respiratory chain | cellular\_component | 0.0 | 1.0 | 1.0 | 0 | cluster\_plot | 3 |
| 2551 | GO:0003697 | single-stranded DNA binding | molecular\_function | 0.0 | 1.0 | 1.0 | 0 | cluster\_plot | 9 |
| 2552 | GO:0007015 | actin filament organization | biological\_process | 0.0 | 1.0 | 1.0 | 0 | cluster\_plot | 4 |
| 2553 | GO:0042454 | ribonucleoside catabolic process | biological\_process | 0.0 | 1.0 | 1.0 | 0 | cluster\_plot | 41 |
| 2554 | GO:0044422 | organelle part | cellular\_component | 0.0 | 1.0 | 1.0 | 0 | cluster\_plot | 532 |
| 2555 | GO:0009164 | nucleoside catabolic process | biological\_process | 0.0 | 1.0 | 1.0 | 0 | cluster\_plot | 41 |
| 2556 | GO:0017182 | peptidyl-diphthamide metabolic process | biological\_process | 0.0 | 1.0 | 1.0 | 0 | cluster\_plot | 4 |
| 2557 | GO:0006026 | aminoglycan catabolic process | biological\_process | 0.0 | 1.0 | 1.0 | 0 | cluster\_plot | 18 |
| 2558 | GO:0048518 | positive regulation of biological process | biological\_process | 0.0 | 1.0 | 1.0 | 0 | cluster\_plot | 12 |
| 2559 | GO:0032956 | regulation of actin cytoskeleton organization | biological\_process | 0.0 | 1.0 | 1.0 | 0 | cluster\_plot | 10 |
| 2560 | GO:1902099 | regulation of metaphase/anaphase transition of cell cycle | biological\_process | 0.0 | 1.0 | 1.0 | 0 | cluster\_plot | 5 |
| 2561 | GO:0004751 | ribose-5-phosphate isomerase activity | molecular\_function | 0.0 | 1.0 | 1.0 | 0 | cluster\_plot | 4 |
| 2562 | GO:0044429 | mitochondrial part | cellular\_component | 0.0 | 1.0 | 1.0 | 0 | cluster\_plot | 75 |
| 2563 | GO:0009228 | thiamine biosynthetic process | biological\_process | 0.0 | 1.0 | 1.0 | 0 | cluster\_plot | 8 |
| 2564 | GO:0042132 | fructose 1,6-bisphosphate 1-phosphatase activity | molecular\_function | 0.0 | 1.0 | 1.0 | 0 | cluster\_plot | 11 |
| 2565 | GO:0006613 | cotranslational protein targeting to membrane | biological\_process | 0.0 | 1.0 | 1.0 | 0 | cluster\_plot | 17 |
| 2566 | GO:0042277 | peptide binding | molecular\_function | 0.0 | 1.0 | 1.0 | 0 | cluster\_plot | 12 |
| 2567 | GO:0015267 | channel activity | molecular\_function | 0.0 | 1.0 | 1.0 | 0 | cluster\_plot | 76 |
| 2568 | GO:0016855 | racemase and epimerase activity, acting on amino acids and derivatives | molecular\_function | 0.0 | 1.0 | 1.0 | 0 | cluster\_plot | 5 |
| 2569 | GO:0003999 | adenine phosphoribosyltransferase activity | molecular\_function | 0.0 | 1.0 | 1.0 | 0 | cluster\_plot | 8 |
| 2570 | GO:0051383 | kinetochore organization | biological\_process | 0.0 | 1.0 | 1.0 | 0 | cluster\_plot | 1 |
| 2571 | GO:0004413 | homoserine kinase activity | molecular\_function | 0.0 | 1.0 | 1.0 | 0 | cluster\_plot | 3 |
| 2572 | GO:0006412 | translation | biological\_process | 0.0 | 1.0 | 1.0 | 0 | cluster\_plot | 439 |
| 2573 | GO:0006760 | folic acid-containing compound metabolic process | biological\_process | 0.0 | 1.0 | 1.0 | 0 | cluster\_plot | 24 |
| 2574 | GO:0030604 | 1-deoxy-D-xylulose-5-phosphate reductoisomerase activity | molecular\_function | 0.0 | 1.0 | 1.0 | 0 | cluster\_plot | 1 |
| 2575 | GO:0004076 | biotin synthase activity | molecular\_function | 0.0 | 1.0 | 1.0 | 0 | cluster\_plot | 2 |
| 2576 | GO:0006457 | protein folding | biological\_process | 0.0 | 1.0 | 1.0 | 0 | cluster\_plot | 169 |
| 2577 | GO:0072527 | pyrimidine-containing compound metabolic process | biological\_process | 0.0 | 1.0 | 1.0 | 0 | cluster\_plot | 40 |
| 2578 | GO:0005199 | structural constituent of cell wall | molecular\_function | 0.0 | 1.0 | 1.0 | 0 | cluster\_plot | 1 |
| 2579 | GO:0044265 | cellular macromolecule catabolic process | biological\_process | 0.0 | 1.0 | 1.0 | 0 | cluster\_plot | 167 |
| 2580 | GO:1902223 | erythrose 4-phosphate/phosphoenolpyruvate family amino acid biosynthetic process | biological\_process | 0.0 | 1.0 | 1.0 | 0 | cluster\_plot | 8 |
| 2581 | GO:0009062 | fatty acid catabolic process | biological\_process | 0.0 | 1.0 | 1.0 | 0 | cluster\_plot | 7 |
| 2582 | GO:0004506 | squalene monooxygenase activity | molecular\_function | 0.0 | 1.0 | 1.0 | 0 | cluster\_plot | 16 |
| 2583 | GO:0004084 | branched-chain-amino-acid transaminase activity | molecular\_function | 0.0 | 1.0 | 1.0 | 0 | cluster\_plot | 9 |
| 2584 | GO:0006213 | pyrimidine nucleoside metabolic process | biological\_process | 0.0 | 1.0 | 1.0 | 0 | cluster\_plot | 12 |
| 2585 | GO:0022402 | cell cycle process | biological\_process | 0.0 | 1.0 | 1.0 | 0 | cluster\_plot | 43 |
| 2586 | GO:0003991 | acetylglutamate kinase activity | molecular\_function | 0.0 | 1.0 | 1.0 | 0 | cluster\_plot | 1 |
| 2587 | GO:0071824 | protein-DNA complex subunit organization | biological\_process | 0.0 | 1.0 | 1.0 | 0 | cluster\_plot | 32 |
| 2588 | GO:0016801 | hydrolase activity, acting on ether bonds | molecular\_function | 0.0 | 1.0 | 1.0 | 0 | cluster\_plot | 2 |
| 2589 | GO:0004615 | phosphomannomutase activity | molecular\_function | 0.0 | 1.0 | 1.0 | 0 | cluster\_plot | 1 |
| 2590 | GO:0006269 | DNA replication, synthesis of RNA primer | biological\_process | 0.0 | 1.0 | 1.0 | 0 | cluster\_plot | 3 |
| 2591 | GO:0009081 | branched-chain amino acid metabolic process | biological\_process | 0.0 | 1.0 | 1.0 | 0 | cluster\_plot | 26 |
| 2592 | GO:0004332 | fructose-bisphosphate aldolase activity | molecular\_function | 0.0 | 1.0 | 1.0 | 0 | cluster\_plot | 11 |
| 2593 | GO:0004642 | phosphoribosylformylglycinamidine synthase activity | molecular\_function | 0.0 | 1.0 | 1.0 | 0 | cluster\_plot | 4 |
| 2594 | GO:0017038 | protein import | biological\_process | 0.0 | 1.0 | 1.0 | 0 | cluster\_plot | 30 |
| 2595 | GO:0008276 | protein methyltransferase activity | molecular\_function | 0.0 | 1.0 | 1.0 | 0 | cluster\_plot | 28 |
| 2596 | GO:0045145 | single-stranded DNA 5'-3' exodeoxyribonuclease activity | molecular\_function | 0.0 | 1.0 | 1.0 | 0 | cluster\_plot | 2 |
| 2597 | GO:0006546 | glycine catabolic process | biological\_process | 0.0 | 1.0 | 1.0 | 0 | cluster\_plot | 14 |
| 2598 | GO:0016278 | lysine N-methyltransferase activity | molecular\_function | 0.0 | 1.0 | 1.0 | 0 | cluster\_plot | 18 |
| 2599 | GO:0045254 | pyruvate dehydrogenase complex | cellular\_component | 0.0 | 1.0 | 1.0 | 0 | cluster\_plot | 1 |
| 2600 | GO:0000275 | mitochondrial proton-transporting ATP synthase complex, catalytic core F(1) | cellular\_component | 0.0 | 1.0 | 1.0 | 0 | cluster\_plot | 4 |
| 2601 | GO:0045900 | negative regulation of translational elongation | biological\_process | 0.0 | 1.0 | 1.0 | 0 | cluster\_plot | 2 |
| 2602 | GO:0046395 | carboxylic acid catabolic process | biological\_process | 0.0 | 1.0 | 1.0 | 0 | cluster\_plot | 37 |
| 2603 | GO:0016418 | S-acetyltransferase activity | molecular\_function | 0.0 | 1.0 | 1.0 | 0 | cluster\_plot | 1 |
| 2604 | GO:0051606 | detection of stimulus | biological\_process | 0.0 | 1.0 | 1.0 | 0 | cluster\_plot | 8 |
| 2605 | GO:0043174 | nucleoside salvage | biological\_process | 0.0 | 1.0 | 1.0 | 0 | cluster\_plot | 2 |
| 2606 | GO:0015927 | trehalase activity | molecular\_function | 0.0 | 1.0 | 1.0 | 0 | cluster\_plot | 2 |
| 2607 | GO:0006528 | asparagine metabolic process | biological\_process | 0.0 | 1.0 | 1.0 | 0 | cluster\_plot | 10 |
| 2608 | GO:0019358 | nicotinate nucleotide salvage | biological\_process | 0.0 | 1.0 | 1.0 | 0 | cluster\_plot | 2 |
| 2609 | GO:0033170 | protein-DNA loading ATPase activity | molecular\_function | 0.0 | 1.0 | 1.0 | 0 | cluster\_plot | 5 |
| 2610 | GO:0042278 | purine nucleoside metabolic process | biological\_process | 0.0 | 1.0 | 1.0 | 0 | cluster\_plot | 112 |
| 2611 | GO:0009512 | cytochrome b6f complex | cellular\_component | 0.0 | 1.0 | 1.0 | 0 | cluster\_plot | 1 |
| 2612 | GO:0004831 | tyrosine-tRNA ligase activity | molecular\_function | 0.0 | 1.0 | 1.0 | 0 | cluster\_plot | 2 |
| 2613 | GO:0071267 | L-methionine salvage | biological\_process | 0.0 | 1.0 | 1.0 | 0 | cluster\_plot | 2 |
| 2614 | GO:0033865 | nucleoside bisphosphate metabolic process | biological\_process | 0.0 | 1.0 | 1.0 | 0 | cluster\_plot | 12 |
| 2615 | GO:0019725 | cellular homeostasis | biological\_process | 0.0 | 1.0 | 1.0 | 0 | cluster\_plot | 161 |
| 2616 | GO:0042579 | microbody | cellular\_component | 0.0 | 1.0 | 1.0 | 0 | cluster\_plot | 8 |
| 2617 | GO:0031577 | spindle checkpoint | biological\_process | 0.0 | 1.0 | 1.0 | 0 | cluster\_plot | 2 |
| 2618 | GO:0004347 | glucose-6-phosphate isomerase activity | molecular\_function | 0.0 | 1.0 | 1.0 | 0 | cluster\_plot | 4 |
| 2619 | GO:0008202 | steroid metabolic process | biological\_process | 0.0 | 1.0 | 1.0 | 0 | cluster\_plot | 22 |
| 2620 | GO:0009084 | glutamine family amino acid biosynthetic process | biological\_process | 0.0 | 1.0 | 1.0 | 0 | cluster\_plot | 36 |
| 2621 | GO:0003937 | IMP cyclohydrolase activity | molecular\_function | 0.0 | 1.0 | 1.0 | 0 | cluster\_plot | 7 |
| 2622 | GO:0032312 | regulation of ARF GTPase activity | biological\_process | 0.0 | 1.0 | 1.0 | 0 | cluster\_plot | 28 |
| 2623 | GO:0055076 | transition metal ion homeostasis | biological\_process | 0.0 | 1.0 | 1.0 | 0 | cluster\_plot | 6 |
| 2624 | GO:0051170 | nuclear import | biological\_process | 0.0 | 1.0 | 1.0 | 0 | cluster\_plot | 9 |
| 2625 | GO:0009073 | aromatic amino acid family biosynthetic process | biological\_process | 0.0 | 1.0 | 1.0 | 0 | cluster\_plot | 35 |
| 2626 | GO:0006298 | mismatch repair | biological\_process | 0.0 | 1.0 | 1.0 | 0 | cluster\_plot | 20 |
| 2627 | GO:0000302 | response to reactive oxygen species | biological\_process | 0.0 | 1.0 | 1.0 | 0 | cluster\_plot | 1 |
| 2628 | GO:0072511 | divalent inorganic cation transport | biological\_process | 0.0 | 1.0 | 1.0 | 0 | cluster\_plot | 59 |
| 2629 | GO:0090529 | cell septum assembly | biological\_process | 0.0 | 1.0 | 1.0 | 0 | cluster\_plot | 3 |
| 2630 | GO:0030258 | lipid modification | biological\_process | 0.0 | 1.0 | 1.0 | 0 | cluster\_plot | 36 |
| 2631 | GO:0042085 | 5-methyltetrahydropteroyltri-L-glutamate-dependent methyltransferase activity | molecular\_function | 0.0 | 1.0 | 1.0 | 0 | cluster\_plot | 3 |
| 2632 | GO:0009892 | negative regulation of metabolic process | biological\_process | 0.0 | 1.0 | 1.0 | 0 | cluster\_plot | 34 |
| 2633 | GO:0008889 | glycerophosphodiester phosphodiesterase activity | molecular\_function | 0.0 | 1.0 | 1.0 | 0 | cluster\_plot | 16 |
| 2634 | GO:0055072 | iron ion homeostasis | biological\_process | 0.0 | 1.0 | 1.0 | 0 | cluster\_plot | 6 |
| 2635 | GO:0031072 | heat shock protein binding | molecular\_function | 0.0 | 1.0 | 1.0 | 0 | cluster\_plot | 8 |
| 2636 | GO:0017006 | protein-tetrapyrrole linkage | biological\_process | 0.0 | 1.0 | 1.0 | 0 | cluster\_plot | 3 |
| 2637 | GO:0034227 | tRNA thio-modification | biological\_process | 0.0 | 1.0 | 1.0 | 0 | cluster\_plot | 1 |
| 2638 | GO:0006435 | threonyl-tRNA aminoacylation | biological\_process | 0.0 | 1.0 | 1.0 | 0 | cluster\_plot | 3 |
| 2639 | GO:0008716 | D-alanine-D-alanine ligase activity | molecular\_function | 0.0 | 1.0 | 1.0 | 0 | cluster\_plot | 1 |
| 2640 | GO:0000213 | tRNA-intron endonuclease activity | molecular\_function | 0.0 | 1.0 | 1.0 | 0 | cluster\_plot | 1 |
| 2641 | GO:0005096 | GTPase activator activity | molecular\_function | 0.0 | 1.0 | 1.0 | 0 | cluster\_plot | 55 |
| 2642 | GO:0006950 | response to stress | biological\_process | 0.0 | 1.0 | 1.0 | 0 | cluster\_plot | 503 |
| 2643 | GO:0016624 | oxidoreductase activity, acting on the aldehyde or oxo group of donors, disulfide as acceptor | molecular\_function | 0.0 | 1.0 | 1.0 | 0 | cluster\_plot | 9 |
| 2644 | GO:0003919 | FMN adenylyltransferase activity | molecular\_function | 0.0 | 1.0 | 1.0 | 0 | cluster\_plot | 1 |
| 2645 | GO:0038023 | signaling receptor activity | molecular\_function | 0.0 | 1.0 | 1.0 | 0 | cluster\_plot | 66 |
| 2646 | GO:0019220 | regulation of phosphate metabolic process | biological\_process | 0.0 | 1.0 | 1.0 | 0 | cluster\_plot | 66 |
| 2647 | GO:0008297 | single-stranded DNA exodeoxyribonuclease activity | molecular\_function | 0.0 | 1.0 | 1.0 | 0 | cluster\_plot | 2 |
| 2648 | GO:0009060 | aerobic respiration | biological\_process | 0.0 | 1.0 | 1.0 | 0 | cluster\_plot | 1 |
| 2649 | GO:0045333 | cellular respiration | biological\_process | 0.0 | 1.0 | 1.0 | 0 | cluster\_plot | 1 |
| 2650 | GO:0045905 | positive regulation of translational termination | biological\_process | 0.0 | 1.0 | 1.0 | 0 | cluster\_plot | 3 |
| 2651 | GO:0016731 | oxidoreductase activity, acting on iron-sulfur proteins as donors, NAD or NADP as acceptor | molecular\_function | 0.0 | 1.0 | 1.0 | 0 | cluster\_plot | 1 |
| 2652 | GO:0042430 | indole-containing compound metabolic process | biological\_process | 0.0 | 1.0 | 1.0 | 0 | cluster\_plot | 16 |
| 2653 | GO:0016109 | tetraterpenoid biosynthetic process | biological\_process | 0.0 | 1.0 | 1.0 | 0 | cluster\_plot | 9 |
| 2654 | GO:0005741 | mitochondrial outer membrane | cellular\_component | 0.0 | 1.0 | 1.0 | 0 | cluster\_plot | 11 |
| 2655 | GO:0031515 | tRNA (m1A) methyltransferase complex | cellular\_component | 0.0 | 1.0 | 1.0 | 0 | cluster\_plot | 3 |
| 2656 | GO:0019674 | NAD metabolic process | biological\_process | 0.0 | 1.0 | 1.0 | 0 | cluster\_plot | 10 |
| 2657 | GO:0017176 | phosphatidylinositol N-acetylglucosaminyltransferase activity | molecular\_function | 0.0 | 1.0 | 1.0 | 0 | cluster\_plot | 7 |
| 2658 | GO:0004594 | pantothenate kinase activity | molecular\_function | 0.0 | 1.0 | 1.0 | 0 | cluster\_plot | 1 |
| 2659 | GO:0090150 | establishment of protein localization to membrane | biological\_process | 0.0 | 1.0 | 1.0 | 0 | cluster\_plot | 34 |
| 2660 | GO:0006566 | threonine metabolic process | biological\_process | 0.0 | 1.0 | 1.0 | 0 | cluster\_plot | 4 |
| 2661 | GO:0030131 | clathrin adaptor complex | cellular\_component | 0.0 | 1.0 | 1.0 | 0 | cluster\_plot | 21 |
| 2662 | GO:0004042 | acetyl-CoA:L-glutamate N-acetyltransferase activity | molecular\_function | 0.0 | 1.0 | 1.0 | 0 | cluster\_plot | 2 |
| 2663 | GO:0022613 | ribonucleoprotein complex biogenesis | biological\_process | 0.0 | 1.0 | 1.0 | 0 | cluster\_plot | 25 |
| 2664 | GO:0009582 | detection of abiotic stimulus | biological\_process | 0.0 | 1.0 | 1.0 | 0 | cluster\_plot | 8 |
| 2665 | GO:0042597 | periplasmic space | cellular\_component | 0.0 | 1.0 | 1.0 | 0 | cluster\_plot | 2 |
| 2666 | GO:0008836 | diaminopimelate decarboxylase activity | molecular\_function | 0.0 | 1.0 | 1.0 | 0 | cluster\_plot | 1 |
| 2667 | GO:0051382 | kinetochore assembly | biological\_process | 0.0 | 1.0 | 1.0 | 0 | cluster\_plot | 1 |
| 2668 | GO:0051254 | positive regulation of RNA metabolic process | biological\_process | 0.0 | 1.0 | 1.0 | 0 | cluster\_plot | 4 |
| 2669 | GO:0016638 | oxidoreductase activity, acting on the CH-NH2 group of donors | molecular\_function | 0.0 | 1.0 | 1.0 | 0 | cluster\_plot | 25 |
| 2670 | GO:0005254 | chloride channel activity | molecular\_function | 0.0 | 1.0 | 1.0 | 0 | cluster\_plot | 13 |
| 2671 | GO:0048585 | negative regulation of response to stimulus | biological\_process | 0.0 | 1.0 | 1.0 | 0 | cluster\_plot | 2 |
| 2672 | GO:0000278 | mitotic cell cycle | biological\_process | 0.0 | 1.0 | 1.0 | 0 | cluster\_plot | 3 |
| 2673 | GO:0005960 | glycine cleavage complex | cellular\_component | 0.0 | 1.0 | 1.0 | 0 | cluster\_plot | 7 |
| 2674 | GO:0002097 | tRNA wobble base modification | biological\_process | 0.0 | 1.0 | 1.0 | 0 | cluster\_plot | 1 |
| 2675 | GO:0009236 | cobalamin biosynthetic process | biological\_process | 0.0 | 1.0 | 1.0 | 0 | cluster\_plot | 1 |
| 2676 | GO:0046164 | alcohol catabolic process | biological\_process | 0.0 | 1.0 | 1.0 | 0 | cluster\_plot | 9 |
| 2677 | GO:0046165 | alcohol biosynthetic process | biological\_process | 0.0 | 1.0 | 1.0 | 0 | cluster\_plot | 9 |
| 2678 | GO:0015154 | disaccharide transmembrane transporter activity | molecular\_function | 0.0 | 1.0 | 1.0 | 0 | cluster\_plot | 6 |
| 2679 | GO:0043021 | ribonucleoprotein complex binding | molecular\_function | 0.0 | 1.0 | 1.0 | 0 | cluster\_plot | 9 |
| 2680 | GO:0043605 | cellular amide catabolic process | biological\_process | 0.0 | 1.0 | 1.0 | 0 | cluster\_plot | 3 |
| 2681 | GO:0031625 | ubiquitin protein ligase binding | molecular\_function | 0.0 | 1.0 | 1.0 | 0 | cluster\_plot | 19 |
| 2682 | GO:0004420 | hydroxymethylglutaryl-CoA reductase (NADPH) activity | molecular\_function | 0.0 | 1.0 | 1.0 | 0 | cluster\_plot | 5 |
| 2683 | GO:0046168 | glycerol-3-phosphate catabolic process | biological\_process | 0.0 | 1.0 | 1.0 | 0 | cluster\_plot | 9 |
| 2684 | GO:0022904 | respiratory electron transport chain | biological\_process | 0.0 | 1.0 | 1.0 | 0 | cluster\_plot | 11 |
| 2685 | GO:0016774 | phosphotransferase activity, carboxyl group as acceptor | molecular\_function | 0.0 | 1.0 | 1.0 | 0 | cluster\_plot | 6 |
| 2686 | GO:0004411 | homogentisate 1,2-dioxygenase activity | molecular\_function | 0.0 | 1.0 | 1.0 | 0 | cluster\_plot | 1 |
| 2687 | GO:0032993 | protein-DNA complex | cellular\_component | 0.0 | 1.0 | 1.0 | 0 | cluster\_plot | 34 |
| 2688 | GO:0043161 | proteasome-mediated ubiquitin-dependent protein catabolic process | biological\_process | 0.0 | 1.0 | 1.0 | 0 | cluster\_plot | 8 |
| 2689 | GO:0004222 | metalloendopeptidase activity | molecular\_function | 0.0 | 1.0 | 1.0 | 0 | cluster\_plot | 51 |
| 2690 | GO:0032271 | regulation of protein polymerization | biological\_process | 0.0 | 1.0 | 1.0 | 0 | cluster\_plot | 10 |
| 2691 | GO:0006662 | glycerol ether metabolic process | biological\_process | 0.0 | 1.0 | 1.0 | 0 | cluster\_plot | 7 |
| 2692 | GO:0031669 | cellular response to nutrient levels | biological\_process | 0.0 | 1.0 | 1.0 | 0 | cluster\_plot | 2 |
| 2693 | GO:0044085 | cellular component biogenesis | biological\_process | 0.0 | 1.0 | 1.0 | 0 | cluster\_plot | 27 |
| 2694 | GO:1901617 | organic hydroxy compound biosynthetic process | biological\_process | 0.0 | 1.0 | 1.0 | 0 | cluster\_plot | 17 |
| 2695 | GO:0006423 | cysteinyl-tRNA aminoacylation | biological\_process | 0.0 | 1.0 | 1.0 | 0 | cluster\_plot | 4 |
| 2696 | GO:0008661 | 1-deoxy-D-xylulose-5-phosphate synthase activity | molecular\_function | 0.0 | 1.0 | 1.0 | 0 | cluster\_plot | 5 |
| 2697 | GO:0006721 | terpenoid metabolic process | biological\_process | 0.0 | 1.0 | 1.0 | 0 | cluster\_plot | 19 |
| 2698 | GO:0015931 | nucleobase-containing compound transport | biological\_process | 0.0 | 1.0 | 1.0 | 0 | cluster\_plot | 9 |
| 2699 | GO:0006351 | transcription, DNA-templated | biological\_process | 0.0 | 1.0 | 1.0 | 0 | cluster\_plot | 143 |
| 2700 | GO:0004797 | thymidine kinase activity | molecular\_function | 0.0 | 1.0 | 1.0 | 0 | cluster\_plot | 6 |
| 2701 | GO:0090066 | regulation of anatomical structure size | biological\_process | 0.0 | 1.0 | 1.0 | 0 | cluster\_plot | 10 |
| 2702 | GO:0045275 | respiratory chain complex III | cellular\_component | 0.0 | 1.0 | 1.0 | 0 | cluster\_plot | 5 |
| 2703 | GO:0031331 | positive regulation of cellular catabolic process | biological\_process | 0.0 | 1.0 | 1.0 | 0 | cluster\_plot | 1 |
| 2704 | GO:0009110 | vitamin biosynthetic process | biological\_process | 0.0 | 1.0 | 1.0 | 0 | cluster\_plot | 32 |
| 2705 | GO:0003896 | DNA primase activity | molecular\_function | 0.0 | 1.0 | 1.0 | 0 | cluster\_plot | 4 |
| 2706 | GO:0006562 | proline catabolic process | biological\_process | 0.0 | 1.0 | 1.0 | 0 | cluster\_plot | 4 |
| 2707 | GO:1901271 | lipooligosaccharide biosynthetic process | biological\_process | 0.0 | 1.0 | 1.0 | 0 | cluster\_plot | 3 |
| 2708 | GO:0016628 | oxidoreductase activity, acting on the CH-CH group of donors, NAD or NADP as acceptor | molecular\_function | 0.0 | 1.0 | 1.0 | 0 | cluster\_plot | 10 |
| 2709 | GO:0010639 | negative regulation of organelle organization | biological\_process | 0.0 | 1.0 | 1.0 | 0 | cluster\_plot | 2 |
| 2710 | GO:0007346 | regulation of mitotic cell cycle | biological\_process | 0.0 | 1.0 | 1.0 | 0 | cluster\_plot | 5 |
| 2711 | GO:0016830 | carbon-carbon lyase activity | molecular\_function | 0.0 | 1.0 | 1.0 | 0 | cluster\_plot | 89 |
| 2712 | GO:0030653 | beta-lactam antibiotic metabolic process | biological\_process | 0.0 | 1.0 | 1.0 | 0 | cluster\_plot | 1 |
| 2713 | GO:0015450 | P-P-bond-hydrolysis-driven protein transmembrane transporter activity | molecular\_function | 0.0 | 1.0 | 1.0 | 0 | cluster\_plot | 7 |
| 2714 | GO:0008690 | 3-deoxy-manno-octulosonate cytidylyltransferase activity | molecular\_function | 0.0 | 1.0 | 1.0 | 0 | cluster\_plot | 2 |
| 2715 | GO:0046084 | adenine biosynthetic process | biological\_process | 0.0 | 1.0 | 1.0 | 0 | cluster\_plot | 8 |
| 2716 | GO:0042549 | photosystem II stabilization | biological\_process | 0.0 | 1.0 | 1.0 | 0 | cluster\_plot | 4 |
| 2717 | GO:0009112 | nucleobase metabolic process | biological\_process | 0.0 | 1.0 | 1.0 | 0 | cluster\_plot | 20 |
| 2718 | GO:0018904 | ether metabolic process | biological\_process | 0.0 | 1.0 | 1.0 | 0 | cluster\_plot | 7 |
| 2719 | GO:0010309 | acireductone dioxygenase [iron(II)-requiring] activity | molecular\_function | 0.0 | 1.0 | 1.0 | 0 | cluster\_plot | 4 |
| 2720 | GO:0005978 | glycogen biosynthetic process | biological\_process | 0.0 | 1.0 | 1.0 | 0 | cluster\_plot | 3 |
| 2721 | GO:0034622 | cellular macromolecular complex assembly | biological\_process | 0.0 | 1.0 | 1.0 | 0 | cluster\_plot | 64 |
| 2722 | GO:0046467 | membrane lipid biosynthetic process | biological\_process | 0.0 | 1.0 | 1.0 | 0 | cluster\_plot | 26 |
| 2723 | GO:0022406 | membrane docking | biological\_process | 0.0 | 1.0 | 1.0 | 0 | cluster\_plot | 17 |
| 2724 | GO:0008897 | holo-[acyl-carrier-protein] synthase activity | molecular\_function | 0.0 | 1.0 | 1.0 | 0 | cluster\_plot | 7 |
| 2725 | GO:0001104 | RNA polymerase II transcription cofactor activity | molecular\_function | 0.0 | 1.0 | 1.0 | 0 | cluster\_plot | 21 |
| 2726 | GO:0004568 | chitinase activity | molecular\_function | 0.0 | 1.0 | 1.0 | 0 | cluster\_plot | 18 |
| 2727 | GO:0009295 | nucleoid | cellular\_component | 0.0 | 1.0 | 1.0 | 0 | cluster\_plot | 1 |
| 2728 | GO:0004175 | endopeptidase activity | molecular\_function | 0.0 | 1.0 | 1.0 | 0 | cluster\_plot | 281 |
| 2729 | GO:0045893 | positive regulation of transcription, DNA-templated | biological\_process | 0.0 | 1.0 | 1.0 | 0 | cluster\_plot | 4 |
| 2730 | GO:0008121 | ubiquinol-cytochrome-c reductase activity | molecular\_function | 0.0 | 1.0 | 1.0 | 0 | cluster\_plot | 3 |
| 2731 | GO:2001070 | starch binding | molecular\_function | 0.0 | 1.0 | 1.0 | 0 | cluster\_plot | 10 |
| 2732 | GO:0016987 | sigma factor activity | molecular\_function | 0.0 | 1.0 | 1.0 | 0 | cluster\_plot | 12 |
| 2733 | GO:0015125 | bile acid transmembrane transporter activity | molecular\_function | 0.0 | 1.0 | 1.0 | 0 | cluster\_plot | 11 |
| 2734 | GO:0042592 | homeostatic process | biological\_process | 0.0 | 1.0 | 1.0 | 0 | cluster\_plot | 169 |
| 2735 | GO:0015108 | chloride transmembrane transporter activity | molecular\_function | 0.0 | 1.0 | 1.0 | 0 | cluster\_plot | 15 |
| 2736 | GO:0042548 | regulation of photosynthesis, light reaction | biological\_process | 0.0 | 1.0 | 1.0 | 0 | cluster\_plot | 4 |
| 2737 | GO:0006183 | GTP biosynthetic process | biological\_process | 0.0 | 1.0 | 1.0 | 0 | cluster\_plot | 7 |
| 2738 | GO:0015035 | protein disulfide oxidoreductase activity | molecular\_function | 0.0 | 1.0 | 1.0 | 0 | cluster\_plot | 75 |
| 2739 | GO:0003918 | DNA topoisomerase type II (ATP-hydrolyzing) activity | molecular\_function | 0.0 | 1.0 | 1.0 | 0 | cluster\_plot | 11 |
| 2740 | GO:0022610 | biological adhesion | biological\_process | 0.0 | 1.0 | 1.0 | 0 | cluster\_plot | 4 |
| 2741 | GO:0046036 | CTP metabolic process | biological\_process | 0.0 | 1.0 | 1.0 | 0 | cluster\_plot | 7 |
| 2742 | GO:0031369 | translation initiation factor binding | molecular\_function | 0.0 | 1.0 | 1.0 | 0 | cluster\_plot | 4 |
| 2743 | GO:0016128 | phytosteroid metabolic process | biological\_process | 0.0 | 1.0 | 1.0 | 0 | cluster\_plot | 3 |
| 2744 | GO:0015385 | sodium:proton antiporter activity | molecular\_function | 0.0 | 1.0 | 1.0 | 0 | cluster\_plot | 5 |
| 2745 | GO:0016633 | galactonolactone dehydrogenase activity | molecular\_function | 0.0 | 1.0 | 1.0 | 0 | cluster\_plot | 2 |
| 2746 | GO:0000123 | histone acetyltransferase complex | cellular\_component | 0.0 | 1.0 | 1.0 | 0 | cluster\_plot | 14 |
| 2747 | GO:0006547 | histidine metabolic process | biological\_process | 0.0 | 1.0 | 1.0 | 0 | cluster\_plot | 17 |
| 2748 | GO:0006006 | glucose metabolic process | biological\_process | 0.0 | 1.0 | 1.0 | 0 | cluster\_plot | 41 |
| 2749 | GO:0033178 | proton-transporting two-sector ATPase complex, catalytic domain | cellular\_component | 0.0 | 1.0 | 1.0 | 0 | cluster\_plot | 31 |
| 2750 | GO:0042765 | GPI-anchor transamidase complex | cellular\_component | 0.0 | 1.0 | 1.0 | 0 | cluster\_plot | 9 |
| 2751 | GO:0016840 | carbon-nitrogen lyase activity | molecular\_function | 0.0 | 1.0 | 1.0 | 0 | cluster\_plot | 24 |
| 2752 | GO:0032065 | cortical protein anchoring | biological\_process | 0.0 | 1.0 | 1.0 | 0 | cluster\_plot | 2 |
| 2753 | GO:0004649 | poly(ADP-ribose) glycohydrolase activity | molecular\_function | 0.0 | 1.0 | 1.0 | 0 | cluster\_plot | 2 |
| 2754 | GO:0046379 | extracellular polysaccharide metabolic process | biological\_process | 0.0 | 1.0 | 1.0 | 0 | cluster\_plot | 7 |
| 2755 | GO:0032465 | regulation of cytokinesis | biological\_process | 0.0 | 1.0 | 1.0 | 0 | cluster\_plot | 1 |
| 2756 | GO:0019693 | ribose phosphate metabolic process | biological\_process | 0.0 | 1.0 | 1.0 | 0 | cluster\_plot | 122 |
| 2757 | GO:0003861 | 3-isopropylmalate dehydratase activity | molecular\_function | 0.0 | 1.0 | 1.0 | 0 | cluster\_plot | 1 |
| 2758 | GO:0006430 | lysyl-tRNA aminoacylation | biological\_process | 0.0 | 1.0 | 1.0 | 0 | cluster\_plot | 3 |
| 2759 | GO:0004013 | adenosylhomocysteinase activity | molecular\_function | 0.0 | 1.0 | 1.0 | 0 | cluster\_plot | 2 |
| 2760 | GO:0008156 | negative regulation of DNA replication | biological\_process | 0.0 | 1.0 | 1.0 | 0 | cluster\_plot | 2 |
| 2761 | GO:0016999 | antibiotic metabolic process | biological\_process | 0.0 | 1.0 | 1.0 | 0 | cluster\_plot | 1 |
| 2762 | GO:0009106 | lipoate metabolic process | biological\_process | 0.0 | 1.0 | 1.0 | 0 | cluster\_plot | 7 |
| 2763 | GO:0008972 | phosphomethylpyrimidine kinase activity | molecular\_function | 0.0 | 1.0 | 1.0 | 0 | cluster\_plot | 1 |
| 2764 | GO:0005343 | organic acid:sodium symporter activity | molecular\_function | 0.0 | 1.0 | 1.0 | 0 | cluster\_plot | 11 |
| 2765 | GO:0000272 | polysaccharide catabolic process | biological\_process | 0.0 | 1.0 | 1.0 | 0 | cluster\_plot | 34 |
| 2766 | GO:0004046 | aminoacylase activity | molecular\_function | 0.0 | 1.0 | 1.0 | 0 | cluster\_plot | 3 |
| 2767 | GO:0006537 | glutamate biosynthetic process | biological\_process | 0.0 | 1.0 | 1.0 | 0 | cluster\_plot | 8 |
| 2768 | GO:0000045 | autophagic vacuole assembly | biological\_process | 0.0 | 1.0 | 1.0 | 0 | cluster\_plot | 1 |
| 2769 | GO:0061134 | peptidase regulator activity | molecular\_function | 0.0 | 1.0 | 1.0 | 0 | cluster\_plot | 22 |
| 2770 | GO:0030410 | nicotianamine synthase activity | molecular\_function | 0.0 | 1.0 | 1.0 | 0 | cluster\_plot | 2 |
| 2771 | GO:0004815 | aspartate-tRNA ligase activity | molecular\_function | 0.0 | 1.0 | 1.0 | 0 | cluster\_plot | 1 |
| 2772 | GO:0051220 | cytoplasmic sequestering of protein | biological\_process | 0.0 | 1.0 | 1.0 | 0 | cluster\_plot | 2 |
| 2773 | GO:0016125 | sterol metabolic process | biological\_process | 0.0 | 1.0 | 1.0 | 0 | cluster\_plot | 5 |
| 2774 | GO:0006044 | N-acetylglucosamine metabolic process | biological\_process | 0.0 | 1.0 | 1.0 | 0 | cluster\_plot | 1 |
| 2775 | GO:0043178 | alcohol binding | molecular\_function | 0.0 | 1.0 | 1.0 | 0 | cluster\_plot | 3 |
| 2776 | GO:0034968 | histone lysine methylation | biological\_process | 0.0 | 1.0 | 1.0 | 0 | cluster\_plot | 16 |
| 2777 | GO:0016899 | oxidoreductase activity, acting on the CH-OH group of donors, oxygen as acceptor | molecular\_function | 0.0 | 1.0 | 1.0 | 0 | cluster\_plot | 5 |
| 2778 | GO:0005850 | eukaryotic translation initiation factor 2 complex | cellular\_component | 0.0 | 1.0 | 1.0 | 0 | cluster\_plot | 2 |
| 2779 | GO:0019825 | oxygen binding | molecular\_function | 0.0 | 1.0 | 1.0 | 0 | cluster\_plot | 2 |

---
