# Supplementary material for: The Effect of Different Pollination on the Expression of Dangshan Su Pear MicroRNA
Source: Biomed Res Int. 2017 Apr 10;2017:2794040. doi: 10.1155/2017/2794040 (PMC5402243; doi:10.1155/2017/2794040)
Supplement: Supplementary file 1 — These Supplementary Materials contained the detailed data of the primer sequences used in this study, those known and novel microRNAs, differentially expressed microRNAs, the target genes of the differentially expressed microRNAs and which corresponding GO function enrichment analysis and KEGG pathway analysis in “Dangshan su” Pear of different male parent pollination. [file 2794040.f1.zip › Supplementary Material/Supplementary Material8/kegg.enrich.html]

KEGG Pathway Enrichment Analysis


# KEGG Pathway Enrichment Analysis

---

  

Note: Total detectd: 表示检测到的所有在该pathway的基因数目，百分比为：该数目/pathway中所有的已知基因

```
1.红色外框表示上调基因，绿色外框表示下调基因，
2.鼠标悬停在基因上即可显示其基因名，差异表达情况，以及ID号
3.点击基因名或其他代谢底物和离子（图中小的空心圈）均自动连接到KEGG数据库，显示该物质的详细介绍
* 各通路内的基因or蛋白的层次聚类仅针对富集的通路，层次聚类采用欧氏距离，聚类标准采用wald法。
```

| # | KEGGid | Description | Class | SubClass | Ratio | pvalue | qvalue | diffNum | diff\_cluster | Totaldetectd(%%) | nodiff\_cluster |
| 1 | 04626 | Plant-pathogen interaction | Organismal Systems | Environmental adaptation | 11.3917966247 | 2.81411620363e-16 | 9.23030114792e-14 | 25 | cluster\_plot | 302(32.18) | cluster\_plot |
| 2 | 00450 | Selenocompound metabolism | Metabolism | Metabolism of other amino acids | 26.2119815668 | 3.51528592754e-05 | 0.00576506892116 | 4 | cluster\_plot | 21(13.33) | cluster\_plot |
| 3 | 00920 | Sulfur metabolism | Metabolism | Energy metabolism | 13.2320099256 | 7.34593712645e-05 | 0.00803155792491 | 5 | cluster\_plot | 52(8.05) | cluster\_plot |
| 4 | 04612 | Antigen processing and presentation | Organismal Systems | Immune system | 7.64516129032 | 0.000249475058538 | 0.0204569548001 | 6 | cluster\_plot | 108(19.67) | cluster\_plot |
| 5 | 00902 | Monoterpenoid biosynthesis | Metabolism | Metabolism of terpenoids and polyketides | 19.6589861751 | 0.000740203341759 | 0.0485573392194 | 3 | cluster\_plot | 21(4.88) | cluster\_plot |
| 6 | 00511 | Other glycan degradation | Metabolism | Glycan biosynthesis and metabolism | 8.09487666034 | 0.0290132069417 | 1.0 | 2 | cluster\_plot | 34(16.67) | cluster\_plot |
| 7 | 05152 | Tuberculosis | Human Diseases | Infectious diseases | 2.48698017878 | 0.0431057466676 | 1.0 | 6 | cluster\_plot | 332(13.92) | cluster\_plot |
| 8 | 00230 | Purine metabolism | Metabolism | Nucleotide metabolism | 2.68775201613 | 0.0470832460964 | 1.0 | 5 | cluster\_plot | 256(13.70) | cluster\_plot |
| 9 | 04670 | Leukocyte transendothelial migration | Organismal Systems | Immune system | 12.5102639296 | 0.0844455205791 | 1.0 | 1 | cluster\_plot | 11(1.72) | cluster\_plot |
| 10 | 00061 | Fatty acid biosynthesis | Metabolism | Lipid metabolism | 4.10784785749 | 0.0920038809485 | 1.0 | 2 | cluster\_plot | 67(5.73) | cluster\_plot |
| 11 | 05206 | MicroRNAs in cancer | Human Diseases | Cancers | 2.64640198511 | 0.183154596612 | 1.0 | 2 | cluster\_plot | 104(3.48) | cluster\_plot |
| 12 | 00780 | Biotin metabolism | Metabolism | Metabolism of cofactors and vitamins | 5.09677419355 | 0.185896217364 | 1.0 | 1 | cluster\_plot | 27(11.76) | cluster\_plot |
| 13 | 01212 | Fatty acid metabolism | Metabolism | Overview | 2.10096035459 | 0.255045508192 | 1.0 | 2 | cluster\_plot | 131(3.84) | cluster\_plot |
| 14 | 04930 | Type II diabetes mellitus | Human Diseases | Endocrine and metabolic diseases | 2.86693548387 | 0.301959789523 | 1.0 | 1 | cluster\_plot | 48(7.69) | cluster\_plot |
| 15 | 00910 | Nitrogen metabolism | Metabolism | Energy metabolism | 2.64640198511 | 0.322085023502 | 1.0 | 1 | cluster\_plot | 52(8.05) | cluster\_plot |
| 16 | 04380 | Osteoclast differentiation | Organismal Systems | Development | 2.37263626251 | 0.351173670512 | 1.0 | 1 | cluster\_plot | 58(7.08) | cluster\_plot |
| 17 | 04920 | Adipocytokine signaling pathway | Organismal Systems | Endocrine system | 2.37263626251 | 0.351173670512 | 1.0 | 1 | cluster\_plot | 58(12.70) | cluster\_plot |
| 18 | 04075 | Plant hormone signal transduction | Environmental Information Processing | Signal transduction | 1.35246096536 | 0.355525335524 | 1.0 | 4 | cluster\_plot | 407(55.71) | cluster\_plot |
| 19 | 04152 | AMPK signaling pathway | Environmental Information Processing | Signal transduction | 1.60015003751 | 0.363828464972 | 1.0 | 2 | cluster\_plot | 172(22.13) | cluster\_plot |
| 20 | 04976 | Bile secretion | Organismal Systems | Digestive system | 2.18433179724 | 0.374443868831 | 1.0 | 1 | cluster\_plot | 63(3.09) | cluster\_plot |
| 21 | 04140 | Regulation of autophagy | Cellular Processes | Transport and catabolism | 2.1502016129 | 0.378995206898 | 1.0 | 1 | cluster\_plot | 64(35.14) | cluster\_plot |
| 22 | 03440 | Homologous recombination | Genetic Information Processing | Replication and repair | 2.11712158809 | 0.383512909193 | 1.0 | 1 | cluster\_plot | 65(30.14) | cluster\_plot |
| 23 | 03460 | Fanconi anemia pathway | Genetic Information Processing | Replication and repair | 2.08504398827 | 0.387997228179 | 1.0 | 1 | cluster\_plot | 66(42.62) | cluster\_plot |
| 24 | 01040 | Biosynthesis of unsaturated fatty acids | Metabolism | Lipid metabolism | 2.08504398827 | 0.387997228179 | 1.0 | 1 | cluster\_plot | 66(4.72) | cluster\_plot |
| 25 | 03430 | Mismatch repair | Genetic Information Processing | Replication and repair | 2.02371916509 | 0.396866716308 | 1.0 | 1 | cluster\_plot | 68(35.85) | cluster\_plot |
| 26 | 02010 | ABC transporters | Environmental Information Processing | Membrane transport | 1.85963382738 | 0.422699644557 | 1.0 | 1 | cluster\_plot | 74(1.96) | cluster\_plot |
| 27 | 04066 | HIF-1 signaling pathway | Environmental Information Processing | Signal transduction | 1.83483870968 | 0.426894560633 | 1.0 | 1 | cluster\_plot | 75(16.49) | cluster\_plot |
| 28 | 03015 | mRNA surveillance pathway | Genetic Information Processing | Translation | 1.38304425353 | 0.431957278086 | 1.0 | 2 | cluster\_plot | 199(71.64) | cluster\_plot |
| 29 | 04728 | Dopaminergic synapse | Organismal Systems | Nervous system | 1.69892473118 | 0.451421908625 | 1.0 | 1 | cluster\_plot | 81(10.17) | cluster\_plot |
| 30 | 03030 | DNA replication | Genetic Information Processing | Replication and repair | 1.63824884793 | 0.463282980632 | 1.0 | 1 | cluster\_plot | 84(54.72) | cluster\_plot |
| 31 | 05230 | Central carbon metabolism in cancer | Human Diseases | Cancers | 1.5637829912 | 0.478693629268 | 1.0 | 1 | cluster\_plot | 88(8.90) | cluster\_plot |
| 32 | 04261 | Adrenergic signaling in cardiomyocytes | Organismal Systems | Circulatory system | 1.5462123958 | 0.482475561711 | 1.0 | 1 | cluster\_plot | 89(6.77) | cluster\_plot |
| 33 | 03420 | Nucleotide excision repair | Genetic Information Processing | Replication and repair | 1.5462123958 | 0.482475561711 | 1.0 | 1 | cluster\_plot | 89(49.25) | cluster\_plot |
| 34 | 04070 | Phosphatidylinositol signaling system | Environmental Information Processing | Signal transduction | 1.37612903226 | 0.522282560913 | 1.0 | 1 | cluster\_plot | 100(17.17) | cluster\_plot |
| 35 | 04113 | Meiosis - yeast | Cellular Processes | Cell growth and death | 1.28610189931 | 0.545980805274 | 1.0 | 1 | cluster\_plot | 107(24.11) | cluster\_plot |
| 36 | 00562 | Inositol phosphate metabolism | Metabolism | Carbohydrate metabolism | 1.26250369932 | 0.552529822956 | 1.0 | 1 | cluster\_plot | 109(16.26) | cluster\_plot |
| 37 | 00270 | Cysteine and methionine metabolism | Metabolism | Amino acid metabolism | 1.13729672087 | 0.589858309637 | 1.0 | 1 | cluster\_plot | 121(16.67) | cluster\_plot |
| 38 | 04068 | FoxO signaling pathway | Environmental Information Processing | Signal transduction | 1.11880409127 | 0.595764947943 | 1.0 | 1 | cluster\_plot | 123(14.62) | cluster\_plot |
| 39 | 03013 | RNA transport | Genetic Information Processing | Translation | 1.00082111437 | 0.598820392574 | 1.0 | 2 | cluster\_plot | 275(59.62) | cluster\_plot |
| 40 | 00620 | Pyruvate metabolism | Metabolism | Carbohydrate metabolism | 1.07510080645 | 0.610156368564 | 1.0 | 1 | cluster\_plot | 128(19.71) | cluster\_plot |
| 41 | 03040 | Spliceosome | Genetic Information Processing | Transcription | 0.95564516129 | 0.623262834293 | 1.0 | 2 | cluster\_plot | 288(68.28) | cluster\_plot |
| 42 | 04145 | Phagosome | Cellular Processes | Transport and catabolism | 0.990020886517 | 0.640013227993 | 1.0 | 1 | cluster\_plot | 139(18.92) | cluster\_plot |
| 43 | 04932 | Non-alcoholic fatty liver disease (NAFLD) | Human Diseases | Endocrine and metabolic diseases | 0.969104952294 | 0.64774537368 | 1.0 | 1 | cluster\_plot | 142(29.33) | cluster\_plot |
| 44 | 04146 | Peroxisome | Cellular Processes | Transport and catabolism | 0.876515307171 | 0.683949698232 | 1.0 | 1 | cluster\_plot | 157(46.99) | cluster\_plot |
| 45 | 04151 | PI3K-Akt signaling pathway | Environmental Information Processing | Signal transduction | 0.854738529353 | 0.692949535357 | 1.0 | 1 | cluster\_plot | 161(9.81) | cluster\_plot |
| 46 | 04114 | Oocyte meiosis | Cellular Processes | Cell growth and death | 0.849462365591 | 0.695158531499 | 1.0 | 1 | cluster\_plot | 162(20.59) | cluster\_plot |
| 47 | 00010 | Glycolysis / Gluconeogenesis | Metabolism | Carbohydrate metabolism | 0.828993392927 | 0.703834284806 | 1.0 | 1 | cluster\_plot | 166(19.11) | cluster\_plot |
| 48 | 01200 | Carbon metabolism | Metabolism | Overview | 0.81669378769 | 0.70475461104 | 1.0 | 2 | cluster\_plot | 337(2.95) | cluster\_plot |
| 49 | 01230 | Biosynthesis of amino acids | Metabolism | Overview | 0.795450307664 | 0.717971655804 | 1.0 | 2 | cluster\_plot | 346(8.44) | cluster\_plot |
| 50 | 05016 | Huntington's disease | Human Diseases | Neurodegenerative diseases | 0.731983527797 | 0.747261518828 | 1.0 | 1 | cluster\_plot | 188(38.42) | cluster\_plot |
| 51 | 04120 | Ubiquitin mediated proteolysis | Genetic Information Processing | Folding, sorting and degradation | 0.688064516129 | 0.768163166721 | 1.0 | 1 | cluster\_plot | 200(40.30) | cluster\_plot |
| 52 | 05203 | Viral carcinogenesis | Human Diseases | Cancers | 0.677896075004 | 0.773108501821 | 1.0 | 1 | cluster\_plot | 203(16.24) | cluster\_plot |
| 53 | 00190 | Oxidative phosphorylation | Metabolism | Energy metabolism | 0.0 | 1.0 | 1.0 | 0 | cluster\_plot | 196(14.44) | cluster\_plot |
| 54 | 04115 | p53 signaling pathway | Cellular Processes | Cell growth and death | 0.0 | 1.0 | 1.0 | 0 | cluster\_plot | 56(19.40) | cluster\_plot |
| 55 | 00710 | Carbon fixation in photosynthetic organisms | Metabolism | Energy metabolism | 0.0 | 1.0 | 1.0 | 0 | cluster\_plot | 107(23.91) | cluster\_plot |
| 56 | 05220 | Chronic myeloid leukemia | Human Diseases | Cancers | 0.0 | 1.0 | 1.0 | 0 | cluster\_plot | 42(8.22) | cluster\_plot |
| 57 | 00524 | Butirosin and neomycin biosynthesis | Metabolism | Biosynthesis of other secondary metabolites | 0.0 | 1.0 | 1.0 | 0 | cluster\_plot | 10(1.54) | cluster\_plot |
| 58 | 00480 | Glutathione metabolism | Metabolism | Metabolism of other amino acids | 0.0 | 1.0 | 1.0 | 0 | cluster\_plot | 139(15.52) | cluster\_plot |
| 59 | 05142 | Chagas disease (American trypanosomiasis) | Human Diseases | Infectious diseases | 0.0 | 1.0 | 1.0 | 0 | cluster\_plot | 225(8.18) | cluster\_plot |
| 60 | 05161 | Hepatitis B | Human Diseases | Infectious diseases | 0.0 | 1.0 | 1.0 | 0 | cluster\_plot | 77(8.67) | cluster\_plot |
| 61 | 04122 | Sulfur relay system | Genetic Information Processing | Folding, sorting and degradation | 0.0 | 1.0 | 1.0 | 0 | cluster\_plot | 21(17.31) | cluster\_plot |
| 62 | 05033 | Nicotine addiction | Human Diseases | Substance dependence | 0.0 | 1.0 | 1.0 | 0 | cluster\_plot | 13(0.88) | cluster\_plot |
| 63 | 00624 | Polycyclic aromatic hydrocarbon degradation | Metabolism | Xenobiotics biodegradation and metabolism | 0.0 | 1.0 | 1.0 | 0 | cluster\_plot | 54(1.10) | cluster\_plot |
| 64 | 05212 | Pancreatic cancer | Human Diseases | Cancers | 0.0 | 1.0 | 1.0 | 0 | cluster\_plot | 44(8.00) | cluster\_plot |
| 65 | 05110 | Vibrio cholerae infection | Human Diseases | Infectious diseases | 0.0 | 1.0 | 1.0 | 0 | cluster\_plot | 71(25.35) | cluster\_plot |
| 66 | 04650 | Natural killer cell mediated cytotoxicity | Organismal Systems | Immune system | 0.0 | 1.0 | 1.0 | 0 | cluster\_plot | 50(4.46) | cluster\_plot |
| 67 | 00680 | Methane metabolism | Metabolism | Energy metabolism | 0.0 | 1.0 | 1.0 | 0 | cluster\_plot | 91(6.36) | cluster\_plot |
| 68 | 00361 | Chlorocyclohexane and chlorobenzene degradation | Metabolism | Xenobiotics biodegradation and metabolism | 0.0 | 1.0 | 1.0 | 0 | cluster\_plot | 17(0.58) | cluster\_plot |
| 69 | 05166 | HTLV-I infection | Human Diseases | Infectious diseases | 0.0 | 1.0 | 1.0 | 0 | cluster\_plot | 152(17.70) | cluster\_plot |
| 70 | 04390 | Hippo signaling pathway | Environmental Information Processing | Signal transduction | 0.0 | 1.0 | 1.0 | 0 | cluster\_plot | 54(5.37) | cluster\_plot |
| 71 | 04024 | cAMP signaling pathway | Environmental Information Processing | Signal transduction | 0.0 | 1.0 | 1.0 | 0 | cluster\_plot | 95(4.85) | cluster\_plot |
| 72 | 04623 | Cytosolic DNA-sensing pathway | Organismal Systems | Immune system | 0.0 | 1.0 | 1.0 | 0 | cluster\_plot | 40(25.42) | cluster\_plot |
| 73 | 00627 | Aminobenzoate degradation | Metabolism | Xenobiotics biodegradation and metabolism | 0.0 | 1.0 | 1.0 | 0 | cluster\_plot | 49(1.69) | cluster\_plot |
| 74 | 04611 | Platelet activation | Organismal Systems | Immune system | 0.0 | 1.0 | 1.0 | 0 | cluster\_plot | 35(3.45) | cluster\_plot |
| 75 | 05020 | Prion diseases | Human Diseases | Neurodegenerative diseases | 0.0 | 1.0 | 1.0 | 0 | cluster\_plot | 39(6.49) | cluster\_plot |
| 76 | 04013 | MAPK signaling pathway - fly | Environmental Information Processing | Signal transduction | 0.0 | 1.0 | 1.0 | 0 | cluster\_plot | 27(6.67) | cluster\_plot |
| 77 | 04724 | Glutamatergic synapse | Organismal Systems | Nervous system | 0.0 | 1.0 | 1.0 | 0 | cluster\_plot | 68(5.19) | cluster\_plot |
| 78 | 00592 | alpha-Linolenic acid metabolism | Metabolism | Lipid metabolism | 0.0 | 1.0 | 1.0 | 0 | cluster\_plot | 89(18.67) | cluster\_plot |
| 79 | 04010 | MAPK signaling pathway | Environmental Information Processing | Signal transduction | 0.0 | 1.0 | 1.0 | 0 | cluster\_plot | 94(6.64) | cluster\_plot |
| 80 | 05131 | Shigellosis | Human Diseases | Infectious diseases | 0.0 | 1.0 | 1.0 | 0 | cluster\_plot | 55(12.94) | cluster\_plot |
| 81 | 00564 | Glycerophospholipid metabolism | Metabolism | Lipid metabolism | 0.0 | 1.0 | 1.0 | 0 | cluster\_plot | 147(15.77) | cluster\_plot |
| 82 | 00904 | Diterpenoid biosynthesis | Metabolism | Metabolism of terpenoids and polyketides | 0.0 | 1.0 | 1.0 | 0 | cluster\_plot | 42(5.36) | cluster\_plot |
| 83 | 00950 | Isoquinoline alkaloid biosynthesis | Metabolism | Biosynthesis of other secondary metabolites | 0.0 | 1.0 | 1.0 | 0 | cluster\_plot | 24(3.90) | cluster\_plot |
| 84 | 04974 | Protein digestion and absorption | Organismal Systems | Digestive system | 0.0 | 1.0 | 1.0 | 0 | cluster\_plot | 16(3.74) | cluster\_plot |
| 85 | 00052 | Galactose metabolism | Metabolism | Carbohydrate metabolism | 0.0 | 1.0 | 1.0 | 0 | cluster\_plot | 81(10.60) | cluster\_plot |
| 86 | 00030 | Pentose phosphate pathway | Metabolism | Carbohydrate metabolism | 0.0 | 1.0 | 1.0 | 0 | cluster\_plot | 73(11.19) | cluster\_plot |
| 87 | 03022 | Basal transcription factors | Genetic Information Processing | Transcription | 0.0 | 1.0 | 1.0 | 0 | cluster\_plot | 63(66.67) | cluster\_plot |
| 88 | 00410 | beta-Alanine metabolism | Metabolism | Metabolism of other amino acids | 0.0 | 1.0 | 1.0 | 0 | cluster\_plot | 64(17.44) | cluster\_plot |
| 89 | 04110 | Cell cycle | Cellular Processes | Cell growth and death | 0.0 | 1.0 | 1.0 | 0 | cluster\_plot | 181(33.55) | cluster\_plot |
| 90 | 03018 | RNA degradation | Genetic Information Processing | Folding, sorting and degradation | 0.0 | 1.0 | 1.0 | 0 | cluster\_plot | 177(53.93) | cluster\_plot |
| 91 | 04141 | Protein processing in endoplasmic reticulum | Genetic Information Processing | Folding, sorting and degradation | 0.0 | 1.0 | 1.0 | 0 | cluster\_plot | 349(38.30) | cluster\_plot |
| 92 | 04971 | Gastric acid secretion | Organismal Systems | Digestive system | 0.0 | 1.0 | 1.0 | 0 | cluster\_plot | 14(1.30) | cluster\_plot |
| 93 | 00940 | Phenylpropanoid biosynthesis | Metabolism | Biosynthesis of other secondary metabolites | 0.0 | 1.0 | 1.0 | 0 | cluster\_plot | 298(12.08) | cluster\_plot |
| 94 | 04144 | Endocytosis | Cellular Processes | Transport and catabolism | 0.0 | 1.0 | 1.0 | 0 | cluster\_plot | 211(19.69) | cluster\_plot |
| 95 | 00254 | Aflatoxin biosynthesis | Metabolism | Biosynthesis of other secondary metabolites | 0.0 | 1.0 | 1.0 | 0 | cluster\_plot | 3(2.04) | cluster\_plot |
| 96 | 00240 | Pyrimidine metabolism | Metabolism | Nucleotide metabolism | 0.0 | 1.0 | 1.0 | 0 | cluster\_plot | 177(16.23) | cluster\_plot |
| 97 | 05032 | Morphine addiction | Human Diseases | Substance dependence | 0.0 | 1.0 | 1.0 | 0 | cluster\_plot | 14(0.60) | cluster\_plot |
| 98 | 04725 | Cholinergic synapse | Organismal Systems | Nervous system | 0.0 | 1.0 | 1.0 | 0 | cluster\_plot | 29(2.86) | cluster\_plot |
| 99 | 05168 | Herpes simplex infection | Human Diseases | Infectious diseases | 0.0 | 1.0 | 1.0 | 0 | cluster\_plot | 122(18.49) | cluster\_plot |
| 100 | 05145 | Toxoplasmosis | Human Diseases | Infectious diseases | 0.0 | 1.0 | 1.0 | 0 | cluster\_plot | 282(6.84) | cluster\_plot |
| 101 | 04918 | Thyroid hormone synthesis | Organismal Systems | Endocrine system | 0.0 | 1.0 | 1.0 | 0 | cluster\_plot | 26(4.59) | cluster\_plot |
| 102 | 00740 | Riboflavin metabolism | Metabolism | Metabolism of cofactors and vitamins | 0.0 | 1.0 | 1.0 | 0 | cluster\_plot | 15(9.52) | cluster\_plot |
| 103 | 05120 | Epithelial cell signaling in Helicobacter pylori infection | Human Diseases | Infectious diseases | 0.0 | 1.0 | 1.0 | 0 | cluster\_plot | 50(12.15) | cluster\_plot |
| 104 | 04662 | B cell receptor signaling pathway | Organismal Systems | Immune system | 0.0 | 1.0 | 1.0 | 0 | cluster\_plot | 59(10.45) | cluster\_plot |
| 105 | 05205 | Proteoglycans in cancer | Human Diseases | Cancers | 0.0 | 1.0 | 1.0 | 0 | cluster\_plot | 86(4.73) | cluster\_plot |
| 106 | 00260 | Glycine, serine and threonine metabolism | Metabolism | Amino acid metabolism | 0.0 | 1.0 | 1.0 | 0 | cluster\_plot | 105(18.39) | cluster\_plot |
| 107 | 00720 | Carbon fixation pathways in prokaryotes | Metabolism | Energy metabolism | 0.0 | 1.0 | 1.0 | 0 | cluster\_plot | 53(6.56) | cluster\_plot |
| 108 | 01053 | Biosynthesis of siderophore group nonribosomal peptides | Metabolism | Metabolism of terpenoids and polyketides | 0.0 | 1.0 | 1.0 | 0 | cluster\_plot | 1(1.85) | cluster\_plot |
| 109 | 00626 | Naphthalene degradation | Metabolism | Xenobiotics biodegradation and metabolism | 0.0 | 1.0 | 1.0 | 0 | cluster\_plot | 19(2.29) | cluster\_plot |
| 110 | 00785 | Lipoic acid metabolism | Metabolism | Metabolism of cofactors and vitamins | 0.0 | 1.0 | 1.0 | 0 | cluster\_plot | 8(13.33) | cluster\_plot |
| 111 | 04810 | Regulation of actin cytoskeleton | Cellular Processes | Cell motility | 0.0 | 1.0 | 1.0 | 0 | cluster\_plot | 106(10.23) | cluster\_plot |
| 112 | 04712 | Circadian rhythm - plant | Organismal Systems | Environmental adaptation | 0.0 | 1.0 | 1.0 | 0 | cluster\_plot | 67(54.05) | cluster\_plot |
| 113 | 00970 | Aminoacyl-tRNA biosynthesis | Genetic Information Processing | Translation | 0.0 | 1.0 | 1.0 | 0 | cluster\_plot | 93(19.55) | cluster\_plot |
| 114 | 00401 | Novobiocin biosynthesis | Metabolism | Biosynthesis of other secondary metabolites | 0.0 | 1.0 | 1.0 | 0 | cluster\_plot | 7(2.44) | cluster\_plot |
| 115 | 05231 | Choline metabolism in cancer | Human Diseases | Cancers | 0.0 | 1.0 | 1.0 | 0 | cluster\_plot | 98(17.07) | cluster\_plot |
| 116 | 00523 | Polyketide sugar unit biosynthesis | Metabolism | Metabolism of terpenoids and polyketides | 0.0 | 1.0 | 1.0 | 0 | cluster\_plot | 4(1.32) | cluster\_plot |
| 117 | 02020 | Two-component system | Environmental Information Processing | Signal transduction | 0.0 | 1.0 | 1.0 | 0 | cluster\_plot | 19(1.59) | cluster\_plot |
| 118 | 04919 | Thyroid hormone signaling pathway | Organismal Systems | Endocrine system | 0.0 | 1.0 | 1.0 | 0 | cluster\_plot | 84(11.92) | cluster\_plot |
| 119 | 00400 | Phenylalanine, tyrosine and tryptophan biosynthesis | Metabolism | Amino acid metabolism | 0.0 | 1.0 | 1.0 | 0 | cluster\_plot | 67(12.00) | cluster\_plot |
| 120 | 04921 | Oxytocin signaling pathway | Organismal Systems | Endocrine system | 0.0 | 1.0 | 1.0 | 0 | cluster\_plot | 103(8.51) | cluster\_plot |
| 121 | 03450 | Non-homologous end-joining | Genetic Information Processing | Replication and repair | 0.0 | 1.0 | 1.0 | 0 | cluster\_plot | 6(23.81) | cluster\_plot |
| 122 | 04012 | ErbB signaling pathway | Environmental Information Processing | Signal transduction | 0.0 | 1.0 | 1.0 | 0 | cluster\_plot | 39(6.52) | cluster\_plot |
| 123 | 00630 | Glyoxylate and dicarboxylate metabolism | Metabolism | Carbohydrate metabolism | 0.0 | 1.0 | 1.0 | 0 | cluster\_plot | 88(13.84) | cluster\_plot |
| 124 | 05164 | Influenza A | Human Diseases | Infectious diseases | 0.0 | 1.0 | 1.0 | 0 | cluster\_plot | 226(15.04) | cluster\_plot |
| 125 | 00072 | Synthesis and degradation of ketone bodies | Metabolism | Lipid metabolism | 0.0 | 1.0 | 1.0 | 0 | cluster\_plot | 11(21.05) | cluster\_plot |
| 126 | 04917 | Prolactin signaling pathway | Organismal Systems | Endocrine system | 0.0 | 1.0 | 1.0 | 0 | cluster\_plot | 39(6.06) | cluster\_plot |
| 127 | 00604 | Glycosphingolipid biosynthesis - ganglio series | Metabolism | Glycan biosynthesis and metabolism | 0.0 | 1.0 | 1.0 | 0 | cluster\_plot | 7(2.60) | cluster\_plot |
| 128 | 04664 | Fc epsilon RI signaling pathway | Organismal Systems | Immune system | 0.0 | 1.0 | 1.0 | 0 | cluster\_plot | 38(7.94) | cluster\_plot |
| 129 | 04622 | RIG-I-like receptor signaling pathway | Organismal Systems | Immune system | 0.0 | 1.0 | 1.0 | 0 | cluster\_plot | 13(9.84) | cluster\_plot |
| 130 | 04270 | Vascular smooth muscle contraction | Organismal Systems | Circulatory system | 0.0 | 1.0 | 1.0 | 0 | cluster\_plot | 54(3.79) | cluster\_plot |
| 131 | 00965 | Betalain biosynthesis | Metabolism | Biosynthesis of other secondary metabolites | 0.0 | 1.0 | 1.0 | 0 | cluster\_plot | 3(2.33) | cluster\_plot |
| 132 | 04910 | Insulin signaling pathway | Organismal Systems | Endocrine system | 0.0 | 1.0 | 1.0 | 0 | cluster\_plot | 167(22.11) | cluster\_plot |
| 133 | 00980 | Metabolism of xenobiotics by cytochrome P450 | Metabolism | Xenobiotics biodegradation and metabolism | 0.0 | 1.0 | 1.0 | 0 | cluster\_plot | 88(2.46) | cluster\_plot |
| 134 | 05169 | Epstein-Barr virus infection | Human Diseases | Infectious diseases | 0.0 | 1.0 | 1.0 | 0 | cluster\_plot | 305(34.05) | cluster\_plot |
| 135 | 05014 | Amyotrophic lateral sclerosis (ALS) | Human Diseases | Neurodegenerative diseases | 0.0 | 1.0 | 1.0 | 0 | cluster\_plot | 37(13.04) | cluster\_plot |
| 136 | 00983 | Drug metabolism - other enzymes | Metabolism | Xenobiotics biodegradation and metabolism | 0.0 | 1.0 | 1.0 | 0 | cluster\_plot | 31(13.89) | cluster\_plot |
| 137 | 00830 | Retinol metabolism | Metabolism | Metabolism of cofactors and vitamins | 0.0 | 1.0 | 1.0 | 0 | cluster\_plot | 42(5.47) | cluster\_plot |
| 138 | 00071 | Fatty acid degradation | Metabolism | Lipid metabolism | 0.0 | 1.0 | 1.0 | 0 | cluster\_plot | 65(3.73) | cluster\_plot |
| 139 | 00623 | Toluene degradation | Metabolism | Xenobiotics biodegradation and metabolism | 0.0 | 1.0 | 1.0 | 0 | cluster\_plot | 17(0.85) | cluster\_plot |
| 140 | 00051 | Fructose and mannose metabolism | Metabolism | Carbohydrate metabolism | 0.0 | 1.0 | 1.0 | 0 | cluster\_plot | 95(10.91) | cluster\_plot |
| 141 | 00750 | Vitamin B6 metabolism | Metabolism | Metabolism of cofactors and vitamins | 0.0 | 1.0 | 1.0 | 0 | cluster\_plot | 19(9.09) | cluster\_plot |
| 142 | 05211 | Renal cell carcinoma | Human Diseases | Cancers | 0.0 | 1.0 | 1.0 | 0 | cluster\_plot | 42(8.43) | cluster\_plot |
| 143 | 04914 | Progesterone-mediated oocyte maturation | Organismal Systems | Endocrine system | 0.0 | 1.0 | 1.0 | 0 | cluster\_plot | 87(30.30) | cluster\_plot |
| 144 | 04015 | Rap1 signaling pathway | Environmental Information Processing | Signal transduction | 0.0 | 1.0 | 1.0 | 0 | cluster\_plot | 57(3.31) | cluster\_plot |
| 145 | 00942 | Anthocyanin biosynthesis | Metabolism | Biosynthesis of other secondary metabolites | 0.0 | 1.0 | 1.0 | 0 | cluster\_plot | 15(2.83) | cluster\_plot |
| 146 | 00790 | Folate biosynthesis | Metabolism | Metabolism of cofactors and vitamins | 0.0 | 1.0 | 1.0 | 0 | cluster\_plot | 20(8.93) | cluster\_plot |
| 147 | 00195 | Photosynthesis | Metabolism | Energy metabolism | 0.0 | 1.0 | 1.0 | 0 | cluster\_plot | 67(27.17) | cluster\_plot |
| 148 | 04666 | Fc gamma R-mediated phagocytosis | Organismal Systems | Immune system | 0.0 | 1.0 | 1.0 | 0 | cluster\_plot | 100(21.13) | cluster\_plot |
| 149 | 04630 | Jak-STAT signaling pathway | Environmental Information Processing | Signal transduction | 0.0 | 1.0 | 1.0 | 0 | cluster\_plot | 8(3.05) | cluster\_plot |
| 150 | 04350 | TGF-beta signaling pathway | Environmental Information Processing | Signal transduction | 0.0 | 1.0 | 1.0 | 0 | cluster\_plot | 65(12.05) | cluster\_plot |
| 151 | 00640 | Propanoate metabolism | Metabolism | Carbohydrate metabolism | 0.0 | 1.0 | 1.0 | 0 | cluster\_plot | 33(8.23) | cluster\_plot |
| 152 | 04964 | Proximal tubule bicarbonate reclamation | Organismal Systems | Excretory system | 0.0 | 1.0 | 1.0 | 0 | cluster\_plot | 16(3.70) | cluster\_plot |
| 153 | 04520 | Adherens junction | Cellular Processes | Cellular commiunity | 0.0 | 1.0 | 1.0 | 0 | cluster\_plot | 46(5.94) | cluster\_plot |
| 154 | 03410 | Base excision repair | Genetic Information Processing | Replication and repair | 0.0 | 1.0 | 1.0 | 0 | cluster\_plot | 58(26.92) | cluster\_plot |
| 155 | 04621 | NOD-like receptor signaling pathway | Organismal Systems | Immune system | 0.0 | 1.0 | 1.0 | 0 | cluster\_plot | 38(5.80) | cluster\_plot |
| 156 | 04978 | Mineral absorption | Organismal Systems | Digestive system | 0.0 | 1.0 | 1.0 | 0 | cluster\_plot | 25(5.49) | cluster\_plot |
| 157 | 00966 | Glucosinolate biosynthesis | Metabolism | Biosynthesis of other secondary metabolites | 0.0 | 1.0 | 1.0 | 0 | cluster\_plot | 5(0.88) | cluster\_plot |
| 158 | 00730 | Thiamine metabolism | Metabolism | Metabolism of cofactors and vitamins | 0.0 | 1.0 | 1.0 | 0 | cluster\_plot | 25(12.68) | cluster\_plot |
| 159 | 05162 | Measles | Human Diseases | Infectious diseases | 0.0 | 1.0 | 1.0 | 0 | cluster\_plot | 252(11.19) | cluster\_plot |
| 160 | 05340 | Primary immunodeficiency | Human Diseases | Immune diseases | 0.0 | 1.0 | 1.0 | 0 | cluster\_plot | 1(1.92) | cluster\_plot |
| 161 | 05410 | Hypertrophic cardiomyopathy (HCM) | Human Diseases | Cardiovascular diseases | 0.0 | 1.0 | 1.0 | 0 | cluster\_plot | 30(3.30) | cluster\_plot |
| 162 | 05034 | Alcoholism | Human Diseases | Substance dependence | 0.0 | 1.0 | 1.0 | 0 | cluster\_plot | 130(8.81) | cluster\_plot |
| 163 | 04150 | mTOR signaling pathway | Environmental Information Processing | Signal transduction | 0.0 | 1.0 | 1.0 | 0 | cluster\_plot | 75(25.49) | cluster\_plot |
| 164 | 00909 | Sesquiterpenoid and triterpenoid biosynthesis | Metabolism | Metabolism of terpenoids and polyketides | 0.0 | 1.0 | 1.0 | 0 | cluster\_plot | 67(3.51) | cluster\_plot |
| 165 | 00903 | Limonene and pinene degradation | Metabolism | Metabolism of terpenoids and polyketides | 0.0 | 1.0 | 1.0 | 0 | cluster\_plot | 55(1.98) | cluster\_plot |
| 166 | 00603 | Glycosphingolipid biosynthesis - globo series | Metabolism | Glycan biosynthesis and metabolism | 0.0 | 1.0 | 1.0 | 0 | cluster\_plot | 18(7.14) | cluster\_plot |
| 167 | 04310 | Wnt signaling pathway | Environmental Information Processing | Signal transduction | 0.0 | 1.0 | 1.0 | 0 | cluster\_plot | 97(13.85) | cluster\_plot |
| 168 | 00770 | Pantothenate and CoA biosynthesis | Metabolism | Metabolism of cofactors and vitamins | 0.0 | 1.0 | 1.0 | 0 | cluster\_plot | 53(20.51) | cluster\_plot |
| 169 | 04510 | Focal adhesion | Cellular Processes | Cellular commiunity | 0.0 | 1.0 | 1.0 | 0 | cluster\_plot | 64(6.45) | cluster\_plot |
| 170 | 05223 | Non-small cell lung cancer | Human Diseases | Cancers | 0.0 | 1.0 | 1.0 | 0 | cluster\_plot | 39(8.96) | cluster\_plot |
| 171 | 00300 | Lysine biosynthesis | Metabolism | Amino acid metabolism | 0.0 | 1.0 | 1.0 | 0 | cluster\_plot | 19(10.00) | cluster\_plot |
| 172 | 04723 | Retrograde endocannabinoid signaling | Organismal Systems | Nervous system | 0.0 | 1.0 | 1.0 | 0 | cluster\_plot | 58(3.27) | cluster\_plot |
| 173 | 00362 | Benzoate degradation | Metabolism | Xenobiotics biodegradation and metabolism | 0.0 | 1.0 | 1.0 | 0 | cluster\_plot | 13(1.72) | cluster\_plot |
| 174 | 00130 | Ubiquinone and other terpenoid-quinone biosynthesis | Metabolism | Metabolism of cofactors and vitamins | 0.0 | 1.0 | 1.0 | 0 | cluster\_plot | 48(9.80) | cluster\_plot |
| 175 | 00250 | Alanine, aspartate and glutamate metabolism | Metabolism | Amino acid metabolism | 0.0 | 1.0 | 1.0 | 0 | cluster\_plot | 77(19.55) | cluster\_plot |
| 176 | 04744 | Phototransduction | Organismal Systems | Sensory system | 0.0 | 1.0 | 1.0 | 0 | cluster\_plot | 15(3.33) | cluster\_plot |
| 177 | 00440 | Phosphonate and phosphinate metabolism | Metabolism | Metabolism of other amino acids | 0.0 | 1.0 | 1.0 | 0 | cluster\_plot | 11(3.30) | cluster\_plot |
| 178 | 00600 | Sphingolipid metabolism | Metabolism | Lipid metabolism | 0.0 | 1.0 | 1.0 | 0 | cluster\_plot | 40(17.86) | cluster\_plot |
| 179 | 04730 | Long-term depression | Organismal Systems | Nervous system | 0.0 | 1.0 | 1.0 | 0 | cluster\_plot | 37(4.71) | cluster\_plot |
| 180 | 05146 | Amoebiasis | Human Diseases | Infectious diseases | 0.0 | 1.0 | 1.0 | 0 | cluster\_plot | 36(3.42) | cluster\_plot |
| 181 | 00534 | Glycosaminoglycan biosynthesis - heparan sulfate / heparin | Metabolism | Glycan biosynthesis and metabolism | 0.0 | 1.0 | 1.0 | 0 | cluster\_plot | 20(6.67) | cluster\_plot |
| 182 | 00563 | Glycosylphosphatidylinositol(GPI)-anchor biosynthesis | Metabolism | Glycan biosynthesis and metabolism | 0.0 | 1.0 | 1.0 | 0 | cluster\_plot | 38(37.74) | cluster\_plot |
| 183 | 04340 | Hedgehog signaling pathway | Environmental Information Processing | Signal transduction | 0.0 | 1.0 | 1.0 | 0 | cluster\_plot | 20(6.67) | cluster\_plot |
| 184 | 00520 | Amino sugar and nucleotide sugar metabolism | Metabolism | Carbohydrate metabolism | 0.0 | 1.0 | 1.0 | 0 | cluster\_plot | 195(12.62) | cluster\_plot |
| 185 | 03050 | Proteasome | Genetic Information Processing | Folding, sorting and degradation | 0.0 | 1.0 | 1.0 | 0 | cluster\_plot | 94(60.00) | cluster\_plot |
| 186 | 00310 | Lysine degradation | Metabolism | Amino acid metabolism | 0.0 | 1.0 | 1.0 | 0 | cluster\_plot | 49(5.20) | cluster\_plot |
| 187 | 04940 | Type I diabetes mellitus | Human Diseases | Endocrine and metabolic diseases | 0.0 | 1.0 | 1.0 | 0 | cluster\_plot | 17(6.45) | cluster\_plot |
| 188 | 05416 | Viral myocarditis | Human Diseases | Cardiovascular diseases | 0.0 | 1.0 | 1.0 | 0 | cluster\_plot | 21(6.00) | cluster\_plot |
| 189 | 04912 | GnRH signaling pathway | Organismal Systems | Endocrine system | 0.0 | 1.0 | 1.0 | 0 | cluster\_plot | 64(6.85) | cluster\_plot |
| 190 | 04750 | Inflammatory mediator regulation of TRP channels | Organismal Systems | Sensory system | 0.0 | 1.0 | 1.0 | 0 | cluster\_plot | 24(1.60) | cluster\_plot |
| 191 | 04742 | Taste transduction | Organismal Systems | Sensory system | 0.0 | 1.0 | 1.0 | 0 | cluster\_plot | 1(1.64) | cluster\_plot |
| 192 | 03070 | Bacterial secretion system | Environmental Information Processing | Membrane transport | 0.0 | 1.0 | 1.0 | 0 | cluster\_plot | 26(6.02) | cluster\_plot |
| 193 | 05221 | Acute myeloid leukemia | Human Diseases | Cancers | 0.0 | 1.0 | 1.0 | 0 | cluster\_plot | 31(8.33) | cluster\_plot |
| 194 | 00550 | Peptidoglycan biosynthesis | Metabolism | Glycan biosynthesis and metabolism | 0.0 | 1.0 | 1.0 | 0 | cluster\_plot | 3(2.35) | cluster\_plot |
| 195 | 04972 | Pancreatic secretion | Organismal Systems | Digestive system | 0.0 | 1.0 | 1.0 | 0 | cluster\_plot | 52(4.13) | cluster\_plot |
| 196 | 00565 | Ether lipid metabolism | Metabolism | Lipid metabolism | 0.0 | 1.0 | 1.0 | 0 | cluster\_plot | 50(11.94) | cluster\_plot |
| 197 | 00908 | Zeatin biosynthesis | Metabolism | Metabolism of terpenoids and polyketides | 0.0 | 1.0 | 1.0 | 0 | cluster\_plot | 26(7.94) | cluster\_plot |
| 198 | 00941 | Flavonoid biosynthesis | Metabolism | Biosynthesis of other secondary metabolites | 0.0 | 1.0 | 1.0 | 0 | cluster\_plot | 86(8.97) | cluster\_plot |
| 199 | 04961 | Endocrine and other factor-regulated calcium reabsorption | Organismal Systems | Excretory system | 0.0 | 1.0 | 1.0 | 0 | cluster\_plot | 53(9.09) | cluster\_plot |
| 200 | 00140 | Steroid hormone biosynthesis | Metabolism | Lipid metabolism | 0.0 | 1.0 | 1.0 | 0 | cluster\_plot | 14(1.10) | cluster\_plot |
| 201 | 00330 | Arginine and proline metabolism | Metabolism | Amino acid metabolism | 0.0 | 1.0 | 1.0 | 0 | cluster\_plot | 116(13.29) | cluster\_plot |
| 202 | 04977 | Vitamin digestion and absorption | Organismal Systems | Digestive system | 0.0 | 1.0 | 1.0 | 0 | cluster\_plot | 6(1.65) | cluster\_plot |
| 203 | 00360 | Phenylalanine metabolism | Metabolism | Amino acid metabolism | 0.0 | 1.0 | 1.0 | 0 | cluster\_plot | 156(8.38) | cluster\_plot |
| 204 | 00471 | D-Glutamine and D-glutamate metabolism | Metabolism | Metabolism of other amino acids | 0.0 | 1.0 | 1.0 | 0 | cluster\_plot | 11(3.85) | cluster\_plot |
| 205 | 04660 | T cell receptor signaling pathway | Organismal Systems | Immune system | 0.0 | 1.0 | 1.0 | 0 | cluster\_plot | 53(7.00) | cluster\_plot |
| 206 | 04745 | Phototransduction - fly | Organismal Systems | Sensory system | 0.0 | 1.0 | 1.0 | 0 | cluster\_plot | 14(1.96) | cluster\_plot |
| 207 | 04915 | Estrogen signaling pathway | Organismal Systems | Endocrine system | 0.0 | 1.0 | 1.0 | 0 | cluster\_plot | 87(5.93) | cluster\_plot |
| 208 | 04916 | Melanogenesis | Organismal Systems | Endocrine system | 0.0 | 1.0 | 1.0 | 0 | cluster\_plot | 52(6.10) | cluster\_plot |
| 209 | 00364 | Fluorobenzoate degradation | Metabolism | Xenobiotics biodegradation and metabolism | 0.0 | 1.0 | 1.0 | 0 | cluster\_plot | 17(1.41) | cluster\_plot |
| 210 | 05218 | Melanoma | Human Diseases | Cancers | 0.0 | 1.0 | 1.0 | 0 | cluster\_plot | 39(8.93) | cluster\_plot |
| 211 | 04360 | Axon guidance | Organismal Systems | Development | 0.0 | 1.0 | 1.0 | 0 | cluster\_plot | 68(4.80) | cluster\_plot |
| 212 | 04722 | Neurotrophin signaling pathway | Organismal Systems | Nervous system | 0.0 | 1.0 | 1.0 | 0 | cluster\_plot | 268(11.93) | cluster\_plot |
| 213 | 04370 | VEGF signaling pathway | Environmental Information Processing | Signal transduction | 0.0 | 1.0 | 1.0 | 0 | cluster\_plot | 53(14.29) | cluster\_plot |
| 214 | 05215 | Prostate cancer | Human Diseases | Cancers | 0.0 | 1.0 | 1.0 | 0 | cluster\_plot | 66(13.79) | cluster\_plot |
| 215 | 04911 | Insulin secretion | Organismal Systems | Endocrine system | 0.0 | 1.0 | 1.0 | 0 | cluster\_plot | 3(1.11) | cluster\_plot |
| 216 | 04142 | Lysosome | Cellular Processes | Transport and catabolism | 0.0 | 1.0 | 1.0 | 0 | cluster\_plot | 112(25.38) | cluster\_plot |
| 217 | 05216 | Thyroid cancer | Human Diseases | Cancers | 0.0 | 1.0 | 1.0 | 0 | cluster\_plot | 30(6.98) | cluster\_plot |
| 218 | 00100 | Steroid biosynthesis | Metabolism | Lipid metabolism | 0.0 | 1.0 | 1.0 | 0 | cluster\_plot | 58(15.24) | cluster\_plot |
| 219 | 04062 | Chemokine signaling pathway | Organismal Systems | Immune system | 0.0 | 1.0 | 1.0 | 0 | cluster\_plot | 45(3.87) | cluster\_plot |
| 220 | 00960 | Tropane, piperidine and pyridine alkaloid biosynthesis | Metabolism | Biosynthesis of other secondary metabolites | 0.0 | 1.0 | 1.0 | 0 | cluster\_plot | 37(6.67) | cluster\_plot |
| 221 | 05100 | Bacterial invasion of epithelial cells | Human Diseases | Infectious diseases | 0.0 | 1.0 | 1.0 | 0 | cluster\_plot | 36(10.53) | cluster\_plot |
| 222 | 05200 | Pathways in cancer | Human Diseases | Cancers | 0.0 | 1.0 | 1.0 | 0 | cluster\_plot | 110(6.04) | cluster\_plot |
| 223 | 04550 | Signaling pathways regulating pluripotency of stem cells | Cellular Processes | Cellular commiunity | 0.0 | 1.0 | 1.0 | 0 | cluster\_plot | 39(3.39) | cluster\_plot |
| 224 | 00430 | Taurine and hypotaurine metabolism | Metabolism | Metabolism of other amino acids | 0.0 | 1.0 | 1.0 | 0 | cluster\_plot | 22(7.69) | cluster\_plot |
| 225 | 04320 | Dorso-ventral axis formation | Organismal Systems | Development | 0.0 | 1.0 | 1.0 | 0 | cluster\_plot | 27(7.41) | cluster\_plot |
| 226 | 04966 | Collecting duct acid secretion | Organismal Systems | Excretory system | 0.0 | 1.0 | 1.0 | 0 | cluster\_plot | 41(31.43) | cluster\_plot |
| 227 | 05204 | Chemical carcinogenesis | Human Diseases | Cancers | 0.0 | 1.0 | 1.0 | 0 | cluster\_plot | 79(2.29) | cluster\_plot |
| 228 | 00905 | Brassinosteroid biosynthesis | Metabolism | Metabolism of terpenoids and polyketides | 0.0 | 1.0 | 1.0 | 0 | cluster\_plot | 29(9.86) | cluster\_plot |
| 229 | 05031 | Amphetamine addiction | Human Diseases | Substance dependence | 0.0 | 1.0 | 1.0 | 0 | cluster\_plot | 39(2.70) | cluster\_plot |
| 230 | 00531 | Glycosaminoglycan degradation | Metabolism | Glycan biosynthesis and metabolism | 0.0 | 1.0 | 1.0 | 0 | cluster\_plot | 21(7.25) | cluster\_plot |
| 231 | 00643 | Styrene degradation | Metabolism | Xenobiotics biodegradation and metabolism | 0.0 | 1.0 | 1.0 | 0 | cluster\_plot | 5(5.56) | cluster\_plot |
| 232 | 00513 | Various types of N-glycan biosynthesis | Metabolism | Glycan biosynthesis and metabolism | 0.0 | 1.0 | 1.0 | 0 | cluster\_plot | 54(10.40) | cluster\_plot |
| 233 | 05202 | Transcriptional misregulation in cancers | Human Diseases | Cancers | 0.0 | 1.0 | 1.0 | 0 | cluster\_plot | 66(5.94) | cluster\_plot |
| 234 | 05210 | Colorectal cancer | Human Diseases | Cancers | 0.0 | 1.0 | 1.0 | 0 | cluster\_plot | 59(14.71) | cluster\_plot |
| 235 | 00982 | Drug metabolism - cytochrome P450 | Metabolism | Xenobiotics biodegradation and metabolism | 0.0 | 1.0 | 1.0 | 0 | cluster\_plot | 85(2.37) | cluster\_plot |
| 236 | 04727 | GABAergic synapse | Organismal Systems | Nervous system | 0.0 | 1.0 | 1.0 | 0 | cluster\_plot | 52(5.13) | cluster\_plot |
| 237 | 05010 | Alzheimer's disease | Human Diseases | Neurodegenerative diseases | 0.0 | 1.0 | 1.0 | 0 | cluster\_plot | 181(36.71) | cluster\_plot |
| 238 | 00232 | Caffeine metabolism | Metabolism | Biosynthesis of other secondary metabolites | 0.0 | 1.0 | 1.0 | 0 | cluster\_plot | 3(5.13) | cluster\_plot |
| 239 | 04022 | cGMP - PKG signaling pathway | Environmental Information Processing | Signal transduction | 0.0 | 1.0 | 1.0 | 0 | cluster\_plot | 88(7.53) | cluster\_plot |
| 240 | 05012 | Parkinson's disease | Human Diseases | Neurodegenerative diseases | 0.0 | 1.0 | 1.0 | 0 | cluster\_plot | 129(30.67) | cluster\_plot |
| 241 | 05217 | Basal cell carcinoma | Human Diseases | Cancers | 0.0 | 1.0 | 1.0 | 0 | cluster\_plot | 10(3.92) | cluster\_plot |
| 242 | 00945 | Stilbenoid, diarylheptanoid and gingerol biosynthesis | Metabolism | Biosynthesis of other secondary metabolites | 0.0 | 1.0 | 1.0 | 0 | cluster\_plot | 92(10.00) | cluster\_plot |
| 243 | 04721 | Synaptic vesicle cycle | Organismal Systems | Nervous system | 0.0 | 1.0 | 1.0 | 0 | cluster\_plot | 100(29.11) | cluster\_plot |
| 244 | 00040 | Pentose and glucuronate interconversions | Metabolism | Carbohydrate metabolism | 0.0 | 1.0 | 1.0 | 0 | cluster\_plot | 170(12.12) | cluster\_plot |
| 245 | 00590 | Arachidonic acid metabolism | Metabolism | Lipid metabolism | 0.0 | 1.0 | 1.0 | 0 | cluster\_plot | 45(6.25) | cluster\_plot |
| 246 | 03320 | PPAR signaling pathway | Organismal Systems | Endocrine system | 0.0 | 1.0 | 1.0 | 0 | cluster\_plot | 69(11.90) | cluster\_plot |
| 247 | 00290 | Valine, leucine and isoleucine biosynthesis | Metabolism | Amino acid metabolism | 0.0 | 1.0 | 1.0 | 0 | cluster\_plot | 34(14.29) | cluster\_plot |
| 248 | 04970 | Salivary secretion | Organismal Systems | Digestive system | 0.0 | 1.0 | 1.0 | 0 | cluster\_plot | 14(1.04) | cluster\_plot |
| 249 | 00860 | Porphyrin and chlorophyll metabolism | Metabolism | Metabolism of cofactors and vitamins | 0.0 | 1.0 | 1.0 | 0 | cluster\_plot | 75(9.93) | cluster\_plot |
| 250 | 04614 | Renin-angiotensin system | Organismal Systems | Endocrine system | 0.0 | 1.0 | 1.0 | 0 | cluster\_plot | 1(3.33) | cluster\_plot |
| 251 | 04713 | Circadian entrainment | Organismal Systems | Environmental adaptation | 0.0 | 1.0 | 1.0 | 0 | cluster\_plot | 36(1.45) | cluster\_plot |
| 252 | 04962 | Vasopressin-regulated water reabsorption | Organismal Systems | Excretory system | 0.0 | 1.0 | 1.0 | 0 | cluster\_plot | 66(12.77) | cluster\_plot |
| 253 | 03008 | Ribosome biogenesis in eukaryotes | Genetic Information Processing | Translation | 0.0 | 1.0 | 1.0 | 0 | cluster\_plot | 139(61.90) | cluster\_plot |
| 254 | 00196 | Photosynthesis - antenna proteins | Metabolism | Energy metabolism | 0.0 | 1.0 | 1.0 | 0 | cluster\_plot | 28(22.73) | cluster\_plot |
| 255 | 05140 | Leishmaniasis | Human Diseases | Infectious diseases | 0.0 | 1.0 | 1.0 | 0 | cluster\_plot | 204(5.13) | cluster\_plot |
| 256 | 04726 | Serotonergic synapse | Organismal Systems | Nervous system | 0.0 | 1.0 | 1.0 | 0 | cluster\_plot | 30(3.01) | cluster\_plot |
| 257 | 04111 | Cell cycle - yeast | Cellular Processes | Cell growth and death | 0.0 | 1.0 | 1.0 | 0 | cluster\_plot | 130(24.06) | cluster\_plot |
| 258 | 00053 | Ascorbate and aldarate metabolism | Metabolism | Carbohydrate metabolism | 0.0 | 1.0 | 1.0 | 0 | cluster\_plot | 70(14.85) | cluster\_plot |
| 259 | 04260 | Cardiac muscle contraction | Organismal Systems | Circulatory system | 0.0 | 1.0 | 1.0 | 0 | cluster\_plot | 26(11.11) | cluster\_plot |
| 260 | 00906 | Carotenoid biosynthesis | Metabolism | Metabolism of terpenoids and polyketides | 0.0 | 1.0 | 1.0 | 0 | cluster\_plot | 56(8.03) | cluster\_plot |
| 261 | 00930 | Caprolactam degradation | Metabolism | Xenobiotics biodegradation and metabolism | 0.0 | 1.0 | 1.0 | 0 | cluster\_plot | 6(2.33) | cluster\_plot |
| 262 | 00900 | Terpenoid backbone biosynthesis | Metabolism | Metabolism of terpenoids and polyketides | 0.0 | 1.0 | 1.0 | 0 | cluster\_plot | 68(22.13) | cluster\_plot |
| 263 | 00532 | Glycosaminoglycan biosynthesis - chondroitin sulfate / dermatan sulfate | Metabolism | Glycan biosynthesis and metabolism | 0.0 | 1.0 | 1.0 | 0 | cluster\_plot | 15(2.94) | cluster\_plot |
| 264 | 01220 | Degradation of aromatic compounds | Metabolism | Overview | 0.0 | 1.0 | 1.0 | 0 | cluster\_plot | 31(0.47) | cluster\_plot |
| 265 | 01210 | 2-Oxocarboxylic acid metabolism | Metabolism | Overview | 0.0 | 1.0 | 1.0 | 0 | cluster\_plot | 83(8.75) | cluster\_plot |
| 266 | 04913 | Ovarian Steroidogenesis | Organismal Systems | Endocrine system | 0.0 | 1.0 | 1.0 | 0 | cluster\_plot | 8(1.03) | cluster\_plot |
| 267 | 03060 | Protein export | Genetic Information Processing | Folding, sorting and degradation | 0.0 | 1.0 | 1.0 | 0 | cluster\_plot | 72(56.10) | cluster\_plot |
| 268 | 03020 | RNA polymerase | Genetic Information Processing | Transcription | 0.0 | 1.0 | 1.0 | 0 | cluster\_plot | 65(49.09) | cluster\_plot |
| 269 | 00591 | Linoleic acid metabolism | Metabolism | Lipid metabolism | 0.0 | 1.0 | 1.0 | 0 | cluster\_plot | 29(5.88) | cluster\_plot |
| 270 | 00073 | Cutin, suberine and wax biosynthesis | Metabolism | Lipid metabolism | 0.0 | 1.0 | 1.0 | 0 | cluster\_plot | 42(14.29) | cluster\_plot |
| 271 | 04710 | Circadian rhythm | Organismal Systems | Environmental adaptation | 0.0 | 1.0 | 1.0 | 0 | cluster\_plot | 54(18.75) | cluster\_plot |
| 272 | 00460 | Cyanoamino acid metabolism | Metabolism | Metabolism of other amino acids | 0.0 | 1.0 | 1.0 | 0 | cluster\_plot | 112(8.87) | cluster\_plot |
| 273 | 05133 | Pertussis | Human Diseases | Infectious diseases | 0.0 | 1.0 | 1.0 | 0 | cluster\_plot | 232(4.72) | cluster\_plot |
| 274 | 00514 | Other types of O-glycan biosynthesis | Metabolism | Glycan biosynthesis and metabolism | 0.0 | 1.0 | 1.0 | 0 | cluster\_plot | 10(4.35) | cluster\_plot |
| 275 | 00650 | Butanoate metabolism | Metabolism | Carbohydrate metabolism | 0.0 | 1.0 | 1.0 | 0 | cluster\_plot | 32(7.59) | cluster\_plot |
| 276 | 00500 | Starch and sucrose metabolism | Metabolism | Carbohydrate metabolism | 0.0 | 1.0 | 1.0 | 0 | cluster\_plot | 369(16.92) | cluster\_plot |
| 277 | 04620 | Toll-like receptor signaling pathway | Organismal Systems | Immune system | 0.0 | 1.0 | 1.0 | 0 | cluster\_plot | 218(7.07) | cluster\_plot |
| 278 | 05160 | Hepatitis C | Human Diseases | Infectious diseases | 0.0 | 1.0 | 1.0 | 0 | cluster\_plot | 53(10.48) | cluster\_plot |
| 279 | 04540 | Gap junction | Cellular Processes | Cellular commiunity | 0.0 | 1.0 | 1.0 | 0 | cluster\_plot | 55(4.76) | cluster\_plot |
| 280 | 04960 | Aldosterone-regulated sodium reabsorption | Organismal Systems | Excretory system | 0.0 | 1.0 | 1.0 | 0 | cluster\_plot | 23(5.26) | cluster\_plot |
| 281 | 00363 | Bisphenol degradation | Metabolism | Xenobiotics biodegradation and metabolism | 0.0 | 1.0 | 1.0 | 0 | cluster\_plot | 47(2.13) | cluster\_plot |
| 282 | 04720 | Long-term potentiation | Organismal Systems | Nervous system | 0.0 | 1.0 | 1.0 | 0 | cluster\_plot | 69(13.46) | cluster\_plot |
| 283 | 04020 | Calcium signaling pathway | Environmental Information Processing | Signal transduction | 0.0 | 1.0 | 1.0 | 0 | cluster\_plot | 57(4.91) | cluster\_plot |
| 284 | 05134 | Legionellosis | Human Diseases | Infectious diseases | 0.0 | 1.0 | 1.0 | 0 | cluster\_plot | 94(15.58) | cluster\_plot |
| 285 | 00660 | C5-Branched dibasic acid metabolism | Metabolism | Carbohydrate metabolism | 0.0 | 1.0 | 1.0 | 0 | cluster\_plot | 10(6.15) | cluster\_plot |
| 286 | 00340 | Histidine metabolism | Metabolism | Amino acid metabolism | 0.0 | 1.0 | 1.0 | 0 | cluster\_plot | 34(11.22) | cluster\_plot |
| 287 | 00350 | Tyrosine metabolism | Metabolism | Amino acid metabolism | 0.0 | 1.0 | 1.0 | 0 | cluster\_plot | 51(7.85) | cluster\_plot |
| 288 | 00521 | Streptomycin biosynthesis | Metabolism | Biosynthesis of other secondary metabolites | 0.0 | 1.0 | 1.0 | 0 | cluster\_plot | 20(8.70) | cluster\_plot |
| 289 | 00020 | Citrate cycle (TCA cycle) | Metabolism | Carbohydrate metabolism | 0.0 | 1.0 | 1.0 | 0 | cluster\_plot | 83(18.81) | cluster\_plot |
| 290 | 04530 | Tight junction | Cellular Processes | Cellular commiunity | 0.0 | 1.0 | 1.0 | 0 | cluster\_plot | 40(13.04) | cluster\_plot |
| 291 | 04210 | Apoptosis | Cellular Processes | Cell growth and death | 0.0 | 1.0 | 1.0 | 0 | cluster\_plot | 199(6.90) | cluster\_plot |
| 292 | 00510 | N-Glycan biosynthesis | Metabolism | Glycan biosynthesis and metabolism | 0.0 | 1.0 | 1.0 | 0 | cluster\_plot | 68(21.64) | cluster\_plot |
| 293 | 05130 | Pathogenic Escherichia coli infection | Human Diseases | Infectious diseases | 0.0 | 1.0 | 1.0 | 0 | cluster\_plot | 49(13.56) | cluster\_plot |
| 294 | 00540 | Lipopolysaccharide biosynthesis | Metabolism | Glycan biosynthesis and metabolism | 0.0 | 1.0 | 1.0 | 0 | cluster\_plot | 6(5.56) | cluster\_plot |
| 295 | 04130 | SNARE interactions in vesicular transport | Genetic Information Processing | Folding, sorting and degradation | 0.0 | 1.0 | 1.0 | 0 | cluster\_plot | 67(37.50) | cluster\_plot |
| 296 | 04391 | Hippo signaling pathway -fly | Environmental Information Processing | Signal transduction | 0.0 | 1.0 | 1.0 | 0 | cluster\_plot | 56(7.79) | cluster\_plot |
| 297 | 00981 | Insect hormone biosynthesis | Metabolism | Metabolism of terpenoids and polyketides | 0.0 | 1.0 | 1.0 | 0 | cluster\_plot | 2(2.04) | cluster\_plot |
| 298 | 00062 | Fatty acid elongation | Metabolism | Lipid metabolism | 0.0 | 1.0 | 1.0 | 0 | cluster\_plot | 57(6.48) | cluster\_plot |
| 299 | 04973 | Carbohydrate digestion and absorption | Organismal Systems | Digestive system | 0.0 | 1.0 | 1.0 | 0 | cluster\_plot | 21(4.35) | cluster\_plot |
| 300 | 04011 | MAPK signaling pathway - yeast | Environmental Information Processing | Signal transduction | 0.0 | 1.0 | 1.0 | 0 | cluster\_plot | 16(5.56) | cluster\_plot |
| 301 | 00760 | Nicotinate and nicotinamide metabolism | Metabolism | Metabolism of cofactors and vitamins | 0.0 | 1.0 | 1.0 | 0 | cluster\_plot | 25(8.40) | cluster\_plot |
| 302 | 04014 | Ras signaling pathway | Environmental Information Processing | Signal transduction | 0.0 | 1.0 | 1.0 | 0 | cluster\_plot | 85(5.07) | cluster\_plot |
| 303 | 00281 | Geraniol degradation | Metabolism | Metabolism of terpenoids and polyketides | 0.0 | 1.0 | 1.0 | 0 | cluster\_plot | 5(2.27) | cluster\_plot |
| 304 | 05322 | Systemic lupus erythematosus | Human Diseases | Immune diseases | 0.0 | 1.0 | 1.0 | 0 | cluster\_plot | 66(8.14) | cluster\_plot |
| 305 | 05132 | Salmonella infection | Human Diseases | Infectious diseases | 0.0 | 1.0 | 1.0 | 0 | cluster\_plot | 55(10.31) | cluster\_plot |
| 306 | 00253 | Tetracycline biosynthesis | Metabolism | Metabolism of terpenoids and polyketides | 0.0 | 1.0 | 1.0 | 0 | cluster\_plot | 7(6.25) | cluster\_plot |
| 307 | 04975 | Fat digestion and absorption | Organismal Systems | Digestive system | 0.0 | 1.0 | 1.0 | 0 | cluster\_plot | 10(5.19) | cluster\_plot |
| 308 | 05143 | African trypanosomiasis | Human Diseases | Infectious diseases | 0.0 | 1.0 | 1.0 | 0 | cluster\_plot | 4(5.66) | cluster\_plot |
| 309 | 00944 | Flavone and flavonol biosynthesis | Metabolism | Biosynthesis of other secondary metabolites | 0.0 | 1.0 | 1.0 | 0 | cluster\_plot | 4(2.86) | cluster\_plot |
| 310 | 00380 | Tryptophan metabolism | Metabolism | Amino acid metabolism | 0.0 | 1.0 | 1.0 | 0 | cluster\_plot | 51(4.95) | cluster\_plot |
| 311 | 04064 | NF-kappa B signaling pathway | Environmental Information Processing | Signal transduction | 0.0 | 1.0 | 1.0 | 0 | cluster\_plot | 198(4.08) | cluster\_plot |
| 312 | 05323 | Rheumatoid arthritis | Human Diseases | Immune diseases | 0.0 | 1.0 | 1.0 | 0 | cluster\_plot | 48(17.50) | cluster\_plot |
| 313 | 00280 | Valine, leucine and isoleucine degradation | Metabolism | Amino acid metabolism | 0.0 | 1.0 | 1.0 | 0 | cluster\_plot | 66(10.29) | cluster\_plot |
| 314 | 04711 | Circadian rhythm - fly | Organismal Systems | Environmental adaptation | 0.0 | 1.0 | 1.0 | 0 | cluster\_plot | 8(5.56) | cluster\_plot |
| 315 | 04330 | Notch signaling pathway | Environmental Information Processing | Signal transduction | 0.0 | 1.0 | 1.0 | 0 | cluster\_plot | 21(30.00) | cluster\_plot |
| 316 | 03010 | Ribosome | Genetic Information Processing | Translation | 0.0 | 1.0 | 1.0 | 0 | cluster\_plot | 455(83.33) | cluster\_plot |
| 317 | 04112 | Cell cycle - Caulobacter | Cellular Processes | Cell growth and death | 0.0 | 1.0 | 1.0 | 0 | cluster\_plot | 34(13.89) | cluster\_plot |
| 318 | 00625 | Chloroalkane and chloroalkene degradation | Metabolism | Xenobiotics biodegradation and metabolism | 0.0 | 1.0 | 1.0 | 0 | cluster\_plot | 32(4.26) | cluster\_plot |
| 319 | 05214 | Glioma | Human Diseases | Cancers | 0.0 | 1.0 | 1.0 | 0 | cluster\_plot | 54(6.48) | cluster\_plot |
| 320 | 00670 | One carbon pool by folate | Metabolism | Metabolism of cofactors and vitamins | 0.0 | 1.0 | 1.0 | 0 | cluster\_plot | 35(15.62) | cluster\_plot |
| 321 | 04668 | TNF signaling pathway | Environmental Information Processing | Signal transduction | 0.0 | 1.0 | 1.0 | 0 | cluster\_plot | 34(3.36) | cluster\_plot |
| 322 | 05213 | Endometrial cancer | Human Diseases | Cancers | 0.0 | 1.0 | 1.0 | 0 | cluster\_plot | 48(15.09) | cluster\_plot |
| 323 | 05219 | Bladder cancer | Human Diseases | Cancers | 0.0 | 1.0 | 1.0 | 0 | cluster\_plot | 33(6.82) | cluster\_plot |
| 324 | 04740 | Olfactory transduction | Organismal Systems | Sensory system | 0.0 | 1.0 | 1.0 | 0 | cluster\_plot | 14(4.76) | cluster\_plot |
| 325 | 05222 | Small cell lung cancer | Human Diseases | Cancers | 0.0 | 1.0 | 1.0 | 0 | cluster\_plot | 23(10.53) | cluster\_plot |
| 326 | 04080 | Neuroactive ligand-receptor interaction | Environmental Information Processing | Signaling molecules and interaction | 0.0 | 1.0 | 1.0 | 0 | cluster\_plot | 2(0.29) | cluster\_plot |
| 327 | 00621 | Dioxin degradation | Metabolism | Xenobiotics biodegradation and metabolism | 0.0 | 1.0 | 1.0 | 0 | cluster\_plot | 7(0.98) | cluster\_plot |
| 328 | 00561 | Glycerolipid metabolism | Metabolism | Lipid metabolism | 0.0 | 1.0 | 1.0 | 0 | cluster\_plot | 127(21.74) | cluster\_plot |

---
